# Supplementary material for: Insights into the Oxidative Stress Response of Salmonella enterica serovar Enteritidis Revealed by the Next Generation Sequencing Approach
Source: Antioxidants (Basel). 2020 Sep 10;9(9):849. doi: 10.3390/antiox9090849 (PMC7555449; doi:10.3390/antiox9090849)
Supplement: Supplementary file 1 [file antioxidants-09-00849-s001.zip › antioxidants-897047-supplementary/Supplementary files descriptions.docx]

Supplementary files description

**Table S1**. List of genes showed statistically significant induction in their expression due to the H_2_O_2_ 3 mM treatment.

**Table S2**. List of genes showed statistically significant repression in their expression due to the H_2_O_2_ 3 mM treatment.
