# Supplementary material for: Insights into the Oxidative Stress Response of Salmonella enterica serovar Enteritidis Revealed by the Next Generation Sequencing Approach
Source: Antioxidants (Basel). 2020 Sep 10;9(9):849. doi: 10.3390/antiox9090849 (PMC7555449; doi:10.3390/antiox9090849)
Supplement: Supplementary file 1 [file antioxidants-09-00849-s001.zip › antioxidants-897047-supplementary/Table S1.pdf]

Feature ID	Experiment - Range (original values)	Experiment - IQR (original values)	Experiment - Difference (original values)	Experiment - Fold Change (original values)	EDGE test: WT H202 vs WT NT , tagwise dispersions - P-value	EDGE test: WT H202 vs WT NT , tagwise dispersions - Fold change	WT H202 vs WT NT ABS|FC|	WT H202 vs WT NT Log2FC	WT H202 vs WT NT Log2FC +-	EDGE test: WT H202 vs WT NT , tagwise dispersions - FDR p-value correction	WT NT - WT.1.S22 - Expression values	WT NT - WT.2.S23 - Expression values	WT NT - WT.3.S24 - Expression values	WT NT - Means	WT H202 - WT.1.H2O2 - Expression values	WT H202 - WT.2.H2O2 - Expression values	WT H202 - WT.3.H2O2 - Expression values	WT H202 - Means
SEN1808A	3	2	2.333333	#DIV/0!	0.004777	29.98048	29.98048	4.905952	4.905952	0.008798	0	0	0	0	2	2	3	2.333333
sufA	6363	3605	4952.667	438	1.52E-87	903.3434	903.3434	9.819131	9.819131	2.32E-85	13	14	7	11.33333	6370	3618	4904	4964
ycfR	7137	5513	6472.667	432.5111	6.88E-91	913.0788	913.0788	9.834596	9.834596	1.17E-88	18	14	13	15	5527	6786	7150	6487.667
sitB	11959	6835	9299.667	241.5086	7.81E-82	509.8224	509.8224	8.993851	8.993851	1.08E-79	42	37	37	38.66667	6872	9147	11996	9338.333
sufB	27553	16502	21874	171.4468	3.84E-88	358.1901	358.1901	8.484581	8.484581	6.08E-86	144	135	106	128.3333	27659	16637	21711	22002.33
sufC	11310	7968	9951.667	158.1316	2.69E-90	331.4417	331.4417	8.372611	8.372611	4.42E-88	71	67	52	63.33333	10648	8035	11362	10015
sitC	4261	2577	3402.333	130.2025	4.4E-74	275.3116	275.3116	8.104922	8.104922	5.57E-72	31	24	24	26.33333	2601	3400	4285	3428.667
SEN0540	16282	10581	13020.67	125.7987	1.11E-82	264.9638	264.9638	8.049651	8.049651	1.58E-80	121	108	84	104.3333	12320	10689	16366	13125
dps	382557	247774	336090.3	116.06	1.07E-72	248.9996	248.9996	7.96	7.96	1.31E-70	3475	2580	2708	2921	250482	381415	385137	339011.3
sufS	24899	14267	19620.67	105.7367	4.23E-69	224.9551	224.9551	7.813493	7.813493	4.93E-67	188	223	151	187.3333	14455	19919	25050	19808
sitA	18916	10754	14196.33	101.6832	1.47E-72	214.3065	214.3065	7.743532	7.743532	1.76E-70	155	131	137	141	10891	13074	19047	14337.33
iroN	11060	6560	9300.333	92.77961	2.32E-66	200.4198	200.4198	7.646881	7.646881	2.44E-64	135	91	78	101.3333	6651	10416	11138	9401.667
nrdH	70	47	56.33333	85.5	6.08E-39	144.3118	144.3118	7.173046	7.173046	3.29E-37	0	2	0	0.666667	47	54	70	57
sufD	21795	16250	18363.67	82.01618	1.94E-77	173.4557	173.4557	7.438423	7.438423	2.6E-75	253	243	184	226.6667	17299	16493	21979	18590.33
SEN1163	483	42	316.6667	74.07692	1.34E-19	156.4221	156.4221	7.2893	7.2893	2.02E-18	5	3	5	4.333333	47	486	430	321
SEN1801	15402	14365	14847.67	69.10856	3.53E-66	144.0292	144.0292	7.170218	7.170218	3.64E-64	223	160	271	218	15562	14588	15047	15065.67
sitD	4257	2549	3463	57.77049	7.46E-64	123.6429	123.6429	6.950036	6.950036	7.35E-62	74	62	47	61	2611	3657	4304	3524
ynhA	7030	3895	5473.333	53.79743	3.97E-62	114.8003	114.8003	6.842983	6.842983	3.66E-60	119	108	84	103.6667	4003	5614	7114	5577
fes	1402	1278	1322.333	51.21519	5.97E-86	108.275	108.275	6.758556	-6.75856	8.82E-84	29	32	18	26.33333	1319	1307	1420	1348.667
bfd	1325	698	1097	48.69565	2.03E-75	101.0969	101.0969	6.659595	-6.65959	2.65E-73	29	25	15	23	1297	723	1340	1120
asnA	25463	1787	15478.67	47.52906	8.79E-20	94.99261	94.99261	6.569743	6.569743	1.37E-18	396	277	325	332.6667	25740	2112	19582	15811.33
mntH	15666	6710	10176	46.63229	5.3E-49	97.36622	97.36622	6.605349	-6.60535	4.05E-47	276	230	163	223	15829	6940	8428	10399
SEN0541	147	78	104	45.57143	3.96E-48	88.92522	88.92522	6.474521	6.474521	2.98E-46	2	2	3	2.333333	90	80	149	106.3333
yjcB	1662	656	1291	42.20213	2.3E-43	89.96324	89.96324	6.491264	6.491264	1.46E-41	32	29	33	31.33333	688	1588	1691	1322.333
trxC	12939	5184	8317.667	36.54558	4.82E-44	75.94272	75.94272	6.24684	6.24684	3.19E-42	280	241	181	234	13120	5425	7110	8551.667
iroB	4250	2122	3282	35.66901	1.5E-42	77.04143	77.04143	6.267563	6.267563	9.23E-41	103	97	84	94.66667	2219	4334	3577	3376.667
fepB	3168	1882	2427.667	34.25571	1.5E-67	71.26242	71.26242	6.15507	6.15507	1.7E-65	78	74	67	73	3235	1956	2311	2500.667
ybiJ	6463	3802	5391.333	33.21912	2.13E-63	69.03002	69.03002	6.109152	6.109152	2.05E-61	169	190	143	167.3333	6606	3971	6099	5558.667
ybdZ	84	60	73	32.28571	2.53E-45	64.07987	64.07987	6.001799	6.001799	1.75E-43	3	3	1	2.333333	63	78	85	75.33333
SEN3195	3275	1617	2557.667	31.56972	5.6E-43	67.51773	67.51773	6.077195	6.077195	3.49E-41	100	73	78	83.66667	1695	2881	3348	2641.333
yehE	2225	944	1517.667	31.15232	7.85E-62	64.53932	64.53932	6.012106	6.012106	7.1E-60	67	40	44	50.33333	2265	988	1451	1568
fhuA	10919	7745	9523	31.00945	3.94E-67	65.05172	65.05172	6.023515	6.023515	4.36E-65	356	318	278	317.3333	11197	8063	10261	9840.333
fepD	2249	1489	1863.667	30.11979	1.47E-64	62.23179	62.23179	5.95958	5.95958	1.48E-62	46	86	60	64	2295	1549	1939	1927.667

Feature ID	Experiment - Range (original values)	Experiment - IQR (original values)	Experiment - Difference (original values)	Experiment - Fold Change (original values)	EDGE test: WT H202 vs WT NT , tagwise dispersions - P-value	EDGE test: WT H202 vs WT NT , tagwise dispersions - Fold change	WT H202 vs WT NT ABS|FC|	WT H202 vs WT NT Log2FC	WT H202 vs WT NT Log2FC +-	EDGE test: WT H202 vs WT NT , tagwise dispersions - FDR p-value correction	WT NT - WT.1.S22 - Expression values	WT NT - WT.2.S23 - Expression values	WT NT - WT.3.S24 - Expression values	WT NT - Means	WT H202 - WT.1.H2O2 - Expression values	WT H202 - WT.2.H2O2 - Expression values	WT H202 - WT.3.H2O2 - Expression values	WT H202 - Means
ibpB	19519	13231	17219.67	28.39077	1.45E-45	58.5387	58.5387	5.871319	5.871319	1.02E-43	487	754	645	628.6667	19663	13876	20006	17848.33
yhcN	2430	1242	1899	28.38942	6.11E-53	60.61444	60.61444	5.92159	5.92159	4.75E-51	79	68	61	69.33333	1310	2104	2491	1968.333
yjfR	4338	2357	3115.333	26.19137	9.14E-44	54.8323	54.8323	5.776954	5.776954	5.96E-42	118	134	119	123.6667	2476	2785	4456	3239
narI	14860	823	10050.33	26.10491	8.42E-14	56.85785	56.85785	5.829288	5.829288	7.31E-13	322	523	356	400.3333	1179	15182	14991	10450.67
yebF	7777	4342	5808.667	22.59356	8.15E-45	47.49016	47.49016	5.569557	5.569557	5.55E-43	343	253	211	269	7988	4595	5650	6077.667
cirA	2688	1720	2065	22.21575	9.01E-55	46.77571	46.77571	5.547688	-5.54769	7.54E-53	99	111	82	97.33333	1819	1898	2770	2162.333
ahpC	344409	193692	284212	21.96216	8.57E-37	47.50984	47.50984	5.570155	5.570155	3.96E-35	16262	13052	11361	13558.33	206744	355770	330797	297770.3
recN	20219	10814	15149	21.50857	9.32E-42	45.11494	45.11494	5.495533	-5.49553	5.58E-40	956	655	605	738.6667	20824	11469	15370	15887.67
SEN2649	144	85	109.3333	20.29412	1.88E-53	41.59217	41.59217	5.37824	5.37824	1.52E-51	7	6	4	5.666667	148	106	91	115
yebG	4633	1944	2989	19.75941	5.16E-38	41.09461	41.09461	5.360877	5.360877	2.6E-36	185	169	124	159.3333	4757	2113	2575	3148.333
ahpF	26891	24044	25554	18.93683	2.49E-44	39.98735	39.98735	5.321472	-5.32147	1.67E-42	1593	1350	1331	1424.667	27320	25394	28222	26978.67
iroC	7084	2119	4872.667	18.80512	3.87E-26	41.24102	41.24102	5.366008	5.366008	1E-24	316	280	225	273.6667	2399	7309	5731	5146.333
sodA	108839	67000	85140	17.28435	6.87E-20	36.26296	36.26296	5.180425	5.180425	1.09E-18	3089	10156	2440	5228.333	89737	70089	111279	90368.33
acnA	48392	36189	40907.33	16.6673	7.23E-34	36.04869	36.04869	5.171875	5.171875	2.97E-32	3890	2193	1750	2611	38382	42031	50142	43518.33
ibpA	28543	18328	23547.33	15.86574	1.7E-31	32.55266	32.55266	5.024704	5.024704	6.03E-30	1064	1885	1803	1584	25656	20131	29607	25131.33
iroE	1188	710	989	15.835	6.27E-46	33.70022	33.70022	5.074686	5.074686	4.56E-44	72	66	62	66.66667	776	1141	1250	1055.667
SEN0346	3360	2472	2964.333	14.83048	9.59E-42	31.37552	31.37552	4.971568	4.971568	5.67E-40	282	186	175	214.3333	3535	2658	3343	3178.667
SEN2380	115	81	95.66667	14.66667	4.45E-56	30.06288	30.06288	4.909911	4.909911	3.79E-54	6	9	6	7	121	87	100	102.6667
SEN1800	11937	8515	10086.33	14.63632	1.44E-32	31.4146	31.4146	4.973364	4.973364	5.66E-31	1152	563	504	739.6667	10959	9078	12441	10826
ydiE	801	399	589.6667	14.1037	2.96E-47	29.46467	29.46467	4.880914	4.880914	2.19E-45	48	62	25	45	826	447	631	634.6667
nrdI	71	30	48	14.09091	4.2E-26	28.76466	28.76466	4.846225	4.846225	1.08E-24	5	6	0	3.666667	35	49	71	51.66667
narJ	7747	197	4865.667	13.55116	1.36E-08	29.67416	29.67416	4.891135	4.891135	6.35E-08	313	510	340	387.6667	284	8031	7445	5253.333
SEN0542	1123	495	754.6667	13.43956	8.66E-39	28.19511	28.19511	4.817373	4.817373	4.57E-37	59	66	57	60.66667	554	712	1180	815.3333
narH	15496	463	9287	12.50805	9.24E-07	27.46261	27.46261	4.779397	-4.7794	3.25E-06	628	1091	702	807	464	15960	13858	10094
sulA	17175	6139	10627.67	12.12845	2.93E-28	25.20792	25.20792	4.655805	4.655805	8.49E-27	1142	999	724	955	17899	7138	9711	11582.67
yheR	823	637	725	11.875	1.89E-56	25.23617	25.23617	4.657421	4.657421	1.64E-54	70	80	50	66.66667	707	795	873	791.6667
entF	2006	1395	1772.667	11.59363	1.27E-38	24.95416	24.95416	4.641209	-4.64121	6.61E-37	206	166	130	167.3333	1561	2136	2123	1940
nagB	24276	11069	19063.67	11.29912	2.22E-30	23.27114	23.27114	4.54047	-4.54047	7.39E-29	1780	2100	1673	1851	25949	12849	23946	20914.67
SEN0277A	25	5	13.33333	11	5.33E-11	20.45991	20.45991	4.354728	4.354728	3.28E-10	1	1	2	1.333333	26	12	6	14.66667
SEN0716	1898	1228	1483	10.82119	9.82E-40	22.44856	22.44856	4.488551	4.488551	5.51E-38	123	186	144	151	2021	1372	1509	1634
ynhG	12295	6333	9441.333	10.78039	7.94E-27	23.34409	23.34409	4.544985	4.544985	2.13E-25	1358	798	740	965.3333	7131	11054	13035	10406.67
iagB	73	52	57.66667	10.61111	6.6E-39	21.8091	21.8091	4.446859	4.446859	3.52E-37	9	5	4	6	77	57	57	63.66667
cof	3493	2828	3122	10.366	1.63E-31	22.26445	22.26445	4.47667	4.47667	5.83E-30	475	313	212	333.3333	3705	3141	3520	3455.333

Feature ID	Experiment - Range (original values)	Experiment - IQR (original values)	Experiment - Difference (original values)	Experiment - Fold Change (original values)	EDGE test: WT H202 vs WT NT , tagwise dispersions - P-value	EDGE test: WT H202 vs WT NT , tagwise dispersions - Fold change	WT H202 vs WT NT ABS|FC|	WT H202 vs WT NT Log2FC	WT H202 vs WT NT Log2FC +-	EDGE test: WT H202 vs WT NT , tagwise dispersions - FDR p-value correction	WT NT - WT.1.S22 - Expression values	WT NT - WT.2.S23 - Expression values	WT NT - WT.3.S24 - Expression values	WT NT - Means	WT H202 - WT.1.H2O2 - Expression values	WT H202 - WT.2.H2O2 - Expression values	WT H202 - WT.3.H2O2 - Expression values	WT H202 - Means
ypeC	4587	2327	3223	10.30606	3.41E-32	21.55585	21.55585	4.430008	4.430008	1.27E-30	385	380	274	346.3333	4861	2707	3140	3569.333
yebE	5389	2998	4050.667	9.948454	1.54E-32	20.80805	20.80805	4.37907	4.37907	5.93E-31	564	379	415	452.6667	5768	3413	4329	4503.333
SEN0085	1208	716	968	9.694611	1.73E-43	20.21175	20.21175	4.337122	4.337122	1.11E-41	133	93	108	111.3333	1301	824	1113	1079.333
SEN0277	58	29	43.33333	9.666667	1.84E-30	19.5889	19.5889	4.291964	4.291964	6.21E-29	5	7	3	5	61	34	50	48.33333
SEN1469	3249	1286	2171.667	9.652058	4.14E-24	21.20334	21.20334	4.40622	4.40622	9.26E-23	308	248	197	251	1534	3446	2288	2422.667
polB	8454	4565	6677	9.585941	1.93E-32	20.07682	20.07682	4.327459	-4.32746	7.24E-31	935	771	627	777.6667	9081	5336	7947	7454.667
yshA	42	19	28.33333	9.5	1.34E-22	18.90185	18.90185	4.240456	4.240456	2.55E-21	2	4	4	3.333333	44	23	28	31.66667
ybdA	585	436	515.6667	9.099476	1.24E-59	19.01678	19.01678	4.249201	4.249201	1.1E-57	68	60	63	63.66667	645	499	594	579.3333
gpmA	67355	44703	57473.67	9.092223	1.04E-23	19.42808	19.42808	4.280071	4.280071	2.23E-22	6101	10071	5135	7102.333	50804	70434	72490	64576
ndh	11100	7123	9300.667	8.958357	5.39E-32	18.67898	18.67898	4.223344	4.223344	1.97E-30	1181	1215	1110	1168.667	10894	8304	12210	10469.33
katG	438736	298602	372278.3	8.829472	1.65E-24	18.43584	18.43584	4.204441	4.204441	3.78E-23	36782	50449	55414	47548.33	349051	434911	475518	419826.7
nifJ	7740	3102	5290.667	8.558095	7.16E-28	17.71515	17.71515	4.146912	4.146912	2.02E-26	745	762	593	700	8333	3847	5792	5990.667
ybeD	4950	3959	4482.667	8.39714	1.36E-32	17.64701	17.64701	4.141352	4.141352	5.39E-31	621	638	559	606	5177	4580	5509	5088.667
phoH	5762	3136	4251.333	8.351009	2.08E-25	17.8088	17.8088	4.154518	-4.15452	5.1E-24	866	480	389	578.3333	4722	3616	6151	4829.667
SEN1978	12	8	9.666667	8.25	1.3E-10	15.48513	15.48513	3.952811	3.952811	7.68E-10	1	1	2	1.333333	13	9	11	11
SEN0912	43	24	31	8.153846	9.26E-24	16.54067	16.54067	4.047946	4.047946	1.99E-22	6	2	5	4.333333	32	29	45	35.33333
dadA	2015	928	1418	8.043046	4.63E-34	16.75187	16.75187	4.06625	-4.06625	1.95E-32	211	239	154	201.3333	2169	1139	1550	1619.333
SEN0539	1814	1009	1364.667	7.998291	1.21E-37	16.74227	16.74227	4.065423	4.065423	5.75E-36	215	196	174	195	1486	1205	1988	1559.667
aes	946	664	767	7.930723	5.71E-42	16.59683	16.59683	4.052836	-4.05284	3.47E-40	114	113	105	110.6667	1051	777	805	877.6667
sicP	92	69	79.33333	7.611111	3.55E-41	15.89613	15.89613	3.990604	3.990604	2.07E-39	16	13	7	12	99	82	93	91.33333
narK	905	53	487.6667	7.59009	3.35E-09	16.84899	16.84899	4.07459	4.07459	1.67E-08	59	112	51	74	76	956	653	561.6667
23S rRNA-3	7	3	4.333333	7.5	9.11E-05	12.82118	12.82118	3.680458	3.680458	0.000233	2	0	0	0.666667	7	5	3	5
soxS	1098	517	801.3333	7.497297	1.16E-37	15.58899	15.58899	3.962456	3.962456	5.59E-36	138	127	105	123.3333	1203	644	927	924.6667
dinP	5239	2997	3999.333	7.426352	1.55E-28	15.63105	15.63105	3.966343	3.966343	4.58E-27	788	578	501	622.3333	5740	3575	4550	4621.667
narG	20176	524	10906.67	7.378168	2.93E-07	16.33828	16.33828	4.030184	-4.03018	1.13E-06	1584	2035	1511	1710	902	21078	15870	12616.67
yhhV	229	191	199.3333	7.361702	8.25E-46	15.55817	15.55817	3.959601	3.959601	5.89E-44	44	22	28	31.33333	251	222	219	230.6667
pdhR	3637	1508	2584.667	7.324633	5.83E-23	14.84361	14.84361	3.89177	3.89177	1.16E-21	316	414	496	408.6667	3953	1922	3105	2993.333
safA	74	21	41.66667	7.25	1.02E-13	15.42921	15.42921	3.947592	3.947592	8.8E-13	10	1	9	6.666667	30	75	40	48.33333
SEN2228	88	42	62	7.2	1.99E-23	14.88281	14.88281	3.895575	3.895575	4.1E-22	8	8	14	10	50	70	96	72
SEN0538	107	94	98.66667	7.166667	6.53E-40	15.08695	15.08695	3.915229	3.915229	3.71E-38	21	12	15	16	109	119	116	114.6667
avrA	1126	679	826	7.103448	6.07E-34	15.13876	15.13876	3.920176	-3.92018	2.52E-32	182	132	92	135.3333	855	811	1218	961.3333
SEN0537A	149	78	107.6667	7.09434	1.44E-36	14.75981	14.75981	3.883602	3.883602	6.6E-35	24	16	13	17.66667	162	94	120	125.3333
SEN4269	3	1	2	7	0.004847	10.13997	10.13997	3.341982	3.341982	0.008911	1	0	0	0.333333	1	3	3	2.333333

Feature ID	Experiment - Range (original values)	Experiment - IQR (original values)	Experiment - Difference (original values)	Experiment - Fold Change (original values)	EDGE test: WT H202 vs WT NT , tagwise dispersions - P-value	EDGE test: WT H202 vs WT NT , tagwise dispersions - Fold change	WT H202 vs WT NT ABS|FC|	WT H202 vs WT NT Log2FC	WT H202 vs WT NT Log2FC +-	EDGE test: WT H202 vs WT NT , tagwise dispersions - FDR p-value correction	WT NT - WT.1.S22 - Expression values	WT NT - WT.2.S23 - Expression values	WT NT - WT.3.S24 - Expression values	WT NT - Means	WT H202 - WT.1.H2O2 - Expression values	WT H202 - WT.2.H2O2 - Expression values	WT H202 - WT.3.H2O2 - Expression values	WT H202 - Means
SEN1013A	3	2	2	7	0.00477	10.11586	10.11586	3.338547	3.338547	0.008787	1	0	0	0.333333	2	2	3	2.333333
iroD	627	216	480.6667	6.934156	8.23E-24	14.91947	14.91947	3.899124	-3.89912	1.78E-22	80	92	71	81	296	698	691	561.6667
SEN1013B	28	19	21.66667	6.909091	4.78E-19	13.96229	13.96229	3.803464	3.803464	6.83E-18	3	6	2	3.666667	30	24	22	25.33333
entA	254	88	171.3333	6.908046	8.11E-23	15.00999	15.00999	3.907851	3.907851	1.57E-21	37	25	25	29	113	279	209	200.3333
ssaM	37	19	27.33333	6.857143	2.05E-22	13.92534	13.92534	3.799641	3.799641	3.87E-21	6	5	3	4.666667	40	24	32	32
SEN4265	2351	1338	1700.667	6.797727	2.79E-29	14.38869	14.38869	3.846863	3.846863	8.52E-28	366	283	231	293.3333	1779	1621	2582	1994
SEN1418	86	33	51.33333	6.703704	4.68E-22	13.80967	13.80967	3.787606	3.787606	8.65E-21	13	9	5	9	91	42	48	60.33333
SEN3381	577	449	480.6667	6.699605	9.86E-38	14.30581	14.30581	3.838529	3.838529	4.8E-36	123	71	59	84.33333	636	520	539	565
SEN0016	24	19	20.66667	6.636364	2.99E-18	13.47449	13.47449	3.752159	3.752159	3.94E-17	4	5	2	3.666667	23	26	24	24.33333
entB	381	87	236.3333	6.626984	2.59E-19	14.52174	14.52174	3.860142	-3.86014	3.83E-18	48	45	33	42	132	414	289	278.3333
SEN1423	20	7	13	6.571429	1.4E-12	12.87569	12.87569	3.686578	3.686578	1.08E-11	3	2	2	2.333333	22	9	15	15.33333
SEN1009	23	15	16.66667	6.555556	1.17E-15	13.16479	13.16479	3.718613	3.718613	1.23E-14	6	2	1	3	24	18	17	19.66667
SEN0440	1089	456	838.6667	6.469565	8.18E-32	13.44507	13.44507	3.749005	3.749005	2.95E-30	167	176	117	153.3333	1206	623	1147	992
nemA	11167	4193	7785	6.350515	1.24E-19	13.48902	13.48902	3.753714	3.753714	1.88E-18	1384	1641	1340	1455	5577	9636	12507	9240
SEN3371	1585	246	842.6667	6.266667	2.62E-15	12.59581	12.59581	3.654872	3.654872	2.64E-14	113	231	136	160	1698	382	928	1002.667
SEN1162	76	9	43.66667	6.24	4.5E-08	13.79085	13.79085	3.78564	3.78564	1.92E-07	15	6	4	8.333333	9	80	67	52
nifU	8315	4255	6531.667	6.208666	1.09E-21	13.1251	13.1251	3.714257	3.714257	1.95E-20	1177	1268	1317	1254	5523	8342	9492	7785.667
yehW	1089	739	910.6667	6.049908	2.47E-34	12.6555	12.6555	3.661693	3.661693	1.05E-32	172	210	159	180.3333	1114	911	1248	1091
SEN1156	2	2	1.666667	6	0.009148	8.71281	8.71281	3.123138	3.123138	0.015834	0	1	0	0.333333	2	2	2	2
SEN0280	3	1	1.666667	6	0.009191	8.70343	8.70343	3.121584	3.121584	0.015903	1	0	0	0.333333	3	1	2	2
sifB	49	22	31.33333	5.947368	2.64E-18	11.98557	11.98557	3.583227	3.583227	3.49E-17	4	4	11	6.333333	53	26	34	37.66667
dinI	1292	833	1033.667	5.922222	1.49E-34	12.35708	12.35708	3.627266	-3.62727	6.41E-33	231	182	217	210	1474	1050	1207	1243.667
bfr	2413	1279	1961.333	5.899251	9.06E-25	12.37477	12.37477	3.62933	3.62933	2.15E-23	536	334	331	400.3333	2728	1613	2744	2361.667
ssaC	65	24	45.66667	5.892857	2.42E-23	12.04089	12.04089	3.58987	3.58987	4.92E-22	11	7	10	9.333333	72	34	59	55
SEN0276	23	9	14.66667	5.888889	1.49E-12	11.65214	11.65214	3.542523	3.542523	1.13E-11	2	2	5	3	25	11	17	17.66667
envF	39	11	24.33333	5.866667	3.91E-16	11.81354	11.81354	3.562369	-3.56237	4.28E-15	6	4	5	5	43	16	29	29.33333
SEN4292	2182	1406	1835.667	5.792863	2.06E-28	12.07364	12.07364	3.593789	3.593789	6.05E-27	405	370	374	383	2552	1780	2324	2218.667
SEN0921	46	31	36.33333	5.73913	4.13E-25	11.85397	11.85397	3.567299	3.567299	1E-23	10	6	7	7.666667	42	38	52	44
yhaK	1154	184	570.6667	5.716253	6.78E-18	11.71449	11.71449	3.550222	3.550222	8.61E-17	142	130	91	121	1245	314	516	691.6667
recA	52652	18436	32774	5.652534	1.76E-19	11.64236	11.64236	3.541312	-3.54131	2.64E-18	7398	7099	6636	7044.333	59288	25535	34632	39818.33
ybdB	199	50	127	5.646341	3.53E-17	12.20124	12.20124	3.608956	3.608956	4.2E-16	25	32	25	27.33333	75	224	164	154.3333
SEN4317	475	220	349.6667	5.621145	1.01E-35	11.74555	11.74555	3.554042	3.554042	4.58E-34	87	84	56	75.66667	531	304	441	425.3333
pduJ	122	56	78.33333	5.607843	4.21E-27	11.68888	11.68888	3.547065	-3.54706	1.14E-25	24	16	11	17	133	72	81	95.33333

Feature ID	Experiment - Range (original values)	Experiment - IQR (original values)	Experiment - Difference (original values)	Experiment - Fold Change (original values)	EDGE test: WT H202 vs WT NT , tagwise dispersions - P-value	EDGE test: WT H202 vs WT NT , tagwise dispersions - Fold change	WT H202 vs WT NT ABS|FC|	WT H202 vs WT NT Log2FC	WT H202 vs WT NT Log2FC +-	EDGE test: WT H202 vs WT NT , tagwise dispersions - FDR p-value correction	WT NT - WT.1.S22 - Expression values	WT NT - WT.2.S23 - Expression values	WT NT - WT.3.S24 - Expression values	WT NT - Means	WT H202 - WT.1.H2O2 - Expression values	WT H202 - WT.2.H2O2 - Expression values	WT H202 - WT.3.H2O2 - Expression values	WT H202 - Means
asnB	8445	235	4447.667	5.580501	2.72E-09	11.20203	11.20203	3.485688	-3.48569	1.37E-08	1141	906	866	971	9311	911	6034	5418.667
entC	199	146	169.3333	5.495575	2.98E-35	11.73123	11.73123	3.552282	3.552282	1.32E-33	48	42	23	37.66667	188	211	222	207
yfhF	8460	3823	6653.333	5.389707	3.27E-19	11.51515	11.51515	3.525461	3.525461	4.79E-18	1584	1505	1458	1515.667	5328	9261	9918	8169
yfiP	2783	737	1505.333	5.384466	1.69E-18	10.9764	10.9764	3.456333	3.456333	2.28E-17	339	342	349	343.3333	3122	1079	1345	1848.667
SEN0912A	133	97	118	5.37037	8.83E-38	11.18508	11.18508	3.483504	3.483504	4.35E-36	25	30	26	27	154	123	158	145
ugtL	44	23	30.33333	5.333333	2.28E-20	10.93429	10.93429	3.450788	3.450788	3.73E-19	6	8	7	7	50	32	30	37.33333
pduK	122	72	92.66667	5.276923	1.62E-29	11.11652	11.11652	3.474634	3.474634	5.01E-28	30	15	20	21.66667	137	114	92	114.3333
SEN0338	432	287	348	5.261224	7.29E-38	11.08737	11.08737	3.470845	3.470845	3.63E-36	99	77	69	81.66667	424	364	501	429.6667
sptP	711	432	548	5.259067	1.61E-35	11.0889	11.0889	3.471044	3.471044	7.2E-34	149	133	104	128.6667	650	565	815	676.6667
entE	171	111	145.3333	5.23301	1.52E-32	11.12027	11.12027	3.47512	-3.47512	5.89E-31	39	35	29	34.33333	146	193	200	179.6667
SEN0538A	419	231	291.6667	5.127358	5.71E-32	10.81485	10.81485	3.434942	3.434942	2.07E-30	84	71	57	70.66667	309	302	476	362.3333
yejG	3794	1868	2535.333	5.065206	1.13E-20	10.49936	10.49936	3.392229	3.392229	1.87E-19	561	755	555	623.6667	4349	2429	2699	3159
pagK	685	546	618.6667	5.052402	2.47E-33	10.6206	10.6206	3.408793	3.408793	9.88E-32	177	127	154	152.6667	802	700	812	771.3333
pagC	1594	1272	1415.333	5.024645	3.14E-23	10.505	10.505	3.393004	3.393004	6.33E-22	292	339	424	351.6667	1611	1886	1804	1767
hscB	3340	1180	2581	5.011917	1.89E-16	10.74968	10.74968	3.426222	3.426222	2.1E-15	631	669	630	643.3333	1811	3970	3892	3224.333
SEN1381	6	1	4	5	6.18E-05	9.184735	9.184735	3.199238	3.199238	0.000163	1	1	1	1	6	7	2	5
SEN1358	363	237	290	4.918919	2.15E-30	10.31008	10.31008	3.365983	3.365983	7.2E-29	61	86	75	74	312	356	424	364
lysA	3244	2005	2355.667	4.853326	1.07E-19	10.35954	10.35954	3.372888	3.372888	1.65E-18	869	437	528	611.3333	2533	2687	3681	2967
hemH	2275	973	1508.667	4.84537	1.71E-24	10.09437	10.09437	3.335479	3.335479	3.91E-23	454	385	338	392.3333	2613	1358	1732	1901
pagO	223	113	153.3333	4.833333	9.96E-31	10.16384	10.16384	3.345373	3.345373	3.44E-29	53	36	31	40	177	149	254	193.3333
dnaK	55499	33923	42649.67	4.788163	1.23E-18	9.952703	9.952703	3.315088	-3.31509	1.69E-17	9033	14080	10663	11258.67	52607	44586	64532	53908.33
ssaB	21	6	11.33333	4.777778	4.31E-10	9.560949	9.560949	3.257154	3.257154	2.43E-09	4	5	0	3	21	10	12	14.33333
SEN1410	1337	215	716.6667	4.72617	1.2E-16	9.553263	9.553263	3.255994	3.255994	1.35E-15	183	200	194	192.3333	1520	409	798	909
hopD	284	146	195	4.65625	3.42E-30	9.761985	9.761985	3.287175	3.287175	1.11E-28	74	40	46	53.33333	324	192	229	248.3333
grxA	7700	5102	6038.333	4.615768	6.49E-20	9.593842	9.593842	3.262109	3.262109	1.04E-18	1436	1768	1806	1670	9136	7119	6870	7708.333
SEN1734	2227	563	1156.667	4.595855	1.91E-17	9.528987	9.528987	3.252323	3.252323	2.36E-16	411	301	253	321.6667	2480	864	1091	1478.333
SEN0278	23	5	14.33333	4.583333	5.89E-11	9.173439	9.173439	3.197463	3.197463	3.61E-10	4	4	4	4	27	9	19	18.33333
abrB	1089	498	827.3333	4.566092	1.02E-27	9.487302	9.487302	3.245998	3.245998	2.86E-26	259	229	208	232	1297	727	1154	1059.333
SEN0681	11505	5627	8349.667	4.540995	1.83E-19	9.536639	9.536639	3.253481	3.253481	2.75E-18	2478	2827	1769	2358	10744	8105	13274	10707.67
SEN2869	79	40	54	4.521739	7.79E-23	9.507521	9.507521	3.249069	3.249069	1.51E-21	25	14	7	15.33333	68	54	86	69.33333
SEN0802	188	104	143	4.487805	1.91E-32	9.351593	9.351593	3.225212	3.225212	7.23E-31	39	52	32	41	220	143	189	184
yjbE	182	71	110.3333	4.447917	1.76E-23	9.357426	9.357426	3.226112	3.226112	3.65E-22	47	32	17	32	199	103	125	142.3333
nlpD	44141	32768	36913	4.399613	1.7E-18	9.493425	9.493425	3.246929	-3.24693	2.29E-17	13659	11267	7648	10858	47489	51789	44035	47771

Feature ID	Experiment - Range (original values)	Experiment - IQR (original values)	Experiment - Difference (original values)	Experiment - Fold Change (original values)	EDGE test: WT H202 vs WT NT , tagwise dispersions - P-value	EDGE test: WT H202 vs WT NT , tagwise dispersions - Fold change	WT H202 vs WT NT ABS|FC|	WT H202 vs WT NT Log2FC	WT H202 vs WT NT Log2FC +-	EDGE test: WT H202 vs WT NT , tagwise dispersions - FDR p-value correction	WT NT - WT.1.S22 - Expression values	WT NT - WT.2.S23 - Expression values	WT NT - WT.3.S24 - Expression values	WT NT - Means	WT H202 - WT.1.H2O2 - Expression values	WT H202 - WT.2.H2O2 - Expression values	WT H202 - WT.3.H2O2 - Expression values	WT H202 - Means
osmY	1713	650	998	4.334076	6.48E-17	9.365977	9.365977	3.22743	3.22743	7.53E-16	465	261	172	299.3333	911	1096	1885	1297.333
clpA	134567	80718	112469	4.333798	4.51E-18	9.256292	9.256292	3.210434	-3.21043	5.76E-17	35347	36649	29212	33736	116065	158771	163779	146205
SEN2648	201	95	150.6667	4.29927	1.82E-32	8.965383	8.965383	3.164365	3.164365	6.97E-31	50	51	36	45.66667	237	145	207	196.3333
SEN1387	57065	24314	38229	4.282023	6.31E-18	8.935674	8.935674	3.159577	3.159577	8.03E-17	14160	10740	10044	11648	67109	35054	47468	49877
SEN4247	17266	10646	11887.33	4.278059	1.13E-12	9.376006	9.376006	3.228973	3.228973	8.8E-12	6350	2883	1646	3626.333	13529	14100	18912	15513.67
soxR	294	96	202.3333	4.263441	2.04E-25	8.80128	8.80128	3.137713	3.137713	5.02E-24	75	54	57	62	348	153	292	264.3333
SEN1977	197	111	154.6667	4.244755	6.09E-34	8.843735	8.843735	3.144656	3.144656	2.52E-32	48	53	42	47.66667	239	159	209	202.3333
SEN2750	212	40	144.3333	4.231343	1.04E-18	8.759022	8.759022	3.13077	3.13077	1.44E-17	58	47	29	44.66667	239	87	241	189
pqaA	142	30	73.66667	4.202899	5.31E-16	8.597378	8.597378	3.103897	3.103897	5.75E-15	29	16	24	23	158	54	78	96.66667
SEN1715	124	48	80.66667	4.142857	4.31E-24	8.657698	8.657698	3.113983	3.113983	9.57E-23	36	25	16	25.66667	140	73	106	106.3333
yneH	950	271	683	4.128244	9.91E-17	8.900769	8.900769	3.15393	3.15393	1.14E-15	216	243	196	218.3333	487	1146	1071	901.3333
SEN2878	2519	1892	2177.333	4.116412	1.14E-21	8.726441	8.726441	3.125393	3.125393	2.02E-20	866	653	577	698.6667	2987	2545	3096	2876
SEN0017	1008	467	717.3333	4.109827	5.34E-26	8.5916	8.5916	3.102927	3.102927	1.37E-24	285	211	196	230.6667	1204	678	962	948
yeiB	1592	906	1236.333	4.083126	2.7E-25	8.588137	8.588137	3.102345	3.102345	6.57E-24	448	435	320	401	1912	1341	1659	1637.333
SEN4026	21	8	13.33333	4.076923	1.98E-11	8.299474	8.299474	3.05302	3.05302	1.31E-10	5	5	3	4.333333	24	16	13	17.66667
SEN1538	79	62	68.66667	4.074627	1.21E-29	8.541667	8.541667	3.094518	3.094518	3.76E-28	27	20	20	22.33333	92	82	99	91
yneI	2096	741	1453.667	4.051784	1.13E-16	8.815934	8.815934	3.140113	3.140113	1.28E-15	555	499	375	476.3333	1240	2471	2079	1930
osmC	497	345	368.6667	4.038462	6.72E-24	8.686981	8.686981	3.118855	3.118855	1.47E-22	188	98	78	121.3333	452	443	575	490
yehX	436	292	337	4.026946	2.27E-30	8.485795	8.485795	3.08505	3.08505	7.52E-29	114	124	96	111.3333	406	407	532	448.3333
mig-3A	134	90	105	4	1.5E-30	8.444206	8.444206	3.077962	3.077962	5.13E-29	46	33	26	35	137	123	160	140
pagD	16	9	10	4	3.53E-09	8.041318	8.041318	3.007432	3.007432	1.76E-08	2	6	2	3.333333	18	11	11	13.33333
SEN0337	459	334	360.3333	3.986188	1.14E-28	8.509785	8.509785	3.089123	3.089123	3.4E-27	161	113	88	120.6667	449	447	547	481
msrA	2900	1549	2187.333	3.980018	1.18E-18	8.528787	8.528787	3.092341	3.092341	1.63E-17	876	747	579	734	2296	2989	3479	2921.333
yjaB	1862	1137	1501.667	3.934853	6.48E-22	8.212197	8.212197	3.037768	3.037768	1.18E-20	519	515	501	511.6667	2025	1652	2363	2013.333
pduL	142	46	87	3.932584	7.85E-20	8.19485	8.19485	3.034717	-3.03472	1.23E-18	33	25	31	29.66667	167	106	77	116.6667
SEN1423A	87	41	52.66667	3.925926	2.24E-19	8.252438	8.252438	3.04482	3.04482	3.34E-18	28	16	10	18	97	58	57	70.66667
ybaO	765	389	568	3.912821	4.32E-27	8.220697	8.220697	3.039261	3.039261	1.17E-25	243	187	155	195	920	576	793	763
sopE2	56	37	43.66667	3.911111	5.51E-24	8.198419	8.198419	3.035346	3.035346	1.21E-22	20	16	9	15	65	53	58	58.66667
SEN1360A	1729	1304	1521.667	3.902098	5.53E-22	8.227986	8.227986	3.040539	3.040539	1.01E-20	635	459	479	524.3333	2188	1783	2167	2046
sseAb	32	17	24	3.88	2.03E-17	7.989363	7.989363	2.99808	2.99808	2.49E-16	7	10	8	8.333333	33	25	39	32.33333
wcaE	34	9	23	3.875	1.29E-14	7.969638	7.969638	2.994514	2.994514	1.19E-13	11	9	4	8	38	18	37	31
hmpA	949	528	689	3.870833	5.67E-24	8.135849	8.135849	3.024293	3.024293	1.24E-22	240	307	173	240	1122	768	897	929
mgtC	77	36	51.66667	3.87037	3.69E-22	8.080118	8.080118	3.014376	3.014376	6.88E-21	24	16	14	18	66	52	91	69.66667

Feature ID	Experiment - Range (original values)	Experiment - IQR (original values)	Experiment - Difference (original values)	Experiment - Fold Change (original values)	EDGE test: WT H202 vs WT NT , tagwise dispersions - P-value	EDGE test: WT H202 vs WT NT , tagwise dispersions - Fold change	WT H202 vs WT NT ABS|FC|	WT H202 vs WT NT Log2FC	WT H202 vs WT NT Log2FC +-	EDGE test: WT H202 vs WT NT , tagwise dispersions - FDR p-value correction	WT NT - WT.1.S22 - Expression values	WT NT - WT.2.S23 - Expression values	WT NT - WT.3.S24 - Expression values	WT NT - Means	WT H202 - WT.1.H2O2 - Expression values	WT H202 - WT.2.H2O2 - Expression values	WT H202 - WT.3.H2O2 - Expression values	WT H202 - Means
SEN0342	33	19	22	3.869565	7.84E-14	8.150159	8.150159	3.026828	3.026828	6.82E-13	11	6	6	7.666667	25	39	25	29.66667
SEN1007	11	3	6.666667	3.857143	6.84E-07	7.611038	7.611038	2.928093	2.928093	2.48E-06	4	1	2	2.333333	12	5	10	9
dinF	3110	1685	2353	3.832665	7.69E-20	8.049462	8.049462	3.008892	-3.00889	1.21E-18	956	849	687	830.6667	3797	2534	3220	3183.667
SEN0029	28	10	16	3.823529	1.04E-12	7.859933	7.859933	2.974517	2.974517	8.18E-12	8	7	2	5.666667	30	18	17	21.66667
dadX	917	583	719.3333	3.791721	1.73E-22	7.90166	7.90166	2.982156	-2.98216	3.27E-21	217	339	217	257.6667	1134	800	997	977
folE	3406	1888	2776.667	3.785953	2.53E-19	7.957221	7.957221	2.992265	-2.99226	3.76E-18	1168	1018	804	996.6667	4204	2906	4210	3773.333
yfiK	327	238	258.6667	3.732394	4.05E-18	7.616137	7.616137	2.929059	2.929059	5.2E-17	68	74	142	94.66667	395	312	353	353.3333
SEN1417	53	18	30	3.727273	1.45E-14	7.668395	7.668395	2.938925	2.938925	1.32E-13	11	12	10	11	63	31	29	41
SEN2191	121	108	109.6667	3.719008	2.64E-29	7.823975	7.823975	2.967902	2.967902	8.12E-28	38	48	35	40.33333	148	146	156	150
fdoH	9129	3993	6316	3.71578	1.75E-14	7.893075	7.893075	2.980587	-2.98059	1.58E-13	2065	3012	1900	2325.667	6058	8838	11029	8641.667
gltA	85322	35509	54071	3.702919	3.39E-15	7.882442	7.882442	2.978643	2.978643	3.36E-14	23765	21119	15130	20004.67	56628	65147	100452	74075.67
phnT	395	174	293.3333	3.699387	1.24E-28	7.706689	7.706689	2.946111	2.946111	3.69E-27	117	117	92	108.6667	487	291	428	402
23S rRNA-[illegible]	148	39	71.33333	3.675	4.47E-09	7.937794	7.937794	2.988738	2.988738	2.19E-08	54	15	11	26.66667	159	87	48	98
SEN1988	5	3	2.666667	3.666667	0.001495	6.759656	6.759656	2.75695	2.75695	0.003032	3	0	0	1	5	3	3	3.666667
pduM	48	12	23	3.653846	1.42E-09	7.637323	7.637323	2.933067	2.933067	7.4E-09	17	4	5	8.666667	52	26	17	31.66667
SEN3842	38	19	27.33333	3.645161	3.88E-18	7.533055	7.533055	2.913235	2.913235	5.02E-17	12	9	10	10.33333	47	29	37	37.66667
prlC	14030	9750	11323.67	3.644069	7.64E-18	7.706881	7.706881	2.946147	2.946147	9.67E-17	4861	4303	3684	4282.667	15052	14053	17714	15606.33
spy	1250	972	990.6667	3.63709	6.37E-15	7.930229	7.930229	2.987362	2.987362	6.15E-14	657	277	193	375.6667	1407	1249	1443	1366.333
pduD	137	18	58	3.636364	8.72E-10	7.387078	7.387078	2.885004	2.885004	4.7E-09	27	13	26	22	150	46	44	80
yqjH	246	169	213	3.62963	4.2E-31	7.574884	7.574884	2.921224	2.921224	1.47E-29	79	86	78	81	324	248	310	294
ybgS	352	257	289	3.603604	1.24E-25	7.688274	7.688274	2.94266	2.94266	3.13E-24	163	92	78	111	430	349	421	400
deoB	32311	23186	25599	3.573971	5.37E-13	7.493333	7.493333	2.905608	2.905608	4.36E-12	7437	15227	7172	9945.333	36527	30623	39483	35544.33
SEN4028	58	34	46	3.555556	1.39E-23	7.399967	7.399967	2.887519	2.887519	2.94E-22	18	21	15	18	73	52	67	64
yhhA	1541	851	1104.333	3.513657	1.07E-19	7.429489	7.429489	2.893263	2.893263	1.65E-18	619	347	352	439.3333	1888	1203	1540	1543.667
SEN1157	8	1	5	3.5	3.03E-05	6.917534	6.917534	2.790258	2.790258	8.39E-05	3	2	1	2	9	9	3	7
otsB	327	142	208.6667	3.494024	1.36E-24	7.345244	7.345244	2.87681	2.87681	3.16E-23	97	86	68	83.66667	254	228	395	292.3333
ymdF	1035	419	647.3333	3.483376	1.03E-15	7.528759	7.528759	2.912412	2.912412	1.09E-14	396	193	193	260.6667	612	884	1228	908
ydiU	1586	787	1218.333	3.479647	6.18E-22	7.27882	7.27882	2.863705	2.863705	1.13E-20	522	547	405	491.3333	1991	1309	1829	1709.667
yijF	3919	2436	3116.333	3.4759	1.16E-16	7.236049	7.236049	2.855202	2.855202	1.32E-15	1105	1542	1129	1258.667	5024	3565	4536	4375
SEN1107	37	11	23	3.464286	1.7E-14	7.120036	7.120036	2.831885	2.831885	1.55E-13	9	13	6	9.333333	43	20	34	32.33333
pduT	28	15	19.66667	3.458333	2.57E-15	7.151214	7.151214	2.838188	-2.83819	2.59E-14	8	12	4	8	32	23	28	27.66667
pduQ	64	53	54.66667	3.447761	1.45E-24	7.265409	7.265409	2.861044	2.861044	3.36E-23	31	19	17	22.33333	81	72	78	77
SEN1979	11	4	7.333333	3.444444	1.02E-06	6.944498	6.944498	2.79587	2.79587	3.57E-06	2	3	4	3	7	13	11	10.33333

Feature ID	Experiment - Range (original values)	Experiment - IQR (original values)	Experiment - Difference (original values)	Experiment - Fold Change (original values)	EDGE test: WT H202 vs WT NT , tagwise dispersions - P-value	EDGE test: WT H202 vs WT NT , tagwise dispersions - Fold change	WT H202 vs WT NT ABS|FC|	WT H202 vs WT NT Log2FC	WT H202 vs WT NT Log2FC +-	EDGE test: WT H202 vs WT NT , tagwise dispersions - FDR p-value correction	WT NT - WT.1.S22 - Expression values	WT NT - WT.2.S23 - Expression values	WT NT - WT.3.S24 - Expression values	WT NT - Means	WT H202 - WT.1.H2O2 - Expression values	WT H202 - WT.2.H2O2 - Expression values	WT H202 - WT.3.H2O2 - Expression values	WT H202 - Means
SEN1359	574	233	362.6667	3.428571	8.22E-22	7.227133	7.227133	2.853423	2.853423	1.47E-20	154	166	128	149.3333	387	447	702	512
SEN3512	7	6	5.666667	3.428571	2.26E-06	6.820111	6.820111	2.769795	2.769795	7.44E-06	4	2	1	2.333333	8	8	8	8
pgtE	177	97	144	3.413408	7.8E-24	7.288331	7.288331	2.865589	-2.86559	1.69E-22	66	64	49	59.66667	161	226	224	203.6667
sinR	187	121	150	3.406417	1.04E-29	7.173191	7.173191	2.842615	2.842615	3.28E-28	77	58	52	62.33333	219	179	239	212.3333
SEN3346	418	186	301.6667	3.39418	3.81E-19	7.35618	7.35618	2.878957	2.878957	5.52E-18	175	112	91	126	476	509	298	427.6667
yiaM	10	5	6.333333	3.375	2.2E-06	6.751813	6.751813	2.755275	2.755275	7.29E-06	4	3	1	2.666667	11	8	8	9
SEN0339	393	278	328.6667	3.353222	2.71E-28	7.05183	7.05183	2.817998	2.817998	7.89E-27	154	136	129	139.6667	469	414	522	468.3333
stbA	12	5	7	3.333333	1.23E-06	6.715267	6.715267	2.747445	2.747445	4.25E-06	4	0	5	3	9	12	9	10
stbB	11	1	4.666667	3.333333	9.83E-05	6.481895	6.481895	2.696416	2.696416	0.00025	3	1	2	2	12	2	6	6.666667
SEN3510	290	139	231.6667	3.332215	2.89E-26	6.942583	6.942583	2.795472	2.795472	7.59E-25	108	102	88	99.33333	374	241	378	331
SEN1229	463	286	383	3.307229	2.81E-27	6.911528	6.911528	2.789005	2.789005	7.77E-26	183	150	165	166	583	451	613	549
SEN0803	175	110	126.6667	3.30303	1.63E-24	6.994981	6.994981	2.80632	2.80632	3.77E-23	79	42	44	55	174	154	217	181.6667
SEN3820	752	357	540.6667	3.294201	2.55E-24	6.887729	6.887729	2.784028	2.784028	5.73E-23	260	245	202	235.6667	954	602	773	776.3333
SEN4266	1384	814	993	3.291538	3.2E-21	6.981587	6.981587	2.803555	2.803555	5.47E-20	506	451	343	433.3333	1287	1265	1727	1426.333
pduN	13	11	11.33333	3.266667	5.31E-10	6.68457	6.68457	2.740835	2.740835	2.94E-09	4	6	5	5	17	16	16	16.33333
fdoG	42733	21580	30265.33	3.259619	7.66E-13	6.887399	6.887399	2.783959	2.783959	6.09E-12	11057	17477	11648	13394	33228	43960	53790	43659.33
SEN1989	6	2	3	3.25	0.001424	6.166855	6.166855	2.624535	2.624535	0.002907	3	1	0	1.333333	6	1	6	4.333333
ssrB	256	131	181.6667	3.215447	7.75E-21	6.894971	6.894971	2.785544	2.785544	1.3E-19	112	69	65	82	200	270	321	263.6667
SEN1360	440	154	278	3.206349	3.07E-20	6.790314	6.790314	2.763478	2.763478	4.96E-19	136	134	108	126	288	376	548	404
yfhP	4071	2386	3195.667	3.201378	6.48E-15	6.756611	6.756611	2.7563	2.7563	6.21E-14	1994	1078	1283	1451.667	5124	3669	5149	4647.333
ssaD	37	15	23.33333	3.1875	4.08E-15	6.60043	6.60043	2.72256	2.72256	4.03E-14	11	14	7	10.66667	44	26	32	34
ssrA	285	154	207	3.18662	1.26E-23	6.731297	6.731297	2.750884	2.750884	2.67E-22	134	79	71	94.66667	356	233	316	301.6667
nifS	7345	4918	5778.333	3.154219	1.64E-15	6.608812	6.608812	2.724391	2.724391	1.7E-14	2596	2663	2788	2682.333	7581	7860	9941	8460.667
SEN2507	126	91	104.6667	3.136054	3.43E-27	6.563493	6.563493	2.714464	2.714464	9.39E-26	50	48	49	49	174	140	147	153.6667
citB	75	47	58.33333	3.134146	6.11E-23	6.6222	6.6222	2.727311	-2.72731	1.21E-21	34	32	16	27.33333	91	79	87	85.66667
fhuC	275	137	209.3333	3.128814	1.51E-25	6.589011	6.589011	2.720062	2.720062	3.77E-24	112	111	72	98.33333	328	248	347	307.6667
rmbA	17	6	11.33333	3.125	1.61E-09	6.440295	6.440295	2.687127	2.687127	8.29E-09	7	5	4	5.333333	21	18	11	16.66667
16S rRNA-4	52	17	24	3.117647	1.88E-09	6.537813	6.537813	2.708808	2.708808	9.58E-09	22	7	5	11.33333	57	25	24	35.33333
SEN0916A	178	113	129	3.114754	2.52E-23	6.624926	6.624926	2.727904	2.727904	5.1E-22	90	50	43	61	186	163	221	190
pphB	31	6	15.33333	3.090909	2.9E-10	6.351001	6.351001	2.666984	-2.66698	1.66E-09	9	9	4	7.333333	35	15	18	22.66667
sdhD	3167	228	1608	3.082003	2.79E-08	6.187118	6.187118	2.629268	2.629268	1.24E-07	691	924	702	772.3333	3858	696	2587	2380.333
SEN2173	226	140	169	3.077869	1.03E-26	6.517672	6.517672	2.704357	2.704357	2.75E-25	97	88	59	81.33333	285	228	238	250.3333
SEN1424	87	60	65.66667	3.073684	1.09E-21	6.427682	6.427682	2.684299	2.684299	1.95E-20	26	40	29	31.66667	90	89	113	97.33333

Feature ID	Experiment - Range (original values)	Experiment - IQR (original values)	Experiment - Difference (original values)	Experiment - Fold Change (original values)	EDGE test: WT H202 vs WT NT , tagwise dispersions - P-value	EDGE test: WT H202 vs WT NT , tagwise dispersions - Fold change	WT H202 vs WT NT ABS|FC|	WT H202 vs WT NT Log2FC	WT H202 vs WT NT Log2FC +-	EDGE test: WT H202 vs WT NT , tagwise dispersions - FDR p-value correction	WT NT - WT.1.S22 - Expression values	WT NT - WT.2.S23 - Expression values	WT NT - WT.3.S24 - Expression values	WT NT - Means	WT H202 - WT.1.H2O2 - Expression values	WT H202 - WT.2.H2O2 - Expression values	WT H202 - WT.3.H2O2 - Expression values	WT H202 - Means
SEN0110A	2102	626	1235.333	3.047514	1E-15	6.333108	6.333108	2.662914	2.662914	1.06E-14	741	552	517	603.3333	2619	1178	1719	1838.667
dnaJ	4315	2098	3067.667	3.042388	1.43E-15	6.40965	6.40965	2.680246	-2.68025	1.49E-14	1665	1629	1212	1502	4455	3727	5527	4569.667
fimH	137	46	89	3.038168	2.09E-21	6.299043	6.299043	2.655133	2.655133	3.65E-20	50	42	39	43.66667	176	88	134	132.6667
exbB	3138	1124	1971.667	3.033345	1.37E-14	6.315861	6.315861	2.658979	2.658979	1.26E-13	975	1175	759	969.6667	3897	2099	2828	2941.333
SEN0034	11	7	9.333333	3	8.82E-09	6.146118	6.146118	2.619675	2.619675	4.19E-08	5	5	4	4.666667	15	15	12	14
ssaE	15	4	9.333333	3	6.79E-08	6.092957	6.092957	2.607142	2.607142	2.83E-07	5	3	6	4.666667	15	9	18	14
csgB	9	3	6	3	9.68E-06	6.074853	6.074853	2.60285	2.60285	2.88E-05	5	2	2	3	11	11	5	9
spaQ	16	0	7.333333	3	3.5E-06	6.037848	6.037848	2.594035	2.594035	1.12E-05	1	5	5	3.666667	17	11	5	11
SEN1154	7	3	3.333333	3	0.000271	5.839964	5.839964	2.54596	2.54596	0.000637	4	1	0	1.666667	7	4	4	5
SEN2799	5	1	2.666667	3	0.002444	5.740998	5.740998	2.521301	2.521301	0.004754	1	2	1	1.333333	5	6	1	4
SEN0014	7	1	2.666667	3	0.002475	5.684964	5.684964	2.507151	2.507151	0.004806	1	2	1	1.333333	8	2	2	4
rpoS	47387	33868	36131.67	2.976244	2.01E-12	6.484378	6.484378	2.696968	2.696968	1.51E-11	27158	15453	12238	18283	49321	59625	54298	54414.67
SEN0804	145	96	111.6667	2.959064	1.41E-23	6.258477	6.258477	2.645812	2.645812	2.96E-22	82	45	44	57	189	141	176	168.6667
yncJ	55	14	30.66667	2.957447	5.16E-14	6.093424	6.093424	2.607253	2.607253	4.57E-13	15	20	12	15.66667	67	29	43	46.33333
SEN0806	396	191	259.6667	2.957286	1.28E-20	6.271746	6.271746	2.648867	2.648867	2.12E-19	192	115	91	132.6667	487	306	384	392.3333
SEN2997	1571	707	1033.667	2.954001	3.32E-18	6.237058	6.237058	2.640866	2.640866	4.34E-17	670	525	392	529	1963	1232	1493	1562.667
SEN1364	18	9	11.66667	2.944444	1.2E-09	6.060239	6.060239	2.599375	2.599375	6.32E-09	7	6	5	6	15	15	23	17.66667
SEN3893	56	26	39.33333	2.934426	9.83E-19	6.087653	6.087653	2.605886	2.605886	1.37E-17	19	24	18	20.33333	74	45	60	59.66667
SEN0579	15	9	9.666667	2.933333	1.66E-08	6.031077	6.031077	2.592416	2.592416	7.66E-08	10	3	2	5	17	12	15	14.66667
SEN1361	13	7	9	2.928571	1.74E-08	5.986336	5.986336	2.581673	2.581673	8E-08	7	3	4	4.666667	14	11	16	13.66667
SEN1008	14	5	7	2.909091	1.79E-06	5.897841	5.897841	2.560187	2.560187	6.01E-06	7	1	3	3.666667	15	8	9	10.66667
yqhC	1100	581	862	2.901471	3.07E-19	6.025492	6.025492	2.591079	2.591079	4.52E-18	422	521	417	453.3333	1517	1003	1426	1315.333
sthA	66	18	40.33333	2.890625	9.6E-16	6.005677	6.005677	2.586327	2.586327	1.02E-14	26	21	17	21.33333	63	39	83	61.66667
SEN1016	336	90	177	2.88968	1.18E-16	6.076671	6.076671	2.603281	2.603281	1.33E-15	131	91	59	93.66667	395	181	236	270.6667
SEN2743	384	211	296	2.881356	6.17E-25	6.033653	6.033653	2.593032	2.593032	1.47E-23	169	163	140	157.3333	524	374	462	453.3333
rna-AM93	16	8	10	2.875	2.32E-08	5.875661	5.875661	2.554751	2.554751	1.04E-07	5	2	9	5.333333	18	15	13	15.33333
yciG	37	10	19.33333	2.870968	1.15E-11	5.914081	5.914081	2.564154	2.564154	7.83E-11	13	6	12	10.33333	43	22	24	29.66667
hscA	2863	981	2048.333	2.869486	3.58E-12	6.096835	6.096835	2.608061	-2.60806	2.61E-11	1048	1157	1082	1095.667	2063	3458	3911	3144
SEN1984	18	3	9.333333	2.866667	4.53E-07	5.968348	5.968348	2.577332	2.577332	1.69E-06	8	2	5	5	16	20	7	14.33333
SEN2201	31	6	18	2.862069	4.86E-12	5.907008	5.907008	2.562428	2.562428	3.5E-11	12	11	6	9.666667	37	17	29	27.66667
tdcG	110343	23640	51134	2.856223	1.8E-06	5.951105	5.951105	2.573158	2.573158	6.05E-06	43945	7438	31259	27547.33	117781	54899	63364	78681.33
lexA	10500	4239	7278.667	2.855068	4.52E-14	5.95968	5.95968	2.575235	2.575235	4.03E-13	4565	3804	3402	3923.667	13902	8043	11662	11202.33
SEN2624	318	217	232.6667	2.846561	5.14E-21	6.068545	6.068545	2.601351	2.601351	8.73E-20	179	102	97	126	342	319	415	358.6667

Feature ID	Experiment - Range (original values)	Experiment - IQR (original values)	Experiment - Difference (original values)	Experiment - Fold Change (original values)	EDGE test: WT H202 vs WT NT , tagwise dispersions - P-value	EDGE test: WT H202 vs WT NT , tagwise dispersions - Fold change	WT H202 vs WT NT ABS|FC|	WT H202 vs WT NT Log2FC	WT H202 vs WT NT Log2FC +-	EDGE test: WT H202 vs WT NT , tagwise dispersions - FDR p-value correction	WT NT - WT.1.S22 - Expression values	WT NT - WT.2.S23 - Expression values	WT NT - WT.3.S24 - Expression values	WT NT - Means	WT H202 - WT.1.H2O2 - Expression values	WT H202 - WT.2.H2O2 - Expression values	WT H202 - WT.3.H2O2 - Expression values	WT H202 - Means
sseJ	36	12	22.66667	2.837838	1.48E-12	5.887319	5.887319	2.557611	2.557611	1.13E-11	20	8	9	12.33333	44	21	40	35
yhcQ	290	92	188	2.825243	1.62E-21	5.922465	5.922465	2.566198	2.566198	2.84E-20	120	117	72	103	362	209	302	291
nagA	6073	1005	3678.333	2.823967	1.29E-11	5.792712	5.792712	2.534239	2.534239	8.78E-11	2017	2276	1757	2016.667	7830	3022	6233	5695
SEN2647	497	249	371	2.818627	2.41E-23	5.938072	5.938072	2.569995	2.569995	4.91E-22	238	217	157	204	654	466	605	575
SEN1362	14	2	6.666667	2.818182	7.51E-06	5.64157	5.64157	2.496097	2.496097	2.29E-05	3	5	3	3.666667	17	4	10	10.33333
SEN1543A	199	103	126	2.808612	1.1E-19	5.952325	5.952325	2.573453	2.573453	1.69E-18	99	56	54	69.66667	175	159	253	195.6667
exbD	1089	450	742.6667	2.804049	3.73E-18	5.832892	5.832892	2.544211	2.544211	4.85E-17	393	505	337	411.6667	1426	843	1194	1154.333
cyoD	2490	931	1330.667	2.802257	6.08E-06	6.086268	6.086268	2.605558	2.605558	1.88E-05	459	1390	366	738.3333	729	2622	2856	2069
fdoI	4369	1460	2746	2.79048	4.66E-11	5.947212	5.947212	2.572214	-2.57221	2.91E-10	1392	2023	1186	1533.667	2852	4432	5555	4279.667
nrdF	186	130	147.3333	2.789474	6.1E-25	5.918218	5.918218	2.565163	2.565163	1.46E-23	103	81	63	82.33333	229	211	249	229.6667
SEN3476B	21	5	10	2.764706	7.22E-08	5.669393	5.669393	2.503194	2.503194	2.98E-07	8	5	4	5.666667	25	12	10	15.66667
spaS	23	7	12.33333	2.761905	3.99E-09	5.663849	5.663849	2.501783	2.501783	1.97E-08	8	6	7	7	29	14	15	19.33333
sseBb	50	38	41	2.757143	9.81E-20	5.791177	5.791177	2.533857	2.533857	1.52E-18	30	18	22	23.33333	65	60	68	64.33333
fhuF	307	115	207.3333	2.757062	1.34E-21	5.734109	5.734109	2.519569	2.519569	2.36E-20	131	117	106	118	413	232	331	325.3333
yhcR	140	53	90	2.753247	2.95E-21	5.730739	5.730739	2.518721	2.518721	5.09E-20	54	55	45	51.33333	185	107	132	141.3333
xapA	12	5	7	2.75	9.74E-07	5.643325	5.643325	2.496545	2.496545	3.42E-06	8	1	3	4	8	12	13	11
ydeI	130	99	99	2.736842	7.95E-22	5.833071	5.833071	2.544256	2.544256	1.43E-20	82	49	40	57	170	148	150	156
wcaF	20	5	8.666667	2.733333	2.84E-07	5.616583	5.616583	2.489693	2.489693	1.09E-06	9	5	1	5	21	10	10	13.66667
SEN1986	633	287	474.3333	2.731144	6.37E-21	5.700482	5.700482	2.511084	2.511084	1.08E-19	295	284	243	274	876	571	798	748.3333
SEN1981A	28	15	20	2.714286	6.04E-12	5.73803	5.73803	2.520556	2.520556	4.31E-11	18	8	9	11.66667	24	36	35	31.66667
yjiJ	396	275	325.3333	2.703316	8.81E-23	5.677333	5.677333	2.505213	2.505213	1.69E-21	206	182	185	191	511	460	578	516.3333
clpB	31541	15717	22130.33	2.695378	3.26E-12	5.802497	5.802497	2.536674	-2.53667	2.4E-11	16397	13692	9071	13053.33	29409	35530	40612	35183.67
SEN1980	137	64	109	2.668367	2.07E-23	5.559375	5.559375	2.474923	2.474923	4.25E-22	68	68	60	65.33333	194	132	197	174.3333
SEN2870	75	23	51.66667	2.666667	9.96E-16	5.537958	5.537958	2.469354	2.469354	1.05E-14	41	25	27	31	98	50	100	82.66667
SEN1013	147	78	98.66667	2.662921	7.94E-22	5.636558	5.636558	2.494815	2.494815	1.43E-20	74	60	44	59.33333	145	138	191	158
23S rRNA	155	85	88.33333	2.65625	1.42E-09	5.75398	5.75398	2.52456	2.52456	7.4E-09	108	28	24	53.33333	179	113	133	141.6667
cyoB	8313	3363	4522.333	2.656128	3.02E-06	5.694755	5.694755	2.509634	2.509634	9.72E-06	1481	5037	1674	2730.667	3421	8544	9794	7253
SEN4008	88	55	72.33333	2.643939	7.16E-23	5.513391	5.513391	2.46294	2.46294	1.4E-21	42	46	44	44	130	99	120	116.3333
SEN0164	93	23	56.66667	2.634615	8.4E-16	5.510264	5.510264	2.462121	2.462121	9.01E-15	48	34	22	34.66667	115	57	102	91.33333
yjbJ	1326	596	922.6667	2.630153	6.49E-17	5.55136	5.55136	2.472841	2.472841	7.53E-16	716	557	425	566	1751	1153	1562	1488.667
SEN2744	502	225	356	2.61086	2.95E-21	5.458175	5.458175	2.448419	2.448419	5.09E-20	257	211	195	221	697	436	598	577
sbmC	6001	546	2696.333	2.609751	2.34E-08	5.335311	5.335311	2.415572	2.415572	1.05E-07	2068	1522	1435	1675	7436	1801	3877	4371.333
SEN0015	22	8	12.33333	2.608696	1.86E-09	5.381787	5.381787	2.428085	2.428085	9.46E-09	10	7	6	7.666667	28	15	17	20

Feature ID	Experiment - Range (original values)	Experiment - IQR (original values)	Experiment - Difference (original values)	Experiment - Fold Change (original values)	EDGE test: WT H202 vs WT NT , tagwise dispersions - P-value	EDGE test: WT H202 vs WT NT , tagwise dispersions - Fold change	WT H202 vs WT NT ABS|FC|	WT H202 vs WT NT Log2FC	WT H202 vs WT NT Log2FC +-	EDGE test: WT H202 vs WT NT , tagwise dispersions - FDR p-value correction	WT NT - WT.1.S22 - Expression values	WT NT - WT.2.S23 - Expression values	WT NT - WT.3.S24 - Expression values	WT NT - Means	WT H202 - WT.1.H2O2 - Expression values	WT H202 - WT.2.H2O2 - Expression values	WT H202 - WT.3.H2O2 - Expression values	WT H202 - Means
ydiN	24	11	12.33333	2.608696	2.01E-06	5.375918	5.375918	2.426511	2.426511	6.69E-06	16	2	5	7.666667	26	8	26	20
SEN2877	2162	1419	1706.333	2.607726	9.01E-15	5.532042	5.532042	2.467812	2.467812	8.48E-14	1290	1041	853	1061.333	2828	2460	3015	2767.667
rna-AM93	44	17	30	2.607143	8.42E-12	5.553559	5.553559	2.473413	-2.47341	5.9E-11	27	14	15	18.66667	56	58	32	48.66667
allP	22	15	17.66667	2.606061	4.93E-13	5.422791	5.422791	2.439036	-2.43904	4.03E-12	14	8	11	11	30	26	30	28.66667
ytfF	525	320	396.6667	2.601615	1.07E-20	5.485248	5.485248	2.455557	2.455557	1.78E-19	262	264	217	247.6667	609	582	742	644.3333
SEN1982	51	25	34.66667	2.6	2.25E-17	5.447125	5.447125	2.445495	2.445495	2.73E-16	27	21	17	21.66667	55	46	68	56.33333
tonB	370	83	208	2.583756	1.08E-17	5.372643	5.372643	2.425632	2.425632	1.35E-16	146	144	104	131.3333	474	227	317	339.3333
yejF	2571	1305	1799.667	2.580966	1.27E-13	5.373874	5.373874	2.425963	2.425963	1.09E-12	1088	1272	1055	1138.333	3626	2393	2795	2938
potE	14862	3535	7622	2.580017	4.36E-07	5.218617	5.218617	2.383668	-2.38367	1.63E-06	2392	4841	7239	4824	17254	11708	8376	12446
ygbA	615	135	353.3333	2.5727	5.25E-13	5.222313	5.222313	2.384689	2.384689	4.27E-12	143	270	261	224.6667	758	396	580	578
yfgJ	147	61	93.66667	2.569832	2.79E-20	5.365902	5.365902	2.423821	2.423821	4.52E-19	74	51	54	59.66667	198	115	147	153.3333
SEN4251	32	14	19.33333	2.567568	3.97E-13	5.355872	5.355872	2.421122	2.421122	3.28E-12	16	13	8	12.33333	40	27	28	31.66667
csgA	60	35	41	2.556962	9.48E-18	5.38398	5.38398	2.428673	-2.42867	1.19E-16	33	24	22	26.33333	59	61	82	67.33333
rpoH	17328	5761	12963.33	2.556783	8.47E-12	5.278183	5.278183	2.400041	2.400041	5.93E-11	8230	8874	7877	8327	25205	13991	24675	21290.33
SEN2751	658	168	410	2.54717	1.34E-12	5.346449	5.346449	2.418581	2.418581	1.03E-11	394	226	175	265	833	359	833	675
yceI	294	101	190.3333	2.539084	6.41E-15	5.505299	5.505299	2.460821	2.460821	6.18E-14	144	153	74	123.6667	245	368	329	314
SEN0271	23	1	10.66667	2.52381	6.81E-08	5.224225	5.224225	2.385217	2.385217	2.83E-07	10	10	1	7	18	11	24	17.66667
zraP	1348	478	858.6667	2.519764	7.08E-16	5.239915	5.239915	2.389543	2.389543	7.64E-15	656	521	518	565	1866	999	1406	1423.667
yjcC	306	82	171.6667	2.519174	2.85E-18	5.267454	5.267454	2.397106	2.397106	3.75E-17	136	118	85	113	391	200	263	284.6667
yccT	171	91	113.3333	2.517857	2.69E-20	5.307598	5.307598	2.408059	2.408059	4.37E-19	76	89	59	74.66667	167	167	230	188
hpaG	214	99	146.6667	2.517241	4.67E-22	5.297063	5.297063	2.405193	2.405193	8.65E-21	108	103	79	96.66667	235	202	293	243.3333
SEN1171A	28	5	14.66667	2.517241	7.77E-10	5.216612	5.216612	2.383113	2.383113	4.25E-09	12	11	6	9.666667	23	16	34	24.33333
SEN0037	43	23	33.33333	2.515152	2.1E-17	5.242603	5.242603	2.390283	2.390283	2.55E-16	24	20	22	22	58	45	63	55.33333
ydhM	783	259	555.6667	2.514078	4.25E-17	5.256489	5.256489	2.3941	2.3941	5.01E-16	425	383	293	367	1076	642	1050	922.6667
SEN1392	96	53	63	2.512	3.15E-19	5.301197	5.301197	2.406318	2.406318	4.62E-18	59	35	31	41.66667	127	88	99	104.6667
pduO	49	14	26.66667	2.509434	5.71E-13	5.192704	5.192704	2.376486	2.376486	4.62E-12	20	13	20	17.66667	62	34	37	44.33333
ydjM	257	63	141.6667	2.507092	1.99E-17	5.211088	5.211088	2.381585	2.381585	2.45E-16	108	96	78	94	335	159	213	235.6667
mdlA	841	300	546.3333	2.50367	4.42E-17	5.205284	5.205284	2.379977	-2.37998	5.2E-16	367	407	316	363.3333	1157	667	905	909.6667
dcoC	6	3	4	2.5	0.000106	5.022561	5.022561	2.328423	2.328423	0.000268	3	4	1	2.666667	7	7	6	6.666667
SEN1917	40	8	15	2.5	6.27E-07	5.179276	5.179276	2.37275	2.37275	2.29E-06	17	9	4	10	44	13	18	25
SEN0280A	5	2	3	2.5	0.000942	4.91941	4.91941	2.298485	2.298485	0.002001	2	1	3	2	5	4	6	5
SEN2392	2300	757	1304	2.496557	6.21E-13	5.240157	5.240157	2.38961	2.38961	5E-12	1052	886	676	871.3333	2976	1643	1907	2175.333
foxA	1000	623	708.6667	2.494027	1.03E-17	5.305382	5.305382	2.407457	2.407457	1.29E-16	595	448	380	474.3333	1098	1071	1380	1183

Feature ID	Experiment - Range (original values)	Experiment - IQR (original values)	Experiment - Difference (original values)	Experiment - Fold Change (original values)	EDGE test: WT H202 vs WT NT , tagwise dispersions - P-value	EDGE test: WT H202 vs WT NT , tagwise dispersions - Fold change	WT H202 vs WT NT ABS|FC|	WT H202 vs WT NT Log2FC	WT H202 vs WT NT Log2FC +-	EDGE test: WT H202 vs WT NT , tagwise dispersions - FDR p-value correction	WT NT - WT.1.S22 - Expression values	WT NT - WT.2.S23 - Expression values	WT NT - WT.3.S24 - Expression values	WT NT - Means	WT H202 - WT.1.H2O2 - Expression values	WT H202 - WT.2.H2O2 - Expression values	WT H202 - WT.3.H2O2 - Expression values	WT H202 - Means
SEN0328	91	57	71.66667	2.493056	5.53E-19	5.243591	5.243591	2.390555	2.390555	7.83E-18	44	53	47	48	104	120	135	119.6667
cyoA	2511	1243	1442.333	2.481849	2.55E-07	5.236021	5.236021	2.388471	2.388471	9.85E-07	528	1771	621	973.3333	1864	2344	3039	2415.667
cyoC	2695	1264	1403	2.473739	2.62E-05	5.363624	5.363624	2.423208	2.423208	7.32E-05	544	1808	504	952	839	3027	3199	2355
SEN4264	63	28	46.66667	2.473684	1.3E-18	5.14722	5.14722	2.363793	2.363793	1.78E-17	35	29	31	31.66667	92	59	84	78.33333
phnS	106	25	59.33333	2.447154	1.32E-14	5.098485	5.098485	2.350068	2.350068	1.22E-13	56	36	31	41	137	61	103	100.3333
fpr	3396	964	2091	2.444726	2.74E-12	5.07473	5.07473	2.343331	-2.34333	2.04E-11	1532	1525	1285	1447.333	4681	2489	3445	3538.333
SEN2133	66	20	45.66667	2.442105	6.11E-16	5.063599	5.063599	2.340163	2.340163	6.6E-15	36	29	30	31.66667	87	50	95	77.33333
SEN0329	17	6	7.666667	2.4375	2.31E-06	4.96718	4.96718	2.312427	2.312427	7.6E-06	4	10	2	5.333333	19	7	13	13
SEN1173	6	4	3.333333	2.428571	0.000748	4.811435	4.811435	2.266467	2.266467	0.001619	1	1	5	2.333333	5	5	7	5.666667
SEN4248	15	2	8	2.411765	1.52E-06	4.929341	4.929341	2.301395	2.301395	5.16E-06	6	8	3	5.666667	18	7	16	13.66667
ygaM	469	230	330	2.410256	4.38E-19	5.086301	5.086301	2.346617	2.346617	6.3E-18	306	212	184	234	653	442	597	564
ynaJ	2257	1199	1624	2.398393	1.77E-12	5.143264	5.143264	2.362684	2.362684	1.33E-11	1434	1132	918	1161.333	2331	2850	3175	2785.333
ilvC	2674	534	1516	2.391251	1.54E-10	5.137216	5.137216	2.360987	2.360987	9.05E-10	1285	1193	791	1089.667	1727	2625	3465	2605.667
celB	6484	397	2363.667	2.385231	3.74E-06	4.791505	4.791505	2.260479	2.260479	1.19E-05	1638	1939	1542	1706.333	7794	1310	3106	4070
mscL	1321	261	804.6667	2.374715	7.56E-12	5.056555	5.056555	2.338155	2.338155	5.34E-11	605	619	532	585.3333	866	1451	1853	1390
sdhC	5916	315	2095.667	2.370314	1.75E-05	4.722944	4.722944	2.239686	2.239686	5.03E-05	1402	1717	1469	1529.333	6774	858	3243	3625
SEN2227	32	27	25	2.363636	1.74E-14	4.982541	4.982541	2.316882	2.316882	1.57E-13	27	13	15	18.33333	42	43	45	43.33333
yccV	1471	625	1009.667	2.359515	8.01E-15	4.932884	4.932884	2.302432	2.302432	7.59E-14	869	697	662	742.6667	2133	1322	1802	1752.333
pegD	81	50	58	2.359375	4.75E-19	4.989253	4.989253	2.318824	2.318824	6.81E-18	59	38	31	42.66667	112	88	102	100.6667
yncC	30	7	16.66667	2.351351	8.83E-11	4.868351	4.868351	2.283433	2.283433	5.34E-10	14	13	10	12.33333	40	20	27	29
rpsV	3002	1708	2134.333	2.344886	8.74E-12	4.977554	4.977554	2.315437	2.315437	6.11E-11	2197	1332	1232	1587	4234	3040	3890	3721.333
yiaO	26	13	17	2.342105	1.66E-10	4.893914	4.893914	2.290989	2.290989	9.7E-10	10	19	9	12.66667	35	31	23	29.66667
ybiU	233	95	152.6667	2.331395	2.56E-17	4.991943	4.991943	2.319601	2.319601	3.1E-16	140	122	82	114.6667	217	270	315	267.3333
SEN0906	47	24	29.66667	2.328358	2.31E-14	4.884953	4.884953	2.288345	2.288345	2.07E-13	28	20	19	22.33333	46	44	66	52
SEN1918	43	1	15	2.323529	2.47E-06	4.801535	4.801535	2.263496	2.263496	8.07E-06	14	14	6	11.33333	49	17	13	26.33333
SEN2879	57	18	30.66667	2.314286	4.4E-12	4.809286	4.809286	2.265823	2.265823	3.19E-11	26	22	22	23.33333	79	43	40	54
yohG	75	44	53.66667	2.308943	3.58E-19	4.847161	4.847161	2.27714	2.27714	5.2E-18	47	40	36	41	111	84	89	94.66667
yigI	29	11	20	2.304348	1.13E-10	4.842197	4.842197	2.275662	2.275662	6.73E-10	13	19	14	15.33333	39	42	25	35.33333
SEN0501	58	22	36	2.301205	1.83E-15	4.79247	4.79247	2.260769	2.260769	1.89E-14	32	26	25	27.66667	83	48	60	63.66667
SEN0917	76	38	53.66667	2.298387	1.18E-18	4.816447	4.816447	2.267969	2.267969	1.63E-17	47	40	37	41.33333	94	78	113	95
yljA	4344	2552	3295.333	2.295166	5.73E-12	4.88485	4.88485	2.288314	2.288314	4.09E-11	2778	2789	2066	2544.333	5330	5779	6410	5839.667
fhuE	248	105	158	2.288043	4.22E-18	4.846952	4.846952	2.277078	2.277078	5.41E-17	145	126	97	122.6667	345	266	231	280.6667
SEN3186	5	1	3	2.285714	0.001785	4.537255	4.537255	2.18182	2.18182	0.003574	2	3	2	2.333333	7	3	6	5.333333

Feature ID	Experiment - Range (original values)	Experiment - IQR (original values)	Experiment - Difference (original values)	Experiment - Fold Change (original values)	EDGE test: WT H202 vs WT NT , tagwise dispersions - P-value	EDGE test: WT H202 vs WT NT , tagwise dispersions - Fold change	WT H202 vs WT NT ABS|FC|	WT H202 vs WT NT Log2FC	WT H202 vs WT NT Log2FC +-	EDGE test: WT H202 vs WT NT , tagwise dispersions - FDR p-value correction	WT NT - WT.1.S22 - Expression values	WT NT - WT.2.S23 - Expression values	WT NT - WT.3.S24 - Expression values	WT NT - Means	WT H202 - WT.1.H2O2 - Expression values	WT H202 - WT.2.H2O2 - Expression values	WT H202 - WT.3.H2O2 - Expression values	WT H202 - Means
ssaV	50	39	40.66667	2.284211	3.04E-17	4.808736	4.808736	2.265658	2.265658	3.64E-16	39	25	31	31.66667	72	70	75	72.33333
yiiG	13	3	6.333333	2.266667	3.39E-06	4.654511	4.654511	2.21863	2.21863	1.08E-05	7	6	2	5	15	9	10	11.33333
acs	629	375	477	2.253065	1.89E-16	4.781951	4.781951	2.257599	-2.2576	2.1E-15	420	434	288	380.6667	861	795	917	857.6667
yqgA	228	94	147.3333	2.252125	9.1E-20	4.736338	4.736338	2.243772	2.243772	1.42E-18	141	119	93	117.6667	321	213	261	265
SEN0327	339	186	235	2.25	3.96E-18	4.7241	4.7241	2.240039	2.240039	5.09E-17	198	185	181	188	378	371	520	423
SEN1415	41	5	21.66667	2.25	4.64E-10	4.610267	4.610267	2.20485	2.20485	2.59E-09	14	21	17	17.33333	55	22	40	39
ldhA	2185	142	1248.667	2.243279	1.51E-08	4.796295	4.796295	2.26192	-2.26192	7.01E-08	1018	1076	919	1004.333	1160	2495	3104	2253
gcd	682	38	332	2.22963	3.58E-08	4.896134	4.896134	2.291643	-2.29164	1.56E-07	285	294	231	270	256	913	637	602
SEN0101	26	4	14.33333	2.228571	2.04E-08	4.554335	4.554335	2.18724	2.18724	9.27E-08	11	9	15	11.66667	35	14	29	26
SEN1512	463	300	311.6667	2.227034	1.38E-13	4.829628	4.829628	2.271912	2.271912	1.18E-12	391	213	158	254	513	563	621	565.6667
ompX	59320	12504	23151	2.220744	5.13E-05	5.010396	5.010396	2.324925	2.324925	0.000137	23557	22284	11053	18964.67	10624	69944	45779	42115.67
yjiN	395	38	207	2.220039	1.44E-12	4.541711	4.541711	2.183236	2.183236	1.11E-11	176	167	166	169.6667	561	205	364	376.6667
SEN3572	66	14	40.66667	2.22	4.31E-12	4.604438	4.604438	2.203025	2.203025	3.13E-11	43	29	28	33.33333	87	41	94	74
SEN3372	188	36	90.66667	2.219731	3.71E-10	4.518761	4.518761	2.175927	2.175927	2.1E-09	59	100	64	74.33333	247	81	167	165
SEN0854	713	380	434.6667	2.216418	5.8E-11	4.773823	4.773823	2.255145	2.255145	3.57E-10	599	256	217	357.3333	810	636	930	792
SEN2644	2857	1068	1664	2.21165	1.06E-09	4.672982	4.672982	2.224344	2.224344	5.64E-09	2061	1007	1052	1373.333	3864	2120	3128	3037.333
fepC	359	177	246.3333	2.197731	1.42E-18	4.635641	4.635641	2.212769	2.212769	1.94E-17	245	200	172	205.6667	448	377	531	452
SEN2322	18330	11008	7218.333	2.194693	0.004541	4.87539	4.87539	2.285518	2.285518	0.008383	3151	14159	816	6042	3266	19146	17369	13260.33
yqaE	53	16	28.66667	2.194444	2.6E-10	4.590264	4.590264	2.198577	2.198577	1.5E-09	37	21	14	24	67	30	61	52.66667
SEN0532	12	3	6.333333	2.1875	2.25E-05	4.451085	4.451085	2.154157	2.154157	6.34E-05	7	5	4	5.333333	15	4	16	11.66667
rna-AM93	38	14	21.33333	2.185185	1.45E-12	4.594346	4.594346	2.19986	2.19986	1.11E-11	24	19	11	18	49	36	33	39.33333
pduP	94	43	60.66667	2.174194	5.78E-17	4.54697	4.54697	2.184906	2.184906	6.73E-16	54	52	49	51.66667	143	99	95	112.3333
ybbY	63	41	49.66667	2.164063	3.7E-18	4.537663	4.537663	2.18195	2.18195	4.82E-17	48	38	42	42.66667	101	83	93	92.33333
yeaR	46	9	22	2.157895	1.16E-09	4.510409	4.510409	2.173258	2.173258	6.11E-09	21	20	16	19	29	32	62	41
SEN0744A	13	7	7.333333	2.157895	1.47E-06	4.468186	4.468186	2.159689	2.159689	5.02E-06	6	11	2	6.333333	13	13	15	13.66667
yjjQ	17	10	7.666667	2.15	4.96E-06	4.452809	4.452809	2.154716	2.154716	1.55E-05	14	4	2	6.666667	19	7	17	14.33333
sdhA	6492	1290	3602	2.146404	3.32E-07	4.368455	4.368455	2.127123	2.127123	1.26E-06	2736	4026	2664	3142	8247	2829	9156	6744
SEN1971A	21	9	10.66667	2.142857	2.61E-07	4.435321	4.435321	2.149038	2.149038	1.01E-06	16	5	7	9.333333	26	11	23	20
SEN1335	51	33	37	2.132653	4.6E-15	4.443036	4.443036	2.151546	2.151546	4.51E-14	30	31	37	32.66667	81	64	64	69.66667
deoD	9286	6090	6017.333	2.130653	1.64E-08	4.47219	4.47219	2.160982	-2.16098	7.58E-08	3953	7927	4086	5322	10603	10176	13239	11339.33
SEN2554	40	13	20.33333	2.12963	2.56E-11	4.476861	4.476861	2.162488	2.162488	1.65E-10	25	19	10	18	50	33	32	38.33333
SEN2975	106	39	72.66667	2.129534	1.61E-18	4.449004	4.449004	2.153482	2.153482	2.18E-17	69	68	56	64.33333	162	107	142	137
eutC	151	119	76.33333	2.122549	1.94E-05	4.754853	4.754853	2.249401	2.249401	5.53E-05	154	29	21	68	113	148	172	144.3333

Feature ID	Experiment - Range (original values)	Experiment - IQR (original values)	Experiment - Difference (original values)	Experiment - Fold Change (original values)	EDGE test: WT H202 vs WT NT , tagwise dispersions - P-value	EDGE test: WT H202 vs WT NT , tagwise dispersions - Fold change	WT H202 vs WT NT ABS|FC|	WT H202 vs WT NT Log2FC	WT H202 vs WT NT Log2FC +-	EDGE test: WT H202 vs WT NT , tagwise dispersions - FDR p-value correction	WT NT - WT.1.S22 - Expression values	WT NT - WT.2.S23 - Expression values	WT NT - WT.3.S24 - Expression values	WT NT - Means	WT H202 - WT.1.H2O2 - Expression values	WT H202 - WT.2.H2O2 - Expression values	WT H202 - WT.3.H2O2 - Expression values	WT H202 - Means
hpaE	377	117	211.6667	2.117958	3.86E-11	4.621669	4.621669	2.208414	2.208414	2.43E-10	286	169	113	189.3333	282	431	490	401
SEN4084	15	2	6.333333	2.117647	1.8E-05	4.381341	4.381341	2.131372	2.131372	5.15E-05	8	6	3	5.666667	18	12	6	12
yifZ	194	36	108.6667	2.116438	1.74E-14	4.401744	4.401744	2.138075	2.138075	1.57E-13	123	89	80	97.33333	274	125	219	206
yaiU	1752	1455	1440.667	2.115067	1.88E-11	4.485685	4.485685	2.165328	2.165328	1.25E-10	1570	1080	1226	1292	2832	2685	2681	2732.667
ybeR	26	7	13	2.114286	7.11E-09	4.391196	4.391196	2.134614	2.134614	3.43E-08	17	10	8	11.66667	34	16	24	24.66667
SEN0973	299	138	212.3333	2.109756	3.12E-18	4.434685	4.434685	2.148832	2.148832	4.1E-17	218	192	164	191.3333	418	330	463	403.6667
wcaG	48	20	31	2.107143	3.2E-11	4.507703	4.507703	2.172392	2.172392	2.03E-10	39	21	24	28	44	69	64	59
SEN4027	38	10	21.33333	2.103448	2.9E-12	4.382928	4.382928	2.131895	2.131895	2.15E-11	24	19	15	19.33333	53	29	40	40.66667
SEN3185	39	21	26	2.098592	3.41E-12	4.469253	4.469253	2.160034	2.160034	2.5E-11	32	22	17	23.66667	43	56	50	49.66667
yaaA	2140	1222	1548	2.091165	1.99E-11	4.43402	4.43402	2.148615	2.148615	1.31E-10	1761	1353	1142	1418.667	3043	2575	3282	2966.667
SEN1183	252	80	133.6667	2.080863	2.02E-14	4.325727	4.325727	2.112943	2.112943	1.82E-13	136	113	122	123.6667	365	202	205	257.3333
23S rRNA-(	871	531	371.3333	2.078412	0.000533	4.628201	4.628201	2.210451	2.210451	0.001184	854	77	102	344.3333	948	566	633	715.6667
pipB	36	18	24	2.074627	7.75E-14	4.351434	4.351434	2.121491	-2.12149	6.76E-13	29	21	17	22.33333	53	39	47	46.33333
stfE	8	3	5	2.071429	3.34E-05	4.228154	4.228154	2.080028	2.080028	9.17E-05	2	6	6	4.666667	10	9	10	9.666667
hilA	51	26	37	2.067308	9.1E-16	4.3354	4.3354	2.116165	2.116165	9.7E-15	44	31	29	34.66667	80	57	78	71.66667
ubiC	1528	275	853.6667	2.064422	6.47E-12	4.294491	4.294491	2.102487	2.102487	4.6E-11	881	858	667	802	2195	1133	1639	1655.667
SEN0081	33	14	22.33333	2.063492	2.65E-11	4.383602	4.383602	2.132117	2.132117	1.71E-10	27	20	16	21	34	49	47	43.33333
mutM	576	179	339	2.062696	6.53E-13	4.264263	4.264263	2.092296	2.092296	5.24E-12	262	349	346	319	611	525	838	658
cyoE	5573	3135	2510.667	2.054754	0.000105	4.430382	4.430382	2.147431	2.147431	0.000267	1377	4512	1252	2380.333	2029	5819	6825	4891
SEN0385	24375	11240	16283.67	2.039206	3.61E-10	4.292959	4.292959	2.101973	2.101973	2.05E-09	18969	14765	13274	15669.33	37649	26005	32205	31953
ptrB	2105	534	1196	2.034602	1.02E-10	4.242683	4.242683	2.084977	2.084977	6.13E-10	1328	1149	991	1156	3096	1683	2277	2352
slyB	3603	2441	2809.667	2.029182	5.86E-11	4.299023	4.299023	2.104009	2.104009	3.6E-10	3091	2749	2350	2730	5476	5190	5953	5539.667
sipD	93	13	54.33333	2.025157	3.41E-14	4.210522	4.210522	2.073999	2.073999	3.05E-13	60	56	43	53	136	69	117	107.3333
SEN2610	612	304	386	2.014011	1.13E-14	4.251891	4.251891	2.088105	2.088105	1.06E-13	465	359	318	380.6667	930	663	707	766.6667
SEN1759	94	9	43	2.007813	3.81E-09	4.087541	4.087541	2.031233	2.031233	1.89E-08	35	51	42	42.66667	129	44	84	85.66667
yggR	168	77	102.6667	2.006536	1.51E-16	4.250128	4.250128	2.087506	2.087506	1.7E-15	120	106	80	102	183	183	248	204.6667
SEN1635	15	8	10	2	6.17E-08	4.142963	4.142963	2.050663	2.050663	2.59E-07	8	9	13	10	23	20	17	20
SEN1983	14	6	8.333333	2	5.26E-07	4.134129	4.134129	2.047583	2.047583	1.95E-06	8	6	11	8.333333	20	16	14	16.66667
SEN1171	7	4	3	2	0.001314	4.054642	4.054642	2.019575	2.019575	0.002699	7	0	2	3	6	5	7	6
SEN1006	4	4	2.333333	2	0.004429	4.005642	4.005642	2.002034	2.002034	0.008205	5	1	1	2.333333	5	4	5	4.666667
SEN4085	8	1	2.666667	2	0.005588	3.994074	3.994074	1.997861	1.997861	0.010145	2	4	2	2.666667	10	3	3	5.333333
SEN1171E	6	2	2	2	0.007205	3.963115	3.963115	1.986635	1.986635	0.012755	4	1	1	2	7	3	2	4
SEN2135	6	1	1.666667	2	0.029453	3.894474	3.894474	1.961429	1.961429	0.045937	2	2	1	1.666667	7	1	2	3.333333

Feature ID	Experiment - Range (original values)	Experiment - IQR (original values)	Experiment - Difference (original values)	Experiment - Fold Change (original values)	EDGE test: WT H202 vs WT NT , tagwise dispersions - P-value	EDGE test: WT H202 vs WT NT , tagwise dispersions - Fold change	WT H202 vs WT NT ABS|FC|	WT H202 vs WT NT Log2FC	WT H202 vs WT NT Log2FC +-	EDGE test: WT H202 vs WT NT , tagwise dispersions - FDR p-value correction	WT NT - WT.1.S22 - Expression values	WT NT - WT.2.S23 - Expression values	WT NT - WT.3.S24 - Expression values	WT NT - Means	WT H202 - WT.1.H2O2 - Expression values	WT H202 - WT.2.H2O2 - Expression values	WT H202 - WT.3.H2O2 - Expression values	WT H202 - Means
SEN0786	5	1	1.333333	2	0.028435	3.845419	3.845419	1.943141	1.943141	0.044443	1	3	0	1.333333	5	2	1	2.666667
livK	154	110	119.3333	1.994444	2.01E-17	4.188257	4.188257	2.06635	-2.06635	2.46E-16	114	134	112	120	266	228	224	239.3333
luxS	3938	3054	3311.333	1.982009	1.2E-10	4.183586	4.183586	2.06474	-2.06474	7.12E-10	3828	3067	3221	3372	6770	6275	7005	6683.333
SEN0829	22	4	15	1.978261	1.34E-09	4.092737	4.092737	2.033066	2.033066	6.99E-09	16	15	15	15.33333	35	19	37	30.33333
SEN2553	69	47	53.33333	1.97561	2.81E-17	4.1766	4.1766	2.062329	2.062329	3.37E-16	64	56	44	54.66667	113	103	108	108
yceP	12610	8196	9134	1.965948	1.22E-09	4.177283	4.177283	2.062565	2.062565	6.4E-09	12562	8101	7705	9456	20315	16297	19158	18590
pduH	19	5	9	1.964286	6.42E-07	4.063315	4.063315	2.022657	2.022657	2.34E-06	12	8	8	9.333333	27	13	15	18.33333
ssaJ	19	3	8.666667	1.962963	6.74E-07	4.085654	4.085654	2.030567	2.030567	2.45E-06	12	9	6	9	25	16	12	17.66667
SEN0816	891	216	413.3333	1.960496	9.48E-11	4.110897	4.110897	2.039453	2.039453	5.71E-10	590	374	327	430.3333	1218	561	752	843.6667
ubiA	1359	280	808.6667	1.95738	1.05E-11	4.075233	4.075233	2.026882	2.026882	7.16E-11	900	916	718	844.6667	2077	1180	1703	1653.333
lpfA	88	27	43.33333	1.955882	3.36E-12	4.087943	4.087943	2.031375	2.031375	2.47E-11	59	37	40	45.33333	125	67	74	88.66667
SEN1470	25	16	21	1.954545	5.62E-12	4.106759	4.106759	2.038	2.038	4.03E-11	22	23	21	22	45	46	38	43
SEN3650	157	114	122.6667	1.950904	4.54E-17	4.127955	4.127955	2.045427	2.045427	5.32E-16	164	109	114	129	261	228	266	251.6667
fepG	291	183	221	1.949857	1.65E-15	4.073614	4.073614	2.026309	2.026309	1.7E-14	207	273	218	232.6667	498	401	462	453.6667
SEN0032	93	29	54.33333	1.947674	2.57E-13	4.121266	4.121266	2.043087	2.043087	2.15E-12	83	54	35	57.33333	125	82	128	111.6667
ssaK	10	4	6	1.947368	5.81E-06	4.018444	4.018444	2.006637	2.006637	1.8E-05	8	4	7	6.333333	12	11	14	12.33333
aceE	25220	13891	12147.67	1.944756	3.01E-06	4.241309	4.241309	2.08451	-2.08451	9.7E-06	23723	7743	7108	12858	21634	21055	32328	25005.67
sifA	118	47	70	1.941704	1.06E-14	4.088114	4.088114	2.031436	2.031436	9.93E-14	91	59	73	74.33333	177	136	120	144.3333
uspB	1021	622	803.3333	1.935196	1.23E-12	4.089289	4.089289	2.03185	2.03185	9.55E-12	967	878	732	859	1734	1500	1753	1662.333
SEN0478	135	21	70	1.933333	1.38E-12	4.012177	4.012177	2.004385	2.004385	1.06E-11	74	95	56	75	191	93	151	145
ygaP	1147	256	612.3333	1.928716	9.75E-11	4.055008	4.055008	2.019705	2.019705	5.85E-10	879	623	476	659.3333	1382	810	1623	1271.667
pduU	23	6	8.666667	1.928571	7.4E-06	3.972341	3.972341	1.98999	1.98999	2.26E-05	7	15	6	9.333333	29	13	12	18
eutB	359	232	167.6667	1.924632	1.58E-05	4.257665	4.257665	2.090063	-2.09006	4.57E-05	392	68	84	181.3333	304	316	427	349
pegC	68	32	39	1.92126	1.94E-13	4.080428	4.080428	2.028721	2.028721	1.64E-12	61	40	26	42.33333	94	72	78	81.33333
phnU	40	15	23	1.92	8.78E-12	4.003964	4.003964	2.001429	2.001429	6.13E-11	23	33	19	25	59	38	47	48
nirD	919	103	215.6667	1.919034	0.001457	4.338566	4.338566	2.117218	-2.11722	0.002963	143	357	204	234.6667	93	1012	246	450.3333
SEN4246	51	31	37	1.917355	3.34E-15	4.040654	4.040654	2.014589	2.014589	3.33E-14	51	37	33	40.33333	80	68	84	77.33333
SEN0330	27	4	10.33333	1.911765	2.24E-06	3.966053	3.966053	1.987704	1.987704	7.38E-06	15	15	4	11.33333	31	11	23	21.66667
stbC	300	172	196.3333	1.899237	2.01E-15	4.030947	4.030947	2.011119	2.011119	2.05E-14	268	206	181	218.3333	385	378	481	414.6667
SEN2171	208	92	119.6667	1.884236	1.68E-12	4.051538	4.051538	2.01847	2.01847	1.27E-11	195	137	74	135.3333	254	229	282	255
bcsE	1163	362	811.6667	1.880014	3.87E-11	3.917603	3.917603	1.969971	1.969971	2.44E-10	961	964	842	922.3333	2005	1323	1874	1734
yohL	252	90	131.6667	1.879733	3.54E-12	3.986418	3.986418	1.995093	1.995093	2.59E-11	221	131	97	149.6667	349	205	290	281.3333
SEN0331	11	1	4.666667	1.875	9.31E-05	3.832652	3.832652	1.938343	1.938343	0.000238	6	5	5	5.333333	16	6	8	10

Feature ID	Experiment - Range (original values)	Experiment - IQR (original values)	Experiment - Difference (original values)	Experiment - Fold Change (original values)	EDGE test: WT H202 vs WT NT , tagwise dispersions - P-value	EDGE test: WT H202 vs WT NT , tagwise dispersions - Fold change	WT H202 vs WT NT ABS|FC|	WT H202 vs WT NT Log2FC	WT H202 vs WT NT Log2FC +-	EDGE test: WT H202 vs WT NT , tagwise dispersions - FDR p-value correction	WT NT - WT.1.S22 - Expression values	WT NT - WT.2.S23 - Expression values	WT NT - WT.3.S24 - Expression values	WT NT - Means	WT H202 - WT.1.H2O2 - Expression values	WT H202 - WT.2.H2O2 - Expression values	WT H202 - WT.3.H2O2 - Expression values	WT H202 - Means
fepA	351	174	246	1.868235	3.74E-14	3.989341	3.989341	1.996151	1.996151	3.34E-13	334	277	239	283.3333	451	547	590	529.3333
SEN1144	12	1	4.333333	1.866667	0.000359	3.802868	3.802868	1.927088	1.927088	0.000819	6	4	5	5	16	5	7	9.333333
yqhD	1404	842	1043.333	1.862259	4.03E-10	3.955079	3.955079	1.983707	1.983707	2.27E-09	1374	1137	1119	1210	1979	2258	2523	2253.333
bcfA	97	28	52	1.861878	1.68E-10	3.941038	3.941038	1.978576	1.978576	9.79E-10	81	34	66	60.33333	94	112	131	112.3333
yhgI	1983	275	1143	1.860045	1.19E-08	3.810822	3.810822	1.930102	1.930102	5.61E-08	1132	1445	1410	1329	3115	1685	2616	2472
yiaG	118	79	65.33333	1.859649	4.89E-10	4.013286	4.013286	2.004784	2.004784	2.72E-09	129	50	49	76	126	131	167	141.3333
SEN1714	7	3	2	1.857143	0.012265	3.731725	3.731725	1.899843	1.899843	0.020711	6	0	1	2.333333	7	4	2	4.333333
hpaI	87	4	44.33333	1.852564	4.41E-10	3.973987	3.973987	1.990587	1.990587	2.47E-09	60	62	34	52	64	104	121	96.33333
SEN0722	27	20	21	1.851351	1.44E-11	3.906477	3.906477	1.965868	1.965868	9.65E-11	28	23	23	24.66667	44	50	43	45.66667
SEN1472	1478	540	934.6667	1.850728	6.01E-10	3.939667	3.939667	1.978074	1.978074	3.31E-09	1220	1160	916	1098.667	1700	2006	2394	2033.333
SEN1228	746	419	570	1.846535	6.99E-13	3.87828	3.87828	1.955417	1.955417	5.59E-12	722	668	630	673.3333	1267	1087	1376	1243.333
rna-AM93	534	90	259.3333	1.844734	1.06E-12	3.880745	3.880745	1.956334	1.956334	8.32E-12	364	340	217	307	751	430	518	566.3333
SEN1365	42	24	29.33333	1.838095	2.37E-12	3.833893	3.833893	1.93881	1.93881	1.77E-11	28	47	30	35	69	54	70	64.33333
SEN0801	51	27	34.33333	1.837398	7.11E-12	3.877264	3.877264	1.955039	1.955039	5.04E-11	51	27	45	41	72	78	76	75.33333
ybaY	1574	774	944.3333	1.835447	3.26E-09	3.957469	3.957469	1.984578	1.984578	1.62E-08	1562	1043	786	1130.333	1817	2047	2360	2074.667
cobT	485	261	368.3333	1.831452	4.66E-13	3.832742	3.832742	1.938377	-1.93838	3.82E-12	476	389	464	443	874	725	835	811.3333
SEN3455	208	65	132.3333	1.830544	4.81E-15	3.848251	3.848251	1.944203	1.944203	4.68E-14	173	176	129	159.3333	300	238	337	291.6667
bioD	17	7	11.33333	1.829268	1.59E-08	3.841247	3.841247	1.941575	1.941575	7.35E-08	15	15	11	13.66667	25	28	22	25
ssaH	12	3	4.666667	1.823529	0.000151	3.721036	3.721036	1.895704	1.895704	0.000373	5	4	8	5.666667	16	7	8	10.33333
SEN3447A	187	31	79	1.822917	2.31E-11	3.815893	3.815893	1.932021	1.932021	1.51E-10	117	98	73	96	260	129	136	175
pduW	86	33	55	1.820896	3.12E-15	3.80997	3.80997	1.92978	1.92978	3.12E-14	80	61	60	67	146	94	126	122
udhA	5887	514	2682.333	1.820453	2.31E-07	3.724771	3.724771	1.897152	1.897152	8.99E-07	2956	3683	3169	3269.333	8843	3672	5340	5951.667
ybjP	960	318	685.3333	1.816521	1.69E-11	3.784248	3.784248	1.920007	1.920007	1.12E-10	878	847	793	839.3333	1656	1165	1753	1524.667
SEN4243A	53	42	36.66667	1.814815	7.88E-12	3.878247	3.878247	1.955405	1.955405	5.54E-11	68	34	33	45	86	83	76	81.66667
nirB	1855	68	375.6667	1.809045	0.003581	4.142904	4.142904	2.050642	-2.05064	0.006758	331	667	395	464.3333	169	2024	327	840
SEN0335	13	4	7	1.807692	3.65E-06	3.73752	3.73752	1.902081	1.902081	1.16E-05	9	7	10	8.666667	20	13	14	15.66667
hpaH	95	17	54.33333	1.806931	5.92E-10	3.875198	3.875198	1.95427	1.95427	3.27E-09	65	79	58	67.33333	82	153	130	121.6667
yjgN	301	155	182	1.806499	1.02E-13	3.833446	3.833446	1.938642	1.938642	8.76E-13	310	191	176	225.6667	477	346	400	407.6667
gloA	2113	334	1218.333	1.806487	8.03E-08	3.826175	3.826175	1.935903	1.935903	3.29E-07	1477	1588	1467	1510.667	1811	2796	3580	2729
trxB	2936	1555	2011	1.805797	2.36E-08	3.820959	3.820959	1.933935	1.933935	1.06E-07	2231	2643	2613	2495.667	4185	5167	4168	4506.667
yhjB	69	28	30.33333	1.80531	4.06E-09	3.79286	3.79286	1.923286	1.923286	2E-08	58	30	25	37.66667	94	44	66	68
SEN1981	104	71	82.66667	1.805195	1.88E-15	3.806991	3.806991	1.928651	1.928651	1.93E-14	110	96	102	102.6667	173	183	200	185.3333
spaR	15	2	6.666667	1.8	8.73E-06	3.716926	3.716926	1.89411	1.89411	2.63E-05	10	8	7	8.333333	22	10	13	15

Feature ID	Experiment - Range (original values)	Experiment - IQR (original values)	Experiment - Difference (original values)	Experiment - Fold Change (original values)	EDGE test: WT H202 vs WT NT , tagwise dispersions - P-value	EDGE test: WT H202 vs WT NT , tagwise dispersions - Fold change	WT H202 vs WT NT ABS|FC|	WT H202 vs WT NT Log2FC	WT H202 vs WT NT Log2FC +-	EDGE test: WT H202 vs WT NT , tagwise dispersions - FDR p-value correction	WT NT - WT.1.S22 - Expression values	WT NT - WT.2.S23 - Expression values	WT NT - WT.3.S24 - Expression values	WT NT - Means	WT H202 - WT.1.H2O2 - Expression values	WT H202 - WT.2.H2O2 - Expression values	WT H202 - WT.3.H2O2 - Expression values	WT H202 - Means
sscA	18	11	13.66667	1.788462	9.15E-10	3.757677	3.757677	1.909841	1.909841	4.9E-09	20	18	14	17.33333	32	32	29	31
SEN2130	125	62	88.66667	1.786982	2.64E-14	3.774829	3.774829	1.916411	1.916411	2.36E-13	112	122	104	112.6667	174	201	229	201.3333
gmd	85	13	44	1.785714	7.1E-11	3.789334	3.789334	1.921944	1.921944	4.34E-10	59	65	44	56	72	99	129	100
yjfI	8	4	4.666667	1.777778	3.75E-05	3.67469	3.67469	1.877623	1.877623	0.000102	9	5	4	6	12	9	11	10.66667
SEN0999	12	2	4.666667	1.777778	0.00013	3.633599	3.633599	1.861399	1.861399	0.000323	2	7	9	6	14	9	9	10.66667
wzb	5	3	2.333333	1.777778	0.010312	3.571305	3.571305	1.836451	1.836451	0.017662	2	2	5	3	6	3	7	5.333333
manZ	29946	5559	14363	1.766967	1.07E-05	3.698559	3.698559	1.886963	1.886963	3.16E-05	11464	19929	24788	18727	25488	41410	32372	33090
rmf	2436	1231	936.6667	1.761311	1.12E-05	3.762012	3.762012	1.911505	1.911505	3.29E-05	2249	696	746	1230.333	3132	1392	1977	2167
orf242	642	180	338.6667	1.754829	1.88E-11	3.684965	3.684965	1.881651	1.881651	1.25E-10	578	401	367	448.6667	1009	581	772	787.3333
cbiM	1753	1106	813	1.750924	0.000628	3.527352	3.527352	1.818586	1.818586	0.001377	851	322	2075	1082.667	1958	1772	1957	1895.667
SEN2380A	6	3	4	1.75	0.000163	3.604985	3.604985	1.849993	1.849993	0.000398	7	5	4	5.333333	10	8	10	9.333333
yjfJ	19	7	12	1.75	2.89E-09	3.649578	3.649578	1.86773	1.86773	1.45E-08	19	15	14	16	33	22	29	28
deaD	5495	1338	2977	1.746739	1.1E-08	3.668344	3.668344	1.875129	1.875129	5.2E-08	4781	3974	3205	3986.667	8700	5312	6879	6963.667
SEN4087	21	11	8.666667	1.742857	1.32E-06	3.641245	3.641245	1.864432	1.864432	4.53E-06	20	8	7	11.66667	28	14	19	20.33333
ycfJ	702	335	483.6667	1.733199	2.24E-11	3.706102	3.706102	1.889902	1.889902	1.47E-10	732	752	495	659.6667	1166	1197	1067	1143.333
pduS	33	9	18	1.72973	3.75E-10	3.593702	3.593702	1.845471	1.845471	2.12E-09	25	22	27	24.66667	55	34	39	42.66667
livG	92	46	57	1.72766	2.98E-12	3.605995	3.605995	1.850398	1.850398	2.21E-11	77	66	92	78.33333	123	125	158	135.3333
hflB	15337	5787	10479.67	1.72651	3.38E-08	3.586141	3.586141	1.842432	1.842432	1.48E-07	13721	15060	14493	14424.67	29058	20280	25375	24904.33
invH	48	13	25.66667	1.726415	1.26E-11	3.607832	3.607832	1.851132	1.851132	8.56E-11	35	44	27	35.33333	75	48	60	61
ybfA	1606	734	693	1.722377	8.62E-07	3.763309	3.763309	1.912002	1.912002	3.05E-06	1610	789	479	959.3333	1349	1523	2085	1652.333
yeeA	2455	313	677.3333	1.722104	3.91E-05	3.499641	3.499641	1.807207	1.807207	0.000106	1101	925	788	938	3042	587	1217	1615.333
proP	615	172	341	1.714885	2.22E-09	3.726289	3.726289	1.897739	1.897739	1.12E-08	616	444	371	477	585	986	883	818
yiaN	15	9	10.66667	1.711111	2.86E-08	3.583294	3.583294	1.841286	1.841286	1.26E-07	15	18	12	15	27	26	24	25.66667
yqhE	1013	465	686.3333	1.706346	9.69E-10	3.625918	3.625918	1.858346	1.858346	5.17E-09	1081	962	872	971.6667	1427	1662	1885	1658
SEN0914	20	2	4	1.705882	0.011292	3.541475	3.541475	1.82435	1.82435	0.019237	14	0	3	5.666667	20	5	4	9.666667
sgbU	22	11	11.66667	1.7	8.62E-08	3.606904	3.606904	1.850761	1.850761	3.52E-07	24	12	14	16.66667	25	34	26	28.33333
msgA	170	28	87	1.694149	6.03E-13	3.532217	3.532217	1.820574	1.820574	4.87E-12	146	122	108	125.3333	278	150	209	212.3333
SEN4244	127	82	92.66667	1.693267	7.48E-15	3.569847	3.569847	1.835862	1.835862	7.13E-14	153	124	124	133.6667	222	206	251	226.3333
SEN1005	19	5	9	1.692308	7.56E-07	3.503202	3.503202	1.808674	1.808674	2.72E-06	8	18	13	13	22	17	27	22
SEN1997	7	1	3	1.692308	0.001947	3.456188	3.456188	1.789182	1.789182	0.003866	3	5	5	4.333333	6	6	10	7.333333
SEN0710	21	6	10.33333	1.688889	7.54E-08	3.523309	3.523309	1.816931	1.816931	3.11E-07	20	10	15	15	31	21	24	25.33333
SEN0784	11	7	7.333333	1.6875	1.03E-06	3.534826	3.534826	1.821639	1.821639	3.58E-06	15	8	9	10.66667	16	19	19	18
ygaC	898	286	491.3333	1.687179	2.83E-10	3.534476	3.534476	1.821496	1.821496	1.62E-09	917	597	631	715	1495	883	1241	1206.333

Feature ID	Experiment - Range (original values)	Experiment - IQR (original values)	Experiment - Difference (original values)	Experiment - Fold Change (original values)	EDGE test: WT H202 vs WT NT , tagwise dispersions - P-value	EDGE test: WT H202 vs WT NT , tagwise dispersions - Fold change	WT H202 vs WT NT ABS|FC|	WT H202 vs WT NT Log2FC	WT H202 vs WT NT Log2FC +-	EDGE test: WT H202 vs WT NT , tagwise dispersions - FDR p-value correction	WT NT - WT.1.S22 - Expression values	WT NT - WT.2.S23 - Expression values	WT NT - WT.3.S24 - Expression values	WT NT - Means	WT H202 - WT.1.H2O2 - Expression values	WT H202 - WT.2.H2O2 - Expression values	WT H202 - WT.3.H2O2 - Expression values	WT H202 - Means
SEN2868	108	46	70.66667	1.686084	6.62E-14	3.550392	3.550392	1.827978	1.827978	5.82E-13	134	90	85	103	193	136	192	173.6667
SEN0163	312	148	207	1.684675	1.47E-11	3.610089	3.610089	1.852034	1.852034	9.85E-11	347	280	280	302.3333	428	592	508	509.3333
rbsD	314	44	77.33333	1.684366	0.000168	3.375384	3.375384	1.755052	-1.75505	0.000411	103	140	96	113	362	48	161	190.3333
SEN3492	26	2	13	1.672414	4.52E-08	3.4561	3.4561	1.789145	1.789145	1.93E-07	20	16	22	19.33333	42	21	34	32.33333
yajB	321	126	168.6667	1.668428	1.56E-12	3.52953	3.52953	1.819476	1.819476	1.19E-11	322	235	200	252.3333	521	361	381	421
SEN3428	20	14	10.66667	1.666667	6.1E-08	3.512894	3.512894	1.81266	1.81266	2.56E-07	26	11	11	16	31	25	24	26.66667
smpA	2830	989	1761	1.66612	1.41E-08	3.490437	3.490437	1.803408	1.803408	6.58E-08	2930	2617	2384	2643.667	5214	3606	4394	4404.667
SEN3370	407	55	96.33333	1.664368	0.000376	3.324761	3.324761	1.733251	1.733251	0.000855	98	213	124	145	489	82	153	241.3333
yeaG	1325	651	517.6667	1.663108	1.44E-06	3.581123	3.581123	1.840412	1.840412	4.92E-06	1353	538	451	780.6667	1189	930	1776	1298.333
fur	3095	484	1907.667	1.661542	4.36E-08	3.439611	3.439611	1.782245	-1.78225	1.87E-07	2976	2888	2787	2883.667	5882	3372	5120	4791.333
SEN2006	84	49	42.33333	1.661458	1.61E-10	3.524783	3.524783	1.817534	1.817534	9.44E-10	97	47	48	64	104	84	131	106.3333
lpp	228266	55223	130732.7	1.659149	6.62E-08	3.472344	3.472344	1.79591	1.79591	2.76E-07	215778	206734	172495	198335.7	400761	261957	324487	329068.3
lpfB	22	6	11.33333	1.653846	5.87E-07	3.41876	3.41876	1.773473	1.773473	2.15E-06	21	15	16	17.33333	37	15	34	28.66667
yaiC	597	178	321	1.651556	7.08E-08	3.593104	3.593104	1.845231	1.845231	2.93E-07	637	459	382	492.6667	494	979	968	813.6667
yjeH	574	80	190.6667	1.649262	4.49E-07	3.365766	3.365766	1.750935	1.750935	1.67E-06	297	332	252	293.6667	804	230	419	484.3333
SEN3819	193	76	119.6667	1.649186	1.43E-13	3.462354	3.462354	1.791753	1.791753	1.22E-12	210	179	164	184.3333	357	255	300	304
proC	3700	970	1929	1.646954	2.63E-06	3.59038	3.59038	1.844137	1.844137	8.54E-06	3825	2870	2250	2981.667	2855	5950	5927	4910.667
allB	174	73	84.33333	1.645408	1.23E-11	3.495672	3.495672	1.80557	-1.80557	8.39E-11	185	112	95	130.6667	269	183	193	215
psiF	112	78	47.66667	1.644144	3.69E-06	3.613327	3.613327	1.853328	-1.85333	1.18E-05	146	42	34	74	113	120	132	121.6667
SEN2134	36	9	21.66667	1.643564	1.29E-10	3.418204	3.418204	1.773239	1.773239	7.63E-10	34	30	37	33.66667	57	43	66	55.33333
SEN0083	72	53	49	1.63913	4.73E-12	3.504639	3.504639	1.809266	1.809266	3.42E-11	101	65	64	76.66667	118	136	123	125.6667
SEN1158	26	8	10	1.638298	6.09E-06	3.382813	3.382813	1.758223	1.758223	1.88E-05	21	13	13	15.66667	38	12	27	25.66667
metE	505	128	299.3333	1.637784	3.49E-10	3.386758	3.386758	1.759905	1.759905	1.98E-09	402	554	452	469.3333	907	580	819	768.6667
SEN4156	15	10	11.66667	1.636364	7.83E-09	3.418078	3.418078	1.773185	1.773185	3.75E-08	17	19	19	18.33333	32	29	29	30
csgF	6	4	2.333333	1.636364	0.00703	3.360078	3.360078	1.748495	-1.74849	0.01248	7	1	3	3.666667	4	7	7	6
SEN0655	69	42	55.33333	1.633588	1.85E-12	3.441396	3.441396	1.782994	1.782994	1.4E-11	82	89	91	87.33333	131	151	146	142.6667
invE	35	18	19	1.633333	2.49E-10	3.440353	3.440353	1.782556	1.782556	1.44E-09	40	26	24	30	59	44	44	49
ugpQ	334	92	222	1.631879	1.15E-12	3.418721	3.418721	1.773457	1.773457	8.95E-12	385	362	307	351.3333	625	454	641	573.3333
SEN1165	12	1	8	1.631579	5.8E-07	3.384242	3.384242	1.758833	1.758833	2.13E-06	13	13	12	12.66667	24	14	24	20.66667
ybiV(1)	407	131	235.3333	1.628114	4.86E-11	3.391601	3.391601	1.761966	1.761966	3.02E-10	346	403	375	374.6667	571	506	753	610
pduE	28	8	10.66667	1.627451	8.37E-06	3.426192	3.426192	1.776606	-1.77661	2.53E-05	24	9	18	17	37	30	16	27.66667
SEN4022	60	34	38.66667	1.627027	4.73E-11	3.453195	3.453195	1.787932	1.787932	2.95E-10	58	72	55	61.66667	92	115	94	100.3333
ydcI	898	91	388.6667	1.626545	3.73E-09	3.388007	3.388007	1.760437	1.760437	1.85E-08	653	744	464	620.3333	1362	684	981	1009

Feature ID	Experiment - Range (original values)	Experiment - IQR (original values)	Experiment - Difference (original values)	Experiment - Fold Change (original values)	EDGE test: WT H202 vs WT NT , tagwise dispersions - P-value	EDGE test: WT H202 vs WT NT , tagwise dispersions - Fold change	WT H202 vs WT NT ABS|FC|	WT H202 vs WT NT Log2FC	WT H202 vs WT NT Log2FC +-	EDGE test: WT H202 vs WT NT , tagwise dispersions - FDR p-value correction	WT NT - WT.1.S22 - Expression values	WT NT - WT.2.S23 - Expression values	WT NT - WT.3.S24 - Expression values	WT NT - Means	WT H202 - WT.1.H2O2 - Expression values	WT H202 - WT.2.H2O2 - Expression values	WT H202 - WT.3.H2O2 - Expression values	WT H202 - Means
ygaU	89	37	45.66667	1.622727	1.17E-10	3.471273	3.471273	1.795465	1.795465	6.93E-10	101	64	55	73.33333	96	117	144	119
sinH	32	1	15.33333	1.621622	1.49E-09	3.399459	3.399459	1.765305	1.765305	7.7E-09	29	30	15	24.66667	47	30	43	40
erfK	426	164	260	1.620032	8.92E-12	3.411063	3.411063	1.770221	-1.77022	6.21E-11	493	398	367	419.3333	683	562	793	679.3333
wza	14	6	8	1.615385	9.16E-07	3.370393	3.370393	1.752917	1.752917	3.23E-06	11	17	11	13	25	21	17	21
SEN0755	20	10	11.66667	1.614035	2.82E-08	3.394939	3.394939	1.763386	1.763386	1.25E-07	26	13	18	19	33	31	28	30.66667
yaiY	558	184	260.3333	1.613511	2.5E-10	3.419289	3.419289	1.773696	1.773696	1.45E-09	568	385	320	424.3333	878	569	607	684.6667
SEN0336	154	68	87.33333	1.609302	8.07E-13	3.392417	3.392417	1.762313	1.762313	6.4E-12	169	131	130	143.3333	209	199	284	230.6667
SEN0317	19	12	10.33333	1.607843	4.85E-08	3.377726	3.377726	1.756052	1.756052	2.07E-07	26	14	11	17	30	22	30	27.33333
nrdE	226	113	142.6667	1.607092	7.87E-13	3.419152	3.419152	1.773639	-1.77364	6.25E-12	292	234	179	235	381	347	405	377.6667
ssaI	6	2	3	1.6	0.001405	3.267899	3.267899	1.708363	1.708363	0.002872	5	4	6	5	10	4	10	8
iacP	15	1	5	1.6	0.000275	3.287437	3.287437	1.716963	1.716963	0.000645	8	8	9	8.333333	11	7	22	13.33333
kgtP	301	24	169	1.599291	1.56E-09	3.424731	3.424731	1.775991	1.775991	8.03E-09	294	318	234	282	316	502	535	451
ybaL	561	269	381	1.596244	1.21E-10	3.338018	3.338018	1.738992	1.738992	7.17E-10	610	690	617	639	1003	886	1171	1020
citA	317	70	180	1.596026	6.25E-12	3.317816	3.317816	1.730234	-1.73023	4.45E-11	303	317	286	302	603	373	470	482
SEN2340	93	56	64.33333	1.593846	6.41E-14	3.360783	3.360783	1.748798	1.748798	5.65E-13	126	100	99	108.3333	170	156	192	172.6667
SEN0419	50	7	23.33333	1.588235	8.03E-10	3.320691	3.320691	1.731484	1.731484	4.38E-09	46	39	34	39.66667	60	45	84	63
fimY	51	29	28	1.587413	4.77E-11	3.370459	3.370459	1.752945	1.752945	2.98E-10	67	41	35	47.66667	86	71	70	75.66667
ybaD	772	246	428	1.586837	1.05E-09	3.321339	3.321339	1.731765	1.731765	5.57E-09	912	666	610	729.3333	1382	803	1287	1157.333
SEN2681	168	67	91	1.585837	2.44E-12	3.380018	3.380018	1.757031	1.757031	1.82E-11	206	154	106	155.3333	274	221	244	246.3333
SEN3797	107	23	36	1.583784	3.71E-07	3.261101	3.261101	1.705359	1.705359	1.4E-06	76	56	53	61.66667	155	48	90	97.66667
wcaH	25	3	9.333333	1.583333	7.94E-07	3.323668	3.323668	1.732776	1.732776	2.84E-06	18	21	9	16	21	21	34	25.33333
SEN3578	8	2	2.333333	1.583333	0.005434	3.221357	3.221357	1.687668	1.687668	0.009885	4	3	5	4	11	5	3	6.333333
sbp	107	54	71.33333	1.581522	7.41E-14	3.34075	3.34075	1.740172	1.740172	6.48E-13	143	122	103	122.6667	210	176	196	194
yafH	731	147	311	1.577351	4.75E-08	3.303793	3.303793	1.724123	1.724123	2.03E-07	652	613	351	538.6667	962	505	1082	849.6667
SEN2225	13	8	5	1.576923	0.00018	3.265709	3.265709	1.707396	1.707396	0.000439	14	7	5	8.666667	18	6	17	13.66667
SEN2923	18	5	8	1.571429	8.45E-07	3.277888	3.277888	1.712766	1.712766	3E-06	18	10	14	14	28	19	19	22
SEN4032	54	7	26.33333	1.568345	8.82E-10	3.239638	3.239638	1.695833	1.695833	4.74E-09	41	47	51	46.33333	95	54	69	72.66667
SEN1193	1383	335	782.6667	1.566329	3.67E-08	3.273218	3.273218	1.71071	1.71071	1.59E-07	1625	1290	1231	1382	2614	1603	2277	2164.667
invC	32	13	19.33333	1.563107	4.33E-10	3.262393	3.262393	1.705931	1.705931	2.44E-09	32	36	35	34.33333	64	48	49	53.66667
SEN3166	468	71	223.3333	1.562552	5.35E-10	3.243537	3.243537	1.697568	1.697568	2.97E-09	418	395	378	397	846	466	549	620.3333
SEN1996	4	3	3	1.5625	0.001108	3.218093	3.218093	1.686206	1.686206	0.002311	5	6	5	5.333333	9	8	8	8.333333
hpaD	273	124	117	1.5616	7.34E-07	3.432616	3.432616	1.779309	1.779309	2.65E-06	311	187	127	208.3333	188	388	400	325.3333
yidZ	396	111	243	1.556064	2.46E-11	3.268241	3.268241	1.708514	1.708514	1.6E-10	492	445	374	437	770	556	714	680

Feature ID	Experiment - Range (original values)	Experiment - IQR (original values)	Experiment - Difference (original values)	Experiment - Fold Change (original values)	EDGE test: WT H202 vs WT NT , tagwise dispersions - P-value	EDGE test: WT H202 vs WT NT , tagwise dispersions - Fold change	WT H202 vs WT NT ABS|FC|	WT H202 vs WT NT Log2FC	WT H202 vs WT NT Log2FC +-	EDGE test: WT H202 vs WT NT , tagwise dispersions - FDR p-value correction	WT NT - WT.1.S22 - Expression values	WT NT - WT.2.S23 - Expression values	WT NT - WT.3.S24 - Expression values	WT NT - Means	WT H202 - WT.1.H2O2 - Expression values	WT H202 - WT.2.H2O2 - Expression values	WT H202 - WT.3.H2O2 - Expression values	WT H202 - Means
SEN1502	3	1	1.666667	1.555556	0.010223	3.147375	3.147375	1.654149	1.654149	0.017523	2	4	3	3	5	4	5	4.666667
SEN4243	466	289	329	1.554806	1.6E-10	3.292583	3.292583	1.71922	1.71922	9.37E-10	739	531	509	593	975	820	971	922
SEN4088	220	61	109.6667	1.553872	2.24E-11	3.279023	3.279023	1.713266	1.713266	1.47E-10	222	204	168	198	270	265	388	307.6667
SEN3801	23	7	8.666667	1.553191	2.31E-06	3.224651	3.224651	1.689143	1.689143	7.58E-06	21	12	14	15.66667	35	15	23	24.33333
hepA	678	308	459	1.551241	1.74E-09	3.240934	3.240934	1.69641	1.69641	8.96E-09	780	870	848	832.6667	1261	1156	1458	1291.667
SEN1523	25	6	11.33333	1.548387	2.83E-08	3.253513	3.253513	1.701998	1.701998	1.25E-07	28	22	12	20.66667	37	25	34	32
SEN3343	355	155	219	1.542527	2.86E-11	3.260174	3.260174	1.704949	1.704949	1.82E-10	443	417	351	403.6667	590	572	706	622.6667
phnR	184	72	92.33333	1.539961	2.29E-11	3.279399	3.279399	1.713431	-1.71343	1.49E-10	225	161	127	171	246	233	311	263.3333
SEN2355	43	15	11.33333	1.539683	0.000199	3.139924	3.139924	1.65073	1.65073	0.000482	28	12	23	21	55	13	29	32.33333
yjbH	223	121	144.6667	1.53913	3.31E-12	3.267641	3.267641	1.70825	1.70825	2.43E-11	329	260	216	268.3333	439	381	419	413
SEN3800	74	14	37.33333	1.538462	1.83E-09	3.181338	3.181338	1.669634	1.669634	9.35E-09	71	77	60	69.33333	134	63	123	106.6667
sdhB	3742	1263	1378	1.533833	1.98E-05	3.155268	3.155268	1.657763	1.657763	5.62E-05	2325	3341	2078	2581.333	4030	2053	5795	3959.333
copA	4454	223	2216.667	1.529754	1.69E-06	3.24123	3.24123	1.696541	1.696541	5.71E-06	4241	4395	3917	4184.333	4464	6368	8371	6401
orf32	176	38	85	1.525773	9.9E-12	3.198921	3.198921	1.677585	1.677585	6.83E-11	195	157	133	161.6667	309	186	245	246.6667
ribD	913	444	544.3333	1.515793	1.03E-08	3.206175	3.206175	1.680853	1.680853	4.86E-08	1378	934	854	1055.333	1767	1299	1733	1599.667
SEN2170	165	61	80.33333	1.514957	2.65E-11	3.230333	3.230333	1.691683	1.691683	1.71E-10	204	152	112	156	219	213	277	236.3333
potF	88	33	48.66667	1.514085	1.47E-11	3.180045	3.180045	1.669047	-1.66905	9.87E-11	104	85	95	94.66667	129	128	173	143.3333
priC	143	55	84.66667	1.512097	1.44E-12	3.183505	3.183505	1.670616	-1.67062	1.11E-11	202	152	142	165.3333	285	207	258	250
SEN0326	148	52	74	1.509174	3.83E-11	3.174174	3.174174	1.666381	1.666381	2.41E-10	189	137	110	145.3333	258	157	243	219.3333
SEN0084	68	56	37	1.506849	8.2E-10	3.21687	3.21687	1.685658	1.685658	4.45E-09	112	56	51	73	114	97	119	110
ssaL	42	25	21	1.504	1.81E-09	3.177152	3.177152	1.667734	1.667734	9.24E-09	60	30	35	41.66667	63	53	72	62.66667
uspA	24156	12973	15849.33	1.503175	3.6E-07	3.180784	3.180784	1.669383	1.669383	1.36E-06	38147	29028	27321	31498.67	48566	42001	51477	47348
yeaN	156	44	78.33333	1.501066	2.87E-11	3.15462	3.15462	1.657466	1.657466	1.83E-10	196	152	121	156.3333	277	172	255	234.6667
SEN3799	13	5	7.333333	1.5	9.79E-07	3.157028	3.157028	1.658567	1.658567	3.43E-06	19	15	10	14.66667	20	23	23	22
rna-AM93	192	32	73	1.494357	4.33E-10	3.156406	3.156406	1.658283	1.658283	2.44E-09	188	156	99	147.6667	291	172	199	220.6667
SEN4089	283	100	148	1.492239	9.19E-11	3.178982	3.178982	1.668565	1.668565	5.55E-10	366	294	242	300.6667	394	427	525	448.6667
gtrC	22	5	10	1.491803	3.7E-07	3.113423	3.113423	1.638502	1.638502	1.4E-06	19	24	18	20.33333	40	27	24	30.33333
fadD	1268	28	236.6667	1.489993	0.000972	2.982091	2.982091	1.576324	-1.57632	0.002055	486	505	458	483	1475	207	477	719.6667
livF	66	24	39.33333	1.489627	2.13E-11	3.150214	3.150214	1.65545	-1.65545	1.4E-10	84	89	68	80.33333	108	117	134	119.6667
rspB	13	4	7.333333	1.488889	1.32E-06	3.099458	3.099458	1.632016	1.632016	4.54E-06	14	18	13	15	24	17	26	22.33333
SEN0332	40	15	21.66667	1.488722	8.85E-10	3.136092	3.136092	1.648968	1.648968	4.75E-09	52	38	43	44.33333	58	62	78	66
SEN1425	492	99	241	1.488514	2.1E-08	3.157979	3.157979	1.659002	1.659002	9.51E-08	479	578	423	493.3333	552	736	915	734.3333
dinG	1597	216	937.3333	1.485749	1.86E-07	3.109512	3.109512	1.636688	1.636688	7.34E-07	2051	2072	1666	1929.667	3263	2267	3071	2867

Feature ID	Experiment - Range (original values)	Experiment - IQR (original values)	Experiment - Difference (original values)	Experiment - Fold Change (original values)	EDGE test: WT H202 vs WT NT , tagwise dispersions - P-value	EDGE test: WT H202 vs WT NT , tagwise dispersions - Fold change	WT H202 vs WT NT ABS|FC|	WT H202 vs WT NT Log2FC	WT H202 vs WT NT Log2FC +-	EDGE test: WT H202 vs WT NT , tagwise dispersions - FDR p-value correction	WT NT - WT.1.S22 - Expression values	WT NT - WT.2.S23 - Expression values	WT NT - WT.3.S24 - Expression values	WT NT - Means	WT H202 - WT.1.H2O2 - Expression values	WT H202 - WT.2.H2O2 - Expression values	WT H202 - WT.3.H2O2 - Expression values	WT H202 - Means
yqhA	539	37	197.6667	1.478612	8.39E-09	3.084967	3.084967	1.625255	1.625255	4.01E-08	466	450	323	413	862	429	541	610.6667
eutA	177	96	52.33333	1.475758	5.94E-05	3.237507	3.237507	1.694883	-1.69488	0.000157	223	46	61	110	177	157	153	162.3333
cpsG	19	10	9.666667	1.47541	2.84E-07	3.130053	3.130053	1.646187	-1.64619	1.09E-06	28	18	15	20.33333	27	34	29	30
SEN0994	1020	116	203.3333	1.46851	0.000367	3.003749	3.003749	1.586764	1.586764	0.000836	602	360	340	434	1238	218	456	637.3333
SEN3009	31	14	12.33333	1.468354	7.85E-07	3.054769	3.054769	1.611063	1.611063	2.81E-06	36	21	22	26.33333	52	22	42	38.66667
SEN3843	46	9	22.33333	1.465278	2.6E-10	3.080358	3.080358	1.623098	1.623098	1.5E-09	58	49	37	48	73	55	83	70.33333
SEN4242	352	180	206.6667	1.462687	4.72E-10	3.102213	3.102213	1.633298	1.633298	2.63E-09	575	395	370	446.6667	668	570	722	653.3333
SEN1524	80	46	39.66667	1.46124	8.19E-10	3.129321	3.129321	1.64585	1.64585	4.45E-09	124	74	60	86	117	120	140	125.6667
SEN2969	109	32	50	1.458716	6E-11	3.10143	3.10143	1.632934	1.632934	3.67E-10	141	109	77	109	152	139	186	159
entD	32	5	16.33333	1.457944	2.12E-08	3.082385	3.082385	1.624047	1.624047	9.6E-08	37	41	29	35.66667	42	53	61	52
SEN0620	197	113	117.6667	1.457254	7.24E-11	3.096356	3.096356	1.630572	1.630572	4.41E-10	340	227	205	257.3333	392	331	402	375
creB	369	168	271.3333	1.454495	9.69E-10	3.056387	3.056387	1.611827	-1.61183	5.17E-09	616	623	552	597	921	784	900	868.3333
yhcO	41	12	16	1.45283	3.2E-07	3.017324	3.017324	1.59327	1.59327	1.22E-06	44	26	36	35.33333	55	32	67	51.33333
SEN4280	239	95	99.66667	1.451662	1.78E-09	3.099364	3.099364	1.631972	1.631972	9.13E-09	307	202	153	220.6667	297	272	392	320.3333
SEN4138	21	6	12.33333	1.45122	2.81E-08	3.022985	3.022985	1.595974	1.595974	1.24E-07	26	32	24	27.33333	45	29	45	39.66667
amiA	497	101	294.6667	1.450331	1.12E-09	3.038067	3.038067	1.603154	-1.60315	5.91E-09	728	655	580	654.3333	1077	756	1014	949
ybhP	19	9	6	1.45	5.79E-05	3.005091	3.005091	1.587409	1.587409	0.000154	19	10	11	13.33333	29	10	19	19.33333
SEN0030	14	1	6	1.45	2.01E-05	2.999877	2.999877	1.584904	1.584904	5.7E-05	14	13	13	13.33333	25	11	22	19.33333
SEN2375A	68	16	22	1.445946	1.89E-07	3.069017	3.069017	1.617777	1.617777	7.46E-07	72	51	25	49.33333	93	54	67	71.33333
SEN2169	132	11	58	1.445013	5.97E-10	3.085239	3.085239	1.625382	1.625382	3.3E-09	152	149	90	130.3333	160	183	222	188.3333
SEN1992	12	2	2.666667	1.444444	0.003983	2.964618	2.964618	1.567846	1.567846	0.007456	5	9	4	6	16	6	4	8.666667
SEN3354	55	27	37	1.444	1.57E-11	3.032301	3.032301	1.600413	1.600413	1.05E-10	90	80	80	83.33333	119	107	135	120.3333
asnC	199	16	80.33333	1.443015	6.79E-09	2.983907	2.983907	1.577203	-1.5772	3.29E-08	193	177	174	181.3333	373	179	233	261.6667
SEN3798	25	9	9	1.442623	1.77E-06	3.019336	3.019336	1.594232	1.594232	5.96E-06	27	23	11	20.33333	36	18	34	29.33333
SEN3184	27	2	8.666667	1.440678	4.17E-06	3.052875	3.052875	1.610169	1.610169	1.31E-05	24	27	8	19.66667	24	26	35	28.33333
SEN3833	566	167	302.6667	1.43738	2.05E-09	3.030176	3.030176	1.599402	1.599402	1.04E-08	763	703	610	692	938	870	1176	994.6667
SEN3649	32	12	17	1.432203	2.22E-09	2.999331	2.999331	1.584641	1.584641	1.12E-08	48	36	34	39.33333	61	42	66	56.33333
sscB	11	3	6.333333	1.431818	2.11E-06	2.987284	2.987284	1.578834	1.578834	6.97E-06	15	16	13	14.66667	24	18	21	21
rna-AM93	146	25	42.66667	1.42953	1.27E-07	2.996817	2.996817	1.583431	1.583431	5.08E-07	130	101	67	99.33333	213	94	119	142
SEN0920	114	26	70.66667	1.42915	1.38E-11	2.991907	2.991907	1.581065	1.581065	9.3E-11	170	171	153	164.6667	243	196	267	235.3333
ybiB	1092	167	448	1.428708	1.86E-06	3.033146	3.033146	1.600815	1.600815	6.21E-06	1062	1142	931	1045	975	1481	2023	1493
sgbH	13	5	5	1.428571	5.32E-05	2.987542	2.987542	1.578959	1.578959	0.000142	11	18	6	11.66667	16	15	19	16.66667
yhgH	160	20	79	1.426259	8.08E-10	2.958752	2.958752	1.564989	1.564989	4.4E-09	188	202	166	185.3333	326	182	285	264.3333

Feature ID	Experiment - Range (original values)	Experiment - IQR (original values)	Experiment - Difference (original values)	Experiment - Fold Change (original values)	EDGE test: WT H202 vs WT NT , tagwise dispersions - P-value	EDGE test: WT H202 vs WT NT , tagwise dispersions - Fold change	WT H202 vs WT NT ABS|FC|	WT H202 vs WT NT Log2FC	WT H202 vs WT NT Log2FC +-	EDGE test: WT H202 vs WT NT , tagwise dispersions - FDR p-value correction	WT NT - WT.1.S22 - Expression values	WT NT - WT.2.S23 - Expression values	WT NT - WT.3.S24 - Expression values	WT NT - Means	WT H202 - WT.1.H2O2 - Expression values	WT H202 - WT.2.H2O2 - Expression values	WT H202 - WT.3.H2O2 - Expression values	WT H202 - Means
ybaJ	1317	402	551	1.425045	4.33E-07	3.013488	3.013488	1.591434	1.591434	1.62E-06	1680	1278	931	1296.333	2248	1425	1869	1847.333
sipA	117	26	56.66667	1.42394	2.12E-11	3.01107	3.01107	1.590276	1.590276	1.39E-10	156	143	102	133.6667	219	169	183	190.3333
yhgG	332	102	130	1.418904	2.51E-08	2.940939	2.940939	1.556277	1.556277	1.12E-07	299	388	244	310.3333	576	286	459	440.3333
yfaW	813	205	433	1.417282	1.91E-08	2.99561	2.99561	1.58285	1.58285	8.77E-08	1208	1057	848	1037.667	1489	1262	1661	1470.667
SEN0270	15	2	6.666667	1.416667	2.35E-06	2.967468	2.967468	1.569232	1.569232	7.7E-06	18	20	10	16	24	19	25	22.66667
SEN1726	963	99	213	1.415745	0.000106	2.842581	2.842581	1.507202	1.507202	0.000268	466	506	565	512.3333	1272	309	595	725.3333
fdx	568	146	254.6667	1.412082	2.04E-06	2.992544	2.992544	1.581372	1.581372	6.79E-06	543	697	614	618	551	956	1111	872.6667
SEN3985	37	4	16.66667	1.406504	9.74E-08	2.958242	2.958242	1.56474	1.56474	3.95E-07	50	26	47	41	63	59	51	57.66667
cbiL	1443	649	355	1.406024	0.004094	2.811167	2.811167	1.491169	1.491169	0.007646	668	256	1699	874.3333	1337	1034	1317	1229.333
ylbF	19	2	6.666667	1.4	7.04E-05	2.965325	2.965325	1.56819	1.56819	0.000183	17	14	19	16.66667	19	33	18	23.33333
sseF	15	2	5.666667	1.395349	1.72E-05	2.897433	2.897433	1.534775	1.534775	4.93E-05	16	13	14	14.33333	28	14	18	20
ilvH	142	51	84.33333	1.392248	8.79E-11	2.928039	2.928039	1.549935	1.549935	5.32E-10	254	203	188	215	330	247	321	299.3333
pduC	191	46	38.33333	1.388514	0.000274	2.850475	2.850475	1.511202	-1.5112	0.000643	124	55	117	98.66667	246	94	71	137
SEN4351	38	15	15.33333	1.386555	5.03E-08	2.911028	2.911028	1.541529	1.541529	2.14E-07	53	38	28	39.66667	66	38	61	55
nagC	1839	279	660	1.384989	9.15E-06	2.847706	2.847706	1.5098	-1.5098	2.74E-05	1754	1834	1555	1714.333	3208	1369	2546	2374.333
ydfI	118	36	72.33333	1.384752	1.15E-10	2.901829	2.901829	1.536963	1.536963	6.82E-10	216	168	180	188	286	215	280	260.3333
mdlB	219	39	110.6667	1.384259	3.38E-10	2.894385	2.894385	1.533257	-1.53326	1.92E-09	310	288	266	288	485	327	384	398.6667
yciO	928	32	502.6667	1.378704	4.54E-07	2.871133	2.871133	1.52162	1.52162	1.69E-06	1397	1378	1207	1327.333	2135	1365	1990	1830
SEN4241	146	52	65.33333	1.377649	2.82E-10	2.900773	2.900773	1.536438	1.536438	1.62E-09	214	162	143	173	241	185	289	238.3333
eutK	136	77	44	1.377143	1.37E-06	2.981014	2.981014	1.575803	1.575803	4.71E-06	203	80	67	116.6667	157	142	183	160.6667
cboQ	755	254	205.3333	1.376989	0.000986	2.802033	2.802033	1.486474	1.486474	0.002082	477	201	956	544.6667	731	711	808	750
SEN1155	22	15	9.666667	1.376623	3.57E-07	2.887156	2.887156	1.529649	1.529649	1.35E-06	36	21	20	25.66667	39	25	42	35.33333
SEN0038	101	26	46.33333	1.375676	1.09E-10	2.884044	2.884044	1.528093	1.528093	6.49E-10	145	119	106	123.3333	207	133	169	169.6667
SEN1802	2283	672	831	1.375113	7.71E-06	2.908116	2.908116	1.540085	1.540085	2.35E-05	3025	1686	1935	2215.333	3969	2607	2563	3046.333
SEN3475	193	76	68	1.374312	1.17E-08	2.882828	2.882828	1.527485	1.527485	5.48E-08	241	159	145	181.6667	338	176	235	249.6667
mazG	474	84	222	1.373318	4.71E-08	2.847766	2.847766	1.509831	1.509831	2.01E-07	637	605	542	594.6667	1016	553	881	816.6667
SEN2155	9	2	4.333333	1.371429	6.3E-05	2.863466	2.863466	1.517762	1.517762	0.000166	14	12	9	11.66667	18	14	16	16
yedJ	167	72	82	1.371041	5.15E-10	2.908682	2.908682	1.540365	1.540365	2.86E-09	276	204	183	221	350	283	276	303
SEN1809	13	7	5.666667	1.369565	2.26E-05	2.865165	2.865165	1.518618	1.518618	6.36E-05	13	20	13	15.33333	26	22	15	21
wzxC	16	2	9.666667	1.367089	2.17E-07	2.881401	2.881401	1.526771	1.526771	8.5E-07	28	28	23	26.33333	39	39	30	36
ybaM	47	10	26.66667	1.366972	2.66E-09	2.880052	2.880052	1.526095	1.526095	1.34E-08	77	65	76	72.66667	86	100	112	99.33333
pduV	12	1	5	1.365854	4.19E-05	2.833479	2.833479	1.502574	-1.50257	0.000114	14	13	14	13.66667	20	12	24	18.66667
yjfY	60	14	24	1.363636	4.3E-09	2.847735	2.847735	1.509815	1.509815	2.11E-08	76	69	53	66	113	62	95	90

Feature ID	Experiment - Range (original values)	Experiment - IQR (original values)	Experiment - Difference (original values)	Experiment - Fold Change (original values)	EDGE test: WT H202 vs WT NT , tagwise dispersions - P-value	EDGE test: WT H202 vs WT NT , tagwise dispersions - Fold change	WT H202 vs WT NT ABS|FC|	WT H202 vs WT NT Log2FC	WT H202 vs WT NT Log2FC +-	EDGE test: WT H202 vs WT NT , tagwise dispersions - FDR p-value correction	WT NT - WT.1.S22 - Expression values	WT NT - WT.2.S23 - Expression values	WT NT - WT.3.S24 - Expression values	WT NT - Means	WT H202 - WT.1.H2O2 - Expression values	WT H202 - WT.2.H2O2 - Expression values	WT H202 - WT.3.H2O2 - Expression values	WT H202 - Means
SEN1172	16	4	8.333333	1.357143	1.51E-06	2.855196	2.855196	1.51359	1.51359	5.13E-06	21	25	24	23.33333	30	37	28	31.66667
SEN3896	35	15	20	1.357143	1.89E-09	2.838519	2.838519	1.505138	1.505138	9.6E-09	51	63	54	56	86	69	73	76
wcaI	33	2	11	1.354839	3.91E-05	2.897278	2.897278	1.534698	1.534698	0.000106	32	30	31	31	23	56	47	42
ilvM	156	46	32.33333	1.351449	0.000357	3.047427	3.047427	1.607592	-1.60759	0.000816	141	74	61	92	55	211	107	124.3333
SEN2669	368	34	67.66667	1.351211	0.00018	2.743881	2.743881	1.456218	1.456218	0.000438	197	198	183	192.6667	493	125	163	260.3333
cbiN	740	211	146.6667	1.345098	0.007904	2.710179	2.710179	1.438388	1.438388	0.013881	333	101	841	425	516	544	655	571.6667
SEN2004	22	5	16	1.342857	3.69E-09	2.804114	2.804114	1.487545	1.487545	1.84E-08	47	46	47	46.66667	68	52	68	62.66667
manC	45	14	18	1.339623	3.58E-07	2.805821	2.805821	1.488423	1.488423	1.35E-06	41	66	52	53	58	69	86	71
ybbN	814	224	424.3333	1.339105	1.36E-06	2.796944	2.796944	1.483851	1.483851	4.67E-06	1141	1328	1285	1251.333	1563	1509	1955	1675.667
yfeN	22	18	8	1.338028	4.16E-06	2.817374	2.817374	1.494351	1.494351	1.31E-05	38	16	17	23.66667	37	23	35	31.66667
SEN3984	14	2	3.333333	1.333333	0.000684	2.791063	2.791063	1.480815	1.480815	0.001493	17	4	9	10	11	11	18	13.33333
rna-AM93	24	7	9.666667	1.333333	6.32E-07	2.777518	2.777518	1.473796	-1.4738	2.3E-06	32	30	25	29	42	25	49	38.66667
SEN0830	10	0	4	1.333333	0.000112	2.777308	2.777308	1.473687	1.473687	0.000282	13	10	13	12	20	15	13	16
wcaD	16	4	6.666667	1.327869	1.88E-05	2.785576	2.785576	1.477976	1.477976	5.36E-05	21	16	24	20.33333	20	29	32	27
ssaQ	57	19	19	1.327586	7E-08	2.77352	2.77352	1.471718	1.471718	2.9E-07	71	57	46	58	103	52	76	77
safD	30	4	14.33333	1.325758	4.09E-08	2.761429	2.761429	1.465415	1.465415	1.76E-07	42	43	47	44	56	47	72	58.33333
kefB	386	163	249.3333	1.325359	2.75E-08	2.804535	2.804535	1.487761	1.487761	1.22E-07	812	766	721	766.3333	929	1011	1107	1015.667
SEN4279	113	60	44.33333	1.325183	4.05E-08	2.831462	2.831462	1.501547	1.501547	1.75E-07	187	104	118	136.3333	147	178	217	180.6667
hpaF	66	43	20.66667	1.324607	2.07E-05	2.907556	2.907556	1.539807	1.539807	5.86E-05	102	47	42	63.66667	55	108	90	84.33333
SEN2341	53	14	23.66667	1.322727	1.08E-09	2.803825	2.803825	1.487396	1.487396	5.71E-09	88	76	56	73.33333	92	90	109	97
cheM	11738	4897	4418.333	1.322435	2.06E-05	2.807166	2.807166	1.489114	-1.48911	5.85E-05	18435	13803	8871	13703	20609	13538	20217	18121.33
bcfF	34	11	13	1.322314	5.52E-08	2.775784	2.775784	1.472895	-1.4729	2.34E-07	50	42	29	40.33333	63	39	58	53.33333
SEN1133	412	105	213	1.320783	1.94E-08	2.789221	2.789221	1.479862	1.479862	8.85E-08	750	672	570	664	872	777	982	877
SEN2168	260	121	119.6667	1.320536	3.75E-09	2.815071	2.815071	1.493172	1.493172	1.86E-08	478	352	290	373.3333	473	456	550	493
yfaZ	79	61	60	1.318584	4.36E-10	2.784441	2.784441	1.477388	1.477388	2.45E-09	212	175	178	188.3333	254	239	252	248.3333
lyxK	21	11	10.33333	1.316327	4.53E-07	2.780402	2.780402	1.475293	1.475293	1.69E-06	31	43	24	32.66667	42	45	42	43
SEN4250	15	7	3.666667	1.314286	0.000615	2.72327	2.72327	1.44534	1.44534	0.00135	14	14	7	11.66667	22	7	17	15.33333
SEN2172	27	2	9	1.313953	5.99E-07	2.773252	2.773252	1.471579	1.471579	2.19E-06	32	36	18	28.66667	34	34	45	37.66667
SEN1363	34	18	14.33333	1.313869	3.57E-08	2.757333	2.757333	1.463274	1.463274	1.55E-07	58	40	39	45.66667	62	45	73	60
SEN1419	14	5	4.666667	1.311111	2.6E-05	2.757727	2.757727	1.46348	1.46348	7.26E-05	22	15	8	15	22	17	20	19.66667
yaaI	21	12	9.333333	1.311111	1.62E-07	2.754911	2.754911	1.462006	1.462006	6.44E-07	39	27	24	30	45	32	41	39.33333
acrF	256	104	115	1.310531	7.56E-09	2.751085	2.751085	1.460001	-1.46	3.63E-08	453	349	309	370.3333	532	359	565	485.3333
invB	31	6	7.333333	1.309859	0.000257	2.709182	2.709182	1.437857	1.437857	0.000607	13	38	20	23.66667	44	26	23	31

Feature ID	Experiment - Range (original values)	Experiment - IQR (original values)	Experiment - Difference (original values)	Experiment - Fold Change (original values)	EDGE test: WT H202 vs WT NT , tagwise dispersions - P-value	EDGE test: WT H202 vs WT NT , tagwise dispersions - Fold change	WT H202 vs WT NT ABS|FC|	WT H202 vs WT NT Log2FC	WT H202 vs WT NT Log2FC +-	EDGE test: WT H202 vs WT NT , tagwise dispersions - FDR p-value correction	WT NT - WT.1.S22 - Expression values	WT NT - WT.2.S23 - Expression values	WT NT - WT.3.S24 - Expression values	WT NT - Means	WT H202 - WT.1.H2O2 - Expression values	WT H202 - WT.2.H2O2 - Expression values	WT H202 - WT.3.H2O2 - Expression values	WT H202 - Means
cobS	247	53	94.66667	1.309368	2.86E-07	2.706338	2.706338	1.436342	-1.43634	1.1E-06	327	217	374	306	464	321	417	400.6667
SEN2875	20	4	9.666667	1.308511	2.3E-07	2.747336	2.747336	1.458034	1.458034	8.95E-07	32	33	29	31.33333	36	38	49	41
lpfC	130	20	59.66667	1.30809	1.49E-09	2.747845	2.747845	1.458301	1.458301	7.7E-09	200	220	161	193.6667	258	211	291	253.3333
yjfQ	313	52	188.3333	1.307734	3.47E-08	2.734371	2.734371	1.451209	1.451209	1.51E-07	635	614	587	612	835	666	900	800.3333
fhuB	86	18	44	1.302752	1.72E-09	2.755807	2.755807	1.462475	1.462475	8.87E-09	150	158	128	145.3333	168	186	214	189.3333
SEN4029	24	4	8.666667	1.302326	5.69E-07	2.734623	2.734623	1.451342	1.451342	2.09E-06	33	29	24	28.66667	48	34	30	37.33333
SEN1002	16	5	5.333333	1.301887	8.88E-05	2.715213	2.715213	1.441065	1.441065	0.000228	14	18	21	17.66667	16	23	30	23
SEN3828	105	8	49	1.30123	3.1E-10	2.741082	2.741082	1.454745	1.454745	1.77E-09	183	175	130	162.6667	224	176	235	211.6667
SEN1356	218	74	69.33333	1.300144	5.25E-08	2.757773	2.757773	1.463504	1.463504	2.23E-07	317	222	154	231	372	233	296	300.3333
SEN2190	8	4	3	1.3	0.00054	2.720602	2.720602	1.443926	1.443926	0.001197	14	9	7	10	15	13	11	13
SEN1916	11	6	3	1.3	0.00104	2.694991	2.694991	1.430281	1.430281	0.002181	12	12	6	10	16	6	17	13
SEN1778	36	4	5.666667	1.298246	0.001359	2.663824	2.663824	1.413499	1.413499	0.002782	23	19	15	19	46	10	18	24.66667
SEN1890	9	2	5.333333	1.296296	5.86E-06	2.709269	2.709269	1.437904	1.437904	1.81E-05	19	18	17	18	24	20	26	23.33333
bcsF	27	15	12.66667	1.294574	7.9E-08	2.711158	2.711158	1.438909	1.438909	3.25E-07	40	55	34	43	60	46	61	55.66667
SEN4037	118	22	53	1.2939	9.27E-10	2.71931	2.71931	1.443241	1.443241	4.95E-09	205	192	144	180.3333	255	183	262	233.3333
yhhW	415	87	97.33333	1.292879	2.5E-06	2.665558	2.665558	1.414437	1.414437	8.15E-06	366	352	279	332.3333	658	243	388	429.6667
livM	42	25	33.33333	1.292398	1.32E-09	2.724644	2.724644	1.446068	-1.44607	6.91E-09	114	116	112	114	139	149	154	147.3333
SEN2342	39	8	13.66667	1.29078	8.79E-08	2.743984	2.743984	1.456272	1.456272	3.58E-07	61	52	28	47	60	55	67	60.66667
oat	88	12	25.33333	1.284644	6.94E-07	2.652724	2.652724	1.407475	1.407475	2.51E-06	91	94	82	89	125	65	153	114.3333
SEN1357	153	34	75.33333	1.284635	7.56E-09	2.671667	2.671667	1.41774	1.41774	3.63E-08	289	241	264	264.6667	394	255	371	340
rsmC	158	29	75.66667	1.284461	2.34E-09	2.70051	2.70051	1.433232	1.433232	1.18E-08	302	273	223	266	367	277	381	341.6667
astC	66	12	14.66667	1.283871	9.04E-06	2.684074	2.684074	1.424424	-1.42442	2.71E-05	60	59	36	51.66667	102	47	50	66.33333
pduX	768	162	284.6667	1.280276	7.3E-07	2.666249	2.666249	1.414812	1.414812	2.63E-06	1101	1064	882	1015.667	1650	939	1312	1300.333
uvrB	820	326	479	1.275287	2.56E-06	2.699771	2.699771	1.432837	1.432837	8.32E-06	1898	1795	1527	1740	2189	2121	2347	2219
SEN2005	45	8	17.66667	1.270408	3.82E-08	2.641204	2.641204	1.401196	1.401196	1.65E-07	66	69	61	65.33333	105	60	84	83
yccA	1410	1042	582	1.268864	1.24E-05	2.682312	2.682312	1.423477	1.423477	3.62E-05	2919	1766	1809	2164.667	3176	2213	2851	2746.667
yneG	67	17	37	1.26747	3.9E-08	2.690844	2.690844	1.428059	1.428059	1.68E-07	136	140	139	138.3333	156	203	167	175.3333
SEN0656	168	20	72.66667	1.26553	3.75E-09	2.658877	2.658877	1.410817	1.410817	1.86E-08	305	286	230	273.6667	398	285	356	346.3333
rpmJ2	152	40	45.33333	1.262548	1.04E-07	2.633543	2.633543	1.397005	1.397005	4.2E-07	212	151	155	172.6667	303	156	195	218
glnK	13	4	2	1.26087	0.002749	2.633924	2.633924	1.397214	-1.39721	0.005282	15	2	6	7.666667	11	8	10	9.666667
srlD	3217	705	542.6667	1.260355	0.004099	2.53894	2.53894	1.344227	1.344227	0.00765	933	2499	2821	2084.333	1937	1794	4150	2627
SEN0785	6	3	2.333333	1.259259	0.001219	2.616255	2.616255	1.387503	1.387503	0.002521	9	6	12	9	12	12	10	11.33333
stfG	11	4	3	1.257143	0.000363	2.637443	2.637443	1.39914	1.39914	0.000829	18	10	7	11.66667	17	13	14	14.66667

Feature ID	Experiment - Range (original values)	Experiment - IQR (original values)	Experiment - Difference (original values)	Experiment - Fold Change (original values)	EDGE test: WT H202 vs WT NT , tagwise dispersions - P-value	EDGE test: WT H202 vs WT NT , tagwise dispersions - Fold change	WT H202 vs WT NT ABS|FC|	WT H202 vs WT NT Log2FC	WT H202 vs WT NT Log2FC +-	EDGE test: WT H202 vs WT NT , tagwise dispersions - FDR p-value correction	WT NT - WT.1.S22 - Expression values	WT NT - WT.2.S23 - Expression values	WT NT - WT.3.S24 - Expression values	WT NT - Means	WT H202 - WT.1.H2O2 - Expression values	WT H202 - WT.2.H2O2 - Expression values	WT H202 - WT.3.H2O2 - Expression values	WT H202 - Means
ydeJ	25	9	6	1.257143	6.95E-05	2.58715	2.58715	1.371364	1.371364	0.000181	16	30	24	23.33333	41	19	28	29.33333
bcfG	63	14	19.33333	1.256637	5.73E-08	2.665882	2.665882	1.414613	-1.41461	2.41E-07	98	73	55	75.33333	118	87	79	94.66667
eutL	149	68	34.66667	1.253659	9.07E-06	2.712485	2.712485	1.439615	1.439615	2.72E-05	233	84	93	136.6667	157	161	196	171.3333
SEN4352	141	91	54.33333	1.253106	7.94E-08	2.630959	2.630959	1.395589	1.395589	3.26E-07	279	177	188	214.6667	292	197	318	269
SEN0918	26	10	6.666667	1.25	2.03E-05	2.619868	2.619868	1.389494	1.389494	5.75E-05	39	20	21	26.66667	46	23	31	33.33333
SEN1066	101	24	52.66667	1.25	2.05E-08	2.615741	2.615741	1.38722	1.38722	9.31E-08	210	206	216	210.6667	249	234	307	263.3333
sodC	2942	1197	1389.333	1.249626	1.95E-05	2.657608	2.657608	1.410128	1.410128	5.56E-05	6587	4720	5390	5565.667	6056	7147	7662	6955
ybaN	47	9	18.33333	1.246637	5.71E-09	2.619299	2.619299	1.38918	1.38918	2.78E-08	85	77	61	74.33333	108	76	94	92.66667
SEN0153	64	20	17	1.246377	2.96E-07	2.636452	2.636452	1.398598	1.398598	1.14E-06	96	67	44	69	108	65	85	86
SEN4254	68	31	37.66667	1.246187	9.14E-10	2.62913	2.62913	1.394586	1.394586	4.9E-09	178	147	134	153	200	170	202	190.6667
htpG	3733	1202	916.6667	1.245843	0.00045	2.70602	2.70602	1.436172	1.436172	0.001013	3195	4844	3147	3728.667	2927	6660	4349	4645.333
SEN1436	226	42	57.33333	1.245714	7.1E-07	2.571432	2.571432	1.362572	1.362572	2.57E-06	240	251	209	233.3333	408	182	282	290.6667
SEN1506	18	7	8	1.244898	4.36E-07	2.632233	2.632233	1.396287	1.396287	1.63E-06	40	33	25	32.66667	43	41	38	40.66667
hofB	47	38	14.66667	1.243094	1.85E-06	2.610348	2.610348	1.384242	-1.38424	6.18E-06	85	52	44	60.33333	87	47	91	75
yciE	14	3	2.666667	1.242424	0.001	2.593928	2.593928	1.375139	1.375139	0.002106	13	15	5	11	19	10	12	13.66667
fimW	19	2	7	1.241379	9.31E-07	2.594019	2.594019	1.375189	1.375189	3.28E-06	30	31	26	29	45	29	34	36
ybbA	157	79	51.66667	1.241058	7.77E-08	2.634296	2.634296	1.397418	1.397418	3.2E-07	296	193	154	214.3333	311	215	272	266
stfD	14	10	4.333333	1.240741	0.000118	2.647408	2.647408	1.40458	1.40458	0.000296	27	14	13	18	16	27	24	22.33333
SEN0998	10	0	2	1.24	0.004129	2.549306	2.549306	1.350105	1.350105	0.0077	5	8	12	8.333333	15	8	8	10.33333
stpA	133	37	48.66667	1.237398	1.52E-08	2.598699	2.598699	1.37779	1.37779	7.04E-08	233	214	168	205	264	196	301	253.6667
SEN2633	31	9	7.666667	1.237113	7.9E-06	2.61244	2.61244	1.385398	1.385398	2.4E-05	38	37	22	32.33333	53	38	29	40
yeeX	2375	491	811.3333	1.231964	2.7E-05	2.558792	2.558792	1.355463	1.355463	7.53E-05	3662	3660	3171	3497.667	5483	3108	4336	4309
yfeR	30	6	12	1.230769	8.29E-08	2.603759	2.603759	1.380596	1.380596	3.39E-07	60	54	42	52	72	64	56	64
SEN1713	46	26	17.33333	1.229075	6.97E-08	2.580223	2.580223	1.367496	1.367496	2.89E-07	98	57	72	75.66667	103	74	102	93
fnr	4725	114	727.3333	1.228314	0.001077	2.538376	2.538376	1.343906	-1.34391	0.00225	4227	2662	2668	3185.667	6844	2119	2776	3913
yadS	355	187	149.6667	1.228266	2.22E-07	2.581962	2.581962	1.368468	1.368468	8.68E-07	783	596	588	655.6667	943	669	804	805.3333
hha	456	202	171.3333	1.224356	4.14E-07	2.569848	2.569848	1.361683	1.361683	1.55E-06	915	737	639	763.6667	1095	713	997	935
SEN3573	233	22	102.6667	1.222865	3.36E-08	2.571461	2.571461	1.362588	1.362588	1.47E-07	499	484	399	460.6667	581	477	632	563.3333
livH	32	1	17	1.218884	8.71E-09	2.549725	2.549725	1.350342	1.350342	4.14E-08	79	80	74	77.66667	106	80	98	94.66667
ytfE	96	24	32.33333	1.217978	1.04E-08	2.563798	2.563798	1.358283	1.358283	4.94E-08	169	157	119	148.3333	182	145	215	180.6667
nagE	17472	1542	2356	1.217933	0.002888	2.454924	2.454924	1.295679	1.295679	0.005537	10245	11787	10400	10810.67	22522	5050	11928	13166.67
fimD	32	16	14.33333	1.217172	3.43E-08	2.571263	2.571263	1.362477	1.362477	1.5E-07	82	55	61	66	87	77	77	80.33333
SEN3742A	66	29	28.66667	1.217172	1.54E-08	2.539494	2.539494	1.344541	1.344541	7.15E-08	128	153	115	132	181	124	177	160.6667

Feature ID	Experiment - Range (original values)	Experiment - IQR (original values)	Experiment - Difference (original values)	Experiment - Fold Change (original values)	EDGE test: WT H202 vs WT NT , tagwise dispersions - P-value	EDGE test: WT H202 vs WT NT , tagwise dispersions - Fold change	WT H202 vs WT NT ABS|FC|	WT H202 vs WT NT Log2FC	WT H202 vs WT NT Log2FC +-	EDGE test: WT H202 vs WT NT , tagwise dispersions - FDR p-value correction	WT NT - WT.1.S22 - Expression values	WT NT - WT.2.S23 - Expression values	WT NT - WT.3.S24 - Expression values	WT NT - Means	WT H202 - WT.1.H2O2 - Expression values	WT H202 - WT.2.H2O2 - Expression values	WT H202 - WT.3.H2O2 - Expression values	WT H202 - Means
SEN0315	128	75	64	1.215973	9.88E-09	2.571191	2.571191	1.362437	1.362437	4.69E-08	352	277	260	296.3333	367	326	388	360.3333
mgtB	180	11	59.33333	1.215758	6.2E-08	2.565157	2.565157	1.359047	1.359047	2.59E-07	303	288	234	275	299	290	414	334.3333
SEN3513	350	83	165.3333	1.215652	3.39E-07	2.549317	2.549317	1.350111	1.350111	1.29E-06	764	847	689	766.6667	955	802	1039	932
SEN3596	32	11	4.666667	1.215385	0.001639	2.596724	2.596724	1.376693	1.376693	0.003304	30	21	14	21.66667	11	25	43	26.33333
SEN4331A	97	44	38	1.211503	3.48E-08	2.538217	2.538217	1.343815	1.343815	1.52E-07	174	218	147	179.6667	232	177	244	217.6667
yjgD	1098	220	318.6667	1.211177	4.41E-05	2.490734	2.490734	1.316571	1.316571	0.000119	1371	1565	1591	1509	2422	1324	1737	1827.667
ycfQ	1322	271	237.6667	1.211072	7.94E-05	2.505021	2.505021	1.324822	1.324822	0.000205	1261	1194	923	1126	2160	838	1093	1363.667
SEN1971	1498	314	536	1.209894	5.9E-05	2.58465	2.58465	1.369969	1.369969	0.000156	2941	2093	2627	2553.667	2685	3591	2993	3089.667
SEN4294	213	144	75	1.209497	1.12E-07	2.546769	2.546769	1.348668	1.348668	4.51E-07	463	319	292	358	505	329	465	433
rpmE2	97	18	20.66667	1.207358	1.07E-06	2.529123	2.529123	1.338637	1.338637	3.73E-06	117	107	75	99.66667	172	89	100	120.3333
thiC	183	44	65	1.206349	4.75E-07	2.562386	2.562386	1.357488	1.357488	1.76E-06	334	329	282	315	290	385	465	380
SEN3373	225	61	44	1.205928	3.08E-05	2.457483	2.457483	1.297181	1.297181	8.5E-05	188	249	204	213.6667	355	130	288	257.6667
potG	43	12	16.66667	1.200803	5.66E-08	2.513408	2.513408	1.329645	-1.32964	2.39E-07	80	92	77	83	95	84	120	99.66667
ecnB	89	18	18.33333	1.20073	1.6E-06	2.547495	2.547495	1.349079	-1.34908	5.44E-06	128	92	54	91.33333	143	84	102	109.6667
uvrD	1253	64	572.3333	1.20021	1.84E-05	2.520322	2.520322	1.333608	1.333608	5.26E-05	2958	3022	2596	2858.667	3466	2978	3849	3431
araB	50	13	20	1.2	1.82E-08	2.511435	2.511435	1.328512	-1.32851	8.35E-08	104	108	88	100	127	95	138	120
creA	233	6	120.6667	1.199229	3.77E-07	2.504585	2.504585	1.324572	-1.32457	1.42E-06	613	607	597	605.6667	741	608	830	726.3333
ygiW	1033	39	264	1.196917	0.000117	2.549457	2.549457	1.35019	1.35019	0.000293	1356	1349	1317	1340.667	1002	1777	2035	1604.667
stbD	38	19	12	1.196721	3.47E-07	2.548822	2.548822	1.349831	1.349831	1.32E-06	85	51	47	61	68	70	81	73
glxK	51	14	13.66667	1.196172	2.45E-07	2.538299	2.538299	1.343862	1.343862	9.51E-07	94	66	49	69.66667	80	70	100	83.33333
SEN2800	12	3	3.333333	1.196078	0.000125	2.488136	2.488136	1.315065	1.315065	0.000312	14	21	16	17	26	16	19	20.33333
SEN0308	37	8	16.66667	1.195313	2.78E-08	2.49682	2.49682	1.320092	1.320092	1.24E-07	82	91	83	85.33333	119	88	99	102
23S rRNA-	40	17	4	1.193548	0.009672	2.621928	2.621928	1.390628	1.390628	0.016637	47	8	7	20.66667	29	25	20	24.66667
bolA	486	218	132.6667	1.190796	6.6E-06	2.555788	2.555788	1.353768	1.353768	2.03E-05	887	649	550	695.3333	615	833	1036	828
yfjF	499	190	161.3333	1.186656	7.45E-07	2.485011	2.485011	1.313252	1.313252	2.68E-06	987	858	748	864.3333	1247	797	1033	1025.667
SEN1435	75	32	17.33333	1.18638	8.97E-07	2.469688	2.469688	1.304329	1.304329	3.17E-06	105	101	73	93	144	69	118	110.3333
SEN3473	415	106	115	1.185884	2.74E-06	2.488243	2.488243	1.315127	1.315127	8.87E-06	758	470	628	618.6667	711	605	885	733.6667
SEN0365	48	18	13.33333	1.185185	8.1E-08	2.49512	2.49512	1.319109	1.319109	3.32E-07	87	75	54	72	102	68	86	85.33333
SEN0420	40	11	9	1.184932	1.43E-05	2.437903	2.437903	1.285641	1.285641	4.14E-05	44	46	56	48.66667	79	39	55	57.66667
yiiR	182	103	58.33333	1.183631	8.73E-08	2.49291	2.49291	1.317831	1.317831	3.56E-07	399	297	257	317.6667	439	293	396	376
SEN2282	917	229	183.6667	1.18239	0.000186	2.548058	2.548058	1.349398	1.349398	0.000452	1104	1042	875	1007	627	1401	1544	1190.667
SEN0996	46	23	14	1.179487	1.44E-07	2.468241	2.468241	1.303483	1.303483	5.72E-07	95	67	72	78	113	70	93	92
yhjC	75	12	25	1.177725	1.3E-07	2.437524	2.437524	1.285416	1.285416	5.19E-07	133	144	145	140.6667	197	122	178	165.6667

Feature ID	Experiment - Range (original values)	Experiment - IQR (original values)	Experiment - Difference (original values)	Experiment - Fold Change (original values)	EDGE test: WT H202 vs WT NT , tagwise dispersions - P-value	EDGE test: WT H202 vs WT NT , tagwise dispersions - Fold change	WT H202 vs WT NT ABS|FC|	WT H202 vs WT NT Log2FC	WT H202 vs WT NT Log2FC +-	EDGE test: WT H202 vs WT NT , tagwise dispersions - FDR p-value correction	WT NT - WT.1.S22 - Expression values	WT NT - WT.2.S23 - Expression values	WT NT - WT.3.S24 - Expression values	WT NT - Means	WT H202 - WT.1.H2O2 - Expression values	WT H202 - WT.2.H2O2 - Expression values	WT H202 - WT.3.H2O2 - Expression values	WT H202 - Means
spaO	11	5	4.666667	1.175	9.04E-06	2.474414	2.474414	1.307087	1.307087	2.71E-05	27	32	21	26.66667	32	32	30	31.33333
SEN1146	13	6	3	1.173077	0.00012	2.456589	2.456589	1.296657	1.296657	0.000299	20	21	11	17.33333	22	15	24	20.33333
htgA	53	24	16.66667	1.17301	9.76E-08	2.458762	2.458762	1.297932	1.297932	3.95E-07	100	112	77	96.33333	121	88	130	113
yhcP	155	40	43.66667	1.172596	7.68E-08	2.468714	2.468714	1.30376	1.30376	3.16E-07	296	258	205	253	360	245	285	296.6667
yqjI	192	75	99.33333	1.171857	2.19E-06	2.48676	2.48676	1.314268	1.314268	7.26E-06	556	631	547	578	588	739	705	677.3333
acrE	41	13	15	1.171756	7.66E-07	2.427669	2.427669	1.279572	1.279572	2.75E-06	70	90	102	87.33333	107	89	111	102.3333
ydfZ	342	87	47.66667	1.171463	0.000358	2.398247	2.398247	1.26198	1.26198	0.000819	304	180	350	278	522	238	217	325.6667
manY	14244	5605	2529.667	1.171	0.002747	2.473873	2.473873	1.306771	1.306771	0.00528	9358	15911	19111	14793.33	11381	23602	16986	17323
yjjY	1002	200	275.6667	1.170832	4.31E-05	2.424814	2.424814	1.277874	1.277874	0.000116	1565	1738	1538	1613.667	2398	1396	1874	1889.333
ribA	1025	470	319.6667	1.168187	2.58E-05	2.448484	2.448484	1.291889	1.291889	7.21E-05	2212	1832	1658	1900.667	2683	1742	2236	2220.333
thiD	60	33	16	1.165517	2.99E-07	2.475622	2.475622	1.307791	1.307791	1.14E-06	135	80	75	96.66667	113	97	128	112.6667
SEN2471	519	130	148	1.164811	2.04E-06	2.447943	2.447943	1.29157	1.29157	6.78E-06	1063	856	775	898	1294	858	986	1046
fljB	347746	150330	63825.33	1.163417	0.000765	2.496985	2.496985	1.320187	1.320187	0.001654	414331	453849	303519	390566.3	265523	484383	613269	454391.7
SEN2465	504	188	163.3333	1.15615	2.71E-06	2.451484	2.451484	1.293655	1.293655	8.77E-06	1267	1029	842	1046	1217	1065	1346	1209.333
SEN1493	361	151	58	1.156054	3.16E-05	2.529645	2.529645	1.338935	1.338935	8.71E-05	604	268	243	371.6667	419	486	384	429.6667
yghB	1979	398	329	1.156023	0.000202	2.396968	2.396968	1.261211	1.261211	0.000488	2379	2163	1784	2108.667	3555	1576	2182	2437.667
grxB	2838	1719	813.6667	1.155935	0.000136	2.482996	2.482996	1.312082	1.312082	0.000338	6825	4490	4339	5218	4709	6209	7177	6031.667
lpxC	12303	8356	3774.667	1.15574	0.000103	2.429899	2.429899	1.280897	1.280897	0.00026	29636	23277	19798	24237	32101	21280	30654	28011.67
SEN2874	66	24	18	1.154286	5.88E-08	2.449854	2.449854	1.292696	1.292696	2.48E-07	148	120	82	116.6667	146	117	141	134.6667
treF	525	127	146	1.153576	2.67E-06	2.435768	2.435768	1.284377	1.284377	8.67E-06	1106	954	792	950.6667	1050	923	1317	1096.667
yjbA	38	28	17.33333	1.153392	5.64E-08	2.452401	2.452401	1.294195	1.294195	2.38E-07	137	103	99	113	126	134	131	130.3333
SEN1507	30	14	6.333333	1.152	2.92E-05	2.373974	2.373974	1.247304	1.247304	8.09E-05	33	47	45	41.66667	63	33	48	48
ssb	1021	184	442.6667	1.151598	7.12E-05	2.405327	2.405327	1.266233	1.266233	0.000185	2640	3152	2968	2920	3661	3113	3314	3362.667
SEN2880	514	284	102.3333	1.151083	9.38E-06	2.454099	2.454099	1.295193	1.295193	2.8E-05	988	570	474	677.3333	860	625	854	779.6667
SEN2144	205	48	91.66667	1.150685	1.77E-06	2.450977	2.450977	1.293357	1.293357	5.96E-06	652	604	569	608.3333	647	774	679	700
SEN2929	17	3	6	1.15	1.63E-06	2.436582	2.436582	1.284859	1.284859	5.52E-06	46	43	31	40	44	48	46	46
SEN3892	18	3	10.66667	1.148837	1.38E-07	2.399253	2.399253	1.262585	1.262585	5.51E-07	70	71	74	71.66667	88	71	88	82.33333
feoA	155	105	34	1.148472	1.81E-06	2.411723	2.411723	1.270064	1.270064	6.08E-06	281	243	163	229	295	176	318	263
stiH	79	17	29	1.146465	4.34E-08	2.435596	2.435596	1.284275	1.284275	1.86E-07	229	204	161	198	221	220	240	227
stfC	69	39	14.33333	1.14527	9.88E-07	2.417784	2.417784	1.273686	1.273686	3.46E-06	124	107	65	98.66667	122	83	134	113
sbcD	229	18	63	1.144385	5.5E-07	2.414751	2.414751	1.271874	1.271874	2.03E-06	469	455	385	436.3333	437	447	614	499.3333
SEN1503	9	3	2	1.142857	0.000703	2.380685	2.380685	1.251377	1.251377	0.001529	14	16	12	14	21	12	15	16
SEN1132	3	1	1	1.142857	0.009474	2.367486	2.367486	1.243356	1.243356	0.016347	7	6	8	7	9	7	8	8

Feature ID	Experiment - Range (original values)	Experiment - IQR (original values)	Experiment - Difference (original values)	Experiment - Fold Change (original values)	EDGE test: WT H202 vs WT NT , tagwise dispersions - P-value	EDGE test: WT H202 vs WT NT , tagwise dispersions - Fold change	WT H202 vs WT NT ABS|FC|	WT H202 vs WT NT Log2FC	WT H202 vs WT NT Log2FC +-	EDGE test: WT H202 vs WT NT , tagwise dispersions - FDR p-value correction	WT NT - WT.1.S22 - Expression values	WT NT - WT.2.S23 - Expression values	WT NT - WT.3.S24 - Expression values	WT NT - Means	WT H202 - WT.1.H2O2 - Expression values	WT H202 - WT.2.H2O2 - Expression values	WT H202 - WT.3.H2O2 - Expression values	WT H202 - Means
SEN1409	123	26	23	1.138277	8.51E-07	2.367042	2.367042	1.243085	1.243085	3.02E-06	181	169	149	166.3333	258	135	175	189.3333
invI	9	1	1.333333	1.137931	0.006955	2.360312	2.360312	1.238978	1.238978	0.012376	7	14	8	9.666667	16	9	8	11
glpE	219	34	40.33333	1.134894	5.88E-06	2.37786	2.37786	1.249664	1.249664	1.82E-05	301	315	281	299	461	315	242	339.3333
yhdN	137	66	24.33333	1.134686	2.4E-06	2.368107	2.368107	1.243735	1.243735	7.86E-06	231	161	150	180.6667	268	131	216	205
SEN0268	79	41	23.33333	1.132325	1.29E-07	2.398268	2.398268	1.261993	1.261993	5.17E-07	225	146	158	176.3333	199	182	218	199.6667
yeaM	126	46	19.66667	1.131991	3.58E-06	2.369303	2.369303	1.244463	1.244463	1.14E-05	178	161	108	149	234	115	157	168.6667
bcfC	44	22	14.33333	1.131098	4.16E-08	2.387308	2.387308	1.255384	-1.25538	1.79E-07	129	110	89	109.3333	131	107	133	123.6667
nusB	556	39	149.3333	1.130044	2.73E-05	2.384692	2.384692	1.253803	1.253803	7.6E-05	1150	1167	1128	1148.333	1032	1273	1588	1297.667
SEN2256	6	2	1.333333	1.129032	0.00376	2.349643	2.349643	1.232442	1.232442	0.007078	9	12	10	10.33333	15	11	9	11.66667
yjgA	261	23	57.33333	1.12675	1.81E-06	2.332668	2.332668	1.221981	1.221981	6.08E-06	438	461	458	452.3333	651	390	488	509.6667
prgH	79	38	16.33333	1.126615	3.86E-07	2.361945	2.361945	1.239975	1.239975	1.45E-06	137	153	97	129	176	111	149	145.3333
SEN2632	281	136	77	1.126299	1.71E-06	2.38647	2.38647	1.254878	1.254878	5.78E-06	725	647	457	609.6667	738	589	733	686.6667
yabN	266	138	60.33333	1.12552	1.07E-06	2.373259	2.373259	1.24687	1.24687	3.72E-06	578	489	375	480.6667	560	422	641	541
SEN4293	206	59	47.33333	1.12522	9.67E-07	2.358243	2.358243	1.237713	1.237713	3.4E-06	457	329	348	378	535	341	400	425.3333
SEN0907	7	1	1.666667	1.125	0.001067	2.351253	2.351253	1.23343	1.23343	0.002235	15	14	11	13.33333	18	13	14	15
SEN1799	61	22	21.33333	1.123552	8.83E-08	2.355535	2.355535	1.236055	1.236055	3.59E-07	192	154	172	172.6667	215	170	197	194
rnt	157	80	73.33333	1.122905	1.25E-06	2.361882	2.361882	1.239937	1.239937	4.3E-06	652	572	566	596.6667	685	602	723	670
SEN3673	24	6	3.666667	1.122222	0.000108	2.315853	2.315853	1.211544	1.211544	0.000273	28	28	34	30	45	21	35	33.66667
nxiA	108	54	20.33333	1.122	7.47E-07	2.34136	2.34136	1.227347	-1.22735	2.69E-06	199	158	143	166.6667	236	128	197	187
ygaF	21	8	4.666667	1.121739	4.09E-06	2.358344	2.358344	1.237774	1.237774	1.29E-05	47	38	30	38.33333	51	35	43	43
pfkA	561	285	205	1.121686	4.74E-05	2.382988	2.382988	1.252772	-1.25277	0.000127	1921	1600	1533	1684.667	1690	1885	2094	1889.667
ybeS	22	1	4.666667	1.119658	4.89E-06	2.364758	2.364758	1.241692	1.241692	1.53E-05	48	39	30	39	40	39	52	43.66667
SEN2971	50	32	9	1.119469	2.78E-06	2.359047	2.359047	1.238204	1.238204	8.99E-06	96	79	51	75.33333	92	60	101	84.33333
purT	48	6	18.66667	1.118393	3.55E-07	2.345894	2.345894	1.230138	-1.23014	1.35E-06	155	156	162	157.6667	161	165	203	176.3333
yjiO	128	24	24.33333	1.116613	1.73E-06	2.304848	2.304848	1.204672	1.204672	5.85E-06	194	218	214	208.6667	296	168	235	233
fhuD	74	28	16	1.115942	8.23E-07	2.391563	2.391563	1.257953	1.257953	2.93E-06	173	141	100	138	130	158	174	154
rarD	141	65	24.66667	1.115806	2.03E-06	2.319276	2.319276	1.213675	-1.21367	6.76E-06	214	248	177	213	306	165	242	237.6667
rtcB	234	68	46	1.115578	1.56E-05	2.407973	2.407973	1.267819	1.267819	4.51E-05	421	425	348	398	397	582	353	444
SEN3474	519	234	75.33333	1.114954	2.56E-05	2.331404	2.331404	1.221199	1.221199	7.16E-05	818	657	491	655.3333	993	474	725	730.6667
ybhO	24	14	3.666667	1.114583	3.82E-05	2.345415	2.345415	1.229843	1.229843	0.000104	42	35	19	32	43	25	39	35.66667
lplA	331	206	71.33333	1.110423	3.98E-06	2.325521	2.325521	1.217554	1.217554	1.26E-05	759	634	545	646	866	535	751	717.3333
cobU	154	31	23.33333	1.109375	0.000107	2.290665	2.290665	1.195767	1.195767	0.00027	196	145	299	213.3333	261	227	222	236.6667
SEN3614	479	114	43	1.108495	0.000239	2.283796	2.283796	1.191434	1.191434	0.000568	430	443	316	396.3333	723	244	351	439.3333

Feature ID	Experiment - Range (original values)	Experiment - IQR (original values)	Experiment - Difference (original values)	Experiment - Fold Change (original values)	EDGE test: WT H202 vs WT NT , tagwise dispersions - P-value	EDGE test: WT H202 vs WT NT , tagwise dispersions - Fold change	WT H202 vs WT NT ABS|FC|	WT H202 vs WT NT Log2FC	WT H202 vs WT NT Log2FC +-	EDGE test: WT H202 vs WT NT , tagwise dispersions - FDR p-value correction	WT NT - WT.1.S22 - Expression values	WT NT - WT.2.S23 - Expression values	WT NT - WT.3.S24 - Expression values	WT NT - Means	WT H202 - WT.1.H2O2 - Expression values	WT H202 - WT.2.H2O2 - Expression values	WT H202 - WT.3.H2O2 - Expression values	WT H202 - Means
SEN2386	249	91	35	1.106816	3.87E-06	2.358	2.358	1.237563	1.237563	1.23E-05	462	308	213	327.6667	417	290	381	362.6667
pspF	130	91	27	1.105606	7.92E-07	2.319427	2.319427	1.213768	1.213768	2.83E-06	305	255	207	255.6667	337	210	301	282.6667
prgK	65	16	13.66667	1.105398	6.96E-07	2.347783	2.347783	1.231299	-1.2313	2.52E-06	140	148	101	129.6667	124	140	166	143.3333
SEN2631	32	15	3.333333	1.105263	0.000267	2.331412	2.331412	1.221204	1.221204	0.000629	39	40	16	31.66667	33	24	48	35
ybbK	929	358	114.6667	1.104496	0.000113	2.39478	2.39478	1.259893	1.259893	0.000284	1683	855	754	1097.333	1054	1213	1369	1212
SEN3012	170	20	14	1.101205	0.000446	2.284385	2.284385	1.191806	1.191806	0.001004	194	109	112	138.3333	249	79	129	152.3333
rna-AM93	15	5	1	1.1	0.014701	2.286796	2.286796	1.193328	1.193328	0.024334	15	10	5	10	20	5	8	11
ytgA	37	9	4	1.097561	0.000288	2.304291	2.304291	1.204323	1.204323	0.000672	62	27	34	41	64	31	40	45
SEN4082	20	16	2.333333	1.097222	0.000517	2.29963	2.29963	1.201402	1.201402	0.001152	35	21	16	24	32	15	32	26.33333
SEN0364	58	48	8.333333	1.096525	1.24E-05	2.303091	2.303091	1.203571	1.203571	3.61E-05	118	78	63	86.33333	111	60	113	94.66667
SEN2609	289	73	68	1.09564	7.36E-06	2.323943	2.323943	1.216575	1.216575	2.25E-05	789	721	623	711	676	749	912	779
yeaQ	128	66	14	1.09417	0.000241	2.386172	2.386172	1.254698	1.254698	0.000571	183	146	117	148.6667	87	215	186	162.6667
rbsA	269	36	17.33333	1.093525	0.001279	2.227383	2.227383	1.155349	-1.15535	0.002634	196	195	165	185.3333	359	90	159	202.6667
SEN0392	142	77	24.33333	1.093231	5.76E-06	2.359818	2.359818	1.238675	1.238675	1.78E-05	339	247	197	261	231	317	308	285.3333
adhE	7593	1842	1286.667	1.090967	0.000801	2.320116	2.320116	1.214197	1.214197	0.001722	13923	15176	13334	14144.33	17982	17922	10389	15431
SEN1004	80	27	20	1.09009	3.26E-07	2.292773	2.292773	1.197094	1.197094	1.24E-06	237	232	197	222	239	210	277	242
SEN1003	19	4	3.666667	1.089431	4.76E-05	2.306477	2.306477	1.205691	1.205691	0.000128	45	34	44	41	40	53	41	44.66667
ybiO	138	30	34.33333	1.088185	6.09E-07	2.29642	2.29642	1.199387	1.199387	2.23E-06	432	393	343	389.3333	410	380	481	423.6667
SEN4040	250	161	54.66667	1.086316	4.37E-06	2.278581	2.278581	1.188136	1.188136	1.38E-05	708	645	547	633.3333	730	542	792	688
SEN3018	483	170	65.66667	1.085914	0.000198	2.3273	2.3273	1.218657	1.218657	0.000479	757	853	683	764.3333	518	971	1001	830
manX	16277	8610	1644.333	1.085565	0.006712	2.297173	2.297173	1.19986	-1.19986	0.011998	12620	19870	25162	19217.33	12539	28816	21230	20861.67
yijP	319	226	77.33333	1.085044	7.34E-06	2.278516	2.278516	1.188094	1.188094	2.25E-05	1080	793	855	909.3333	1037	811	1112	986.6667
yjbG	12	5	1	1.083333	0.009256	2.278273	2.278273	1.18794	1.18794	0.016001	10	16	10	12	6	15	18	13
invJ	17	5	2.666667	1.083333	4.61E-05	2.277169	2.277169	1.187241	1.187241	0.000124	29	42	25	32	40	34	30	34.66667
SEN1790	27	4	7.333333	1.081784	3.17E-07	2.282095	2.282095	1.190359	1.190359	1.21E-06	101	88	80	89.66667	107	92	92	97
yfiE	138	68	24	1.081633	6.8E-07	2.290624	2.290624	1.195741	1.195741	2.47E-06	365	290	227	294	346	270	338	318
yhbH	4646	1446	583	1.081254	0.000486	2.321812	2.321812	1.215251	1.215251	0.001087	10165	5841	5519	7175	6612	7287	9375	7758
SEN0100	20	9	2.333333	1.08046	0.000327	2.270003	2.270003	1.182694	1.182694	0.000754	34	23	30	29	41	32	21	31.33333
SEN0307	49	24	8.333333	1.080128	1.1E-06	2.26138	2.26138	1.177204	1.177204	3.84E-06	122	94	96	104	118	85	134	112.3333
ccmA	11	4	1.666667	1.079365	0.000247	2.273197	2.273197	1.184723	1.184723	0.000583	28	17	18	21	22	22	24	22.66667
ydiZ	324	40	58.33333	1.077126	1.1E-05	2.283542	2.283542	1.191273	1.191273	3.23E-05	919	700	650	756.3333	740	730	974	814.6667
ygiN	527	178	94.33333	1.077028	5.92E-05	2.289073	2.289073	1.194763	1.194763	0.000156	1299	1254	1121	1224.667	1036	1358	1563	1319
cbiP	806	149	70.66667	1.076673	0.001075	2.212081	2.212081	1.145404	1.145404	0.002246	869	545	1351	921.6667	1018	897	1062	992.3333

Feature ID	Experiment - Range (original values)	Experiment - IQR (original values)	Experiment - Difference (original values)	Experiment - Fold Change (original values)	EDGE test: WT H202 vs WT NT , tagwise dispersions - P-value	EDGE test: WT H202 vs WT NT , tagwise dispersions - Fold change	WT H202 vs WT NT ABS|FC|	WT H202 vs WT NT Log2FC	WT H202 vs WT NT Log2FC +-	EDGE test: WT H202 vs WT NT , tagwise dispersions - FDR p-value correction	WT NT - WT.1.S22 - Expression values	WT NT - WT.2.S23 - Expression values	WT NT - WT.3.S24 - Expression values	WT NT - Means	WT H202 - WT.1.H2O2 - Expression values	WT H202 - WT.2.H2O2 - Expression values	WT H202 - WT.3.H2O2 - Expression values	WT H202 - Means
SEN2128	19	4	2.333333	1.076087	8.92E-05	2.264583	2.264583	1.179245	1.179245	0.000229	43	24	25	30.66667	43	27	29	33
pduG	54	7	5	1.075	5.47E-05	2.227739	2.227739	1.15558	1.15558	0.000146	70	64	66	66.66667	105	51	59	71.66667
ybbJ	404	179	51.66667	1.074771	2.54E-05	2.318609	2.318609	1.213259	1.213259	7.12E-05	933	611	529	691	638	790	800	742.6667
ilvD	2237	315	137.6667	1.073253	0.004275	2.371842	2.371842	1.246008	-1.24601	0.007952	2216	1779	1643	1879.333	928	3165	1958	2017
yidY	87	45	15.66667	1.072643	8.91E-07	2.259828	2.259828	1.176213	1.176213	3.15E-06	225	254	168	215.6667	242	197	255	231.3333
araD	76	26	6.333333	1.072519	3.94E-05	2.233053	2.233053	1.159018	-1.15902	0.000107	100	94	68	87.33333	136	60	85	93.66667
SEN3821	28	18	8.666667	1.072022	1.31E-06	2.232951	2.232951	1.158952	1.158952	4.5E-06	111	118	132	120.3333	134	114	139	129
yliH	1749	532	130	1.071025	0.001676	2.238632	2.238632	1.162618	1.162618	0.00337	2693	1388	1410	1830.333	2855	1106	1920	1960.333
SEN4252	13	8	3	1.070313	1.98E-05	2.262448	2.262448	1.177885	1.177885	5.62E-05	44	47	37	42.66667	39	48	50	45.66667
thiG	37	13	4	1.070175	2.97E-05	2.289482	2.289482	1.195021	1.195021	8.21E-05	78	52	41	57	49	62	72	61
SEN2970	45	18	7.333333	1.06962	8.46E-07	2.253866	2.253866	1.172402	1.172402	3E-06	126	101	89	105.3333	111	93	134	112.6667
yodD	16	4	3	1.068702	5.44E-06	2.250851	2.250851	1.170471	1.170471	1.69E-05	51	44	36	43.66667	52	42	46	46.66667
SEN1022	214	4	30	1.067771	2.71E-06	2.263031	2.263031	1.178256	1.178256	8.77E-06	548	424	356	442.6667	570	426	422	472.6667
fucU	45	12	9	1.066667	9.76E-06	2.255268	2.255268	1.173299	-1.1733	2.9E-05	131	131	143	135	114	159	159	144
yehR	60	26	3.666667	1.064706	0.000714	2.216324	2.216324	1.148169	1.148169	0.001551	65	66	39	56.66667	97	37	47	60.33333
SEN0800	15	3	1.333333	1.064516	0.000971	2.209065	2.209065	1.143436	1.143436	0.002053	20	18	24	20.66667	21	15	30	22
smg	5924	2667	658.6667	1.061998	0.000429	2.274167	2.274167	1.185338	1.185338	0.000968	14556	8684	8632	10624	11351	10965	11532	11282.67
SEN0313	38	15	5	1.061983	1.46E-06	2.238159	2.238159	1.162313	1.162313	4.98E-06	101	77	64	80.66667	102	70	85	85.66667
yeeN	745	403	136	1.060905	0.000327	2.205463	2.205463	1.141082	1.141082	0.000754	2246	1995	2458	2233	2398	1982	2727	2369
ratA	288	45	51	1.060666	1.09E-05	2.2437	2.2437	1.16588	-1.16588	3.21E-05	979	807	736	840.6667	848	803	1024	891.6667
ydiV	83	14	5.666667	1.05802	9.17E-05	2.180822	2.180822	1.124872	1.124872	0.000235	95	97	101	97.66667	155	72	83	103.3333
spaP	7	4	1	1.057692	0.000895	2.205889	2.205889	1.14136	1.14136	0.001911	16	20	16	17.33333	20	14	21	18.33333
SEN3589	84	13	8.666667	1.056277	3.12E-05	2.198638	2.198638	1.13661	1.13661	8.61E-05	126	205	131	154	210	134	144	162.6667
mtlR	305	21	30.66667	1.054762	2.32E-05	2.18453	2.18453	1.127323	1.127323	6.51E-05	565	568	547	560	643	412	717	590.6667
rluA	165	41	33.33333	1.054496	7.99E-06	2.237788	2.237788	1.162074	1.162074	2.43E-05	669	631	535	611.6667	597	638	700	645
aceA	127	22	8.666667	1.054167	4.39E-05	2.2616	2.2616	1.177344	1.177344	0.000118	177	215	88	160	155	168	183	168.6667
SEN1576	49	8	3.333333	1.052083	1.11E-05	2.241816	2.241816	1.164668	1.164668	3.26E-05	91	59	42	64	74	61	67	67.33333
SEN0460	339	128	38.33333	1.049935	1.71E-05	2.224494	2.224494	1.153477	1.153477	4.92E-05	982	678	643	767.6667	905	707	806	806
cadA	31842	29111	2319	1.049305	0.008271	2.20868	2.20868	1.143184	1.143184	0.014461	28863	59646	52592	47033.67	29121	58232	60705	49352.67
fxsA	703	119	25.66667	1.048734	0.005188	2.095695	2.095695	1.067429	-1.06743	0.009472	417	536	627	526.6667	950	247	460	552.3333
phoE	83	26	9.666667	1.046178	2.37E-06	2.231797	2.231797	1.158206	-1.15821	7.75E-06	250	211	167	209.3333	193	219	245	219
arcC	28	7	2.666667	1.044944	3.78E-06	2.211063	2.211063	1.14474	1.14474	1.2E-05	73	60	45	59.33333	63	56	67	62
SEN4086	11	3	0.666667	1.044444	0.002977	2.171139	2.171139	1.118452	1.118452	0.005696	14	18	13	15	21	10	16	15.66667

Feature ID	Experiment - Range (original values)	Experiment - IQR (original values)	Experiment - Difference (original values)	Experiment - Fold Change (original values)	EDGE test: WT H202 vs WT NT , tagwise dispersions - P-value	EDGE test: WT H202 vs WT NT , tagwise dispersions - Fold change	WT H202 vs WT NT ABS|FC|	WT H202 vs WT NT Log2FC	WT H202 vs WT NT Log2FC +-	EDGE test: WT H202 vs WT NT , tagwise dispersions - FDR p-value correction	WT NT - WT.1.S22 - Expression values	WT NT - WT.2.S23 - Expression values	WT NT - WT.3.S24 - Expression values	WT NT - Means	WT H202 - WT.1.H2O2 - Expression values	WT H202 - WT.2.H2O2 - Expression values	WT H202 - WT.3.H2O2 - Expression values	WT H202 - Means
cysC	13	8	4	1.044118	1.58E-06	2.205331	2.205331	1.140995	1.140995	5.38E-06	99	87	86	90.66667	91	95	98	94.66667
sseI	21	9	2	1.043796	1.58E-05	2.195491	2.195491	1.134544	1.134544	4.55E-05	50	52	35	45.66667	56	41	46	47.66667
thiE	43	8	2	1.043165	0.002181	2.216688	2.216688	1.148406	1.148406	0.004287	43	51	45	46.33333	23	56	66	48.33333
SEN0297	68	37	7	1.043121	1.06E-06	2.198816	2.198816	1.136727	1.136727	3.71E-06	179	179	129	162.3333	169	142	197	169.3333
yadR	380	288	40.33333	1.042411	2.82E-05	2.187753	2.187753	1.12945	1.12945	7.83E-05	1169	789	895	951	1096	795	1083	991.3333
SEN1787	12	6	0.333333	1.041667	0.031055	2.155026	2.155026	1.107705	1.107705	0.0483	10	10	4	8	15	3	7	8.333333
SEN1496	66	22	5.666667	1.037611	1.27E-06	2.191683	2.191683	1.132039	1.132039	4.38E-06	190	138	124	150.6667	181	133	155	156.3333
basS	383	234	34.33333	1.03705	6.82E-05	2.237408	2.237408	1.161828	1.161828	0.000178	1147	858	775	926.6667	764	1110	1009	961
SEN0343	29	5	1.666667	1.037037	5.86E-05	2.215369	2.215369	1.147547	1.147547	0.000155	61	42	32	45	47	51	42	46.66667
SEN1227	127	52	5.666667	1.035124	3.36E-05	2.190216	2.190216	1.131073	1.131073	9.21E-05	237	110	137	161.3333	182	130	189	167
SEN2746A	33	15	3.666667	1.034268	7.9E-07	2.183343	2.183343	1.126539	1.126539	2.83E-06	124	106	91	107	117	100	115	110.6667
cheR	2508	1153	148.3333	1.034239	0.000571	2.197081	2.197081	1.135588	-1.13559	0.001259	5341	4823	2833	4332.333	5049	3670	4723	4480.667
trpH	467	194	29.33333	1.033768	3.92E-05	2.159649	2.159649	1.110797	1.110797	0.000107	991	884	731	868.6667	1118	651	925	898
prgI	52	15	2	1.031088	0.000241	2.220737	2.220737	1.151038	1.151038	0.000571	75	85	33	64.33333	58	73	68	66.33333
SEN2525	2444	515	76.33333	1.027162	0.001757	2.131021	2.131021	1.091545	1.091545	0.003522	3171	2877	2383	2810.333	4371	1927	2362	2886.667
SEN4253	25	6	2	1.027149	8.09E-06	2.173087	2.173087	1.119746	1.119746	2.45E-05	74	86	61	73.66667	71	77	79	75.66667
yhbL	1035	417	73.66667	1.027007	0.000675	2.183504	2.183504	1.126645	1.126645	0.001475	3029	2512	2642	2727.667	2220	2929	3255	2801.333
SEN3239	93	33	5.666667	1.024709	3.48E-06	2.16343	2.16343	1.11332	1.11332	1.11E-05	283	190	215	229.3333	248	212	245	235
ygiD	269	89	6.333333	1.023869	0.000456	2.116598	2.116598	1.081747	1.081747	0.001026	303	291	202	265.3333	414	145	256	271.6667
SEN1501	22	3	0.666667	1.02381	0.000491	2.131171	2.131171	1.091646	1.091646	0.001098	32	27	25	28	40	18	28	28.66667
yiaL	13	1	0.333333	1.023256	0.006743	2.118192	2.118192	1.082833	1.082833	0.012043	15	14	14	14.33333	20	7	17	14.66667
yeaO	194	32	16	1.022814	1.56E-05	2.169489	2.169489	1.117355	1.117355	4.51E-05	809	680	615	701.3333	712	691	749	717.3333
yijO	115	24	8.333333	1.022543	4.78E-06	2.145624	2.145624	1.101398	1.101398	1.49E-05	387	373	349	369.6667	365	327	442	378
setB	18	9	1.666667	1.021097	2.61E-06	2.140779	2.140779	1.098136	1.098136	8.49E-06	82	82	73	79	86	69	87	80.66667
sopD	17	10	2	1.020906	1.41E-06	2.145268	2.145268	1.101158	1.101158	4.85E-06	99	100	88	95.66667	105	89	99	97.66667
gip	51	20	1.666667	1.018727	1.59E-05	2.180398	2.180398	1.124591	-1.12459	4.58E-05	122	74	71	89	83	95	94	90.66667
SEN2795	27	12	1.333333	1.018519	1.06E-05	2.160924	2.160924	1.111648	1.111648	3.15E-05	79	82	55	72	67	73	80	73.33333
cbiO	961	140	11.66667	1.017335	0.022145	2.056432	2.056432	1.040144	1.040144	0.03536	572	243	1204	673	712	602	740	684.6667
wzc	21	9	0.666667	1.017094	0.000344	2.15872	2.15872	1.110176	1.110176	0.00079	48	33	36	39	28	42	49	39.66667
safC	91	38	5.666667	1.016815	4.69E-06	2.159722	2.159722	1.110846	1.110846	1.47E-05	374	351	286	337	313	338	377	342.6667
SEN3897	21	3	1	1.016393	3.26E-05	2.133138	2.133138	1.092978	1.092978	8.96E-05	58	70	55	61	55	55	76	62
SEN0334	28	17	1.333333	1.015625	6.39E-05	2.149517	2.149517	1.104013	1.104013	0.000168	89	73	94	85.33333	71	99	90	86.66667
SEN1011	56	12	1.333333	1.015444	9.78E-06	2.159625	2.159625	1.110781	1.110781	2.91E-05	116	83	60	86.33333	89	77	97	87.66667

Feature ID	Experiment - Range (original values)	Experiment - IQR (original values)	Experiment - Difference (original values)	Experiment - Fold Change (original values)	EDGE test: WT H202 vs WT NT , tagwise dispersions - P-value	EDGE test: WT H202 vs WT NT , tagwise dispersions - Fold change	WT H202 vs WT NT ABS|FC|	WT H202 vs WT NT Log2FC	WT H202 vs WT NT Log2FC +-	EDGE test: WT H202 vs WT NT , tagwise dispersions - FDR p-value correction	WT NT - WT.1.S22 - Expression values	WT NT - WT.2.S23 - Expression values	WT NT - WT.3.S24 - Expression values	WT NT - Means	WT H202 - WT.1.H2O2 - Expression values	WT H202 - WT.2.H2O2 - Expression values	WT H202 - WT.3.H2O2 - Expression values	WT H202 - Means
lrhA	383	165	14.66667	1.015278	4.58E-05	2.137242	2.137242	1.09575	1.09575	0.000124	1165	782	933	960	1049	855	1020	974.6667
SEN3456	49	7	1.333333	1.01444	8.31E-06	2.167029	2.167029	1.115719	1.115719	2.51E-05	115	96	66	92.33333	89	96	96	93.66667
ssaN	45	17	1.333333	1.014035	8.39E-06	2.134262	2.134262	1.093738	1.093738	2.53E-05	101	110	74	95	119	84	86	96.33333
safB	26	12	0.666667	1.013605	5.61E-05	2.115562	2.115562	1.081041	1.081041	0.000149	55	49	43	49	60	34	55	49.66667
SEN0159	18	6	0.333333	1.012821	0.000641	2.155509	2.155509	1.108028	1.108028	0.001403	37	22	19	26	22	29	28	26.33333
yohF	71	28	1.666667	1.012594	2.48E-05	2.131751	2.131751	1.092039	1.092039	6.97E-05	141	142	114	132.3333	109	113	180	134
SEN1379	9	5	0.333333	1.011628	0.000138	2.126351	2.126351	1.08838	1.08838	0.000342	32	31	23	28.66667	32	26	29	29
SEN3717	18	5	0.333333	1.011111	0.00032	2.118174	2.118174	1.082821	1.082821	0.00074	33	31	26	30	27	23	41	30.33333
eutR	111	23	1.333333	1.010444	7.61E-05	2.178812	2.178812	1.123542	1.123542	0.000197	196	102	85	127.6667	115	125	147	129
SEN3888	28	10	1.666667	1.010309	1.91E-06	2.125934	2.125934	1.088097	1.088097	6.38E-06	165	170	150	161.6667	178	155	157	163.3333
lpxO	81	20	2	1.00936	6.63E-06	2.148213	2.148213	1.103137	1.103137	2.04E-05	261	200	180	213.6667	189	209	249	215.6667
crl	825	167	15.33333	1.008827	0.000795	2.144964	2.144964	1.100953	-1.10095	0.001711	1793	1792	1626	1737	1292	1848	2117	1752.333
thiF	31	9	0.333333	1.007463	0.001493	2.147192	2.147192	1.102451	1.102451	0.003029	49	40	45	44.66667	25	54	56	45
ruvA	124	2	2.333333	1.006228	5.31E-06	2.131816	2.131816	1.092083	1.092083	1.65E-05	447	354	323	374.6667	420	355	356	377
SEN1179	22	8	0.333333	1.00578	3.05E-05	2.099143	2.099143	1.0698	1.0698	8.41E-05	61	52	60	57.66667	60	46	68	58
SEN3519	39	16	0.333333	1.003155	1.67E-05	2.080829	2.080829	1.057158	1.057158	4.81E-05	105	98	114	105.6667	119	80	119	106
ftsJ	2900	51	6.333333	1.001366	0.00139	2.06942	2.06942	1.049226	-1.04923	0.002844	4789	4576	4541	4635.333	6150	3250	4525	4641.667
eutH	244	61	0	-1	0.007078	2.196645	2.196645	1.135302	1.135302	0.012545	313	78	69	153.3333	184	139	137	153.3333
nirC	178	76	0	-1	0.022979	2.158057	2.158057	1.109733	1.109733	0.0366	77	210	148	145	52	230	153	145
yjcH	28	6	0	-1	0.001566	2.126181	2.126181	1.088265	1.088265	0.003162	44	19	16	26.33333	25	21	33	26.33333
invG	35	15	0	-1	2.19E-05	2.114544	2.114544	1.080347	1.080347	6.19E-05	78	61	43	60.66667	65	50	67	60.66667
pudB	44	14	0	-1	0.013693	2.081306	2.081306	1.057489	1.057489	0.022873	56	15	38	36.33333	59	24	26	36.33333
stfA	12	7	0	-1	0.001904	2.078203	2.078203	1.055336	1.055336	0.003785	16	26	19	20.33333	24	14	23	20.33333
SEN3844	21	5	0	-1	0.000316	2.074798	2.074798	1.052971	1.052971	0.000732	35	40	35	36.66667	44	23	43	36.66667
bcr	113	41	-1	-1.00274	5.56E-06	2.107305	2.107305	1.075399	-1.0754	1.73E-05	429	351	316	365.3333	396	328	369	364.3333
tpx	2350	935	-9.66667	-1.00292	0.006037	2.144112	2.144112	1.100381	1.100381	0.010883	2666	4339	2959	3321.333	1992	4342	3601	3311.667
himA	1521	460	-19	-1.00359	0.000598	2.10869	2.10869	1.076347	1.076347	0.001315	6301	4780	4835	5305.333	5295	4895	5669	5286.333
hpaR	181	96	-1.33333	-1.00445	2.81E-05	2.097246	2.097246	1.068496	1.068496	7.8E-05	399	269	235	301	350	218	331	299.6667
ydhD	1524	313	-14.6667	-1.00469	0.001282	2.101222	2.101222	1.071229	1.071229	0.002636	2726	4108	2584	3139.333	2943	3039	3392	3124.667
kdsB	291	48	-9	-1.00828	0.00011	2.073829	2.073829	1.052297	1.052297	0.000276	1057	1181	1049	1095.667	1097	936	1227	1086.667
deoC	3584	951	-26	-1.00882	0.015318	2.059237	2.059237	1.04211	1.04211	0.025241	2136	5187	1603	2975.333	4009	1944	2895	2949.333
stdB	36	21	-1	-1.00901	1.05E-05	2.10536	2.10536	1.074067	1.074067	3.11E-05	125	122	89	112	120	114	99	111
ybdM	81	59	-2	-1.00939	2.25E-05	2.115133	2.115133	1.080748	1.080748	6.34E-05	245	231	169	215	172	217	250	213

Feature ID	Experiment - Range (original values)	Experiment - IQR (original values)	Experiment - Difference (original values)	Experiment - Fold Change (original values)	EDGE test: WT H202 vs WT NT , tagwise dispersions - P-value	EDGE test: WT H202 vs WT NT , tagwise dispersions - Fold change	WT H202 vs WT NT ABS|FC|	WT H202 vs WT NT Log2FC	WT H202 vs WT NT Log2FC +-	EDGE test: WT H202 vs WT NT , tagwise dispersions - FDR p-value correction	WT NT - WT.1.S22 - Expression values	WT NT - WT.2.S23 - Expression values	WT NT - WT.3.S24 - Expression values	WT NT - Means	WT H202 - WT.1.H2O2 - Expression values	WT H202 - WT.2.H2O2 - Expression values	WT H202 - WT.3.H2O2 - Expression values	WT H202 - Means
mug	257	26	-4	-1.00955	5E-05	2.06511	2.06511	1.046218	1.046218	0.000134	501	390	377	422.6667	555	298	403	418.6667
fimF	20	6	-0.33333	-1.0101	0.000205	2.102493	2.102493	1.072101	1.072101	0.000495	46	28	26	33.33333	29	34	36	33
ydiF	19	14	-0.33333	-1.01087	0.000668	2.071223	2.071223	1.050483	1.050483	0.00146	38	33	22	31	36	19	37	30.66667
creC	90	24	-3.33333	-1.01136	1.08E-05	2.067077	2.067077	1.047592	-1.04759	3.18E-05	292	326	272	296.6667	337	247	296	293.3333
stiB	19	0	-0.33333	-1.01149	0.000326	2.096063	2.096063	1.067682	1.067682	0.000753	37	33	18	29.33333	29	29	29	29
treR	141	72	-3.33333	-1.01209	2.18E-05	2.070534	2.070534	1.050003	1.050003	6.15E-05	319	295	223	279	348	207	272	275.6667
SEN2641	152	49	-2	-1.01261	0.001122	2.058664	2.058664	1.041708	1.041708	0.002337	227	140	115	160.6667	232	80	164	158.6667
SEN3832	53	14	-2	-1.01336	2.33E-05	2.062458	2.062458	1.044364	1.044364	6.54E-05	131	183	141	151.6667	145	130	174	149.6667
SEN0033	62	18	-2.33333	-1.01483	3.92E-06	2.085629	2.085629	1.060482	1.060482	1.24E-05	195	151	133	159.6667	158	140	174	157.3333
SEN3112	7	2	-0.33333	-1.01493	0.001	2.061641	2.061641	1.043793	1.043793	0.002106	22	26	20	22.66667	26	19	22	22.33333
yiiU	782	376	-66	-1.01533	0.001027	2.059824	2.059824	1.042521	1.042521	0.002155	4003	4736	4379	4372.667	4343	3954	4623	4306.667
ybdQ	5958	1129	-143.333	-1.0158	0.00131	2.112281	2.112281	1.078802	1.078802	0.002692	12807	7982	6849	9212.667	9111	8607	9490	9069.333
astD	20	9	-1.33333	-1.01606	4.41E-06	2.073998	2.073998	1.052414	1.052414	1.39E-05	95	83	75	84.33333	88	76	85	83
mig-3	44	17	-1.66667	-1.01712	9.31E-06	2.072844	2.072844	1.051611	1.051611	2.78E-05	124	93	80	99	115	80	97	97.33333
yaiW	73	36	-7.66667	-1.01753	1.1E-05	2.068722	2.068722	1.04874	1.04874	3.24E-05	471	450	414	445	439	400	473	437.3333
xapB	25	13	-0.66667	-1.01887	0.000371	2.071718	2.071718	1.050827	1.050827	0.000844	46	41	21	36	40	27	39	35.33333
bigA	199	30	-8.33333	-1.01987	2.92E-05	2.038138	2.038138	1.027252	1.027252	8.09E-05	432	449	402	427.6667	517	318	423	419.3333
yegU	95	50	-5	-1.02063	8.32E-06	2.070121	2.070121	1.049715	1.049715	2.52E-05	290	257	195	247.3333	265	207	255	242.3333
ydcX	224	101	-9.33333	-1.02133	5.64E-05	2.037014	2.037014	1.026456	1.026456	0.00015	483	476	382	447	520	296	497	437.6667
tctE	62	12	-5.66667	-1.02191	9.37E-06	2.073823	2.073823	1.052293	1.052293	2.8E-05	294	267	232	264.3333	256	254	266	258.6667
SEN0913	7	2	-0.33333	-1.02381	0.007793	2.047554	2.047554	1.033901	1.033901	0.013709	15	15	13	14.33333	17	15	10	14
SEN1000	16	5	-0.33333	-1.02381	0.030863	2.011378	2.011378	1.008184	1.008184	0.048035	6	15	22	14.33333	10	14	18	14
ompA	110740	35140	-3084.67	-1.02425	0.009647	2.129101	2.129101	1.090244	-1.09024	0.016602	135328	147970	107497	130265	64082	174822	142637	127180.3
SEN2742	9	1	-0.33333	-1.02857	0.013065	2.041221	2.041221	1.029432	1.029432	0.021987	17	9	10	12	8	10	17	11.66667
envR	22	8	-0.66667	-1.02899	0.005039	2.027746	2.027746	1.019877	-1.01988	0.009226	29	25	17	23.66667	36	14	19	23
SEN0035	46	11	-4	-1.02993	7.74E-06	2.045506	2.045506	1.032457	1.032457	2.36E-05	165	119	129	137.6667	150	120	131	133.6667
ynfD	134	26	-17	-1.03	7.94E-05	2.058675	2.058675	1.041716	1.041716	0.000205	623	568	560	583.6667	490	586	624	566.6667
stdC	12	7	-0.66667	-1.0339	0.003362	2.058936	2.058936	1.041899	1.041899	0.006372	24	24	13	20.33333	17	25	17	19.66667
yebB	33	20	-3	-1.03475	8.81E-05	2.006596	2.006596	1.00475	1.00475	0.000226	71	104	93	89.33333	96	73	90	86.33333
trpS2	124	61	-13	-1.03507	1.66E-05	2.044096	2.044096	1.031463	1.031463	4.78E-05	425	425	301	383.6667	394	333	385	370.6667
SEN1564A	43	1	-3.33333	-1.03788	1.98E-05	2.046318	2.046318	1.03303	1.03303	5.62E-05	117	83	74	91.33333	84	83	97	88
pspC	44	9	-2.66667	-1.0381	0.000103	2.064441	2.064441	1.045751	-1.04575	0.000261	98	66	54	72.66667	71	77	62	70
yihX	319	57	-45	-1.0382	0.000319	2.034936	2.034936	1.024983	1.024983	0.000739	1148	1420	1101	1223	1129	1186	1219	1178

Feature ID	Experiment - Range (original values)	Experiment - IQR (original values)	Experiment - Difference (original values)	Experiment - Fold Change (original values)	EDGE test: WT H202 vs WT NT , tagwise dispersions - P-value	EDGE test: WT H202 vs WT NT , tagwise dispersions - Fold change	WT H202 vs WT NT ABS|FC|	WT H202 vs WT NT Log2FC	WT H202 vs WT NT Log2FC +-	EDGE test: WT H202 vs WT NT , tagwise dispersions - FDR p-value correction	WT NT - WT.1.S22 - Expression values	WT NT - WT.2.S23 - Expression values	WT NT - WT.3.S24 - Expression values	WT NT - Means	WT H202 - WT.1.H2O2 - Expression values	WT H202 - WT.2.H2O2 - Expression values	WT H202 - WT.3.H2O2 - Expression values	WT H202 - Means
SEN0805	48	9	-5	-1.03979	1.36E-05	2.025229	2.025229	1.018085	1.018085	3.94E-05	153	124	115	130.6667	121	105	151	125.6667
ushB	515	59	-20	-1.03989	0.005656	2.101453	2.101453	1.071387	1.071387	0.010258	549	535	480	521.3333	225	740	539	501.3333
ydeA	82	4	-6.33333	-1.04086	5.51E-05	2.026859	2.026859	1.019246	1.019246	0.000146	212	134	138	161.3333	199	130	136	155
gsk	221	70	-25.6667	-1.04135	9.01E-05	2.043585	2.043585	1.031102	1.031102	0.000231	762	636	541	646.3333	566	601	695	620.6667
yceL	130	27	-13	-1.04158	2.66E-05	2.030288	2.030288	1.021684	1.021684	7.41E-05	402	303	272	325.6667	370	276	292	312.6667
yciS	499	130	-42	-1.04179	0.000213	2.003888	2.003888	1.002802	1.002802	0.000513	1175	1048	918	1047	1247	748	1020	1005
pfkB	1671	411	-141.667	-1.04202	0.001748	2.051024	2.051024	1.036345	1.036345	0.003505	4471	3240	2829	3513.333	2800	3208	4107	3371.667
sseE	6	3	-0.66667	-1.04255	0.005364	2.017352	2.017352	1.012463	1.012463	0.009765	17	18	14	16.33333	17	18	12	15.66667
yohC	38	12	-2	-1.04348	0.000487	2.031103	2.031103	1.022263	1.022263	0.001088	69	44	31	48	47	35	56	46
ybbP	110	18	-15	-1.04386	1.58E-05	2.014255	2.014255	1.010247	1.010247	4.55E-05	407	335	329	357	382	297	347	342
SEN1026	32	8	-5.66667	-1.04582	8.9E-06	2.002372	2.002372	1.00171	1.00171	2.67E-05	134	131	123	129.3333	138	106	127	123.6667
SEN0333	28	10	-2.33333	-1.04605	0.000384	2.029263	2.029263	1.020956	1.020956	0.000872	65	52	42	53	38	48	66	50.66667
SEN0581	14	5	-1.33333	-1.04762	0.002567	2.01661	2.01661	1.011932	1.011932	0.004965	25	33	30	29.33333	26	36	22	28
shdA	174	53	-32.3333	-1.04869	8.16E-05	2.019555	2.019555	1.014037	1.014037	0.00021	791	681	617	696.3333	638	628	726	664
ybbM	112	46	-18	-1.05085	2.86E-05	2.006953	2.006953	1.005007	1.005007	7.93E-05	420	371	325	372	341	308	413	354
yahO	216	39	-11.3333	-1.05097	0.000618	2.052361	2.052361	1.037284	1.037284	0.001355	375	159	167	233.6667	197	206	264	222.3333
sthB	54	18	-4.66667	-1.05512	5.84E-05	2.006746	2.006746	1.004858	1.004858	0.000155	117	88	63	89.33333	101	70	83	84.66667
ybeF	120	41	-9	-1.05649	0.000389	2.040347	2.040347	1.028814	1.028814	0.000883	234	150	121	168.3333	114	162	202	159.3333
SEN0393	128	35	-17.3333	-1.05862	0.000113	2.020509	2.020509	1.014719	1.014719	0.000283	367	283	289	313	239	330	318	295.6667
SEN1131	42	5	-5.66667	-1.06204	3.6E-05	2.003142	2.003142	1.002264	1.002264	9.83E-05	122	89	80	97	86	91	97	91.33333
ybeM	59	5	-7	-1.06688	0.000102	2.005062	2.005062	1.003647	1.003647	0.000259	149	90	96	111.6667	100	113	101	104.6667
