# Supplementary material for: Insights into the Oxidative Stress Response of Salmonella enterica serovar Enteritidis Revealed by the Next Generation Sequencing Approach
Source: Antioxidants (Basel). 2020 Sep 10;9(9):849. doi: 10.3390/antiox9090849 (PMC7555449; doi:10.3390/antiox9090849)
Supplement: Supplementary file 1 [file antioxidants-09-00849-s001.zip › antioxidants-897047-supplementary/Table S2.pdf]

Feature ID	Experiment - Range (original values)	Experiment - IQR (original values)	Experiment - Difference (original values)	Experiment - Fold Change (original values)	EDGE test: WT H202 vs WT NT , tagwise dispersions - P-value	EDGE test: WT H202 vs WT NT , tagwise dispersions - Fold change	WT H202 vs WT NT ABS|FC|	WT H202 vs WT NT Log2FC	WT H202 vs WT NT Log2FC +-	EDGE test: WT H202 vs WT NT , tagwise dispersions - FDR p-value correction	WT NT - WT.1.S22 - Expression values	WT NT - WT.2.S23 - Expression values	WT NT - WT.3.S24 - Expression values	WT NT - Means	WT H202 - WT.1.H2O2 - Expression values	WT H202 - WT.2.H2O2 - Expression values	WT H202 - WT.3.H2O2 - Expression values	WT H202 - Means
rseC	544	394	-452	-4.11724	0.000382	-2.00293	2.002933	1.002114	1.002114	0.000867	517	648	626	597	208	104	123	145
rfaQ	981	802	-845.667	-4.14374	0.000285	-2.00951	2.00951	1.006844	-1.00684	0.000665	1043	1175	1126	1114.667	372	194	241	269
celC	973	580	-701	-4.18154	0.001453	-2.05455	2.054552	1.038824	1.038824	0.002956	766	1096	902	921.3333	352	123	186	220.3333
caiA	355	264	-285.333	-4.18216	7.65E-05	-2.00408	2.004077	1.002938	-1.00294	0.000198	421	362	342	375	125	66	78	89.66667
SEN1792	558	95	-296	-4.1828	0.013544	-2.04138	2.04138	1.029545	1.029545	0.022681	182	346	639	389	87	111	81	93
yeaK	323	247	-272.667	-4.18288	0.000107	-2.00474	2.00474	1.003415	1.003415	0.000271	330	351	394	358.3333	103	83	71	85.66667
dcuA	17090	13579	-14478.7	-4.18492	0.002697	-2.01128	2.011284	1.008117	-1.00812	0.005195	20756	17339	18979	19024.67	6212	3666	3760	4546
proQ	2374	1858	-2037.33	-4.19665	0.000412	-2.02366	2.023664	1.016969	1.016969	0.000933	2474	2631	2919	2674.667	751	545	616	637.3333
nlpC	194	138	-158	-4.2027	2.85E-05	-2.00175	2.001753	1.001264	-1.00126	7.91E-05	235	188	199	207.3333	57	41	50	49.33333
mutT	75	44	-57.6667	-4.2037	0.002555	-2.02247	2.022474	1.016121	1.016121	0.004944	59	88	80	75.66667	15	13	26	18
yqiA	1470	956	-1199.67	-4.20481	0.000194	-2.01155	2.011551	1.008308	1.008308	0.000469	1641	1760	1321	1574	468	290	365	374.3333
yggT	472	412	-437	-4.20538	4.27E-05	-2.01209	2.012088	1.008694	1.008694	0.000116	544	577	599	573.3333	150	127	132	136.3333
araC	767	605	-665.333	-4.209	4.75E-05	-2.02801	2.028013	1.020067	1.020067	0.000128	878	916	824	872.6667	254	149	219	207.3333
SEN3732	327	236	-287	-4.21269	0.000112	-2.01124	2.011241	1.008086	1.008086	0.000281	326	398	405	376.3333	78	90	100	89.33333
SEN0993	281	159	-193	-4.21667	0.001852	-2.06548	2.065481	1.046478	1.046478	0.003695	206	313	240	253	101	32	47	60
yacF	695	555	-627.333	-4.21709	3.37E-05	-2.00267	2.002673	1.001927	1.001927	9.22E-05	842	875	750	822.3333	195	180	210	195
imp	4851	4421	-4589.67	-4.22233	0.001082	-2.02235	2.022355	1.016036	1.016036	0.002257	5926	6026	6090	6014	1505	1239	1529	1424.333
wecD	448	347	-385	-4.22626	4.7E-05	-2.0226	2.022601	1.016212	1.016212	0.000127	466	552	495	504.3333	119	104	135	119.3333
yigP	1859	1632	-1760.33	-4.228	0.000238	-2.01839	2.018392	1.013207	1.013207	0.000566	2176	2375	2366	2305.667	544	516	576	545.3333
glyA	10597	6459	-9026	-4.22818	0.002294	-2.03656	2.036561	1.026135	1.026135	0.00449	9314	12934	13218	11822	2912	2621	2855	2796
yfgM	1176	1006	-1094.67	-4.24186	0.000233	-2.00479	2.004794	1.003454	1.003454	0.000555	1475	1362	1460	1432.333	299	358	356	337.6667
SEN2218	551	524	-523	-4.25519	2.09E-05	-2.04086	2.040856	1.029174	1.029174	5.93E-05	685	683	683	683.6667	189	134	159	160.6667
cbiF	3189	812	-1823.33	-4.25595	0.005725	-2.10139	2.101385	1.071341	1.071341	0.010372	2226	1314	3610	2383.333	757	421	502	560
mreC	896	401	-651.667	-4.25833	0.00029	-2.07634	2.076343	1.054045	-1.05404	0.000676	617	875	1063	851.6667	216	167	217	200
allD	131	82	-96.6667	-4.25843	0.000201	-2.03723	2.037232	1.02661	-1.02661	0.000484	149	113	117	126.3333	40	18	31	29.66667
SEN3728	315	224	-249	-4.26201	8.51E-05	-2.0542	2.054197	1.038575	1.038575	0.000219	295	301	380	325.3333	71	65	93	76.33333
rhtC	407	278	-333.333	-4.26797	1.43E-05	-2.02445	2.024455	1.017534	-1.01753	4.13E-05	496	438	372	435.3333	123	89	94	102
yaiE	997	739	-846	-4.27484	4.73E-05	-2.016	2.015996	1.011493	1.011493	0.000127	1252	1065	996	1104.333	257	255	263	258.3333
rffG	1279	775	-1070	-4.27551	0.00026	-2.05925	2.059254	1.042122	1.042122	0.000613	1096	1575	1519	1396.667	321	296	363	326.6667
hemY	2682	1840	-2327.33	-4.28102	0.000742	-2.0496	2.049604	1.035345	1.035345	0.001608	2556	3366	3188	3036.667	716	684	728	709.3333
ptr	1604	1393	-1491.67	-4.28802	0.000243	-2.01658	2.016576	1.011908	1.011908	0.000577	1980	2007	1849	1945.333	403	502	456	453.6667
ydiY	182	95	-130.667	-4.29412	5.16E-05	-2.03371	2.033714	1.024117	1.024117	0.000138	212	163	136	170.3333	48	30	41	39.66667
SEN1201	592	455	-495	-4.3	2.38E-05	-2.046	2.045999	1.032805	1.032805	6.7E-05	723	625	587	645	187	131	132	150
hutI	1740	982	-1425.33	-4.30448	0.000108	-2.03188	2.031885	1.022818	-1.02282	0.000272	2121	2012	1437	1856.667	455	381	458	431.3333
araH	264	239	-242.333	-4.30455	1.23E-05	-2.05559	2.055592	1.039554	1.039554	3.58E-05	310	330	307	315.6667	86	66	68	73.33333
ppa	4788	2798	-4000	-4.3067	0.002455	-2.056	2.056005	1.039844	1.039844	0.004774	4010	5916	5703	5209.667	1128	1289	1212	1209.667
ychA	440	342	-380.333	-4.30725	2.49E-05	-2.07074	2.07074	1.050146	1.050146	6.99E-05	492	457	537	495.3333	133	97	115	115

Feature ID	Experiment - Range (original values)	Experiment - IQR (original values)	Experiment - Difference (original values)	Experiment - Fold Change (original values)	EDGE test: WT H202 vs WT NT , tagwise dispersions - P-value	EDGE test: WT H202 vs WT NT , tagwise dispersions - Fold change	WT H202 vs WT NT ABS|FC|	WT H202 vs WT NT Log2FC	WT H202 vs WT NT Log2FC +-	EDGE test: WT H202 vs WT NT , tagwise dispersions - FDR p-value correction	WT NT - WT.1.S22 - Expression values	WT NT - WT.2.S23 - Expression values	WT NT - WT.3.S24 - Expression values	WT NT - Means	WT H202 - WT.1.H2O2 - Expression values	WT H202 - WT.2.H2O2 - Expression values	WT H202 - WT.3.H2O2 - Expression values	WT H202 - Means
glgS	2592	722	-1284.67	-4.30815	0.011441	-2.04891	2.048915	1.03486	1.03486	0.019475	2786	1203	1030	1673	663	194	308	388.3333
SEN1441	252	124	-165.667	-4.31333	0.000174	-2.02623	2.026231	1.018799	1.018799	0.000425	292	185	170	215.6667	64	46	40	50
dapB	759	505	-579.333	-4.31679	3.99E-05	-2.0451	2.045103	1.032174	-1.03217	0.000108	910	692	660	754	218	155	151	174.6667
ybgJ	644	573	-585	-4.32386	0.000296	-2.03076	2.030758	1.022018	1.022018	0.000691	755	750	778	761	177	217	134	176
yfeZ	267	191	-224	-4.32673	0.000476	-2.03736	2.037358	1.026699	1.026699	0.001066	255	318	301	291.3333	64	87	51	67.33333
ptsA	2539	1695	-2134	-4.32917	0.000232	-2.04785	2.047848	1.034108	1.034108	0.000555	2863	3137	2325	2775	630	598	695	641
tas	2111	1590	-1878.33	-4.33432	0.000162	-2.04924	2.049243	1.035091	1.035091	0.000398	2640	2553	2132	2441.667	619	542	529	563.3333
yciC	958	746	-813.667	-4.3347	3.4E-05	-2.07603	2.076028	1.053826	1.053826	9.3E-05	1150	1054	969	1057.667	317	192	223	244
SEN4271	272	139	-182.333	-4.33537	7.78E-05	-2.03279	2.032792	1.023463	1.023463	0.000201	323	195	193	237	54	51	59	54.66667
ynfA	76	50	-55.6667	-4.34	0.000888	-2.05468	2.05468	1.038914	1.038914	0.001897	87	65	65	72.33333	24	15	11	16.66667
dacD	664	403	-510	-4.34061	1.89E-05	-2.06893	2.068933	1.048887	-1.04889	5.39E-05	779	649	560	662.6667	186	115	157	152.6667
yfcM	214	189	-196.333	-4.34659	5.25E-05	-2.05642	2.056419	1.040134	1.040134	0.00014	260	251	254	255	46	62	68	58.66667
SEN1754	367	151	-263.333	-4.34746	0.000321	-2.07157	2.071573	1.050727	1.050727	0.000743	432	221	373	342	70	65	101	78.66667
ybaX	461	327	-390.667	-4.34857	1.27E-05	-2.05456	2.054556	1.038827	1.038827	3.7E-05	572	504	446	507.3333	119	111	120	116.6667
SEN1755	374	163	-269.333	-4.36667	0.000274	-2.04755	2.047549	1.033898	1.033898	0.000643	445	242	361	349.3333	71	90	79	80
SEN2895	206	169	-173.333	-4.37662	4.47E-05	-2.10106	2.10106	1.071118	1.071118	0.000121	212	247	215	224.6667	70	41	43	51.33333
menB	5240	2016	-4067	-4.37884	0.004783	-2.10718	2.107178	1.075312	1.075312	0.008805	3187	6257	6368	5270.667	1171	1312	1128	1203.667
mukE	878	686	-769.333	-4.37921	0.000221	-2.06083	2.060831	1.043226	1.043226	0.000531	920	1058	1013	997	180	269	234	227.6667
alaS	4950	4143	-4314	-4.3853	0.002707	-2.06029	2.060287	1.042845	1.042845	0.005208	5422	5377	5966	5588.333	1234	1573	1016	1274.333
ada	499	221	-351.333	-4.38907	6.3E-05	-2.04839	2.048395	1.034494	-1.03449	0.000166	592	454	319	455	93	98	120	103.6667
tdcD	68830	26866	-40735.3	-4.39263	0.011734	-2.18768	2.187683	1.129404	1.129404	0.019936	46667	34852	76708	52742.33	20157	7878	7986	12007
wzzB	1837	1167	-1415	-4.39329	0.000264	-2.10648	2.106479	1.074833	1.074833	0.000622	2131	1848	1517	1832	607	294	350	417
lamB	348749	297054	-322256	-4.39437	0.00102	-2.08551	2.085512	1.060401	1.060401	0.002141	438535	418562	394486	417194.3	97432	89786	97597	94938.33
gyrA	4234	3275	-3791.33	-4.39624	0.00219	-2.07277	2.072768	1.051558	1.051558	0.0043	4285	5230	5208	4907.667	996	1343	1010	1116.333
prfB	1679	1339	-1467	-4.39846	0.000208	-2.07497	2.074973	1.053093	1.053093	0.0005	1782	2053	1861	1898.667	374	478	443	431.6667
ydhO	420	296	-339	-4.40134	0.000173	-2.14974	2.149743	1.104164	1.104164	0.000422	451	388	477	438.6667	150	57	92	99.66667
ppx	1429	1117	-1213.33	-4.40824	0.000147	-2.08752	2.087517	1.061788	1.061788	0.000363	1479	1730	1499	1569.333	301	362	405	356
rnd	243	177	-201.333	-4.41243	7.71E-05	-2.12434	2.124335	1.087011	1.087011	0.0002	267	226	288	260.3333	83	49	45	59
yjeJ	723	450	-555.333	-4.41393	8.49E-06	-2.08697	2.086974	1.061413	1.061413	2.56E-05	867	674	613	718	181	144	163	162.6667
flgN	9132	2780	-5727.67	-4.42155	0.004597	-2.10461	2.10461	1.073553	-1.07355	0.008477	10106	7712	4387	7401.667	2441	974	1607	1674
rfaK	1368	1103	-1228.33	-4.42791	5.3E-05	-2.12558	2.125577	1.087855	-1.08785	0.000141	1471	1674	1615	1586.667	368	306	401	358.3333
SEN2463	418	357	-375	-4.42988	1.34E-05	-2.10775	2.107748	1.075702	1.075702	3.88E-05	465	517	471	484.3333	121	108	99	109.3333
rfbP	2775	2045	-2446	-4.43379	0.000319	-2.13129	2.131292	1.091728	-1.09173	0.00074	2768	3399	3308	3158.333	723	624	790	712.3333
nlpB	5241	4838	-4908.67	-4.43664	0.001111	-2.09563	2.09563	1.067384	-1.06738	0.002314	6234	6218	6559	6337	1380	1587	1318	1428.333
gldA	10472	7879	-8853.67	-4.43743	0.000685	-2.09778	2.097777	1.068861	-1.06886	0.001493	12970	10449	10869	11429.33	2570	2498	2659	2575.667
aarF	5055	4038	-4499.33	-4.43986	0.000972	-2.11363	2.113635	1.079726	1.079726	0.002054	5367	5830	6225	5807.333	1170	1329	1425	1308
yheU	296	239	-257	-4.44196	1.34E-05	-2.13192	2.131924	1.092156	1.092156	3.91E-05	338	352	305	331.6667	102	56	66	74.66667

Feature ID	Experiment - Range (original values)	Experiment - IQR (original values)	Experiment - Difference (original values)	Experiment - Fold Change (original values)	EDGE test: WT H202 vs WT NT , tagwise dispersions - P-value	EDGE test: WT H202 vs WT NT , tagwise dispersions - Fold change	WT H202 vs WT NT ABS|FC|	WT H202 vs WT NT Log2FC	WT H202 vs WT NT Log2FC +-	EDGE test: WT H202 vs WT NT , tagwise dispersions - FDR p-value correction	WT NT - WT.1.S22 - Expression values	WT NT - WT.2.S23 - Expression values	WT NT - WT.3.S24 - Expression values	WT NT - Means	WT H202 - WT.1.H2O2 - Expression values	WT H202 - WT.2.H2O2 - Expression values	WT H202 - WT.3.H2O2 - Expression values	WT H202 - Means
ogt	593	332	-443.667	-4.44819	1.08E-05	-2.11308	2.113075	1.079344	1.079344	3.19E-05	692	562	463	572.3333	156	99	131	128.6667
SEN1134	381	254	-306	-4.45113	1.74E-05	-2.13135	2.131349	1.091767	1.091767	4.99E-05	443	406	335	394.6667	123	62	81	88.66667
nuoC	7847	4660	-5978.33	-4.46436	0.000909	-2.15115	2.151152	1.105109	1.105109	0.001938	6286	9317	7509	7704	2081	1470	1626	1725.667
yciB	616	556	-567.667	-4.46843	8.78E-06	-2.15619	2.15619	1.108484	1.108484	2.64E-05	727	731	736	731.3333	200	120	171	163.6667
serB	413	318	-366.667	-4.47003	1.19E-05	-2.12744	2.127442	1.08912	1.08912	3.48E-05	511	416	490	472.3333	98	98	121	105.6667
orf70	331	282	-310	-4.47015	1.01E-05	-2.10841	2.10841	1.076156	1.076156	3E-05	414	413	371	399.3333	83	96	89	89.33333
hilC	302	61	-148.667	-4.48438	0.003304	-2.11161	2.11161	1.078343	1.078343	0.006273	327	108	139	191.3333	56	25	47	42.66667
rfbB	1799	1523	-1580.33	-4.48603	0.000272	-2.18697	2.18697	1.128933	1.128933	0.000638	1890	2108	2103	2033.667	684	309	367	453.3333
rfaY	665	454	-545	-4.48614	1.27E-05	-2.12647	2.126474	1.088463	-1.08846	3.71E-05	696	809	599	701.3333	145	144	180	156.3333
speC	499	313	-419.667	-4.48753	6.56E-06	-2.13289	2.132886	1.092807	1.092807	2.02E-05	584	596	440	540	127	97	137	120.3333
fdnG	1918	1438	-1517.33	-4.48812	0.005119	-2.07193	2.071927	1.050973	-1.05097	0.009354	1865	1802	2190	1952.333	272	669	364	435
yehT	240	159	-202.667	-4.49425	1.27E-05	-2.15655	2.15655	1.108725	1.108725	3.71E-05	220	292	270	260.6667	61	52	61	58
prkB	1594	1073	-1338.67	-4.50436	3.53E-05	-2.15992	2.159922	1.110979	-1.11098	9.65E-05	1785	1876	1501	1720.667	436	282	428	382
gcpE	4006	2719	-3164	-4.50776	0.000265	-2.13662	2.136624	1.095333	-1.09533	0.000623	4802	3782	3614	4066	1015	796	895	902
yrbF	530	414	-465.667	-4.51005	3.98E-06	-2.13805	2.138053	1.096298	1.096298	1.26E-05	654	602	539	598.3333	149	124	125	132.6667
yieE	511	332	-421.333	-4.51111	3.92E-06	-2.1328	2.132799	1.092748	1.092748	1.24E-05	619	549	456	541.3333	124	108	128	120
SEN4193	260	180	-220.667	-4.52128	4.69E-06	-2.14669	2.146686	1.102111	1.102111	1.47E-05	297	313	240	283.3333	60	53	75	62.66667
fabG	5260	4304	-4669.33	-4.52846	0.001007	-2.12645	2.126447	1.088445	1.088445	0.002118	6305	5916	5757	5992.667	1045	1453	1472	1323.333
yceF	482	349	-407.333	-4.53179	9.07E-06	-2.14144	2.141436	1.098579	1.098579	2.72E-05	595	465	508	522.6667	113	116	117	115.3333
cspD	7691	3890	-5396.67	-4.53571	0.002307	-2.1994	2.199399	1.137109	-1.13711	0.004512	8636	5150	6983	6923	2374	945	1260	1526.333
SEN1140	1409	626	-891.333	-4.53704	6.04E-05	-2.14097	2.140966	1.098262	1.098262	0.000159	1608	896	926	1143.333	287	199	270	252
yicM	253	185	-210	-4.53933	3.59E-06	-2.17727	2.177269	1.122519	1.122519	1.14E-05	261	297	250	269.3333	69	44	65	59.33333
mdtI	335	289	-295.333	-4.544	1.71E-05	-2.17841	2.178411	1.123276	1.123276	4.92E-05	371	365	400	378.6667	109	76	65	83.33333
ycfN	522	423	-462	-4.54476	8E-06	-2.15972	2.159723	1.110847	1.110847	2.43E-05	554	650	573	592.3333	132	131	128	130.3333
mutY	576	475	-503.667	-4.54695	7.03E-06	-2.17908	2.179078	1.123718	1.123718	2.16E-05	637	687	613	645.6667	138	111	177	142
cysG	1337	1202	-1216	-4.54864	0.000737	-2.11354	2.113543	1.079663	1.079663	0.001597	1586	1524	1566	1558.667	249	457	322	342.6667
yehS	514	369	-428.667	-4.55249	4.96E-05	-2.20325	2.203245	1.13963	1.13963	0.000133	578	585	485	549.3333	175	71	116	120.6667
yfhL	283	158	-206.333	-4.55747	0.000471	-2.09366	2.093664	1.06603	1.06603	0.001056	329	259	205	264.3333	47	81	46	58
yfjD	690	526	-607.667	-4.56055	1.16E-05	-2.15015	2.150145	1.104434	1.104434	3.39E-05	834	795	706	778.3333	188	180	144	170.6667
syd	643	360	-483.667	-4.56511	1.13E-05	-2.16512	2.165119	1.114446	1.114446	3.31E-05	751	624	483	619.3333	176	108	123	135.6667
add	2038	1043	-1481	-4.57154	6.42E-05	-2.13972	2.139717	1.09742	1.09742	0.000168	2407	1832	1448	1895.667	470	405	369	414.6667
SEN1999	806	342	-521	-4.57666	8.92E-05	-2.16994	2.169936	1.117653	1.117653	0.000229	907	621	472	666.6667	206	101	130	145.6667
uraA	300	115	-198	-4.57831	0.000413	-2.22539	2.225386	1.154056	1.154056	0.000934	170	236	354	253.3333	54	55	57	55.33333
srfB	1251	975	-1077.67	-4.58029	3.98E-05	-2.16883	2.168825	1.116914	1.116914	0.000108	1284	1531	1321	1378.667	280	314	309	301
torR	892	572	-731.667	-4.59247	3.33E-06	-2.18122	2.181224	1.125138	1.125138	1.07E-05	1061	966	779	935.3333	235	169	207	203.6667
fsr	620	279	-386.333	-4.59938	5.19E-05	-2.17081	2.170811	1.118234	-1.11823	0.000139	702	400	379	493.6667	140	82	100	107.3333
pheS	1390	1217	-1275.33	-4.60603	1.87E-05	-2.21682	2.216825	1.148495	1.148495	5.34E-05	1601	1615	1671	1629	384	281	396	353.6667

Feature ID	Experiment - Range (original values)	Experiment - IQR (original values)	Experiment - Difference (original values)	Experiment - Fold Change (original values)	EDGE test: WT H202 vs WT NT , tagwise dispersions - P-value	EDGE test: WT H202 vs WT NT , tagwise dispersions - Fold change	WT H202 vs WT NT ABS|FC|	WT H202 vs WT NT Log2FC	WT H202 vs WT NT Log2FC +-	EDGE test: WT H202 vs WT NT , tagwise dispersions - FDR p-value correction	WT NT - WT.1.S22 - Expression values	WT NT - WT.2.S23 - Expression values	WT NT - WT.3.S24 - Expression values	WT NT - Means	WT H202 - WT.1.H2O2 - Expression values	WT H202 - WT.2.H2O2 - Expression values	WT H202 - WT.3.H2O2 - Expression values	WT H202 - Means
ushA	2582	2078	-2343	-4.60646	0.000232	-2.17231	2.17231	1.11923	1.11923	0.000555	3131	2774	3073	2992.667	549	704	696	649.6667
manA	1434	1006	-1221	-4.60887	6.91E-05	-2.19036	2.190362	1.13117	-1.13117	0.00018	1351	1744	1583	1559.333	345	360	310	338.3333
pepD	7840	7118	-7228.67	-4.61193	0.00034	-2.21687	2.216868	1.148523	1.148523	0.000782	8954	9172	9564	9230	2444	1724	1836	2001.333
ybiH	294	162	-231.333	-4.61458	9.77E-06	-2.20043	2.200431	1.137786	1.137786	2.9E-05	323	339	224	295.3333	85	45	62	64
torT	690	523	-598.667	-4.62097	3.05E-06	-2.19005	2.190047	1.130962	1.130962	9.81E-06	840	775	677	764	192	154	150	165.3333
flhA	479	369	-399.667	-4.62236	5.62E-06	-2.20461	2.204612	1.140525	1.140525	1.74E-05	472	581	477	510	126	103	102	110.3333
smpB	1877	1419	-1460	-4.62583	0.001863	-2.26477	2.264767	1.179363	1.179363	0.003713	2076	1818	1694	1862.667	734	199	275	402.6667
nuoI	2694	1272	-1818.67	-4.63733	0.000497	-2.17977	2.179773	1.124178	1.124178	0.001111	2116	3062	1778	2318.667	368	506	626	500
ycfL	639	570	-582	-4.6375	4.44E-06	-2.22449	2.224486	1.153472	1.153472	1.39E-05	713	777	736	742	199	138	143	160
ybiS	810	508	-633	-4.63793	4.08E-06	-2.17944	2.17944	1.123957	1.123957	1.29E-05	978	767	676	807	168	168	186	174
slyD	7948	5875	-7062.33	-4.6479	0.000498	-2.21748	2.217483	1.148923	1.148923	0.001111	7857	9351	9787	8998.333	1839	1982	1987	1936
flhE	123	78	-93.6667	-4.64935	0.000224	-2.18243	2.182433	1.125938	1.125938	0.000537	113	143	102	119.3333	24	33	20	25.66667
hslJ	819	699	-746.333	-4.65253	2.71E-06	-2.23413	2.234127	1.159711	1.159711	8.77E-06	944	919	989	950.6667	220	170	223	204.3333
rimK	374	320	-346.667	-4.66197	2.3E-06	-2.21846	2.218461	1.149559	1.149559	7.55E-06	459	413	452	441.3333	106	93	85	94.66667
yiaE	3687	2908	-3183.33	-4.66603	0.00016	-2.20577	2.205772	1.141284	1.141284	0.000392	4511	3908	3736	4051.667	824	828	953	868.3333
dgkA	1013	638	-753	-4.66721	5.13E-05	-2.25282	2.252815	1.171729	1.171729	0.000137	1134	913	828	958.3333	305	121	190	205.3333
talC	1628	1130	-1370.33	-4.66726	8.79E-05	-2.21325	2.213247	1.146164	1.146164	0.000226	1527	1939	1766	1744	311	397	413	373.6667
pgtA	723	568	-609.333	-4.67807	2.35E-06	-2.2429	2.242902	1.165366	1.165366	7.72E-06	733	861	731	775	196	138	163	165.6667
ompN	333	119	-191.333	-4.67949	0.000666	-2.14081	2.140815	1.09816	1.09816	0.001457	375	186	169	243.3333	42	64	50	52
rdgC	929	683	-783.667	-4.69074	2.19E-06	-2.22925	2.229249	1.156558	1.156558	7.23E-06	1122	899	967	996	228	193	216	212.3333
yaeL	4668	3139	-3910.33	-4.69248	0.000251	-2.27032	2.27032	1.182896	1.182896	0.000594	4260	5094	5554	4969.333	1170	886	1121	1059
nuoN	6292	2996	-4524	-4.69407	0.001293	-2.18963	2.189632	1.130689	1.130689	0.002659	5698	7208	4340	5748.667	916	1344	1414	1224.667
accD	2381	1784	-2029	-4.69806	7.55E-05	-2.19972	2.199725	1.137323	1.137323	0.000196	2879	2527	2327	2577.667	498	605	543	548.6667
rna-AM93	35	21	-24.6667	-4.7	0.03147	-2.2537	2.253696	1.172293	-1.17229	0.048893	36	35	23	31.33333	17	2	1	6.666667
yggS	513	441	-470	-4.70079	1.56E-06	-2.25477	2.254766	1.172978	1.172978	5.31E-06	597	575	619	597	134	106	141	127
panD	747	580	-624.333	-4.70158	2.06E-06	-2.23851	2.23851	1.162539	1.162539	6.84E-06	893	753	733	793	207	146	153	168.6667
SEN4121	417	214	-312.333	-4.70356	4.73E-06	-2.24332	2.243318	1.165634	1.165634	1.48E-05	411	479	300	396.6667	105	62	86	84.33333
SEN2979	5826	2314	-3506	-4.70483	0.000909	-2.2632	2.263201	1.178365	1.178365	0.001938	3501	6527	3329	4452.333	1123	701	1015	946.3333
rplM	7269	5061	-6317	-4.70571	0.000776	-2.23012	2.230117	1.157119	1.157119	0.001674	6872	8682	8511	8021.667	1413	1890	1811	1704.667
rpsB	26913	7946	-15863.3	-4.71159	0.004501	-2.22757	2.227571	1.155471	1.155471	0.008322	12085	30629	17698	20137.33	3716	4967	4139	4274
amyA	1132	479	-843.667	-4.72206	2.16E-05	-2.22171	2.221707	1.151668	-1.15167	6.11E-05	1321	1195	695	1070.333	275	189	216	226.6667
ybjN	383	320	-333.333	-4.73134	2.51E-06	-2.28437	2.284374	1.191799	1.191799	8.19E-06	411	406	451	422.6667	114	68	86	89.33333
tdcC	129545	52851	-74544.3	-4.73693	0.01545	-2.4066	2.406598	1.266995	1.266995	0.02543	62867	81120	139490	94492.33	39883	9945	10016	19948
flgM	6088	1890	-3794	-4.74162	0.00172	-2.25645	2.256453	1.174057	1.174057	0.003451	6672	4891	2861	4808	1487	584	971	1014
yjeE	699	488	-561.333	-4.74222	2.48E-06	-2.23166	2.231663	1.158119	1.158119	8.09E-06	839	654	641	711.3333	157	153	140	150
tldD	3092	2362	-2732	-4.74247	5.92E-05	-2.25458	2.254575	1.172856	1.172856	0.000156	3736	3542	3108	3462	800	644	746	730
pagP	472	223	-315.667	-4.74308	1.82E-05	-2.21328	2.213276	1.146183	1.146183	5.2E-05	546	312	342	400	89	90	74	84.33333

Feature ID	Experiment - Range (original values)	Experiment - IQR (original values)	Experiment - Difference (original values)	Experiment - Fold Change (original values)	EDGE test: WT H202 vs WT NT , tagwise dispersions - P-value	EDGE test: WT H202 vs WT NT , tagwise dispersions - Fold change	WT H202 vs WT NT ABS|FC|	WT H202 vs WT NT Log2FC	WT H202 vs WT NT Log2FC +-	EDGE test: WT H202 vs WT NT , tagwise dispersions - FDR p-value correction	WT NT - WT.1.S22 - Expression values	WT NT - WT.2.S23 - Expression values	WT NT - WT.3.S24 - Expression values	WT NT - Means	WT H202 - WT.1.H2O2 - Expression values	WT H202 - WT.2.H2O2 - Expression values	WT H202 - WT.3.H2O2 - Expression values	WT H202 - Means
rna-AM93	194	123	-156	-4.744	4.95E-06	-2.24849	2.248487	1.168955	1.168955	1.55E-05	231	164	198	197.6667	47	41	37	41.66667
fkpA	11816	5115	-8385.67	-4.74527	0.001194	-2.33519	2.33519	1.22354	-1.22354	0.002474	7126	11185	13563	10624.67	2959	1747	2011	2239
rpe	1362	1091	-1203.33	-4.7526	3E-05	-2.23402	2.23402	1.159642	1.159642	8.31E-05	1514	1640	1418	1524	278	357	327	320.6667
yicH	2352	1096	-1524.33	-4.76069	7.77E-05	-2.21254	2.21254	1.145704	1.145704	0.000201	2719	1569	1501	1929.667	367	405	444	405.3333
fumA	12287	5844	-8490	-4.76385	0.000536	-2.31265	2.312651	1.209548	-1.20955	0.001189	8330	13895	10012	10745.67	2673	1608	2486	2255.667
pduF	203	105	-133.333	-4.77358	0.000358	-2.30379	2.303786	1.204007	-1.20401	0.000819	218	138	150	168.6667	58	15	33	35.33333
rna-AM93	76	48	-56.6667	-4.77778	7.47E-05	-2.26388	2.263876	1.178795	1.178795	0.000194	87	64	64	71.66667	18	11	16	15
rna-AM93	232	151	-183	-4.78621	9.36E-06	-2.30965	2.309646	1.207671	1.207671	2.8E-05	229	259	206	231.3333	63	27	55	48.33333
cutC	1191	659	-855	-4.78877	0.005052	-2.40342	2.403422	1.26509	-1.26509	0.009246	788	1282	1172	1080.667	457	91	129	225.6667
ppk	3027	2504	-2698	-4.79288	6.68E-05	-2.2783	2.278299	1.187957	1.187957	0.000175	3334	3696	3198	3409.333	694	669	771	711.3333
celD	1380	805	-1062.33	-4.79405	0.000237	-2.34447	2.344467	1.22926	1.22926	0.000566	1011	1584	1432	1342.333	430	206	204	280
murI	1531	1239	-1348.67	-4.80263	1.19E-05	-2.31675	2.316745	1.2121	-1.2121	3.5E-05	1779	1734	1597	1703.333	458	248	358	354.6667
parC	1466	1286	-1376.67	-4.81701	9.01E-06	-2.28665	2.286646	1.193233	-1.19323	2.7E-05	1809	1646	1757	1737.333	379	360	343	360.6667
gtrA	18	12	-14	-4.81818	0.020799	-2.26404	2.264039	1.178899	-1.1789	0.033476	20	19	14	17.66667	7	2	2	3.666667
lig	1905	1360	-1571.67	-4.82401	2.49E-05	-2.25261	2.252607	1.171595	1.171595	6.98E-05	2278	1913	1757	1982.667	373	463	397	411
dam	847	571	-737.667	-4.82872	7.88E-06	-2.30712	2.307123	1.206095	-1.20609	2.4E-05	767	1031	993	930.3333	196	198	184	192.6667
yaoF	440	190	-291.667	-4.83772	7.92E-06	-2.25553	2.255534	1.173469	1.173469	2.41E-05	510	329	264	367.6667	84	74	70	76
yfeK	203	143	-169	-4.84091	3.62E-06	-2.33935	2.339352	1.226109	1.226109	1.15E-05	189	214	236	213	53	33	46	44
SEN3968	207	131	-162.667	-4.84252	4.93E-06	-2.26655	2.266545	1.180495	1.180495	1.54E-05	245	198	172	205	38	48	41	42.33333
yihI	491	385	-431.667	-4.84273	1.45E-06	-2.33363	2.333631	1.222577	1.222577	4.97E-06	559	495	578	544	140	87	110	112.3333
SEN0905	301	135	-196	-4.84314	1.03E-05	-2.29946	2.299457	1.201293	1.201293	3.06E-05	333	213	195	247	61	32	60	51
orn	820	744	-733.333	-4.84615	1.6E-05	-2.34854	2.348537	1.231762	1.231762	4.62E-05	892	917	963	924	281	143	148	190.6667
ygjO	826	553	-655.333	-4.84736	1.06E-06	-2.32282	2.32282	1.215877	1.215877	3.7E-06	776	962	739	825.6667	189	136	186	170.3333
tyrS	4138	3010	-3579.33	-4.84737	0.000855	-2.2789	2.278899	1.188337	1.188337	0.001832	3834	4784	4911	4509.667	824	1194	773	930.3333
citE2	116	52	-75.6667	-4.84746	0.000224	-2.28509	2.285089	1.192251	-1.19225	0.000537	130	70	86	95.33333	27	18	14	19.66667
yidC	2301	1632	-2008.33	-4.8523	0.000116	-2.29688	2.29688	1.199675	1.199675	0.00029	2156	2746	2687	2529.667	445	595	524	521.3333
upp	1208	744	-944.333	-4.85442	5.43E-05	-2.33847	2.338472	1.225566	1.225566	0.000145	968	1199	1401	1189.333	193	224	318	245
rfbS	2308	1446	-1791.33	-4.86063	6.06E-05	-2.30982	2.309824	1.207783	1.207783	0.00016	1910	2688	2168	2255.333	380	464	548	464
SEN1355	839	621	-740	-4.8676	1.19E-06	-2.29998	2.299978	1.20162	1.20162	4.11E-06	954	1029	811	931.3333	194	190	190	191.3333
ychN	493	362	-407.667	-4.87025	3.84E-07	-2.30667	2.306668	1.20581	1.20581	1.44E-06	590	483	466	513	115	97	104	105.3333
prc	3778	2682	-3175.67	-4.87276	0.000114	-2.34397	2.343969	1.228954	-1.22895	0.000285	3480	3958	4549	3995.667	891	771	798	820
srmB	1966	1535	-1685.33	-4.87433	3.79E-06	-2.32361	2.32361	1.216368	1.216368	1.21E-05	2337	2071	1953	2120.333	516	371	418	435
yieN	2577	2094	-2327.67	-4.88376	1.28E-05	-2.32549	2.325486	1.217532	1.217532	3.71E-05	3100	2967	2714	2927	620	523	655	599.3333
aroK	3130	2278	-2762.67	-4.88743	0.000146	-2.32548	2.325477	1.217526	-1.21753	0.000361	3009	3638	3773	3473.333	643	758	731	710.6667
yjgK	644	516	-544.333	-4.8881	1.99E-06	-2.3614	2.3614	1.239643	1.239643	6.64E-06	649	646	758	684.3333	176	114	130	140
yihG	299	233	-256.667	-4.88889	9.54E-07	-2.33147	2.331468	1.221239	1.221239	3.35E-06	323	354	291	322.6667	85	58	55	66
rpsR	4509	1427	-2984.67	-4.89643	0.005818	-2.20146	2.201459	1.13846	1.13846	0.010513	4943	4114	2195	3750.667	434	1096	768	766

Feature ID	Experiment - Range (original values)	Experiment - IQR (original values)	Experiment - Difference (original values)	Experiment - Fold Change (original values)	EDGE test: WT H202 vs WT NT , tagwise dispersions - P-value	EDGE test: WT H202 vs WT NT , tagwise dispersions - Fold change	WT H202 vs WT NT ABS|FC|	WT H202 vs WT NT Log2FC	WT H202 vs WT NT Log2FC +-	EDGE test: WT H202 vs WT NT , tagwise dispersions - FDR p-value correction	WT NT - WT.1.S22 - Expression values	WT NT - WT.2.S23 - Expression values	WT NT - WT.3.S24 - Expression values	WT NT - Means	WT H202 - WT.1.H2O2 - Expression values	WT H202 - WT.2.H2O2 - Expression values	WT H202 - WT.3.H2O2 - Expression values	WT H202 - Means
rfbM	3869	2964	-3454.33	-4.90909	9.46E-05	-2.36108	2.361082	1.239448	-1.23945	0.000241	3824	4551	4639	4338	860	770	1021	883.6667
cypD	3301	2994	-3003	-4.92549	6.45E-05	-2.35903	2.359031	1.238194	1.238194	0.000169	3952	3699	3653	3768	985	651	659	765
yaeJ	507	346	-412.333	-4.93949	4.93E-07	-2.36616	2.366157	1.242546	1.242546	1.83E-06	580	510	461	517	126	73	115	104.6667
phoU	600	554	-570	-4.94009	3.51E-07	-2.35666	2.35666	1.236744	-1.23674	1.33E-06	702	733	709	714.6667	148	133	153	144.6667
ftsY	2059	1899	-1958	-4.94228	1.07E-05	-2.35221	2.352215	1.23402	-1.23402	3.16E-05	2392	2502	2470	2454.667	554	493	443	496.6667
yaeT	12000	10154	-10753	-4.95186	0.000147	-2.35964	2.359637	1.238565	1.238565	0.000363	12871	12985	14566	13474	2566	2717	2880	2721
rplY	4166	1585	-2949.33	-4.95707	0.002403	-2.33711	2.337108	1.224725	1.224725	0.004683	2335	4672	4077	3694.667	506	980	750	745.3333
yqgB	117	106	-108.333	-4.96341	3.07E-06	-2.36856	2.368563	1.244012	1.244012	9.89E-06	140	130	137	135.6667	35	24	23	27.33333
hupA	9480	6674	-7665	-4.96739	0.000509	-2.3659	2.365895	1.242386	1.242386	0.001136	8661	9252	10878	9597	1398	1987	2411	1932
ybeL	5170	2781	-4082.67	-4.97533	9.67E-05	-2.37422	2.374216	1.247451	1.247451	0.000246	5898	5529	3902	5109.667	1232	728	1121	1027
lldR	234	84	-134.333	-4.9901	0.000206	-2.37732	2.377315	1.249333	-1.24933	0.000497	124	262	118	168	34	28	39	33.66667
atpA	24075	11142	-18317.3	-4.9907	0.000618	-2.41209	2.412095	1.270287	1.270287	0.001355	15778	24775	28169	22907.33	5040	4636	4094	4590
ampG	396	310	-350.333	-4.9962	2.95E-07	-2.39546	2.395464	1.260305	-1.26031	1.13E-06	468	393	453	438	108	72	83	87.66667
SEN4214	32424	5731	-18143	-4.99949	0.016239	-2.44742	2.44742	1.291262	1.291262	0.026611	34534	8099	25405	22679.33	8615	2110	2884	4536.333
dnaN	1766	1438	-1605.33	-5.00333	3.9E-06	-2.39528	2.395278	1.260193	-1.26019	1.24E-05	1847	2029	2143	2006.333	409	377	417	401
fabI	4902	3354	-4129	-5.01524	0.00013	-2.40516	2.405156	1.26613	1.26613	0.000323	4382	5239	5851	5157.333	1108	1028	949	1028.333
yhfG	90	43	-60.3333	-5.02222	5.55E-05	-2.35887	2.35887	1.238096	1.238096	0.000147	103	64	59	75.33333	16	13	16	15
SEN2863	241	154	-200	-5.02685	2.43E-07	-2.39306	2.393062	1.258858	1.258858	9.44E-07	277	264	208	249.6667	54	36	59	49.66667
glgA	4431	2372	-3030.33	-5.02791	6.76E-05	-2.38551	2.385511	1.254298	-1.2543	0.000177	5010	3216	3122	3782.667	928	579	750	752.3333
pgm	6123	5455	-5703.67	-5.02801	6.83E-05	-2.38732	2.387316	1.255389	-1.25539	0.000178	7020	7488	6851	7119.667	1487	1396	1365	1416
aroH	1032	505	-721.333	-5.0298	1.18E-06	-2.36032	2.360325	1.238985	1.238985	4.11E-06	1190	824	687	900.3333	182	158	197	179
SEN3894	566	380	-449	-5.03293	2.67E-06	-2.43958	2.439581	1.286633	1.286633	8.67E-06	485	645	551	560.3333	150	79	105	111.3333
yiaC	1475	1330	-1359.67	-5.03861	3.27E-06	-2.40829	2.408294	1.268012	1.268012	1.05E-05	1654	1651	1784	1696.333	321	309	380	336.6667
glpK	105674	71702	-83323.3	-5.04057	0.001639	-2.47687	2.476875	1.308521	1.308521	0.003304	107051	114641	90143	103945	34457	8967	18441	20621.67
bglX	997	859	-905.667	-5.05522	5.55E-07	-2.39585	2.395848	1.260536	-1.26054	2.05E-06	1212	1076	1099	1129	215	217	238	223.3333
SEN2864	597	135	-319.667	-5.06356	0.000353	-2.33207	2.332066	1.221609	1.221609	0.00081	663	328	204	398.3333	69	66	101	78.66667
rna-AM93	150	114	-133	-5.07143	3.93E-07	-2.40821	2.408211	1.267962	1.267962	1.47E-06	173	178	146	165.6667	32	28	38	32.66667
emrA	1285	1129	-1167	-5.07567	8.67E-07	-2.43217	2.432166	1.282242	1.282242	3.07E-06	1520	1428	1412	1453.333	341	235	283	286.3333
gdhA	2186	1121	-1515.67	-5.07803	1.93E-05	-2.35758	2.357576	1.237304	1.237304	5.51E-05	2496	1652	1514	1887.333	393	412	310	371.6667
SEN3093	328	235	-293.667	-5.0787	1.83E-07	-2.39257	2.392569	1.25856	1.25856	7.26E-07	392	398	307	365.6667	72	74	70	72
SEN2427	4199	4014	-4049	-5.07891	9.42E-05	-2.39509	2.39509	1.26008	1.26008	0.00024	4970	5121	5034	5041.667	922	1100	956	992.6667
exo	429	283	-325	-5.0795	1.85E-07	-2.41757	2.41757	1.273557	1.273557	7.3E-07	491	364	359	404.6667	101	62	76	79.66667
uvrY	6513	3813	-4743.67	-5.08116	7.2E-05	-2.42476	2.424761	1.277843	1.277843	0.000187	7357	5302	5059	5906	1397	844	1246	1162.333
SEN2782	712	553	-638.333	-5.10064	5.84E-07	-2.43349	2.433488	1.283026	1.283026	2.14E-06	706	862	814	794	150	153	164	155.6667
mdoB	2735	1404	-1824.33	-5.11195	9.96E-06	-2.42272	2.422722	1.276629	1.276629	2.96E-05	3074	1891	1839	2268	557	339	435	443.6667
ycfM	1073	817	-954.667	-5.12086	1.51E-06	-2.4542	2.454205	1.295256	1.295256	5.14E-06	1037	1283	1239	1186.333	265	220	210	231.6667
SEN1253	36	30	-31.6667	-5.13043	0.000559	-2.42584	2.42584	1.278485	1.278485	0.001237	42	39	37	39.33333	10	7	6	7.666667

Feature ID	Experiment - Range (original values)	Experiment - IQR (original values)	Experiment - Difference (original values)	Experiment - Fold Change (original values)	EDGE test: WT H202 vs WT NT , tagwise dispersions - P-value	EDGE test: WT H202 vs WT NT , tagwise dispersions - Fold change	WT H202 vs WT NT ABS|FC|	WT H202 vs WT NT Log2FC	WT H202 vs WT NT Log2FC +-	EDGE test: WT H202 vs WT NT , tagwise dispersions - FDR p-value correction	WT NT - WT.1.S22 - Expression values	WT NT - WT.2.S23 - Expression values	WT NT - WT.3.S24 - Expression values	WT NT - Means	WT H202 - WT.1.H2O2 - Expression values	WT H202 - WT.2.H2O2 - Expression values	WT H202 - WT.3.H2O2 - Expression values	WT H202 - Means
rne	12658	7036	-10034.7	-5.13119	0.000434	-2.44229	2.442293	1.288237	1.288237	0.000979	9446	13237	14708	12463.67	2050	2827	2410	2429
cyaY	942	788	-827.667	-5.13833	2.1E-07	-2.46288	2.462877	1.300345	-1.30034	8.24E-07	982	1112	989	1027.667	236	170	194	200
mreB	2554	1290	-1855.67	-5.13903	2.91E-05	-2.50517	2.505166	1.324906	-1.32491	8.08E-05	1724	2243	2945	2304	520	391	434	448.3333
gutM	527	106	-346.333	-5.13944	0.000632	-2.53309	2.533091	1.340899	1.340899	0.001385	187	587	516	430	81	60	110	83.66667
yigW	575	388	-483	-5.14	6.73E-07	-2.47836	2.478363	1.309388	1.309388	2.45E-06	511	610	678	599.6667	123	103	124	116.6667
SEN0814	394	246	-310.667	-5.14222	1.22E-07	-2.41574	2.415736	1.272463	1.272463	4.89E-07	465	372	320	385.6667	71	74	80	75
sanA	927	509	-719.667	-5.15192	3.82E-07	-2.4225	2.422503	1.276498	1.276498	1.44E-06	1086	920	673	893	197	159	164	173.3333
metG	1590	1507	-1520.33	-5.1577	7.94E-06	-2.42926	2.429256	1.280515	1.280515	2.41E-05	1863	1886	1909	1886	356	422	319	365.6667
menA	293	257	-265.667	-5.17277	2.18E-07	-2.48393	2.483935	1.312627	1.312627	8.52E-07	334	341	313	329.3333	87	48	56	63.66667
pipA	470	249	-306.333	-5.17727	0.000401	-2.50239	2.502385	1.323304	1.323304	0.000907	504	337	298	379.6667	137	34	49	73.33333
pyrE	291	196	-237	-5.18235	8.03E-06	-2.45976	2.459758	1.298516	-1.29852	2.43E-05	258	290	333	293.6667	42	66	62	56.66667
ybbO	702	661	-682.333	-5.18609	8.43E-08	-2.46169	2.46169	1.299649	1.299649	3.44E-07	861	853	822	845.3333	169	161	159	163
ispF	396	300	-343.333	-5.18699	7.47E-07	-2.47161	2.471608	1.30545	1.30545	2.69E-06	380	423	473	425.3333	80	89	77	82
purR	479	427	-438.667	-5.19108	1.92E-07	-2.48885	2.48885	1.315479	-1.31548	7.56E-07	563	546	521	543.3333	136	84	94	104.6667
oppD	7002	5264	-5941.33	-5.19684	3.44E-05	-2.46531	2.465307	1.301767	1.301767	9.41E-05	8306	6670	7095	7357	1406	1304	1537	1415.667
tatB	3115	2352	-2667	-5.2	3.73E-05	-2.47452	2.474517	1.307147	1.307147	0.000102	3020	3216	3670	3302	555	668	682	635
ycjX	1031	894	-922.667	-5.2003	1.04E-06	-2.52037	2.520371	1.333636	1.333636	3.63E-06	1100	1185	1142	1142.333	299	154	206	219.6667
artJ	996	358	-614.667	-5.20046	1.35E-05	-2.40728	2.40728	1.267404	1.267404	3.91E-05	1121	665	497	761	125	139	175	146.3333
tyrP	1429	1148	-1307.33	-5.20364	6.84E-07	-2.47076	2.470762	1.304956	1.304956	2.48E-06	1714	1698	1443	1618.333	353	285	295	311
SEN2343	1052	461	-665.667	-5.20421	2.31E-05	-2.46132	2.461316	1.29943	1.29943	6.51E-05	615	1206	651	824	154	167	154	158.3333
ygiH	1181	681	-837	-5.20603	4.23E-07	-2.46738	2.467384	1.302982	1.302982	1.58E-06	1342	878	888	1036	239	161	197	199
pcm	955	732	-852.667	-5.20724	8.23E-08	-2.47582	2.475817	1.307905	-1.3079	3.37E-07	1129	1100	937	1055.333	205	174	229	202.6667
ybeQ	1231	656	-913	-5.21385	8.51E-07	-2.4634	2.463404	1.300653	1.300653	3.02E-06	1411	1124	854	1129.667	272	180	198	216.6667
yfaX	286	167	-232	-5.21818	6.19E-08	-2.47655	2.47655	1.308332	1.308332	2.59E-07	328	306	227	287	63	42	60	55
yecM	138	99	-115.333	-5.21951	9.48E-06	-2.53314	2.533135	1.340924	1.340924	2.83E-05	126	153	149	142.6667	40	15	27	27.33333
uvrC	3670	2884	-3277.33	-5.22337	1.17E-05	-2.49788	2.49788	1.320704	1.320704	3.42E-05	4297	4174	3689	4053.333	896	627	805	776
SEN4310	367	328	-343.333	-5.23868	2.98E-08	-2.5123	2.512296	1.329006	1.329006	1.31E-07	410	427	436	424.3333	92	69	82	81
yhcB	3903	3653	-3729.33	-5.24915	1.84E-05	-2.49534	2.495339	1.319236	1.319236	5.25E-05	4565	4746	4510	4607	857	843	933	877.6667
argI	499	197	-297.333	-5.26794	1.21E-05	-2.505	2.505	1.324811	-1.32481	3.54E-05	538	279	284	367	88	39	82	69.66667
rplL	35616	13183	-24287.7	-5.28253	0.000713	-2.51929	2.519286	1.333015	1.333015	0.001551	19248	40098	30531	29959	4482	6065	6467	5671.333
SEN1756	310	141	-224.333	-5.28662	8.02E-07	-2.51468	2.514684	1.330377	1.330377	2.86E-06	353	196	281	276.6667	59	43	55	52.33333
ybgE	4102	2756	-3197.67	-5.29409	4.3E-05	-2.52019	2.520186	1.33353	1.33353	0.000116	4677	3414	3736	3942.333	1001	658	575	744.6667
kdtA	1412	1116	-1264.33	-5.30045	2.35E-07	-2.53401	2.534006	1.34142	-1.34142	9.13E-07	1649	1589	1437	1558.333	324	237	321	294
lysP	1852	1117	-1391	-5.3065	8.31E-07	-2.51055	2.510549	1.328003	1.328003	2.96E-06	2128	1588	1426	1714	384	276	309	323
ycfF	914	736	-821.667	-5.30944	2.87E-07	-2.53622	2.536219	1.34268	1.34268	1.1E-06	927	1022	1088	1012.333	207	191	174	190.6667
hisS	3374	2752	-2940.67	-5.31394	7.5E-06	-2.51611	2.516109	1.331194	1.331194	2.29E-05	4017	3441	3409	3622.333	657	643	745	681.6667
nuoM	6393	2965	-4680.33	-5.31898	0.000148	-2.48809	2.488094	1.315041	1.315041	0.000365	5984	7261	4047	5764	868	1082	1301	1083.667

Feature ID	Experiment - Range (original values)	Experiment - IQR (original values)	Experiment - Difference (original values)	Experiment - Fold Change (original values)	EDGE test: WT H202 vs WT NT , tagwise dispersions - P-value	EDGE test: WT H202 vs WT NT , tagwise dispersions - Fold change	WT H202 vs WT NT ABS|FC|	WT H202 vs WT NT Log2FC	WT H202 vs WT NT Log2FC +-	EDGE test: WT H202 vs WT NT , tagwise dispersions - FDR p-value correction	WT NT - WT.1.S22 - Expression values	WT NT - WT.2.S23 - Expression values	WT NT - WT.3.S24 - Expression values	WT NT - Means	WT H202 - WT.1.H2O2 - Expression values	WT H202 - WT.2.H2O2 - Expression values	WT H202 - WT.3.H2O2 - Expression values	WT H202 - Means
rplW	4416	2424	-3058.67	-5.32015	0.001021	-2.45861	2.458608	1.297842	1.297842	0.002142	3093	4865	3342	3766.667	449	1006	669	708
SEN3352	1879	1416	-1512	-5.32412	3.85E-05	-2.58402	2.584022	1.369618	1.369618	0.000105	1808	2111	1666	1861.667	567	232	250	349.6667
yniB	2156	981	-1536.67	-5.32864	2.5E-06	-2.52772	2.527721	1.337837	1.337837	8.16E-06	2411	1919	1345	1891.667	446	255	364	355
tdk	688	474	-563	-5.33077	3.43E-07	-2.50794	2.507941	1.326504	1.326504	1.3E-06	790	678	611	693	151	137	102	130
proB	1466	1267	-1357.67	-5.33759	1.44E-06	-2.52916	2.529161	1.338659	1.338659	4.92E-06	1575	1764	1673	1670.667	308	333	298	313
ntpA	1120	940	-995.667	-5.34157	1.62E-06	-2.52985	2.529853	1.339053	1.339053	5.51E-06	1178	1185	1312	1225	238	258	192	229.3333
srlA	1374	164	-854.333	-5.35144	0.002689	-2.67527	2.675268	1.419683	1.419683	0.005182	343	1487	1322	1050.667	179	113	297	196.3333
ycaO	310	259	-276.333	-5.36316	1.57E-08	-2.543	2.543	1.346531	1.346531	7.26E-08	367	331	321	339.6667	71	62	57	63.33333
yiiQ	238	139	-186.333	-5.36719	5.45E-07	-2.55649	2.55649	1.354164	1.354164	2.02E-06	275	176	236	229	54	37	37	42.66667
trg	5119	2494	-3667.67	-5.36974	0.000182	-2.59949	2.599492	1.37823	1.37823	0.000443	4672	5585	3264	4507	1282	466	770	839.3333
yeaS	906	847	-867	-5.37143	6.14E-08	-2.55779	2.557785	1.354895	1.354895	2.57E-07	1090	1037	1069	1065.333	221	190	184	198.3333
yaeH	5821	3912	-4657.67	-5.37477	7.27E-05	-2.5354	2.535404	1.342216	1.342216	0.000189	6642	4945	5580	5722.333	821	1033	1340	1064.667
tatC	2001	1424	-1750	-5.375	1.22E-06	-2.58324	2.583242	1.369183	1.369183	4.23E-06	1825	2253	2372	2150	401	371	428	400
sfcA	2806	1521	-2196.33	-5.38098	7.11E-06	-2.59216	2.592156	1.374153	1.374153	2.18E-05	2046	3255	2792	2697.667	530	449	525	501.3333
folB	106	90	-95	-5.38462	8.3E-07	-2.57964	2.579644	1.367172	-1.36717	2.95E-06	115	113	122	116.6667	26	16	23	21.66667
mglC	677	311	-444.333	-5.38487	2.94E-06	-2.5694	2.569396	1.361429	-1.36143	9.47E-06	472	761	404	545.6667	127	84	93	101.3333
nuoH	4714	2677	-3437	-5.38953	5.19E-05	-2.53971	2.539712	1.344665	1.344665	0.000139	3800	5375	3485	4220	661	808	880	783
rna-AM93	132	85	-105.667	-5.40278	8.71E-06	-2.58234	2.582341	1.36868	1.36868	2.63E-05	144	137	108	129.6667	23	12	37	24
scsA	221	87	-138.333	-5.41489	1.2E-05	-2.57586	2.575858	1.365053	1.365053	3.52E-05	240	119	150	169.6667	43	19	32	31.33333
yheS	1705	1373	-1554.67	-5.42085	8.07E-07	-2.57999	2.579993	1.367367	1.367367	2.88E-06	1724	2052	1943	1906.333	347	357	351	351.6667
dniR	2591	1666	-2029.67	-5.42193	1.65E-06	-2.54661	2.546615	1.348581	-1.34858	5.59E-06	3039	2124	2303	2488.667	458	471	448	459
SEN2420	252	197	-224.333	-5.42763	1.37E-08	-2.56308	2.563075	1.357876	1.357876	6.42E-08	301	276	248	275	51	52	49	50.66667
mukF	1279	1117	-1196	-5.42963	1.19E-06	-2.56695	2.566955	1.360058	1.360058	4.11E-06	1372	1533	1493	1466	254	301	255	270
SEN3870	4048	462	-1675.33	-5.4321	0.005387	-2.47808	2.478079	1.309222	1.309222	0.009803	4335	994	831	2053.333	478	287	369	378
ndk	1571	918	-1181	-5.43429	2.22E-06	-2.57147	2.571469	1.362593	-1.36259	7.32E-06	1385	1795	1162	1447.333	224	244	331	266.3333
SEN1386	275	186	-211.667	-5.44056	3.67E-08	-2.57311	2.573113	1.363515	1.363515	1.59E-07	317	232	229	259.3333	58	43	42	47.66667
ygdH	20071	16283	-18377.3	-5.44219	2.23E-05	-2.56999	2.569987	1.361761	1.361761	6.31E-05	24062	23128	20353	22514.33	3991	4070	4350	4137
rna-AM93	66	41	-53.3333	-5.44444	8.18E-05	-2.5947	2.594704	1.37557	1.37557	0.000211	71	52	73	65.33333	18	11	7	12
yfiD	14966	4708	-10603.7	-5.44536	0.000367	-2.61448	2.614479	1.386523	1.386523	0.000837	14718	7022	17227	12989	2314	2261	2581	2385.333
citD2	23	7	-16.3333	-5.45455	0.010169	-2.55979	2.55979	1.356025	-1.35603	0.017451	24	11	25	20	4	5	2	3.666667
nusA	6202	4237	-5179.33	-5.4547	7.74E-05	-2.5894	2.589405	1.372621	1.372621	0.0002	5419	6417	7190	6342	988	1318	1182	1162.667
ychJ	479	405	-433.667	-5.45548	1.24E-08	-2.61725	2.617254	1.388054	1.388054	5.8E-08	554	513	526	531	109	75	108	97.33333
SEN4231	301	258	-278	-5.45989	3.33E-08	-2.60734	2.607339	1.382578	1.382578	1.46E-07	318	343	360	340.3333	59	60	68	62.33333
lpxD	5897	4579	-5148	-5.47912	1.1E-05	-2.58673	2.586726	1.371127	1.371127	3.24E-05	7023	6141	5728	6297.333	1173	1126	1149	1149.333
modC	1480	785	-1148.33	-5.47984	1.42E-06	-2.64377	2.643775	1.402599	-1.4026	4.87E-06	1054	1704	1456	1404.667	276	224	269	256.3333
cyaA	8107	6257	-7085	-5.48418	1.04E-05	-2.62145	2.621454	1.390367	1.390367	3.09E-05	8622	9438	7935	8665	1731	1331	1678	1580
yoaB	1055	928	-952	-5.49057	1.26E-07	-2.65707	2.65707	1.409836	1.409836	5.07E-07	1148	1134	1210	1164	275	155	206	212

Feature ID	Experiment - Range (original values)	Experiment - IQR (original values)	Experiment - Difference (original values)	Experiment - Fold Change (original values)	EDGE test: WT H202 vs WT NT , tagwise dispersions - P-value	EDGE test: WT H202 vs WT NT , tagwise dispersions - Fold change	WT H202 vs WT NT ABS|FC|	WT H202 vs WT NT Log2FC	WT H202 vs WT NT Log2FC +-	EDGE test: WT H202 vs WT NT , tagwise dispersions - FDR p-value correction	WT NT - WT.1.S22 - Expression values	WT NT - WT.2.S23 - Expression values	WT NT - WT.3.S24 - Expression values	WT NT - Means	WT H202 - WT.1.H2O2 - Expression values	WT H202 - WT.2.H2O2 - Expression values	WT H202 - WT.3.H2O2 - Expression values	WT H202 - Means
SEN1797	2788	2297	-2543.33	-5.49088	4.03E-06	-2.60837	2.60837	1.383149	1.383149	1.28E-05	3267	2840	3222	3109.667	479	543	677	566.3333
SEN1975	402	350	-363	-5.5	6.98E-08	-2.65748	2.657483	1.41006	1.41006	2.89E-07	424	460	447	443.6667	110	58	74	80.66667
trmU	1360	1035	-1161.67	-5.50258	5.95E-08	-2.61968	2.619675	1.389388	1.389388	2.5E-07	1579	1369	1311	1419.667	276	219	279	258
sodB	27410	15562	-19876.3	-5.51154	2.7E-05	-2.61126	2.611259	1.384745	1.384745	7.52E-05	30987	22017	19842	24282	5360	3577	4280	4405.667
ymdA	147	106	-125	-5.51807	7.73E-07	-2.62389	2.623886	1.391705	1.391705	2.77E-06	135	170	153	152.6667	23	29	31	27.66667
yeiR	1370	866	-1143.67	-5.53836	1.4E-07	-2.64478	2.644776	1.403146	1.403146	5.59E-07	1481	1560	1146	1395.667	280	190	286	252
pyrL	1289	219	-723	-5.54717	0.008078	-2.72904	2.72904	1.448394	1.448394	0.01416	1353	258	1035	882	316	64	97	159
yfbU	2181	1704	-1916.33	-5.54826	6.87E-07	-2.60571	2.605707	1.381675	1.381675	2.49E-06	2568	2314	2131	2337.667	387	450	427	421.3333
yciK	1483	1202	-1367.67	-5.54878	3.09E-07	-2.61003	2.610032	1.384068	1.384068	1.18E-06	1761	1737	1507	1668.333	278	319	305	300.6667
rfbE	8515	4281	-6769.67	-5.54951	0.000179	-2.64015	2.640148	1.400619	1.400619	0.000437	5874	9686	9213	8257.667	1171	1700	1593	1488
SEN0988	941	703	-780.333	-5.55447	3.1E-08	-2.6744	2.674404	1.419217	1.419217	1.36E-07	909	1068	878	951.6667	212	127	175	171.3333
yadB	365	269	-314.333	-5.55556	2.86E-08	-2.62535	2.625351	1.39251	1.39251	1.26E-07	385	423	342	383.3333	76	73	58	69
SEN1402	128	78	-106.333	-5.55714	2.47E-06	-2.68758	2.687579	1.426307	1.426307	8.08E-06	102	142	145	129.6667	29	17	24	23.33333
hydG	3024	2384	-2742	-5.55987	1.21E-06	-2.64311	2.643111	1.402237	1.402237	4.19E-06	3555	3463	3012	3343.333	628	531	645	601.3333
yggX	504	426	-447.667	-5.56803	4.01E-07	-2.65434	2.654343	1.408355	1.408355	1.5E-06	515	579	543	545.6667	75	89	130	98
dsbD	1215	929	-1038.67	-5.56891	2.15E-07	-2.61012	2.610115	1.384114	-1.38411	8.4E-07	1424	1222	1152	1266	209	250	223	227.3333
gppA	2929	2150	-2573.67	-5.57134	8.89E-06	-2.63792	2.637923	1.399402	1.399402	2.67E-05	2746	3399	3265	3136.667	470	623	596	563
hybG	969	588	-721.333	-5.58475	3.74E-07	-2.71151	2.711508	1.439096	1.439096	1.41E-06	745	786	1105	878.6667	179	136	157	157.3333
maeB	10587	8244	-9144.33	-5.58823	1.79E-05	-2.67125	2.671246	1.417513	-1.41751	5.12E-05	10136	12289	10987	11137.33	2385	1892	1702	1993
yggA	911	575	-699	-5.58862	5.24E-06	-2.67744	2.677445	1.420857	1.420857	1.63E-05	1015	858	681	851.3333	247	106	104	152.3333
yejK	1551	1109	-1310.67	-5.59346	6.62E-08	-2.64203	2.642027	1.401645	1.401645	2.76E-07	1820	1572	1396	1596	300	269	287	285.3333
gutQ	2578	1292	-1895.33	-5.60032	2.26E-06	-2.70867	2.708665	1.437582	1.437582	7.43E-06	1736	2907	2279	2307.333	444	329	463	412
SEN4291	311	175	-228.667	-5.60403	2.77E-07	-2.69347	2.693472	1.429467	1.429467	1.07E-06	338	235	262	278.3333	60	27	62	49.66667
ghmA	2457	2267	-2305.67	-5.60519	6.16E-07	-2.67985	2.679846	1.42215	-1.42215	2.25E-06	2756	2754	2909	2806.333	487	452	563	500.6667
rpsQ	17230	6874	-10958	-5.60678	0.000389	-2.63225	2.632246	1.396294	1.396294	0.000883	9219	19110	11681	13336.67	1880	2911	2345	2378.667
rseB	3439	2502	-2734.33	-5.60843	0.000314	-2.79279	2.792789	1.481706	1.481706	0.000729	2967	3313	3703	3327.667	1051	264	465	593.3333
potD	3074	1393	-2314.33	-5.61329	1.78E-05	-2.72319	2.723192	1.445299	-1.4453	5.1E-05	1891	2999	3558	2816	523	484	498	501.6667
ppiC	902	550	-717	-5.61588	2.1E-06	-2.68628	2.686276	1.425608	1.425608	6.95E-06	711	886	1020	872.3333	118	161	187	155.3333
SEN1196	162	54	-100.333	-5.63077	0.000239	-2.65841	2.658414	1.410566	1.410566	0.000568	174	123	69	122	38	12	15	21.66667
galP	1959	1059	-1606.33	-5.64258	5.34E-06	-2.60465	2.604654	1.381092	-1.38109	1.66E-05	2249	2216	1392	1952.333	290	415	333	346
glgX	3097	1981	-2342.67	-5.65122	4.63E-07	-2.67818	2.678176	1.421251	1.421251	1.72E-06	3524	2518	2497	2846.333	568	427	516	503.6667
aas	628	511	-573.667	-5.65135	5.44E-09	-2.68144	2.681436	1.423006	-1.42301	2.65E-08	717	745	629	697	135	117	118	123.3333
dpiA	1003	355	-572.333	-5.65312	8.29E-06	-2.61007	2.610066	1.384086	-1.38409	2.51E-05	1122	488	476	695.3333	121	119	129	123
rpmC	6583	2908	-4064	-5.65521	0.00036	-2.63047	2.630475	1.395323	1.395323	0.000822	3757	7238	3816	4937	655	1115	849	873
hydH	2376	1599	-1937.33	-5.66079	1.15E-07	-2.68109	2.681093	1.422821	1.422821	4.61E-07	2741	2290	2028	2353	453	365	429	415.6667
ychE	398	227	-284.333	-5.6612	3.2E-07	-2.69357	2.693569	1.429519	1.429519	1.22E-06	441	319	276	345.3333	91	43	49	61
yfcY	679	293	-491.333	-5.66456	1.87E-06	-2.7153	2.715296	1.441109	1.441109	6.26E-06	733	639	418	596.6667	137	54	125	105.3333

Feature ID	Experiment - Range (original values)	Experiment - IQR (original values)	Experiment - Difference (original values)	Experiment - Fold Change (original values)	EDGE test: WT H202 vs WT NT , tagwise dispersions - P-value	EDGE test: WT H202 vs WT NT , tagwise dispersions - Fold change	WT H202 vs WT NT ABS|FC|	WT H202 vs WT NT Log2FC	WT H202 vs WT NT Log2FC +-	EDGE test: WT H202 vs WT NT , tagwise dispersions - FDR p-value correction	WT NT - WT.1.S22 - Expression values	WT NT - WT.2.S23 - Expression values	WT NT - WT.3.S24 - Expression values	WT NT - Means	WT H202 - WT.1.H2O2 - Expression values	WT H202 - WT.2.H2O2 - Expression values	WT H202 - WT.3.H2O2 - Expression values	WT H202 - Means
atpG	12000	6529	-9437.33	-5.67041	7.21E-05	-2.71413	2.714131	1.44049	1.44049	0.000188	8540	12037	13797	11458	2011	2254	1797	2020.667
fadL	1556	795	-1098.33	-5.67376	6.01E-07	-2.73759	2.737588	1.452905	-1.45291	2.2E-06	1037	1742	1221	1333.333	277	186	242	235
yajC	2449	1869	-2079	-5.67892	2.54E-05	-2.66059	2.660588	1.411745	1.411745	7.11E-05	2275	2504	2791	2523.333	342	585	406	444.3333
ygiC	5983	3857	-4538	-5.68802	6.21E-06	-2.70859	2.708586	1.43754	1.43754	1.92E-05	6747	4982	4789	5506	1208	764	932	968
argC	477	296	-366	-5.69231	2.82E-09	-2.68198	2.681978	1.423297	-1.4233	1.41E-08	548	411	373	444	77	71	86	78
SEN0222A	2201	798	-1369.67	-5.696	5.27E-05	-2.68762	2.687625	1.426332	1.426332	0.000141	1080	2451	1453	1661.333	250	343	282	291.6667
serC	1834	1678	-1726.67	-5.70481	1.02E-06	-2.67493	2.674929	1.4195	1.4195	3.56E-06	2100	2146	2035	2093.667	312	432	357	367
cafA	1810	1342	-1596.67	-5.70993	1.55E-07	-2.7611	2.761098	1.465242	1.465242	6.18E-07	1710	2018	2079	1935.667	368	269	380	339
glpX	3111	1780	-2330.33	-5.71092	5.47E-06	-2.78749	2.787491	1.478967	1.478967	1.7E-05	2254	3442	2779	2825	679	331	474	494.6667
ycbK	2065	1629	-1775	-5.71239	2.04E-07	-2.74845	2.748454	1.45862	1.45862	8E-07	2087	1967	2401	2151.667	456	336	338	376.6667
ybhF	1170	825	-987.333	-5.71656	4.28E-08	-2.69598	2.695977	1.430808	1.430808	1.84E-07	1183	1368	1039	1196.667	198	216	214	209.3333
fadR	3501	2433	-2740.67	-5.71715	2.33E-05	-2.77038	2.770377	1.470082	-1.47008	6.55E-05	3842	3203	2920	3321.667	915	341	487	581
yliB	1013	844	-931.667	-5.72927	1.7E-08	-2.73177	2.731772	1.449837	1.449837	7.84E-08	1141	1042	1203	1128.667	198	190	203	197
SEN1521	37	16	-23.6667	-5.73333	0.002509	-2.69276	2.692762	1.429087	1.429087	0.004866	20	40	26	28.66667	4	8	3	5
hemX	4313	3454	-3819.33	-5.73471	6.12E-06	-2.73328	2.733283	1.450635	1.450635	1.89E-05	4222	5027	4629	4626	714	768	938	806.6667
yibP	1368	1181	-1265.67	-5.74625	3.86E-07	-2.72202	2.722018	1.444676	1.444676	1.45E-06	1472	1542	1583	1532.333	291	294	215	266.6667
rna-AM93	95	28	-69.6667	-5.75	6.23E-05	-2.6914	2.691402	1.428358	1.428358	0.000164	103	106	44	84.33333	16	11	17	14.66667
artQ	769	682	-723.333	-5.75877	2.27E-09	-2.7525	2.752505	1.460745	-1.46075	1.15E-08	840	907	879	875.3333	160	138	158	152
yedI	444	330	-376.667	-5.76793	1.45E-09	-2.74076	2.740761	1.454577	1.454577	7.52E-09	512	453	402	455.6667	97	68	72	79
pgtB	1151	616	-866.667	-5.77064	2.75E-08	-2.74005	2.740048	1.454201	1.454201	1.22E-07	1287	1065	793	1048.333	232	136	177	181.6667
ltaA	1187	818	-961.667	-5.77649	2.06E-08	-2.72343	2.723425	1.445422	1.445422	9.34E-08	1363	1111	1015	1163	231	197	176	201.3333
glmU	4155	3774	-3947.67	-5.77926	1.78E-06	-2.76803	2.768025	1.468857	1.468857	6E-06	4653	4874	4794	4773.667	879	719	880	826
yfiC	592	536	-556.667	-5.7851	1.37E-09	-2.77367	2.773673	1.471798	1.471798	7.14E-09	678	686	655	673	136	94	119	116.3333
ybhR	629	612	-606.333	-5.78684	5.82E-09	-2.7686	2.768596	1.469155	1.469155	2.83E-08	736	739	724	733	158	112	110	126.6667
sopB	158	58	-115	-5.79167	6.6E-06	-2.7069	2.706895	1.436639	1.436639	2.03E-05	178	160	79	139	31	21	20	24
dapE	1685	1376	-1495.67	-5.7938	5.56E-08	-2.7517	2.751698	1.460322	1.460322	2.35E-07	1752	1985	1686	1807.667	310	300	326	312
mobB	1040	831	-898.333	-5.80392	8.07E-09	-2.75992	2.759915	1.464624	1.464624	3.87E-08	1017	1220	1019	1085.333	186	180	195	187
rna-AM93	119	79	-101	-5.80952	1.06E-07	-2.74589	2.745885	1.457271	1.457271	4.27E-07	127	139	100	122	21	20	22	21
acrR	850	557	-656	-5.81174	2.32E-08	-2.79371	2.793709	1.482182	1.482182	1.04E-07	935	729	713	792.3333	168	85	156	136.3333
cybC	1500	832	-1203.67	-5.81467	5.64E-08	-2.74859	2.748593	1.458693	1.458693	2.38E-07	1553	1721	1087	1453.667	274	221	255	250
nadD	479	354	-409.667	-5.83858	2E-08	-2.76845	2.768453	1.46908	-1.46908	9.12E-08	442	554	487	494.3333	75	91	88	84.66667
priB	4720	1831	-3276	-5.84138	0.000346	-2.63931	2.63931	1.400161	-1.40016	0.000794	5158	4221	2479	3952.667	438	944	648	676.6667
idnD	488	287	-363.333	-5.84444	6.16E-09	-2.7707	2.770698	1.47025	1.47025	2.99E-08	547	413	355	438.3333	98	59	68	75
rna-AM93	1162	735	-996.667	-5.8539	8.22E-09	-2.76265	2.762655	1.466055	1.466055	3.94E-08	1349	1321	936	1202	228	187	201	205.3333
orf408	90	64	-73	-5.86667	1.65E-05	-2.81525	2.815254	1.493265	1.493265	4.76E-05	99	74	91	88	26	9	10	15
SEN2327	1477	786	-1171.33	-5.86704	9.05E-08	-2.78147	2.781474	1.47585	1.47585	3.68E-07	1548	1674	1014	1412	297	197	228	240.6667
hemD	2133	1722	-1948.67	-5.87167	3.75E-08	-2.78565	2.785647	1.478013	1.478013	1.62E-07	2497	2431	2118	2348.667	396	364	440	400

Feature ID	Experiment - Range (original values)	Experiment - IQR (original values)	Experiment - Difference (original values)	Experiment - Fold Change (original values)	EDGE test: WT H202 vs WT NT , tagwise dispersions - P-value	EDGE test: WT H202 vs WT NT , tagwise dispersions - Fold change	WT H202 vs WT NT ABS|FC|	WT H202 vs WT NT Log2FC	WT H202 vs WT NT Log2FC +-	EDGE test: WT H202 vs WT NT , tagwise dispersions - FDR p-value correction	WT NT - WT.1.S22 - Expression values	WT NT - WT.2.S23 - Expression values	WT NT - WT.3.S24 - Expression values	WT NT - Means	WT H202 - WT.1.H2O2 - Expression values	WT H202 - WT.2.H2O2 - Expression values	WT H202 - WT.3.H2O2 - Expression values	WT H202 - Means
tig	8410	5795	-7098.67	-5.88328	2.61E-05	-2.79385	2.793847	1.482253	1.482253	7.28E-05	7142	8769	9746	8552.333	1347	1678	1336	1453.667
nrfD	1938	368	-1306	-5.89139	0.000147	-2.90483	2.904826	1.538452	1.538452	0.000362	653	2146	1920	1573	285	208	308	267
ycdW	1175	786	-1011.67	-5.89516	6.57E-08	-2.85371	2.853708	1.512838	1.512838	2.74E-07	987	1318	1350	1218.333	244	175	201	206.6667
csiE	1858	815	-1261.67	-5.89651	2.42E-07	-2.7462	2.746202	1.457438	-1.45744	9.4E-07	2100	1389	1069	1519.333	254	242	277	257.6667
mltC	633	553	-586	-5.89694	4.37E-09	-2.79741	2.79741	1.484092	-1.48409	2.14E-08	710	739	668	705.6667	106	115	138	119.6667
SEN4216	54651	10824	-33357.7	-5.8981	0.003902	-2.88731	2.887307	1.529725	1.529725	0.007315	58276	14893	47335	40168	12737	3625	4069	6810.333
ybgI	1026	970	-1002	-5.90375	1.39E-08	-2.8008	2.800797	1.485837	1.485837	6.48E-08	1170	1223	1226	1206.333	200	213	200	204.3333
wecE	997	877	-930.667	-5.90685	8.67E-09	-2.81505	2.815053	1.493162	1.493162	4.13E-08	1058	1173	1130	1120.333	176	181	212	189.6667
SEN1677	669	241	-487.667	-5.9094	6.9E-06	-2.8689	2.868901	1.520498	1.520498	2.12E-05	342	655	764	587	102	101	95	99.33333
dmsB	3468	2895	-2957.33	-5.91251	0.000234	-2.91002	2.910019	1.541029	-1.54103	0.000559	3780	3264	3634	3559.333	1125	312	369	602
ygiB	5256	2751	-3557	-5.91298	9.6E-06	-2.81569	2.815686	1.493487	1.493487	2.86E-05	5766	3678	3399	4281	1014	510	648	724
yhcK	1935	1505	-1713.33	-5.91396	2.83E-08	-2.82828	2.828279	1.499924	1.499924	1.25E-07	2214	2145	1827	2062	445	279	322	348.6667
rpsJ	3018	2186	-2394.33	-5.9165	6.16E-05	-2.7293	2.729304	1.448533	1.448533	0.000162	2674	3348	2622	2881.333	330	695	436	487
hybF	1009	692	-774.667	-5.92373	2.22E-07	-2.87787	2.877871	1.525002	1.525002	8.67E-07	825	837	1134	932	214	133	125	157.3333
allA	207	144	-182.333	-5.92793	7.7E-09	-2.83606	2.836057	1.503886	1.503886	3.69E-08	238	182	238	219.3333	42	31	38	37
SEN0764	841	588	-737	-5.94631	3.96E-09	-2.79799	2.797989	1.48439	1.48439	1.95E-08	971	953	734	886	130	146	171	149
cpdB	11919	5598	-8654.33	-5.95005	2.11E-05	-2.88978	2.889781	1.53096	-1.53096	5.98E-05	7278	13330	10600	10402.67	2154	1411	1680	1748.333
pps	22682	17626	-19054	-5.98014	3.58E-06	-2.84575	2.845751	1.508809	1.508809	1.14E-05	26033	21543	21064	22880	4689	3351	3438	3826
SEN3288	2780	2079	-2362	-5.98312	5.24E-07	-2.81892	2.81892	1.495143	1.495143	1.94E-06	3165	2566	2777	2836	385	487	550	474
panB	563	440	-509	-5.9902	7.53E-10	-2.83615	2.836153	1.503935	1.503935	4.12E-09	630	662	541	611	106	99	101	102
dnaA	3760	3356	-3504	-5.9962	1.07E-06	-2.90046	2.900464	1.536284	-1.53628	3.74E-06	4068	4280	4268	4205.333	872	520	712	701.3333
SEN1151	22	12	-16.6667	-6	0.003819	-2.80534	2.805345	1.488178	1.488178	0.007177	25	15	20	20	3	3	4	3.333333
rplP	17054	7596	-10745.7	-6.01119	0.000319	-2.78822	2.788219	1.479344	1.479344	0.00074	9729	18484	10457	12890	1430	2870	2133	2144.333
smtA	1166	1007	-1083	-6.01389	9.37E-08	-2.81704	2.817041	1.494181	1.494181	3.8E-07	1332	1353	1212	1299	205	256	187	216
dksA	7887	6449	-7031.67	-6.02142	6.69E-06	-2.8498	2.849802	1.510862	1.510862	2.06E-05	7910	9040	8346	8432	1153	1461	1587	1400.333
rplE	14086	9989	-11935	-6.03303	0.000135	-2.81252	2.812523	1.491865	1.491865	0.000335	12371	15621	14927	14306.33	1535	3197	2382	2371.333
ycgB	3466	2410	-2907.33	-6.03871	8.87E-07	-2.91781	2.917813	1.544887	1.544887	3.14E-06	3558	3835	3060	3484.333	712	369	650	577
ttrR	544	432	-472	-6.03915	1.14E-09	-2.90227	2.902273	1.537183	1.537183	6.02E-09	533	617	547	565.6667	107	73	101	93.66667
rpsN	7883	5350	-6396.67	-6.04204	0.000143	-2.8102	2.810204	1.490675	1.490675	0.000354	6622	8690	7684	7665.333	807	1727	1272	1268.667
adk	1669	1421	-1567	-6.04399	2.01E-08	-2.88008	2.880077	1.526107	-1.52611	9.16E-08	1936	1733	1964	1877.667	312	295	325	310.6667
hemC	5604	3883	-4760	-6.04415	9E-07	-2.86924	2.869237	1.520667	1.520667	3.18E-06	6413	5854	4844	5703.667	1061	809	961	943.6667
prpE	2274	32	-822.667	-6.05738	0.015195	-3.14352	3.14352	1.652381	-1.65238	0.025075	389	179	2388	985.3333	203	114	171	162.6667
prpD	2176	69	-762	-6.05752	0.023851	-3.16203	3.162033	1.660852	1.660852	0.037812	349	116	2273	912.6667	185	97	170	150.6667
yggN	3016	1534	-2373.67	-6.05753	1.91E-06	-2.95191	2.951908	1.561648	1.561648	6.37E-06	1994	3119	3416	2843	548	400	460	469.3333
yebC	2383	1627	-1972.33	-6.06159	8.73E-06	-2.85024	2.850237	1.511082	1.511082	2.63E-05	1967	2421	2698	2362	315	514	340	389.6667
rrmA	192	139	-153.667	-6.06593	1.16E-08	-2.87603	2.876027	1.524077	1.524077	5.46E-08	215	171	166	184	41	27	23	30.33333
panC	873	636	-771	-6.07237	1.02E-08	-2.84671	2.846708	1.509295	1.509295	4.83E-08	999	974	796	923	160	170	126	152

Feature ID	Experiment - Range (original values)	Experiment - IQR (original values)	Experiment - Difference (original values)	Experiment - Fold Change (original values)	EDGE test: WT H202 vs WT NT , tagwise dispersions - P-value	EDGE test: WT H202 vs WT NT , tagwise dispersions - Fold change	WT H202 vs WT NT ABS|FC|	WT H202 vs WT NT Log2FC	WT H202 vs WT NT Log2FC +-	EDGE test: WT H202 vs WT NT , tagwise dispersions - FDR p-value correction	WT NT - WT.1.S22 - Expression values	WT NT - WT.2.S23 - Expression values	WT NT - WT.3.S24 - Expression values	WT NT - Means	WT H202 - WT.1.H2O2 - Expression values	WT H202 - WT.2.H2O2 - Expression values	WT H202 - WT.3.H2O2 - Expression values	WT H202 - Means
yheN	488	249	-365.333	-6.07407	3.55E-07	-2.95928	2.959284	1.565248	1.565248	1.35E-06	312	452	548	437.3333	93	63	60	72
yhbV	1264	409	-912.333	-6.07792	0.000206	-2.88033	2.880333	1.526235	1.526235	0.000497	564	1319	1393	1092	129	255	155	179.6667
fliG	785	545	-665.667	-6.12051	1.83E-09	-2.95031	2.950305	1.560864	1.560864	9.33E-09	687	890	810	795.6667	143	105	142	130
rplA	15988	8665	-10991	-6.13838	3.26E-05	-2.87292	2.872921	1.522518	1.522518	8.96E-05	10846	17767	10777	13130	1779	2526	2112	2139
efp	2848	1912	-2459.33	-6.14505	2.75E-07	-2.95332	2.95332	1.562337	1.562337	1.06E-06	2391	3111	3310	2937.333	493	462	479	478
rpsH	6589	4435	-5321.33	-6.153	0.000165	-2.85501	2.855011	1.513496	1.513496	0.000403	5463	7211	6388	6354	622	1448	1028	1032.667
sdaB	5553	2448	-3921.67	-6.15331	3.29E-05	-3.00988	3.009878	1.589705	1.589705	9.04E-05	3137	4777	6134	4682.667	1013	689	581	761
yajG	528	393	-461	-6.16045	1.38E-09	-2.96168	2.961683	1.566417	1.566417	7.17E-09	569	480	602	550.3333	107	74	87	89.33333
pgsA	1493	855	-1113.33	-6.17829	3.7E-08	-2.96195	2.96195	1.566547	-1.56655	1.61E-07	1628	1282	1075	1328.333	290	135	220	215
SEN1403	347	284	-314.667	-6.18681	2.75E-09	-2.93407	2.934072	1.552904	1.552904	1.38E-08	380	346	400	375.3333	62	67	53	60.66667
fumB	25123	11821	-19677.7	-6.18926	4.34E-05	-2.96117	2.961166	1.566165	-1.56617	0.000117	27079	15492	27838	23469.67	4990	3671	2715	3792
fabH	1236	986	-1076.67	-6.19293	2.36E-08	-2.95714	2.957135	1.5642	1.5642	1.06E-07	1188	1254	1410	1284	174	202	246	207.3333
kdgK	1140	871	-973.667	-6.19751	2.33E-08	-2.92699	2.926987	1.549416	-1.54942	1.05E-07	1278	1071	1134	1161	224	200	138	187.3333
yidQ	921	716	-812.333	-6.20726	1.54E-10	-2.96926	2.969257	1.570102	1.570102	9.04E-10	976	1047	882	968.3333	176	126	166	156
fucA	157	128	-142.333	-6.20732	6.39E-10	-2.94467	2.944674	1.558108	-1.55811	3.52E-09	182	173	154	169.6667	31	25	26	27.33333
bax	2842	2163	-2324	-6.21076	1.41E-06	-2.98613	2.986132	1.578278	1.578278	4.83E-06	3164	2656	2490	2770	689	327	322	446
corE	477	351	-400	-6.21739	5.12E-10	-2.98448	2.984477	1.577478	-1.57748	2.85E-09	528	473	429	476.6667	101	51	78	76.66667
gntT	1039	526	-756.667	-6.23041	2.34E-08	-3.00351	3.003512	1.58665	1.58665	1.05E-07	683	1158	863	901.3333	157	119	158	144.6667
tag	2366	2001	-2145	-6.23171	1.46E-08	-2.99733	2.99733	1.583678	1.583678	6.8E-08	2514	2445	2706	2555	444	340	446	410
serS	18028	13714	-16487.3	-6.23463	1.29E-06	-2.94312	2.943123	1.557348	1.557348	4.45E-06	20993	21067	16851	19637	3137	3039	3273	3149.667
SEN1301	403	358	-384.333	-6.24091	2.96E-10	-2.9652	2.965198	1.568128	1.568128	1.69E-09	467	432	474	457.6667	74	75	71	73.33333
folC	875	827	-837.333	-6.24426	7.1E-09	-2.94298	2.942979	1.557277	-1.55728	3.43E-08	993	981	1017	997	142	183	154	159.6667
artI	2542	2038	-2283	-6.2523	7.17E-08	-2.97946	2.979458	1.57505	-1.57505	2.96E-07	2461	2939	2753	2717.667	397	423	484	434.6667
tsf	16381	4670	-9576.33	-6.26654	0.000185	-2.96506	2.965055	1.568059	1.568059	0.000448	6421	17984	9779	11394.67	1603	2101	1751	1818.333
mgsA	3188	2930	-3008	-6.27719	1.56E-07	-2.98571	2.985705	1.578072	-1.57807	6.2E-07	3519	3730	3485	3578	555	542	613	570
fabA	1524	817	-1141.67	-6.27735	3.16E-06	-2.98509	2.985093	1.577776	1.577776	1.02E-05	1015	1357	1702	1358	198	273	178	216.3333
fbp	12297	8773	-10111	-6.28634	1.72E-06	-3.02497	3.024971	1.596921	-1.59692	5.8E-06	10488	13927	11656	12023.67	2393	1630	1715	1912.667
bcp	3349	3011	-3150.67	-6.28635	1.96E-07	-2.98198	2.981976	1.576269	-1.57627	7.71E-07	3913	3594	3733	3746.667	564	583	641	596
yfbS	1829	1183	-1461	-6.28709	2.3E-08	-2.9504	2.950398	1.56091	1.56091	1.04E-07	2058	1703	1451	1737.333	229	268	332	276.3333
yadE	1421	811	-1094.67	-6.28824	2.9E-09	-2.95269	2.952688	1.562029	1.562029	1.45E-08	1609	1289	1007	1301.667	237	196	188	207
hnr	1522	1299	-1401	-6.30013	1.1E-08	-3.02048	3.020484	1.59478	-1.59478	5.19E-08	1545	1699	1752	1665.333	317	246	230	264.3333
mlc	872	685	-777.667	-6.31435	1.08E-09	-3.00925	3.009246	1.589402	-1.5894	5.69E-09	993	969	810	924	193	125	121	146.3333
barA	2098	1685	-1900.67	-6.31903	1.78E-09	-3.01196	3.011959	1.590702	1.590702	9.13E-09	2310	2409	2055	2258	391	311	370	357.3333
SEN0167	3098	129	-1188.33	-6.3209	0.01384	-3.02071	3.020708	1.594887	1.594887	0.023045	808	3213	214	1411.667	213	115	342	223.3333
asrA	134	70	-99.3333	-6.32143	5.86E-07	-2.93592	2.935916	1.553811	-1.55381	2.15E-06	149	118	87	118	15	24	17	18.66667
ybjE	547	499	-515.667	-6.33448	5.04E-11	-3.02944	3.02944	1.599051	1.599051	3.13E-10	628	606	603	612.3333	104	81	105	96.66667
SEN3427	3929	2595	-3269.67	-6.33678	2.09E-06	-3.05829	3.058291	1.612726	1.612726	6.92E-06	4192	4315	3140	3882.333	907	386	545	612.6667

Feature ID	Experiment - Range (original values)	Experiment - IQR (original values)	Experiment - Difference (original values)	Experiment - Fold Change (original values)	EDGE test: WT H202 vs WT NT , tagwise dispersions - P-value	EDGE test: WT H202 vs WT NT , tagwise dispersions - Fold change	WT H202 vs WT NT ABS|FC|	WT H202 vs WT NT Log2FC	WT H202 vs WT NT Log2FC +-	EDGE test: WT H202 vs WT NT , tagwise dispersions - FDR p-value correction	WT NT - WT.1.S22 - Expression values	WT NT - WT.2.S23 - Expression values	WT NT - WT.3.S24 - Expression values	WT NT - Means	WT H202 - WT.1.H2O2 - Expression values	WT H202 - WT.2.H2O2 - Expression values	WT H202 - WT.3.H2O2 - Expression values	WT H202 - Means
hypE	8505	5251	-6900.33	-6.34633	7.49E-06	-3.00838	3.00838	1.588987	1.588987	2.29E-05	6550	9603	8420	8191	1299	1475	1098	1290.667
SEN2527	347	117	-209	-6.35897	9.49E-07	-2.99002	2.990018	1.580154	1.580154	3.34E-06	372	219	153	248	56	25	36	39
frdC	15271	11755	-12694	-6.36215	7.63E-06	-3.08945	3.089446	1.627348	-1.62735	2.33E-05	14527	13640	17017	15061.33	3471	1885	1746	2367.333
rplD	5702	3460	-4165.67	-6.39129	0.00018	-2.93967	2.939666	1.555652	1.555652	0.000438	4158	6161	4496	4938.333	459	1161	698	772.6667
hsdM	773	577	-683.667	-6.39737	1.04E-09	-3.07657	3.07657	1.621323	1.621323	5.54E-09	697	844	890	810.3333	143	117	120	126.6667
SEN0815	228	176	-183.667	-6.40196	8.19E-07	-3.08002	3.080023	1.622941	1.622941	2.92E-06	249	207	197	217.6667	60	21	21	34
potC	1040	699	-825	-6.40393	9.17E-10	-3.09005	3.090046	1.627629	-1.62763	4.91E-09	859	1154	920	977.6667	184	114	160	152.6667
cmk	643	488	-542	-6.42	7.76E-11	-3.06991	3.069913	1.618198	-1.6182	4.71E-10	722	587	617	642	122	79	99	100
SEN3135	324	267	-296.667	-6.42683	9.91E-12	-3.06606	3.066058	1.616385	1.616385	6.83E-11	368	360	326	351.3333	59	44	61	54.66667
rpsF	8375	3096	-5747.67	-6.43772	0.000238	-2.89226	2.892261	1.532198	1.532198	0.000567	9042	7311	4061	6804.667	667	1539	965	1057
ddlA	1485	1425	-1408.33	-6.45161	7.14E-09	-3.0912	3.091197	1.628166	1.628166	3.44E-08	1697	1653	1650	1666.667	338	225	212	258.3333
SEN1071	194	105	-151.333	-6.46988	7.9E-07	-3.0719	3.0719	1.619131	1.619131	2.83E-06	131	216	190	179	26	35	22	27.66667
secG	4331	3064	-3762.67	-6.47961	1.2E-07	-3.05617	3.056174	1.611727	1.611727	4.82E-07	4987	4633	3728	4449.333	740	656	664	686.6667
rplC	9682	6319	-7257	-6.48112	0.000113	-2.98336	2.983357	1.576937	1.576937	0.000283	7499	10528	7716	8581	846	1946	1180	1324
envZ	1696	1481	-1596.33	-6.48568	8.39E-10	-3.09929	3.099286	1.631936	-1.63194	4.54E-09	1941	1943	1778	1887.333	329	247	297	291
yhjW	2420	1843	-2115.67	-6.52393	7.29E-08	-3.1837	3.183698	1.670703	1.670703	3.01E-07	2191	2619	2686	2498.667	535	266	348	383
SEN3211	1156	689	-987.667	-6.52799	2.01E-08	-3.14131	3.141306	1.651365	1.651365	9.16E-08	866	1303	1330	1166.333	185	177	174	178.6667
SEN4051	1198	236	-848.667	-6.53478	4.21E-05	-3.22225	3.222251	1.688069	1.688069	0.000114	384	1329	1293	1002	181	131	148	153.3333
fabB	18619	10356	-14169.3	-6.545	4.5E-06	-3.15157	3.151573	1.656072	-1.65607	1.41E-05	13006	16200	20968	16724.67	2667	2650	2349	2555.333
rna-AM93	177	128	-148	-6.55	5.05E-08	-3.11791	3.117913	1.64058	1.64058	2.15E-07	180	193	151	174.6667	41	23	16	26.66667
yedE	350	124	-272.333	-6.55782	1.75E-06	-3.18846	3.188465	1.672862	1.672862	5.91E-06	172	395	397	321.3333	52	47	48	49
gpsA	7491	5725	-6762	-6.56238	5.69E-07	-3.13821	3.13821	1.649942	1.649942	2.09E-06	6940	8670	8323	7977.667	1253	1179	1215	1215.667
yabI	607	372	-480.333	-6.56371	4.75E-09	-3.19505	3.195055	1.675841	1.675841	2.32E-08	470	661	569	566.6667	107	54	98	86.33333
frr	2973	2709	-2804.67	-6.5685	1.76E-08	-3.11643	3.116434	1.639896	1.639896	8.07E-08	3443	3278	3204	3308.333	546	495	470	503.6667
yoaE	615	468	-510	-6.58394	2.27E-10	-3.17048	3.170483	1.664703	1.664703	1.32E-09	564	682	558	601.3333	117	67	90	91.33333
rplB	17761	8847	-11696.7	-6.58758	0.000252	-3.02881	3.02881	1.598751	1.598751	0.000595	10829	18958	11583	13790	1197	3101	1982	2093.333
aer	2629	1014	-1656	-6.5883	0.000339	-3.2397	3.239697	1.695859	-1.69586	0.00078	1906	2726	1225	1952.333	581	97	211	296.3333
fliB	2579	1736	-2072.33	-6.60595	3.21E-09	-3.16734	3.167338	1.663271	-1.66327	1.6E-08	2335	2869	2122	2442	433	290	386	369.6667
torC	222	191	-200.333	-6.61682	1.05E-08	-3.1143	3.114298	1.638907	1.638907	4.97E-08	225	250	233	236	28	45	34	35.66667
napG	3553	413	-1951	-6.62788	0.00143	-3.14099	3.140988	1.651218	1.651218	0.002918	848	3706	2339	2297.667	153	435	452	346.6667
btuC	230	155	-182.667	-6.64948	1.47E-09	-3.14323	3.143229	1.652248	1.652248	7.62E-09	253	188	204	215	41	33	23	32.33333
hybC	16994	12975	-14229	-6.65615	1.68E-06	-3.20696	3.206961	1.681207	1.681207	5.7E-06	15304	15986	18944	16744.67	3268	2329	1950	2515.667
srfA	432	317	-383	-6.6601	9.62E-11	-3.20829	3.208294	1.681806	1.681806	5.78E-10	387	476	489	450.6667	70	57	76	67.66667
fhlA	6582	3295	-5122.33	-6.67048	2.25E-06	-3.25005	3.250049	1.700462	1.700462	7.42E-06	4177	7306	6594	6025.667	1104	724	882	903.3333
sspA	6668	6297	-6470.33	-6.68238	2.97E-07	-3.16985	3.169846	1.664413	1.664413	1.14E-06	7449	7706	7672	7609	1038	1152	1226	1138.667
fldA	2468	2076	-2169.33	-6.69379	6.56E-09	-3.22589	3.225886	1.689695	-1.6897	3.18E-08	2744	2486	2421	2550.333	522	276	345	381
dnaX	2581	2162	-2338.33	-6.69862	5.14E-09	-3.18032	3.180321	1.669172	-1.66917	2.51E-08	2742	2940	2564	2748.667	470	402	359	410.3333

Feature ID	Experiment - Range (original values)	Experiment - IQR (original values)	Experiment - Difference (original values)	Experiment - Fold Change (original values)	EDGE test: WT H202 vs WT NT , tagwise dispersions - P-value	EDGE test: WT H202 vs WT NT , tagwise dispersions - Fold change	WT H202 vs WT NT ABS|FC|	WT H202 vs WT NT Log2FC	WT H202 vs WT NT Log2FC +-	EDGE test: WT H202 vs WT NT , tagwise dispersions - FDR p-value correction	WT NT - WT.1.S22 - Expression values	WT NT - WT.2.S23 - Expression values	WT NT - WT.3.S24 - Expression values	WT NT - Means	WT H202 - WT.1.H2O2 - Expression values	WT H202 - WT.2.H2O2 - Expression values	WT H202 - WT.3.H2O2 - Expression values	WT H202 - Means
rna-AM93	28	13	-19	-6.7	0.001526	-3.11197	3.111974	1.63783	-1.63783	0.003088	29	17	21	22.33333	1	5	4	3.333333
yhbQ	1519	1047	-1296.33	-6.71072	2.63E-10	-3.18369	3.183693	1.670701	1.670701	1.51E-09	1562	1723	1285	1523.333	238	204	239	227
yqfB	701	524	-619	-6.71385	9.06E-12	-3.21199	3.211989	1.683467	1.683467	6.3E-11	757	783	642	727.3333	125	82	118	108.3333
cysM	332	169	-233	-6.72951	1.04E-09	-3.13519	3.135194	1.648555	-1.64855	5.53E-09	371	241	209	273.6667	40	43	39	40.66667
gcvR	1643	1167	-1349	-6.74043	4.42E-10	-3.17686	3.176864	1.667603	-1.6676	2.47E-09	1852	1501	1399	1584	264	232	209	235
malG	12249	9250	-10648	-6.76087	1.14E-07	-3.21608	3.216084	1.685305	1.685305	4.6E-07	12395	13923	11171	12496.33	1950	1674	1921	1848.333
dcuC	1390	1098	-1223.67	-6.79022	9.1E-08	-3.18006	3.180055	1.669052	1.669052	3.69E-07	1456	1289	1560	1435	191	273	170	211.3333
glk	2300	1656	-1876.33	-6.81508	3.87E-09	-3.21757	3.217574	1.685973	-1.68597	1.92E-08	2547	2074	1976	2199	401	320	247	322.6667
pmgI	7523	5153	-6151	-6.8193	0.000157	-3.13473	3.134725	1.648339	1.648339	0.000384	5921	8213	7490	7208	690	1713	768	1057
SEN3424	3868	594	-1751	-6.83019	0.000254	-3.09343	3.093429	1.629207	1.629207	0.000601	4160	889	1105	2051.333	295	292	314	300.3333
rpsC	26708	12203	-17167	-6.83052	0.000137	-3.15504	3.15504	1.657658	1.657658	0.000341	15106	28468	16760	20111.33	1760	4170	2903	2944.333
yaeB	420	355	-379	-6.83077	9.52E-12	-3.27849	3.278489	1.713031	1.713031	6.6E-11	467	449	416	444	87	47	61	65
tatA	3720	2623	-3034	-6.83088	5.57E-07	-3.2408	3.240803	1.696352	1.696352	2.05E-06	3166	3354	4143	3554.333	423	595	543	520.3333
rna-AM93	116	66	-83.6667	-6.83721	6.54E-08	-3.23785	3.237852	1.695037	-1.69504	2.74E-07	126	79	89	98	20	10	13	14.33333
SEN3859	422	52	-175.333	-6.84444	0.001482	-3.07957	3.079574	1.622731	1.622731	0.003009	449	87	80	205.3333	35	28	27	30
selA	5054	3698	-4221.33	-6.84942	6.56E-08	-3.28487	3.284867	1.715835	1.715835	2.74E-07	4808	5633	4388	4943	896	579	690	721.6667
dlhH	1001	621	-793	-6.88861	2.11E-10	-3.31717	3.317168	1.729952	-1.72995	1.23E-09	1088	775	920	927.6667	154	87	163	134.6667
SEN1793	979	187	-549	-6.90323	3.16E-05	-3.41706	3.417064	1.772757	1.772757	8.69E-05	279	587	1060	642	106	92	81	93
dacA	1085	1022	-1040	-6.90909	5.04E-11	-3.28567	3.285669	1.716187	-1.71619	3.13E-10	1238	1216	1194	1216	203	172	153	176
SEN3167	2460	1020	-1530.67	-6.91753	1.03E-07	-3.31313	3.31313	1.728195	1.728195	4.15E-07	1410	2663	1295	1789.333	298	203	275	258.6667
rna-AM93	262	143	-203.333	-6.92233	2.27E-11	-3.26077	3.26077	1.705213	1.705213	1.48E-10	291	242	180	237.6667	37	29	37	34.33333
yeaJ	389	292	-322.333	-6.93252	9.98E-12	-3.32072	3.320725	1.731498	1.731498	6.86E-11	426	359	345	376.6667	73	37	53	54.33333
SEN3097	1213	990	-1091.67	-6.96539	1.93E-11	-3.33168	3.331682	1.736251	1.736251	1.27E-10	1360	1298	1166	1274.667	226	147	176	183
aspA	303073	265915	-278848	-6.97583	6.13E-07	-3.36557	3.365574	1.750853	-1.75085	2.24E-06	304500	341582	330451	325511	62894	38509	38585	46662.67
pepQ	3599	2699	-3126.67	-6.98978	1.86E-07	-3.31605	3.316047	1.729464	-1.72946	7.34E-07	3219	3679	4048	3648.667	520	597	449	522
ybfE	378	217	-292.333	-7.00685	7.6E-12	-3.31674	3.316743	1.729767	1.729767	5.36E-11	414	342	267	341	50	36	60	48.66667
yffH	268	223	-242.333	-7.00826	9.18E-12	-3.37345	3.37345	1.754225	1.754225	6.37E-11	268	296	284	282.6667	48	28	45	40.33333
yfiO	2766	2570	-2605	-7.06284	1.07E-09	-3.40036	3.400361	1.765688	1.765688	5.67E-09	2978	3008	3118	3034.667	529	352	408	429.6667
glgC	5900	2787	-3922	-7.06495	1.42E-07	-3.34121	3.341213	1.740372	-1.74037	5.67E-07	6379	3877	3450	4568.667	798	479	663	646.6667
hlpA	8932	6505	-7700.33	-7.07921	1.85E-06	-3.33956	3.339558	1.739657	1.739657	6.19E-06	7841	9177	9883	8967	951	1513	1336	1266.667
rpsO	3718	2622	-3107.33	-7.08089	1.83E-07	-3.33569	3.335692	1.737986	1.737986	7.27E-07	3128	4148	3579	3618.333	430	597	506	511
secD	3809	3572	-3659	-7.08144	1.3E-07	-3.32733	3.327326	1.734363	1.734363	5.19E-07	4294	4166	4322	4260.667	513	698	594	601.6667
thrS	55313	37752	-44171.3	-7.10776	9.06E-08	-3.37059	3.370591	1.753002	1.753002	3.68E-07	61454	47429	45327	51403.33	7575	6141	7980	7232
aroA	1143	790	-963	-7.10782	2.15E-10	-3.31319	3.313186	1.728219	-1.72822	1.24E-09	1274	1141	947	1120.667	157	185	131	157.6667
SEN1796	7675	569	-3429.67	-7.12805	0.002283	-3.51215	3.51215	1.812354	1.812354	0.00447	1113	2782	8073	3989.333	398	737	544	559.6667
ttrC	125	73	-88	-7.13953	2.77E-08	-3.37783	3.377826	1.756095	1.756095	1.23E-07	134	87	86	102.3333	21	9	13	14.33333
SEN2978	7718	3076	-5145	-7.18142	5.6E-07	-3.41582	3.415823	1.772233	1.772233	2.07E-06	5608	8366	3958	5977.333	882	648	967	832.3333

Feature ID	Experiment - Range (original values)	Experiment - IQR (original values)	Experiment - Difference (original values)	Experiment - Fold Change (original values)	EDGE test: WT H202 vs WT NT , tagwise dispersions - P-value	EDGE test: WT H202 vs WT NT , tagwise dispersions - Fold change	WT H202 vs WT NT ABS|FC|	WT H202 vs WT NT Log2FC	WT H202 vs WT NT Log2FC +-	EDGE test: WT H202 vs WT NT , tagwise dispersions - FDR p-value correction	WT NT - WT.1.S22 - Expression values	WT NT - WT.2.S23 - Expression values	WT NT - WT.3.S24 - Expression values	WT NT - Means	WT H202 - WT.1.H2O2 - Expression values	WT H202 - WT.2.H2O2 - Expression values	WT H202 - WT.3.H2O2 - Expression values	WT H202 - Means
hpt	435	403	-416.667	-7.18812	9.56E-12	-3.38628	3.386282	1.759702	1.759702	6.62E-11	497	489	466	484	62	77	63	67.33333
flhC	1987	1555	-1703.67	-7.21021	2.63E-10	-3.4655	3.4655	1.793063	1.793063	1.51E-09	2196	1804	1934	1978	365	209	249	274.3333
rpsS	6281	3003	-4292.67	-7.21525	5.63E-05	-3.33585	3.335847	1.738053	1.738053	0.00015	3672	6670	4608	4983.333	389	1014	669	690.6667
ispD	707	612	-649	-7.22045	3.52E-12	-3.46235	3.462352	1.791752	1.791752	2.58E-11	714	748	798	753.3333	102	91	120	104.3333
ttrB	168	97	-124.667	-7.23333	7.5E-10	-3.43723	3.437229	1.781246	1.781246	4.11E-09	179	135	120	144.6667	26	11	23	20
SEN1795	1808	203	-860.333	-7.2343	0.00029	-3.59951	3.599512	1.847801	1.847801	0.000677	344	720	1931	998.3333	123	150	141	138
glyQ	1600	1366	-1493.33	-7.26573	8.69E-10	-3.44928	3.449277	1.786294	1.786294	4.68E-09	1623	1790	1782	1731.667	268	257	190	238.3333
ycfX	978	751	-850.333	-7.26781	1.41E-12	-3.42708	3.42708	1.77698	1.77698	1.08E-11	1104	969	885	986	126	134	147	135.6667
SEN1557	568	357	-433	-7.27536	4.55E-11	-3.43876	3.438763	1.78189	1.78189	2.85E-10	448	629	429	502	72	74	61	69
SEN0216	4106	1875	-2628	-7.28207	1.96E-08	-3.44176	3.441756	1.783145	1.783145	8.94E-08	4418	2305	2416	3046.333	513	312	430	418.3333
SEN1442	367	125	-203.667	-7.29897	2.48E-07	-3.41029	3.410287	1.769893	1.769893	9.61E-07	390	157	161	236	42	23	32	32.33333
dapA	7323	6114	-6680.33	-7.3022	1.14E-08	-3.46295	3.462947	1.792	-1.792	5.36E-08	8320	7731	7170	7740.333	1127	997	1056	1060
SEN4192	258	174	-206	-7.30612	2.86E-11	-3.46472	3.464724	1.79274	1.79274	1.82E-10	220	290	206	238.6667	32	32	34	32.66667
kbl	9208	6107	-7809.33	-7.30972	1.62E-07	-3.50629	3.506291	1.809946	1.809946	6.41E-07	7361	9370	10410	9047	1257	1254	1202	1237.667
mobA	1318	1059	-1147.67	-7.31743	4.93E-12	-3.51458	3.514575	1.81335	-1.81335	3.55E-11	1259	1462	1267	1329.333	200	144	201	181.6667
ygbQ	582	565	-567.333	-7.32714	3.24E-13	-3.50723	3.507228	1.810331	1.810331	2.7E-12	655	659	657	657	102	77	90	89.66667
yacE	585	377	-464.667	-7.33636	4.99E-13	-3.48932	3.489319	1.802945	1.802945	4.07E-12	640	519	455	538	87	55	78	73.33333
pstB	496	432	-452	-7.33645	4.36E-13	-3.49114	3.491139	1.803698	-1.8037	3.6E-12	510	562	498	523.3333	66	66	82	71.33333
rplV	10794	5078	-7084	-7.3705	4.94E-05	-3.40035	3.400348	1.765682	1.765682	0.000133	6134	11456	6998	8196	662	1618	1056	1112
flgB	366	169	-257.333	-7.38017	3.17E-08	-3.56541	3.565411	1.834068	-1.83407	1.39E-07	208	281	404	297.6667	39	44	38	40.33333
SEN3095	1488	1055	-1280.67	-7.38206	6.39E-11	-3.54204	3.542037	1.824579	1.824579	3.9E-10	1567	1632	1245	1481.333	268	144	190	200.6667
atpF	10671	4852	-8016.33	-7.40453	1.09E-06	-3.58732	3.587323	1.842908	1.842908	3.8E-06	6104	9789	11911	9268	1240	1252	1263	1251.667
napB	3179	356	-2034.33	-7.41746	0.000343	-3.59145	3.591454	1.844568	1.844568	0.000787	705	3361	2988	2351.333	182	349	420	317
SEN3595	2216	2047	-2130	-7.44153	2.14E-11	-3.53935	3.539346	1.823483	1.823483	1.41E-10	2527	2478	2377	2460.667	351	311	330	330.6667
rplX	9048	6500	-7528.33	-7.45839	4.44E-06	-3.46941	3.469414	1.794692	1.794692	1.39E-05	7700	9785	8597	8694	737	1560	1200	1165.667
btuB	2740	1517	-2073.67	-7.46002	2.72E-09	-3.62892	3.628916	1.859539	1.859539	1.37E-08	1854	2313	3017	2394.667	337	277	349	321
rna-AM93	976	835	-878.667	-7.46078	1.39E-11	-3.59433	3.594332	1.845724	-1.84572	9.33E-11	1073	1018	953	1014.667	193	97	118	136
yajD	907	558	-672	-7.46154	3.07E-12	-3.54926	3.549259	1.827518	1.827518	2.27E-11	984	682	662	776	131	77	104	104
emrR	1665	1462	-1493	-7.4632	8.3E-10	-3.61069	3.610694	1.852276	1.852276	4.5E-09	1823	1696	1653	1724	344	158	191	231
rna-AM93	361	203	-289.333	-7.47761	2.81E-11	-3.5446	3.544602	1.825624	1.825624	1.8E-10	394	367	241	334	63	33	38	44.66667
rpmH	1196	724	-992	-7.49782	1.98E-10	-3.60792	3.607924	1.851169	1.851169	1.15E-09	876	1212	1346	1144.667	150	152	156	152.6667
pth	533	509	-507	-7.5	2.84E-12	-3.61713	3.61713	1.854845	1.854845	2.11E-11	584	581	590	585	105	57	72	78
rhaR	383	271	-307.667	-7.5	9.58E-13	-3.58375	3.583747	1.841469	1.841469	7.55E-12	323	422	320	355	49	39	54	47.33333
rplN	9483	6202	-7513.67	-7.50534	1.2E-06	-3.51265	3.512654	1.812562	1.812562	4.15E-06	7426	10297	8283	8668.667	814	1427	1224	1155
ipk	1226	1020	-1106.33	-7.50784	7.44E-12	-3.58928	3.589283	1.843696	1.843696	5.27E-11	1270	1183	1376	1276.333	197	163	150	170
yhbP	1100	747	-869.333	-7.52	5.44E-12	-3.5655	3.565497	1.834103	1.834103	3.91E-11	897	1231	880	1002.667	131	133	136	133.3333
yjjP	641	495	-571.333	-7.54198	1.75E-12	-3.62189	3.621889	1.856742	1.856742	1.32E-11	585	670	721	658.6667	92	80	90	87.33333

Feature ID	Experiment - Range (original values)	Experiment - IQR (original values)	Experiment - Difference (original values)	Experiment - Fold Change (original values)	EDGE test: WT H202 vs WT NT , tagwise dispersions - P-value	EDGE test: WT H202 vs WT NT , tagwise dispersions - Fold change	WT H202 vs WT NT ABS|FC|	WT H202 vs WT NT Log2FC	WT H202 vs WT NT Log2FC +-	EDGE test: WT H202 vs WT NT , tagwise dispersions - FDR p-value correction	WT NT - WT.1.S22 - Expression values	WT NT - WT.2.S23 - Expression values	WT NT - WT.3.S24 - Expression values	WT NT - Means	WT H202 - WT.1.H2O2 - Expression values	WT H202 - WT.2.H2O2 - Expression values	WT H202 - WT.3.H2O2 - Expression values	WT H202 - Means
rfaL	2705	1196	-2128.33	-7.55544	4.88E-08	-3.69601	3.696006	1.885967	1.885967	2.08E-07	1522	2873	2964	2453	326	259	389	324.6667
yqgE	1003	806	-907.333	-7.55904	2.11E-13	-3.59926	3.599257	1.847699	1.847699	1.78E-12	1130	949	1058	1045.667	143	127	145	138.3333
melR	2746	1853	-2117.67	-7.5766	2.03E-07	-3.71009	3.710087	1.891453	1.891453	7.98E-07	2295	2912	2112	2439.667	541	166	259	322
sapC	296	194	-234.667	-7.57944	2.8E-11	-3.66048	3.660478	1.872032	1.872032	1.8E-10	235	318	258	270.3333	44	22	41	35.66667
yfiQ	8547	6420	-7346	-7.58834	2.13E-08	-3.6566	3.656603	1.870504	1.870504	9.63E-08	9300	8531	7552	8461	1460	753	1132	1115
SEN4190	167	102	-127.667	-7.60345	6.65E-09	-3.59424	3.594241	1.845687	1.845687	3.22E-08	123	181	137	147	14	23	21	19.33333
yjeQ	2095	1749	-1883.67	-7.6171	2.82E-11	-3.65529	3.655291	1.869986	1.869986	1.8E-10	2316	2190	1999	2168.333	383	221	250	284.6667
asd	4989	3923	-4592	-7.67442	7.16E-09	-3.67704	3.677043	1.878546	1.878546	3.45E-08	4588	5601	5651	5280	737	662	665	688
malM	27383	23114	-24888.3	-7.68263	2.06E-08	-3.65827	3.65827	1.871162	1.871162	9.33E-08	26756	31008	28074	28612.67	3906	3625	3642	3724.333
iclR	1231	652	-929.667	-7.68825	8.37E-12	-3.5773	3.577303	1.838872	1.838872	5.87E-11	1354	1055	797	1068.667	123	145	149	139
yecA	8862	6320	-7412	-7.69356	4.01E-09	-3.64172	3.64172	1.86462	1.86462	1.98E-08	9859	8257	7442	8519.333	1203	997	1122	1107.333
aspS	6128	4816	-5430.33	-7.69585	1.2E-07	-3.63773	3.637731	1.863039	-1.86304	4.84E-07	5630	6306	6788	6241.333	814	959	660	811
yqgF	528	441	-483	-7.70833	1.21E-14	-3.66628	3.666275	1.874315	1.874315	1.12E-13	591	559	515	555	74	63	79	72
oppB	9073	5157	-6623.67	-7.71999	2.56E-08	-3.6864	3.686399	1.882212	1.882212	1.14E-07	9744	6894	6190	7609.333	1253	671	1033	985.6667
nrdD	3550	2518	-3000.33	-7.72218	1.05E-08	-3.64845	3.648451	1.867284	1.867284	4.97E-08	2984	3923	3433	3446.667	373	500	466	446.3333
yecF	588	513	-534.667	-7.7395	5.21E-11	-3.73983	3.739831	1.902973	1.902973	3.22E-10	576	619	647	614	116	63	59	79.33333
SEN4299	7160	3657	-5271.33	-7.7668	1.34E-08	-3.65826	3.658255	1.871156	1.871156	6.25E-08	7816	5954	4381	6050.333	957	656	724	779
rnk	434	306	-367.667	-7.76687	5.46E-14	-3.69366	3.693657	1.88505	1.88505	4.83E-13	477	434	355	422	71	43	49	54.33333
hns	21874	19625	-20656.7	-7.80167	6.88E-09	-3.69893	3.698928	1.887107	1.887107	3.33E-08	24768	23716	22597	23693.67	3245	2972	2894	3037
dnaB	1061	947	-982	-7.81944	4.36E-12	-3.70503	3.705033	1.889487	1.889487	3.16E-11	1093	1177	1108	1126	116	146	170	144
fliE	79	31	-52.3333	-7.82609	1.16E-05	-3.77829	3.778289	1.917733	-1.91773	3.39E-05	39	57	84	60	10	8	5	7.666667
yfgB	918	763	-832	-7.83836	7.19E-14	-3.73251	3.732509	1.900146	1.900146	6.31E-13	1025	966	870	953.6667	151	107	107	121.6667
ddg	1189	913	-969.667	-7.86085	9.23E-05	-3.95968	3.959683	1.985385	1.985385	0.000236	1106	1006	1221	1111	299	32	93	141.3333
flhD	2232	1657	-1893	-7.86699	4.81E-09	-3.84495	3.844954	1.942966	1.942966	2.35E-08	2381	1912	2213	2168.667	423	149	255	275.6667
SEN1428	529	419	-444.333	-7.87113	2.52E-11	-3.80469	3.804688	1.927778	1.927778	1.63E-10	567	476	484	509	99	38	57	64.66667
tgt	2023	1502	-1739.33	-7.87484	9.9E-11	-3.75836	3.758364	1.910105	1.910105	5.93E-10	1763	1957	2257	1992.333	234	264	261	253
SEN3500	677	531	-565.667	-7.95492	4.42E-12	-3.84402	3.844021	1.942616	1.942616	3.2E-11	605	733	603	647	116	56	72	81.33333
SEN3609	1160	861	-983.667	-7.95991	1.04E-10	-3.88854	3.888541	1.959229	1.959229	6.23E-10	989	1247	1139	1125	209	87	128	141.3333
SEN2484	802	558	-682.333	-7.96259	4.9E-14	-3.79713	3.797127	1.924908	1.924908	4.35E-13	887	659	795	780.3333	108	85	101	98
sapD	518	369	-427.333	-7.96739	1.56E-12	-3.81628	3.816279	1.932167	1.932167	1.19E-11	425	568	473	488.6667	78	56	50	61.33333
mppA	6888	5973	-6444.33	-7.9769	2.11E-09	-3.83218	3.832179	1.938165	-1.93817	1.07E-08	6931	7664	7509	7368	958	776	1037	923.6667
fliI	877	499	-649.333	-7.98208	5.56E-12	-3.82228	3.822278	1.934433	-1.93443	3.99E-11	589	961	677	742.3333	105	84	90	93
SEN4217	37425	10179	-24240	-7.99567	9.34E-05	-3.89785	3.897847	1.962677	1.962677	0.000238	39422	12276	31417	27705	6301	1997	2097	3465
pykA	24529	16911	-21248	-8.01331	5.71E-08	-3.80755	3.807549	1.928863	-1.92886	2.41E-07	19815	27372	25646	24277.67	2904	3342	2843	3029.667
SEN0989	9506	5594	-6798	-8.02031	1.82E-06	-3.95001	3.950008	1.981856	1.981856	6.09E-06	6376	9999	6924	7766.333	1630	493	782	968.3333
gudD	22578	1888	-10954.7	-8.03575	0.001231	-3.87201	3.872009	1.953082	1.953082	0.002545	23228	3000	11307	12511.67	2909	650	1112	1557
fliH	822	537	-640.333	-8.03663	2.64E-11	-3.80152	3.80152	1.926576	-1.92658	1.7E-10	633	892	669	731.3333	70	96	107	91

Feature ID	Experiment - Range (original values)	Experiment - IQR (original values)	Experiment - Difference (original values)	Experiment - Fold Change (original values)	EDGE test: WT H202 vs WT NT , tagwise dispersions - P-value	EDGE test: WT H202 vs WT NT , tagwise dispersions - Fold change	WT H202 vs WT NT ABS|FC|	WT H202 vs WT NT Log2FC	WT H202 vs WT NT Log2FC +-	EDGE test: WT H202 vs WT NT , tagwise dispersions - FDR p-value correction	WT NT - WT.1.S22 - Expression values	WT NT - WT.2.S23 - Expression values	WT NT - WT.3.S24 - Expression values	WT NT - Means	WT H202 - WT.1.H2O2 - Expression values	WT H202 - WT.2.H2O2 - Expression values	WT H202 - WT.3.H2O2 - Expression values	WT H202 - Means
ucpA	13207	9108	-10763.3	-8.0456	4.27E-08	-3.89572	3.895719	1.96189	1.96189	1.84E-07	14084	12136	10653	12291	2161	877	1545	1527.667
SEN2428	742	348	-578	-8.04878	1.11E-09	-3.89332	3.893317	1.961	1.961	5.84E-09	430	729	821	660	79	82	85	82
yhbS	7483	5766	-6596.33	-8.0549	1.38E-09	-3.83987	3.839873	1.941059	1.941059	7.17E-09	7602	8278	6714	7531.333	948	795	1062	935
SEN4213	7566	1286	-4755.33	-8.06587	0.000224	-3.96769	3.967692	1.9883	1.9883	0.000537	7879	1769	6637	5428.333	1223	313	483	673
yeaZ	602	478	-544.667	-8.07359	4.71E-15	-3.82255	3.822546	1.934534	1.934534	4.61E-14	675	638	552	621.6667	84	73	74	77
aroG	3118	1800	-2358	-8.074	1.72E-11	-3.79272	3.792718	1.923232	1.923232	1.15E-10	3419	2545	2110	2691.333	389	310	301	333.3333
napH	3627	437	-2075.67	-8.07614	0.00013	-3.84325	3.84325	1.942327	-1.94233	0.000325	773	3800	2534	2369	173	371	336	293.3333
SEN2550	808	421	-595.333	-8.0873	1.31E-12	-3.76045	3.760454	1.910907	1.910907	1.02E-11	886	646	506	679.3333	78	89	85	84
lolB	791	605	-701.667	-8.08754	6.37E-15	-3.86765	3.867649	1.951457	1.951457	6.15E-14	865	827	710	800.6667	118	74	105	99
SEN0311	229	111	-184	-8.16883	9.62E-12	-3.84787	3.847872	1.944061	1.944061	6.65E-11	241	249	139	209.6667	29	20	28	25.66667
fis	361	267	-322.667	-8.17037	4.25E-13	-3.93358	3.933581	1.975843	1.975843	3.51E-12	308	394	401	367.6667	54	40	41	45
rplJ	44530	18124	-30181.3	-8.17123	2.59E-06	-3.86164	3.861638	1.949213	1.949213	8.42E-06	22970	47368	32832	34390	2838	4846	4942	4208.667
phsA	10735	8447	-9202.33	-8.19494	1.25E-08	-3.93518	3.935178	1.976429	-1.97643	5.83E-08	11731	9472	10241	10481.33	1816	1025	996	1279
rna-AM93	637	413	-525.333	-8.19635	4.86E-15	-3.88863	3.888632	1.959263	1.959263	4.72E-14	695	608	492	598.3333	82	58	79	73
yffB	608	550	-583.667	-8.20576	8.96E-15	-3.92474	3.924737	1.972596	1.972596	8.45E-14	634	681	679	664.6667	84	73	86	81
SEN3422	996	109	-416	-8.21387	0.000188	-3.72657	3.726575	1.89785	1.89785	0.000456	1043	165	213	473.6667	70	47	56	57.66667
SEN1500	1252	623	-878.333	-8.23901	3.77E-11	-3.91335	3.913353	1.968405	1.968405	2.38E-10	1335	937	727	999.6667	177	83	104	121.3333
queA	462	326	-396.333	-8.25	3.22E-15	-3.92044	3.920443	1.971017	-1.97102	3.22E-14	506	471	376	451	70	44	50	54.66667
cytR	4094	2226	-2857	-8.26356	8.24E-10	-3.99316	3.993162	1.997532	-1.99753	4.47E-09	2730	4362	2659	3250.333	479	268	433	393.3333
fliO	430	180	-296.333	-8.28689	1.49E-09	-3.95195	3.951947	1.982564	-1.98256	7.7E-09	220	468	323	337	38	44	40	40.66667
ybjX	2961	2346	-2560.67	-8.29535	1.67E-12	-3.9542	3.954205	1.983388	1.983388	1.27E-11	3257	2785	2693	2911.667	410	296	347	351
SEN2804	4357	771	-2627.33	-8.31169	1.56E-05	-3.99352	3.993521	1.997661	1.997661	4.51E-05	4605	1043	3312	2986.667	558	248	272	359.3333
SEN2454	248	212	-229.667	-8.32979	2.06E-15	-3.9668	3.966799	1.987975	1.987975	2.11E-14	274	265	244	261	36	26	32	31.33333
rpsG	6146	3291	-4898	-8.36541	2.02E-08	-4.00642	4.006424	2.002315	2.002315	9.17E-08	3970	6763	5956	5563	617	679	699	665
phnA	484	333	-420	-8.36842	3.04E-10	-3.95235	3.952348	1.98271	1.98271	1.73E-09	382	518	531	477	47	75	49	57
ttrS	4924	3580	-4240.67	-8.37935	7.74E-11	-3.97902	3.979016	1.992412	1.992412	4.71E-10	5419	4869	4158	4815.333	651	495	578	574.6667
ybfM	2125	1828	-1889.33	-8.38021	1.96E-07	-4.10804	4.108036	2.038449	2.038449	7.7E-07	2256	2189	1991	2145.333	474	131	163	256
rplS	10584	4478	-6791	-8.38152	3.34E-06	-3.89658	3.896585	1.96221	1.96221	1.07E-05	5347	11221	6565	7711	637	1254	869	920
sdaC	9350	4537	-7086.67	-8.38194	1.88E-07	-4.03705	4.037046	2.0133	2.0133	7.41E-07	5585	8470	10085	8046.667	1097	1048	735	960
glpT	2699	2061	-2395.67	-8.41692	1.01E-11	-4.00389	4.003894	2.001404	2.001404	6.93E-11	2386	3010	2760	2718.667	311	333	325	323
pcnB	898	856	-863.333	-8.44253	2.93E-15	-4.04558	4.045583	2.016348	2.016348	2.94E-14	967	997	974	979.3333	138	99	111	116
yhfA	2963	2661	-2732.67	-8.44596	7.68E-12	-4.07296	4.072959	2.026077	2.026077	5.41E-11	3010	3246	3043	3099.667	469	283	349	367
yjeA	783	715	-740	-8.44966	3.19E-15	-4.06145	4.061452	2.021995	2.021995	3.19E-14	858	849	811	839.3333	127	75	96	99.33333
rpiA	3351	2465	-2990.33	-8.451	3.97E-11	-4.06951	4.069509	2.024855	2.024855	2.49E-10	2855	3603	3717	3391.667	448	366	390	401.3333
rna-AM93	300	173	-243.667	-8.45918	7.47E-13	-3.94479	3.944786	1.979947	1.979947	5.95E-12	327	296	206	276.3333	33	38	27	32.66667
mioC	1302	1092	-1154.33	-8.47948	5.98E-14	-4.08892	4.08892	2.03172	-2.03172	5.28E-13	1268	1415	1243	1308.667	199	113	151	154.3333
argS	2493	1262	-1857.33	-8.50943	3.57E-11	-3.99368	3.993676	1.997717	-1.99772	2.26E-10	2710	1524	2080	2104.667	262	263	217	247.3333

Feature ID	Experiment - Range (original values)	Experiment - IQR (original values)	Experiment - Difference (original values)	Experiment - Fold Change (original values)	EDGE test: WT H202 vs WT NT , tagwise dispersions - P-value	EDGE test: WT H202 vs WT NT , tagwise dispersions - Fold change	WT H202 vs WT NT ABS|FC|	WT H202 vs WT NT Log2FC	WT H202 vs WT NT Log2FC +-	EDGE test: WT H202 vs WT NT , tagwise dispersions - FDR p-value correction	WT NT - WT.1.S22 - Expression values	WT NT - WT.2.S23 - Expression values	WT NT - WT.3.S24 - Expression values	WT NT - Means	WT H202 - WT.1.H2O2 - Expression values	WT H202 - WT.2.H2O2 - Expression values	WT H202 - WT.3.H2O2 - Expression values	WT H202 - Means
lonH	964	895	-922	-8.5163	2.44E-13	-4.0017	4.001696	2.000612	2.000612	2.04E-12	1051	1075	1008	1044.667	113	144	111	122.6667
oppC	5955	3822	-4789.67	-8.53487	1.12E-10	-4.04418	4.044178	2.015846	2.015846	6.68E-10	6489	5331	4456	5425.333	739	534	634	635.6667
guaB	872	372	-586.333	-8.5819	8.54E-10	-3.91952	3.91952	1.970677	1.970677	4.62E-09	930	616	445	663.6667	73	101	58	77.33333
yhdH	5765	3488	-4938.67	-8.5824	1.38E-08	-4.10922	4.109216	2.038863	2.038863	6.43E-08	4188	6302	6280	5590	717	700	537	651.3333
selB	6653	5051	-5637	-8.59362	1.37E-09	-4.07538	4.075383	2.026936	2.026936	7.16E-09	5797	7312	6029	6379.333	659	746	822	742.3333
ilvB	3944	1582	-2466.67	-8.63674	1.99E-10	-4.02672	4.026717	2.009604	-2.0096	1.16E-09	4221	2231	1917	2789.667	357	277	335	323
rpmB	6205	3513	-4867.67	-8.68175	2.73E-08	-4.11028	4.110279	2.039236	2.039236	1.21E-07	4213	6688	5603	5501.333	483	718	700	633.6667
SEN2998	516	237	-353.667	-8.68841	2.56E-11	-4.13318	4.133179	2.047252	2.047252	1.65E-10	541	376	282	399.6667	68	25	45	46
SEN2935	203	158	-185	-8.70833	1.36E-14	-4.10509	4.105094	2.037415	2.037415	1.26E-13	225	220	182	209	24	26	22	24
rpmG	3781	1791	-3049.33	-8.71332	6.95E-08	-4.16264	4.162642	2.0575	2.0575	2.89E-07	2237	4041	4056	3444.667	275	446	465	395.3333
psd	1002	981	-974	-8.73016	2.07E-15	-4.19129	4.191288	2.067394	-2.06739	2.11E-14	1097	1106	1097	1100	158	104	116	126
ycfW	568	544	-554	-8.73023	4.13E-15	-4.13223	4.13223	2.046921	2.046921	4.07E-14	633	627	617	625.6667	73	77	65	71.66667
tufA	30310	21107	-25507.7	-8.75781	2.06E-08	-4.12976	4.12976	2.046058	2.046058	9.34E-08	24246	33177	28964	28795.67	2867	3858	3139	3288
gntR	1496	1079	-1251	-8.80249	1.33E-11	-4.23556	4.235558	2.082552	2.082552	9.01E-11	1603	1425	1206	1411.333	247	107	127	160.3333
SEN2263	462	421	-440	-8.81065	9.67E-16	-4.18332	4.183322	2.064649	2.064649	1.03E-14	476	517	496	496.3333	55	59	55	56.33333
ycbW	548	331	-407.333	-8.83333	1.02E-13	-4.16169	4.161685	2.057168	2.057168	8.76E-13	586	413	379	459.3333	70	48	38	52
SEN4270	576	380	-454.667	-8.83908	8.64E-16	-4.17277	4.172771	2.061006	2.061006	9.25E-15	625	480	433	512.6667	72	53	49	58
nrfC	2857	727	-1890.67	-8.85596	9.51E-08	-4.33193	4.331931	2.11501	2.11501	3.86E-07	981	3054	2359	2131.333	254	197	271	240.6667
SEN3651	2759	2059	-2411.67	-8.88126	1.46E-12	-4.27554	4.275544	2.096108	2.096108	1.12E-11	2831	2972	2350	2717.667	414	213	291	306
avtA	868	737	-810.667	-8.8961	1.04E-16	-4.22349	4.223494	2.078437	-2.07844	1.19E-15	940	965	835	913.3333	113	97	98	102.6667
pssA	4703	4278	-4400	-8.90893	2.51E-11	-4.28252	4.282517	2.098459	2.098459	1.63E-10	4842	5154	4873	4956.333	654	451	564	556.3333
lpxH	426	404	-412.667	-8.9359	5.26E-17	-4.24272	4.242715	2.084988	2.084988	6.15E-16	475	464	455	464.6667	49	51	56	52
napC	4363	483	-2926.33	-8.9592	0.000127	-4.35419	4.354187	2.122403	2.122403	0.000316	893	4431	4558	3294	195	410	498	367.6667
SEN0708	906	777	-798.667	-8.96013	1.03E-11	-4.33045	4.330447	2.114516	2.114516	7.04E-11	975	848	874	899	161	69	71	100.3333
trmD	17352	7452	-11027.3	-8.97541	3.09E-06	-4.15258	4.15258	2.054008	2.054008	9.94E-06	8788	18216	10226	12410	864	1948	1336	1382.667
fliP	684	373	-504	-9.04255	3.11E-12	-4.37878	4.378782	2.13053	-2.13053	2.29E-11	428	731	541	566.6667	86	47	55	62.66667
ompW	48184	30231	-39442.3	-9.04781	2.77E-09	-4.37299	4.372987	2.128619	2.128619	1.39E-08	46047	34709	52274	44343.33	6135	4090	4478	4901
nanT	22085	6847	-14427.3	-9.05396	2.46E-07	-4.33504	4.335036	2.116044	-2.11604	9.54E-07	16892	23150	8614	16218.67	2542	1065	1767	1791.333
htrB	969	662	-803.667	-9.0906	1.64E-13	-4.31938	4.319376	2.110823	-2.11082	1.39E-12	1040	923	746	903	143	84	71	99.33333
yneC	1481	44	-510.333	-9.10053	0.007051	-4.07326	4.073259	2.026184	2.026184	0.012503	1522	108	90	573.3333	96	41	52	63
rhlB	4749	4499	-4618	-9.12075	3.97E-11	-4.34049	4.340486	2.117857	-2.11786	2.49E-10	5065	5231	5264	5186.667	625	566	515	568.6667
SEN3982	197	116	-165.333	-9.13115	6.91E-12	-4.35171	4.351711	2.121583	2.121583	4.9E-11	207	136	214	185.6667	24	20	17	20.33333
dcuB	23044	12206	-18146	-9.15917	2.36E-08	-4.31626	4.316259	2.109781	-2.10978	1.06E-07	24612	14661	21837	20370	2649	2455	1568	2224
rna-AM93	828	556	-717.667	-9.18631	1.3E-15	-4.38725	4.387255	2.133318	2.133318	1.36E-14	890	884	642	805.3333	115	62	86	87.66667
mviM	3519	2472	-3014	-9.21253	2.25E-12	-4.32323	4.323232	2.11211	2.11211	1.68E-11	3829	3475	2839	3381	310	367	424	367
yddG	122	110	-112.333	-9.21951	5.24E-13	-4.37734	4.377344	2.130056	2.130056	4.27E-12	133	122	123	126	12	11	18	13.66667
ybhK	1236	725	-994.667	-9.22039	7.27E-16	-4.32006	4.320064	2.111053	2.111053	7.81E-15	1349	1154	844	1115.667	131	119	113	121

Feature ID	Experiment - Range (original values)	Experiment - IQR (original values)	Experiment - Difference (original values)	Experiment - Fold Change (original values)	EDGE test: WT H202 vs WT NT , tagwise dispersions - P-value	EDGE test: WT H202 vs WT NT , tagwise dispersions - Fold change	WT H202 vs WT NT ABS|FC|	WT H202 vs WT NT Log2FC	WT H202 vs WT NT Log2FC +-	EDGE test: WT H202 vs WT NT , tagwise dispersions - FDR p-value correction	WT NT - WT.1.S22 - Expression values	WT NT - WT.2.S23 - Expression values	WT NT - WT.3.S24 - Expression values	WT NT - Means	WT H202 - WT.1.H2O2 - Expression values	WT H202 - WT.2.H2O2 - Expression values	WT H202 - WT.3.H2O2 - Expression values	WT H202 - Means
glpF	35954	30280	-30776.3	-9.24882	4.4E-06	-4.60118	4.601181	2.202004	2.202004	1.39E-05	33013	37322	33187	34507.33	7092	1368	2733	3731
aphA	2646	1647	-2083	-9.26587	1.02E-12	-4.48077	4.48077	2.163747	-2.16375	8E-12	2262	1876	2867	2335	306	221	229	252
rna-AM93	511	465	-488	-9.27119	2.18E-18	-4.41931	4.419308	2.14382	2.14382	2.91E-17	563	553	525	547	65	52	60	59
rna-AM93	1017	915	-973.333	-9.27195	1.33E-17	-4.42496	4.424965	2.145666	2.145666	1.66E-16	1118	1116	1039	1091	124	101	128	117.6667
atpH	9967	4339	-7253.33	-9.28321	3.89E-08	-4.4871	4.487103	2.165784	2.165784	1.68E-07	5254	8414	10719	8129	752	915	960	875.6667
SEN2551	503	279	-405.333	-9.38621	8.39E-15	-4.36697	4.366971	2.126633	2.126633	7.93E-14	545	489	327	453.6667	48	55	42	48.33333
fklB	818	609	-725	-9.43023	4.42E-13	-4.55052	4.550515	2.18603	2.18603	3.64E-12	686	868	879	811	120	77	61	86
ydgH	4664	3905	-4251	-9.43452	1.33E-11	-4.53693	4.536929	2.181716	2.181716	9.01E-11	4373	4777	5115	4755	593	451	468	504
napA	26188	3629	-14577.3	-9.43596	7.8E-05	-4.47078	4.470783	2.160528	2.160528	0.000202	5790	27043	16083	16305.33	855	2161	2168	1728
galF	3363	2610	-2975	-9.44371	1.95E-13	-4.53816	4.538159	2.182107	-2.18211	1.65E-12	2959	3669	3354	3327.333	402	306	349	352.3333
fadH	2300	1045	-1800.67	-9.46708	6.65E-13	-4.45485	4.454851	2.155377	-2.15538	5.33E-12	2292	2478	1270	2013.333	225	178	235	212.6667
minC	2402	2086	-2280.67	-9.47831	1.13E-14	-4.53584	4.535836	2.181368	2.181368	1.06E-13	2357	2639	2653	2549.667	285	251	271	269
ychK	2774	2430	-2576	-9.49231	7.99E-15	-4.54584	4.545842	2.184548	2.184548	7.58E-14	3016	2869	2753	2879.333	323	242	345	303.3333
malK	33522	28484	-30531	-9.5013	1.43E-09	-4.45687	4.456872	2.156032	2.156032	7.4E-09	33686	36459	32222	34122.33	2937	4099	3738	3591.333
rna-AM93	481	331	-408.333	-9.56643	2.86E-11	-4.63559	4.635592	2.212754	2.212754	1.82E-10	502	493	373	456	80	21	42	47.66667
SEN2977	7242	2728	-4639.67	-9.58667	1.56E-08	-4.62404	4.624041	2.209154	2.209154	7.23E-08	4702	7562	3276	5180	753	320	548	540.3333
SEN4182	528	56	-266.333	-9.5914	4.59E-06	-4.45058	4.450577	2.153992	2.153992	1.44E-05	554	88	250	297.3333	35	26	32	31
cydA	67490	53119	-60508.3	-9.64981	6.7E-10	-4.63042	4.630421	2.211143	-2.21114	3.68E-09	73028	59157	70326	67503.67	9410	6038	5538	6995.333
rpsL	4494	2396	-3459	-9.66194	5.75E-11	-4.65207	4.652071	2.217873	2.217873	3.54E-10	2803	4857	3915	3858.333	428	363	407	399.3333
sapB	259	236	-245.667	-9.67059	8.13E-17	-4.58375	4.583754	2.19653	2.19653	9.41E-16	272	286	264	274	28	30	27	28.33333
gcvH	15913	14431	-15189	-9.67612	1.9E-11	-4.6195	4.619496	2.207735	-2.20774	1.26E-10	16152	17537	17130	16939.67	1721	1624	1907	1750.667
rph	328	260	-294	-9.73267	2.08E-17	-4.61208	4.612076	2.205416	2.205416	2.54E-16	335	357	291	327.6667	29	31	41	33.66667
SEN2916	576	306	-444.333	-9.76974	1.07E-10	-4.77479	4.774792	2.255438	2.255438	6.37E-10	348	528	609	495	77	42	33	50.66667
SEN0992	587	376	-490.667	-9.81437	1.92E-08	-4.86306	4.863061	2.281865	2.281865	8.79E-08	407	615	617	546.3333	106	30	31	55.66667
ygcB	1423	1006	-1127.67	-9.8329	2.71E-13	-4.75302	4.753019	2.248844	2.248844	2.26E-12	1146	1513	1107	1255.333	192	90	101	127.6667
pepT	12370	7915	-10563.7	-9.87206	2.2E-09	-4.67811	4.678114	2.225927	2.225927	1.11E-08	9084	13375	12804	11754.33	1005	1398	1169	1190.667
yceH	4381	3180	-3747	-9.92143	1.94E-12	-4.65442	4.654416	2.2186	2.2186	1.46E-11	4745	4146	3610	4167	364	430	466	420
tsx	17993	4183	-8919.33	-9.93422	4.12E-06	-4.6757	4.675698	2.225182	2.225182	1.3E-05	5223	18877	5653	9917.667	884	1071	1040	998.3333
pgtC	841	403	-572.333	-9.98953	8.69E-11	-4.77427	4.774266	2.255279	-2.25528	5.27E-10	875	580	453	636	107	34	50	63.66667
pyrD	313	283	-293.667	-9.9898	9.6E-13	-4.69108	4.69108	2.22992	2.22992	7.56E-12	329	318	332	326.3333	35	44	19	32.66667
pyrH	942	582	-758.667	-9.99605	3.37E-15	-4.85132	4.851321	2.278378	-2.27838	3.35E-14	671	846	1012	843	89	70	94	84.33333
rluC	342	295	-313	-10.0288	8.58E-19	-4.75915	4.759152	2.250705	2.250705	1.2E-17	374	329	340	347.6667	38	34	32	34.66667
cutA	578	393	-473.667	-10.051	3.07E-19	-4.73027	4.730274	2.241924	2.241924	4.52E-18	629	504	445	526	54	51	52	52.33333
SEN1204	285	239	-247.667	-10.061	2.34E-14	-4.84562	4.845616	2.27668	2.27668	2.1E-13	260	303	262	275	43	18	21	27.33333
cobD	742	610	-686	-10.0661	2.8E-17	-4.83704	4.837036	2.274123	-2.27412	3.37E-16	679	809	797	761.6667	91	67	69	75.66667
yidE	3280	1690	-2358.67	-10.0718	8.46E-13	-4.76599	4.765986	2.252775	2.252775	6.69E-12	3480	2474	1902	2618.667	368	200	212	260
frdB	39730	36452	-37056.7	-10.174	8.57E-10	-4.92725	4.927252	2.300783	-2.30078	4.62E-09	39627	40988	42673	41096	6000	3175	2943	4039.333

Feature ID	Experiment - Range (original values)	Experiment - IQR (original values)	Experiment - Difference (original values)	Experiment - Fold Change (original values)	EDGE test: WT H202 vs WT NT , tagwise dispersions - P-value	EDGE test: WT H202 vs WT NT , tagwise dispersions - Fold change	WT H202 vs WT NT ABS|FC|	WT H202 vs WT NT Log2FC	WT H202 vs WT NT Log2FC +-	EDGE test: WT H202 vs WT NT , tagwise dispersions - FDR p-value correction	WT NT - WT.1.S22 - Expression values	WT NT - WT.2.S23 - Expression values	WT NT - WT.3.S24 - Expression values	WT NT - Means	WT H202 - WT.1.H2O2 - Expression values	WT H202 - WT.2.H2O2 - Expression values	WT H202 - WT.3.H2O2 - Expression values	WT H202 - Means
ydiA	1615	845	-1142.67	-10.1903	1.58E-16	-4.79332	4.793325	2.261027	2.261027	1.77E-15	1719	1113	969	1267	145	104	124	124.3333
SEN3975	400	310	-350	-10.2105	8.39E-17	-4.9433	4.943302	2.305475	2.305475	9.68E-16	350	388	426	388	48	26	40	38
yfcZ	4104	3425	-3721.33	-10.2417	1.11E-13	-4.92136	4.921361	2.299057	2.299057	9.57E-13	4115	4415	3842	4124	480	311	417	402.6667
leuL	17	6	-12.3333	-10.25	0.001001	-4.62316	4.623158	2.208879	2.208879	0.002107	16	17	8	13.66667	2	2	0	1.333333
ybhQ	1847	1483	-1583	-10.2573	5.08E-09	-5.04015	5.040145	2.333465	2.333465	2.48E-08	1920	1735	1607	1754	316	73	124	171
yciI	1337	1087	-1208.67	-10.2737	1.15E-17	-4.86977	4.869767	2.283853	2.283853	1.44E-16	1457	1218	1342	1339	140	131	120	130.3333
yhcH	4228	1857	-2664	-10.293	1.9E-10	-4.99305	4.993045	2.31992	2.31992	1.11E-09	2292	4402	2158	2950.667	385	174	301	286.6667
rna-AM93	360	256	-288.333	-10.3011	2.76E-17	-4.91297	4.912975	2.296597	-2.2966	3.33E-16	381	285	292	319.3333	43	21	29	31
pepE	2233	1607	-1918	-10.3409	4.22E-15	-4.92402	4.924019	2.299836	2.299836	4.14E-14	1817	2428	2125	2123.333	210	211	195	205.3333
SEN3597	1762	1139	-1408.67	-10.3496	1.78E-15	-4.94598	4.945977	2.306255	2.306255	1.83E-14	1290	1903	1485	1559.333	151	141	160	150.6667
rimM	16584	7634	-11091.7	-10.3891	2.55E-07	-4.81288	4.812879	2.2669	2.2669	9.85E-07	8784	17307	10728	12273	723	1671	1150	1181.333
yfiF	1563	1183	-1403	-10.3951	4.18E-15	-4.94359	4.943591	2.305559	2.305559	4.11E-14	1327	1706	1624	1552.333	144	161	143	149.3333
pspE	1610	1255	-1365.67	-10.3968	4.04E-16	-4.99428	4.994277	2.320276	2.320276	4.41E-15	1381	1731	1421	1511	189	121	126	145.3333
fliQ	178	114	-135	-10.4186	1.1E-12	-4.99721	4.99721	2.321123	-2.32112	8.6E-12	131	185	132	149.3333	17	7	19	14.33333
yebK	2352	1847	-2066.67	-10.4368	0	-4.97218	4.972182	2.313879	2.313879	0	2251	2543	2063	2285.667	216	191	250	219
ycfU	584	474	-514	-10.4601	3.08E-20	-4.95509	4.955088	2.308911	2.308911	4.96E-19	636	529	540	568.3333	56	52	55	54.33333
SEN1065	488	370	-430	-10.4853	1.1E-18	-4.97317	4.973168	2.314165	2.314165	1.52E-17	489	524	413	475.3333	57	43	36	45.33333
yhaO	6013	3099	-4152.67	-10.4954	3.8E-08	-5.17415	5.174147	2.371321	2.371321	1.64E-07	3416	6247	4107	4590	761	234	317	437.3333
ycbC	1231	850	-1021.67	-10.5186	1.13E-19	-4.96219	4.962193	2.310978	2.310978	1.73E-18	1331	1101	955	1129	117	100	105	107.3333
rpsP	4181	2191	-2834	-10.5421	8E-10	-4.89945	4.899447	2.292619	2.292619	4.36E-09	2470	4404	2519	3131	223	389	279	297
glpC	15960	11755	-13516	-10.5632	2.9E-11	-5.0836	5.083602	2.345851	-2.34585	1.84E-10	16935	14877	12976	14929.33	2044	975	1221	1413.333
rna-AM93	559	327	-405	-10.5669	5.44E-08	-5.1685	5.168495	2.369744	-2.36974	2.3E-07	569	411	362	447.3333	82	10	35	42.33333
malT	14645	10887	-12676	-10.6055	5.14E-11	-5.13069	5.130688	2.359152	-2.35915	3.18E-10	15455	14401	12131	13995.67	1905	810	1244	1319.667
SEN2999	575	280	-418.333	-10.6538	1.17E-15	-5.04432	5.044318	2.334659	2.334659	1.22E-14	603	461	321	461.6667	61	28	41	43.33333
kdgT	2174	25	-869.333	-10.6593	0.000732	-5.05872	5.058722	2.338773	2.338773	0.001588	545	2229	104	959.3333	95	55	120	90
rpmA	7735	4647	-6261.67	-10.6631	1.24E-10	-5.06376	5.063755	2.340208	2.340208	7.29E-10	5307	8302	7120	6909.667	567	717	660	648
ansB	50420	40861	-45236.7	-10.668	5.87E-10	-5.22646	5.22646	2.385834	-2.38583	3.25E-09	44853	53349	51545	49915.67	7116	2929	3992	4679
SEN1181	78	18	-45.3333	-10.7143	5.25E-06	-4.99091	4.990915	2.319304	2.319304	1.63E-05	81	22	47	50	7	3	4	4.666667
dctA	9290	4817	-7547.33	-10.7427	3.35E-10	-5.28805	5.288049	2.402736	2.402736	1.91E-09	5636	9812	9518	8322	983	522	819	774.6667
eutT	1249	143	-510.333	-10.7516	3.47E-05	-4.88269	4.882695	2.287678	2.287678	9.48E-05	1285	210	193	562.6667	71	36	50	52.33333
yhfK	3185	2502	-2736.67	-10.7855	1.55E-15	-5.17729	5.17729	2.372197	2.372197	1.61E-14	2879	3412	2758	3016.333	356	227	256	279.6667
cbiT	1922	973	-1337.67	-10.9086	2.84E-11	-5.36332	5.363316	2.423125	2.423125	1.81E-10	1321	1075	2022	1472.667	203	102	100	135
udk	638	562	-591.333	-10.9106	6E-19	-5.2436	5.243601	2.390558	2.390558	8.44E-18	650	616	687	651	76	49	54	59.66667
SEN1399	631	369	-486.667	-11	2.16E-18	-5.18857	5.18857	2.375337	2.375337	2.9E-17	671	525	410	535.3333	65	41	40	48.66667
rimJ	4163	3107	-3647.33	-11.0755	4.88E-15	-5.23056	5.230564	2.386967	2.386967	4.74E-14	4498	4078	3452	4009.333	345	335	406	362
yhjJ	3405	2293	-2846.67	-11.1185	8.97E-14	-5.41811	5.418112	2.43779	2.43779	7.78E-13	2542	3214	3628	3128	372	223	249	281.3333
atpE	8959	3796	-6480.33	-11.2537	4.67E-10	-5.4976	5.4976	2.458802	2.458802	2.6E-09	4429	7373	9535	7112.333	633	576	687	632

Feature ID	Experiment - Range (original values)	Experiment - IQR (original values)	Experiment - Difference (original values)	Experiment - Fold Change (original values)	EDGE test: WT H202 vs WT NT , tagwise dispersions - P-value	EDGE test: WT H202 vs WT NT , tagwise dispersions - Fold change	WT H202 vs WT NT ABS|FC|	WT H202 vs WT NT Log2FC	WT H202 vs WT NT Log2FC +-	EDGE test: WT H202 vs WT NT , tagwise dispersions - FDR p-value correction	WT NT - WT.1.S22 - Expression values	WT NT - WT.2.S23 - Expression values	WT NT - WT.3.S24 - Expression values	WT NT - Means	WT H202 - WT.1.H2O2 - Expression values	WT H202 - WT.2.H2O2 - Expression values	WT H202 - WT.3.H2O2 - Expression values	WT H202 - Means
yheO	2691	1412	-2086.33	-11.2607	9.94E-12	-5.58364	5.583638	2.481205	2.481205	6.84E-11	1594	2459	2816	2289.667	303	125	182	203.3333
SEN2359	151	74	-102.667	-11.2667	3.5E-08	-5.36943	5.369427	2.424768	2.424768	1.53E-07	85	98	155	112.6667	11	15	4	10
SEN2819A	572	381	-475	-11.3261	2.2E-15	-5.40376	5.403755	2.433962	2.433962	2.23E-14	544	602	417	521	72	36	30	46
yneB	9581	302	-3315.33	-11.3496	0.003381	-5.11129	5.111285	2.353686	2.353686	0.006405	9764	600	543	3635.667	537	183	241	320.3333
ivbL	701	421	-549.333	-11.3648	2.12E-11	-5.45074	5.450742	2.446453	2.446453	1.39E-10	731	623	453	602.3333	97	32	30	53
rplK	6225	3897	-4670.67	-11.4489	5.06E-11	-5.33709	5.337086	2.416052	2.416052	3.14E-10	4448	6591	4314	5117.667	366	558	417	447
rna-AM93	616	271	-478	-11.4672	1.63E-16	-5.37736	5.377358	2.426897	2.426897	1.82E-15	599	656	316	523.6667	52	40	45	45.66667
ackA	13015	10516	-12039	-11.5205	1.2E-11	-5.43481	5.434807	2.442229	2.442229	8.18E-11	11594	14015	13941	13183.33	1000	1355	1078	1144.333
SEN4074	1167	855	-961	-11.5219	3.53E-11	-5.64609	5.646089	2.497252	2.497252	2.23E-10	1207	924	1026	1052.333	165	40	69	91.33333
rna-AM93	376	230	-305.333	-11.5287	2.26E-18	-5.46321	5.463207	2.449748	2.449748	3.01E-17	349	400	254	334.3333	39	24	24	29
citF	2181	659	-1188.67	-11.5503	2.52E-06	-5.43092	5.430923	2.441197	-2.4412	8.2E-06	2217	952	735	1301.333	226	76	36	112.6667
SEN1303	4878	3396	-4152.67	-11.6297	1.32E-13	-5.64719	5.647189	2.497533	2.497533	1.13E-12	3780	4653	5197	4543.333	469	319	384	390.6667
ydgT	213	164	-184.333	-11.6346	7.73E-15	-5.60199	5.601986	2.485939	2.485939	7.35E-14	204	176	225	201.6667	28	12	12	17.33333
ydhF	2642	2070	-2342.33	-11.6631	0	-5.52931	5.52931	2.467099	2.467099	0	2571	2830	2285	2562	256	215	188	219.6667
accB	2349	1917	-2143.67	-11.6827	2.83E-16	-5.51067	5.510672	2.462228	-2.46223	3.11E-15	2396	2107	2530	2344.333	181	231	190	200.6667
idnT	1165	575	-814.667	-11.7665	2.31E-15	-5.59902	5.599017	2.485174	2.485174	2.34E-14	1211	818	642	890.3333	114	46	67	75.66667
dpiB	2572	730	-1357	-11.7698	7.25E-10	-5.44417	5.444167	2.444711	2.444711	3.98E-09	2672	946	831	1483	177	101	100	126
yfbB	1962	1190	-1540	-11.7944	2.24E-15	-5.61265	5.612654	2.488683	2.488683	2.27E-14	1335	2080	1633	1682.667	118	145	165	142.6667
znuA	1775	1287	-1514	-11.8921	6.98E-20	-5.66752	5.66752	2.502718	2.502718	1.1E-18	1893	1435	1631	1653	148	118	151	139
fliF	550	351	-448	-11.9268	9.13E-15	-5.66569	5.665686	2.502251	-2.50225	8.57E-14	390	495	582	489	32	52	39	41
SEN0629	2830	791	-1600	-11.9589	9.34E-11	-5.7154	5.715404	2.514855	2.514855	5.63E-10	1353	2933	952	1746	174	103	161	146
yhbC	2052	1726	-1831	-11.986	1.17E-17	-5.78459	5.78459	2.532215	2.532215	1.46E-16	1876	2179	1938	1997.667	223	127	150	166.6667
blc	1383	994	-1187.67	-11.9969	1.01E-20	-5.74315	5.743146	2.521841	2.521841	1.69E-19	1320	1464	1103	1295.667	134	81	109	108
SEN2915	526	215	-387.667	-12.0762	3.73E-10	-5.74281	5.742806	2.521756	2.521756	2.11E-09	249	471	548	422.6667	22	49	34	35
flgD	1132	780	-958.667	-12.1042	1.75E-18	-5.75784	5.757839	2.525527	2.525527	2.35E-17	867	1212	1056	1045	80	92	87	86.33333
crp	19382	17918	-18195.7	-12.1198	7.21E-13	-5.89344	5.893444	2.559111	-2.55911	5.75E-12	19549	19446	20501	19832	2262	1119	1528	1636.333
yebA	3227	2084	-2585.33	-12.1277	0	-5.70315	5.703148	2.511758	2.511758	0	3448	2321	2684	2817.667	237	239	221	232.3333
yecH	375	335	-349.333	-12.1489	3.94E-19	-5.73421	5.734208	2.519594	2.519594	5.69E-18	368	399	375	380.6667	24	37	33	31.33333
SEN4302	6238	885	-2824.67	-12.1794	0.001199	-5.91396	5.913956	2.564123	2.564123	0.002483	6273	973	1986	3077.333	635	35	88	252.6667
caiE	1538	912	-1182	-12.2571	1.49E-20	-5.78493	5.784926	2.532299	-2.5323	2.46E-19	1627	1228	1006	1287	132	89	94	105
moaA	5644	3153	-4442.67	-12.3045	6.88E-15	-5.7545	5.7545	2.524691	2.524691	6.57E-14	6016	4960	3531	4835.667	378	372	429	393
atpI	1956	1712	-1791.33	-12.3137	1.01E-16	-5.98874	5.988743	2.582253	2.582253	1.16E-15	1857	2058	1934	1949.667	228	102	145	158.3333
rcsF	479	443	-457.667	-12.3471	2.11E-22	-5.93596	5.935958	2.569481	2.569481	3.95E-21	507	486	501	498	50	28	43	40.33333
srlR	4150	2146	-3176.67	-12.3588	1.24E-14	-5.99496	5.994964	2.583751	2.583751	1.16E-13	2447	4374	3548	3456.333	314	224	301	279.6667
hypC	4792	3486	-3982.33	-12.3673	6.88E-15	-5.86069	5.860689	2.55107	2.55107	6.57E-14	3835	5129	4034	4332.667	349	365	337	350.3333
melB	9226	2646	-4845.33	-12.4008	4.06E-09	-5.79059	5.790595	2.533712	2.533712	2E-08	9510	3076	3225	5270.333	561	284	430	425
SEN4305	20757	1170	-7146	-12.4032	0.010808	-5.79042	5.79042	2.533668	2.533668	0.018454	20854	1088	1376	7772.667	1577	97	206	626.6667

Feature ID	Experiment - Range (original values)	Experiment - IQR (original values)	Experiment - Difference (original values)	Experiment - Fold Change (original values)	EDGE test: WT H202 vs WT NT , tagwise dispersions - P-value	EDGE test: WT H202 vs WT NT , tagwise dispersions - Fold change	WT H202 vs WT NT ABS|FC|	WT H202 vs WT NT Log2FC	WT H202 vs WT NT Log2FC +-	EDGE test: WT H202 vs WT NT , tagwise dispersions - FDR p-value correction	WT NT - WT.1.S22 - Expression values	WT NT - WT.2.S23 - Expression values	WT NT - WT.3.S24 - Expression values	WT NT - Means	WT H202 - WT.1.H2O2 - Expression values	WT H202 - WT.2.H2O2 - Expression values	WT H202 - WT.3.H2O2 - Expression values	WT H202 - Means
SEN0893	4458	2307	-3238.67	-12.4441	0	-5.82825	5.828249	2.543062	2.543062	0	4713	3265	2587	3521.667	314	255	280	283
ycfV	345	304	-317.333	-12.4699	3.96E-20	-5.98164	5.981638	2.580541	2.580541	6.34E-19	341	327	367	345	38	23	22	27.66667
gcvT	27454	25743	-26565	-12.4735	1.67E-14	-5.97043	5.970431	2.577835	2.577835	1.52E-13	29436	29196	28009	28880.33	2698	1982	2266	2315.333
rna-AM93	216	132	-160.667	-12.4762	2.19E-13	-5.99394	5.993945	2.583506	2.583506	1.84E-12	151	147	226	174.6667	17	15	10	14
hybA	5529	4659	-4993.67	-12.4797	6.44E-15	-5.8974	5.897404	2.56008	-2.56008	6.19E-14	5889	5293	5104	5428.667	500	445	360	435
cibB	1797	1557	-1677.33	-12.4886	4.95E-19	-5.93235	5.932348	2.568603	-2.5686	7.03E-18	1917	1704	1849	1823.333	171	147	120	146
rplU	7763	5000	-6748.33	-12.5422	8.32E-13	-5.99954	5.999541	2.584852	2.584852	6.58E-12	5575	8327	8097	7333	575	615	564	584.6667
SEN4300	1552	581	-1072.33	-12.6982	0.000141	-6.41333	6.413333	2.681074	2.681074	0.000349	1561	620	1311	1164	227	9	39	91.66667
rpsT	4068	3279	-3702	-12.8401	1.53E-14	-6.0587	6.0587	2.599008	2.599008	1.4E-13	3570	4350	4124	4014.667	282	365	291	312.6667
cspE	31238	23057	-25760.7	-12.8476	1.89E-12	-6.20759	6.207592	2.634034	-2.63403	1.42E-11	32677	24888	26240	27935	3253	1439	1831	2174.333
SEN4303	10666	938	-4221	-12.9575	0.003334	-6.19349	6.193487	2.630752	2.630752	0.006322	10708	1048	1966	4574	907	42	110	353
ysaA	1467	1140	-1347.33	-12.9586	1.37E-21	-6.1072	6.1072	2.610511	2.610511	2.41E-20	1568	1561	1251	1460	101	111	126	112.6667
fliK	2357	1358	-1762.33	-13.0708	1.1E-16	-6.19617	6.196167	2.631376	-2.63138	1.25E-15	1502	2494	1729	1908.333	144	157	137	146
yaiZ	1118	914	-1007.67	-13.1406	3.47E-17	-6.35802	6.358022	2.668578	2.668578	4.14E-16	1168	1125	979	1090.667	134	50	65	83
cbiE	1865	1180	-1424.67	-13.142	4.89E-13	-6.47183	6.471826	2.694173	-2.69417	4E-12	1424	1260	1942	1542	195	77	80	117.3333
SEN1443	109	29	-57	-13.2143	5.7E-07	-5.98267	5.982666	2.580788	2.580788	2.1E-06	112	34	39	61.66667	5	6	3	4.666667
mglA	1169	636	-824	-13.2376	3.88E-18	-6.35054	6.350542	2.66688	2.66688	5.02E-17	697	1225	752	891.3333	85	56	61	67.33333
yhcM	1331	1273	-1285.33	-13.3987	4.75E-22	-6.39224	6.392242	2.676322	2.676322	8.73E-21	1375	1373	1419	1389	123	100	88	103.6667
garR	31075	5098	-18164	-13.4184	6.97E-06	-6.59839	6.598385	2.722113	-2.72211	2.14E-05	31555	6171	21154	19626.67	2835	480	1073	1462.667
ompF	7897	3666	-6064.33	-13.4866	5.25E-11	-6.41036	6.41036	2.680405	2.680405	3.24E-10	4180	8266	7204	6550	369	574	514	485.6667
atpB	6626	4161	-5443	-13.5415	1.42E-14	-6.58488	6.584882	2.719158	2.719158	1.3E-13	4594	6046	6991	5877	504	365	433	434
SEN1786	1455	1049	-1287.33	-13.6209	7.95E-18	-6.54804	6.54804	2.711063	2.711063	1E-16	1522	1517	1129	1389.333	159	67	80	102
fliN	1080	575	-825	-13.6923	2.6E-18	-6.55454	6.554543	2.712495	2.712495	3.45E-17	639	1139	892	890	59	64	72	65
SEN2269	517	374	-429.667	-13.7624	9.6E-17	-6.70359	6.703594	2.744935	2.744935	1.11E-15	401	535	454	463.3333	56	18	27	33.66667
yidF	1552	1103	-1370.33	-13.7671	1.17E-23	-6.53647	6.536465	2.708511	2.708511	2.5E-22	1577	1643	1213	1477.667	121	91	110	107.3333
SEN4191	131	83	-98	-13.7826	6.41E-13	-6.5102	6.510198	2.702701	2.702701	5.15E-12	90	135	92	105.6667	4	7	12	7.666667
cspB	1535	521	-964	-13.8533	4.84E-12	-6.57448	6.57448	2.716877	-2.71688	3.49E-11	1573	964	580	1039	128	38	59	75
yjjW	2863	1963	-2322.67	-13.8561	4.83E-19	-6.55776	6.557763	2.713204	2.713204	6.88E-18	2141	3032	2337	2503.333	169	195	178	180.6667
ybeK	4407	1261	-2495.33	-13.8847	1.37E-11	-6.53791	6.537912	2.70883	2.70883	9.23E-11	2042	4575	1450	2689	224	189	168	193.6667
SEN2510	1086	813	-918.667	-13.939	2.38E-24	-6.62258	6.622578	2.727393	2.727393	5.4E-23	932	1153	884	989.6667	71	67	75	71
SEN2179	1400	898	-1114.33	-14.0078	1.71E-17	-6.50189	6.501893	2.70086	2.70086	2.11E-16	1157	1466	977	1200	66	112	79	85.66667
yhdG	1023	892	-950.333	-14.078	1.2E-25	-6.72709	6.727086	2.749982	2.749982	3.04E-24	966	1089	1014	1023	78	66	74	72.66667
yhbT	8574	7541	-7857.67	-14.184	0	-6.77822	6.778222	2.760907	2.760907	0	8152	9091	8118	8453.667	577	517	694	596
seqA	2449	2118	-2303.33	-14.2122	6.92E-23	-6.78119	6.781193	2.761539	2.761539	1.36E-21	2548	2600	2285	2477.667	205	151	167	174.3333
rbsR	3601	3014	-3252.67	-14.2762	0	-6.7804	6.780399	2.76137	2.76137	0	3394	3837	3262	3497.667	248	236	251	245
hypB	17059	13787	-14915	-14.3727	8.88E-16	-6.8076	6.807603	2.767147	2.767147	9.48E-15	14946	18079	15066	16030.33	1167	1159	1020	1115.333
hybB	6170	5480	-5749	-14.4114	2.22E-16	-6.85173	6.85173	2.776468	-2.77647	2.45E-15	5928	6051	6554	6177.667	454	448	384	428.6667

Feature ID	Experiment - Range (original values)	Experiment - IQR (original values)	Experiment - Difference (original values)	Experiment - Fold Change (original values)	EDGE test: WT H202 vs WT NT , tagwise dispersions - P-value	EDGE test: WT H202 vs WT NT , tagwise dispersions - Fold change	WT H202 vs WT NT ABS|FC|	WT H202 vs WT NT Log2FC	WT H202 vs WT NT Log2FC +-	EDGE test: WT H202 vs WT NT , tagwise dispersions - FDR p-value correction	WT NT - WT.1.S22 - Expression values	WT NT - WT.2.S23 - Expression values	WT NT - WT.3.S24 - Expression values	WT NT - Means	WT H202 - WT.1.H2O2 - Expression values	WT H202 - WT.2.H2O2 - Expression values	WT H202 - WT.3.H2O2 - Expression values	WT H202 - Means
SEN2830	1034	596	-778.333	-14.4195	1.71E-20	-6.95132	6.951325	2.797288	2.797288	2.8E-19	658	1077	774	836.3333	62	43	69	58
hscC	1523	777	-1026	-14.6195	1.41E-18	-6.9463	6.946301	2.796245	2.796245	1.92E-17	861	1593	850	1101.333	73	70	83	75.33333
SEN1821	3836	2442	-3147	-14.6628	0	-7.04795	7.047948	2.817203	2.817203	0	2671	4045	3416	3377.333	253	209	229	230.3333
dmsB1	862	609	-663	-14.7172	1.12E-05	-7.47842	7.478424	2.902734	-2.90273	3.29E-05	621	642	871	711.3333	124	12	9	48.33333
aegA	3367	2050	-2616.33	-14.722	3.13E-16	-6.80832	6.808318	2.767298	-2.7673	3.43E-15	3527	2216	2678	2807	160	246	166	190.6667
rna-AM93	383	223	-297.667	-14.7385	8.58E-09	-7.12159	7.121594	2.8322	2.8322	4.09E-08	389	337	232	319.3333	50	9	6	21.66667
rna-AM93	919	537	-710.333	-14.7484	1.33E-11	-7.19282	7.192821	2.846558	-2.84656	9.01E-11	934	772	580	762	97	15	43	51.66667
ftn	1907	1678	-1807	-14.7589	1.32E-19	-6.96674	6.966742	2.800484	-2.80048	2.01E-18	1805	1991	2019	1938.333	127	155	112	131.3333
yfeD	1600	1334	-1479.67	-15.0475	1.14E-24	-7.17572	7.175716	2.843123	2.843123	2.66E-23	1689	1637	1429	1585	132	89	95	105.3333
SEN4301	3361	619	-1749.67	-15.1102	0.000124	-7.42938	7.429381	2.893242	2.893242	0.00031	3381	658	1582	1873.667	313	20	39	124
nmpC	208607	164712	-186550	-15.1831	4.41E-10	-7.02638	7.026382	2.812782	-2.81278	2.47E-09	177615	205685	215808	199702.7	7201	19355	12903	13153
mdtJ	739	658	-682.333	-15.2153	3.94E-17	-7.42648	7.426482	2.892679	2.892679	4.66E-16	762	697	732	730.3333	82	23	39	48
coaA	1060	846	-927.333	-15.3402	1.13E-24	-7.36319	7.363191	2.880331	-2.88033	2.66E-23	951	906	1119	992	75	60	59	64.66667
melA	15479	3728	-7781.67	-15.3573	1.78E-09	-7.0736	7.073604	2.822445	2.822445	9.12E-09	15903	4873	4195	8323.667	735	424	467	542
ygcY	17698	2383	-9071.33	-15.5141	2.02E-06	-7.47982	7.479824	2.903004	2.903004	6.72E-06	17937	2832	8320	9696.333	1187	239	449	625
SEN4262	571	372	-482.667	-15.6263	3.89E-17	-7.50864	7.508641	2.908552	2.908552	4.61E-16	564	589	394	515.6667	59	18	22	33
rbsK	7050	6098	-6503.67	-15.7253	0	-7.5175	7.517499	2.910253	-2.91025	0	6871	7422	6543	6945.333	445	372	508	441.6667
fliL	788	487	-604	-15.7317	8.71E-23	-7.5272	7.527197	2.912113	-2.91211	1.68E-21	527	824	584	645	47	36	40	41
eutQ	1902	173	-767.333	-15.7564	2.37E-06	-7.05784	7.057844	2.819228	2.819228	7.75E-06	1944	289	225	819.3333	62	42	52	52
yebN	446	313	-394.333	-15.7875	3.04E-26	-7.48064	7.480644	2.903162	2.903162	7.92E-25	458	466	339	421	26	20	34	26.66667
ydfH	1674	1224	-1462	-15.8176	1.49E-23	-7.54383	7.54383	2.915297	2.915297	3.12E-22	1751	1624	1307	1560.667	136	77	83	98.66667
cbiD	5471	3746	-4570.67	-16.0022	7.33E-14	-7.7425	7.7425	2.9528	2.9528	6.42E-13	4960	4007	5659	4875.333	465	261	188	304.6667
aidB	5566	3821	-4620	-16.0489	0	-7.61524	7.615242	2.92889	-2.92889	0	5818	4808	4155	4927	334	252	335	307
ydeV	9463	531	-3460.33	-16.0887	0.000146	-7.24468	7.244678	2.856922	2.856922	0.000362	9607	752	710	3689.667	365	144	179	229.3333
fruR	3878	3596	-3689.33	-16.0996	0	-7.60835	7.608348	2.927583	2.927583	0	3856	4084	3861	3933.667	260	267	206	244.3333
rpsU	2341	2111	-2186.67	-16.1152	4.32E-24	-7.64634	7.646341	2.93477	2.93477	9.57E-23	2259	2473	2262	2331.333	154	148	132	144.6667
yhiP	40928	33015	-36692.3	-16.3034	1.11E-15	-7.91943	7.91943	2.985397	2.985397	1.17E-14	39447	42386	35437	39090	3313	1458	2422	2397.667
yfeC	1706	1294	-1508	-16.4403	1.27E-26	-7.84252	7.842521	2.971317	2.971317	3.36E-25	1782	1646	1389	1605.667	122	76	95	97.66667
nrfB	3336	992	-2279.67	-16.4729	3.4E-13	-8.03171	8.031712	3.005708	3.005708	2.82E-12	1129	3461	2691	2427	137	125	180	147.3333
flgJ	1106	758	-880.667	-16.5412	4.91E-23	-7.7751	7.775097	2.958861	-2.95886	9.85E-22	845	1156	811	937.3333	53	67	50	56.66667
hypD	14607	10793	-12457.7	-16.5656	2.22E-16	-7.81491	7.814914	2.96623	-2.96623	2.45E-15	11545	15341	12888	13258	752	915	734	800.3333
gpt	842	589	-687.333	-16.6212	4.35E-25	-7.82442	7.824421	2.967984	2.967984	1.05E-23	875	636	683	731.3333	52	47	33	44
yeeI	4295	2161	-2908	-16.6344	6.93E-19	-7.876	7.875998	2.977463	2.977463	9.72E-18	4427	2523	2332	3094	255	132	171	186
potA	648	550	-589.333	-16.646	1.99E-25	-7.86717	7.867167	2.975844	-2.97584	4.92E-24	614	677	590	627	29	40	44	37.66667
yjjI	9556	6583	-7812.67	-16.7832	0	-8.01647	8.016474	3.002968	3.002968	0	7055	10008	7860	8307.667	561	472	452	495
fliR	421	245	-331	-17.2787	5.77E-23	-8.23581	8.235807	3.04191	-3.04191	1.15E-21	357	433	264	351.3333	30	12	19	20.33333
uppS	2516	2364	-2433.67	-17.37	1.87E-26	-8.26931	8.269306	3.047766	3.047766	4.93E-25	2650	2593	2504	2582.333	172	140	134	148.6667

Feature ID	Experiment - Range (original values)	Experiment - IQR (original values)	Experiment - Difference (original values)	Experiment - Fold Change (original values)	EDGE test: WT H202 vs WT NT , tagwise dispersions - P-value	EDGE test: WT H202 vs WT NT , tagwise dispersions - Fold change	WT H202 vs WT NT ABS|FC|	WT H202 vs WT NT Log2FC	WT H202 vs WT NT Log2FC +-	EDGE test: WT H202 vs WT NT , tagwise dispersions - FDR p-value correction	WT NT - WT.1.S22 - Expression values	WT NT - WT.2.S23 - Expression values	WT NT - WT.3.S24 - Expression values	WT NT - Means	WT H202 - WT.1.H2O2 - Expression values	WT H202 - WT.2.H2O2 - Expression values	WT H202 - WT.3.H2O2 - Expression values	WT H202 - Means
ydeY	3048	64	-1056.67	-17.5969	0.000616	-7.78492	7.784918	2.960682	2.960682	0.001352	3095	150	116	1120.333	92	47	52	63.66667
ygjR	5910	4868	-5367.33	-17.8255	0	-8.58322	8.583222	3.101519	3.101519	0	6139	5782	5138	5686.333	458	229	270	319
nanA	38310	11980	-24268	-17.845	4.49E-13	-8.49844	8.498435	3.087197	3.087197	3.69E-12	24333	39269	13524	25708.67	1544	959	1819	1440.667
flgG	1873	1433	-1607.67	-17.9824	3.02E-21	-8.43908	8.439084	3.077086	3.077086	5.19E-20	1520	1951	1636	1702.333	87	119	78	94.66667
SEN3176	1842	895	-1281	-18.0044	3.52E-19	-8.61819	8.618194	3.107386	3.107386	5.12E-18	1877	1213	979	1356.333	107	35	84	75.33333
SEN3094	385	311	-351	-18.2623	8.53E-30	-8.70261	8.702607	3.121448	3.121448	2.72E-28	381	399	334	371.3333	23	14	24	20.33333
SEN1482	547	452	-483.667	-18.2738	3.88E-29	-8.61163	8.611628	3.106286	3.106286	1.18E-27	569	482	484	511.6667	22	30	32	28
b2145	172	128	-151	-18.4231	2.08E-22	-8.64719	8.647192	3.112232	3.112232	3.91E-21	177	164	138	159.6667	11	10	5	8.666667
yneA	9484	270	-3317	-18.4579	0.000434	-8.34244	8.342435	3.060469	3.060469	0.000979	9573	521	427	3507	324	89	157	190
menD	7932	5692	-6621	-18.6403	0	-8.79336	8.793361	3.136415	3.136415	0	6061	8266	6662	6996.333	334	423	369	375.3333
nrfA	12124	3992	-8462.67	-18.7912	2.63E-13	-9.12444	9.124439	3.189736	3.189736	2.2E-12	4460	12558	9797	8938.333	468	434	525	475.6667
focA	2050	1846	-1930	-19.265	3.31E-27	-9.23164	9.231638	3.206587	-3.20659	9.1E-26	2021	1941	2145	2035.667	127	95	95	105.6667
garK	13019	2643	-8121.33	-19.4436	8.58E-09	-9.52731	9.527306	3.252068	-3.25207	4.09E-08	13197	2997	9491	8561.667	789	178	354	440.3333
SEN1995	1475	527	-1081.33	-19.5371	3.29E-17	-9.57517	9.575173	3.259299	3.259299	3.93E-16	583	1521	1315	1139.667	73	46	56	58.33333
SEN1861	628	481	-565.667	-19.8556	6.5E-26	-9.53119	9.531187	3.252656	3.252656	1.66E-24	510	628	649	595.6667	40	29	21	30
nanK	6575	2695	-4223.67	-20.554	4.44E-16	-9.87536	9.875359	3.303833	-3.30383	4.83E-15	3713	6727	2879	4439.667	312	152	184	216
ydeZ	3762	121	-1325	-20.5813	0.000242	-9.22343	9.223429	3.205303	3.205303	0.000574	3802	214	162	1392.667	122	41	40	67.66667
glpB	24897	17876	-21319	-20.7398	0	-9.93443	9.934428	3.312437	3.312437	0	25693	22714	18790	22399	1530	796	914	1080
rna-AM93	29	23	-26.6667	-21	1.7E-08	-9.48364	9.483644	3.245442	3.245442	7.82E-08	30	24	30	28	1	2	1	1.333333
SEN3423	1524	167	-639.667	-21.2	4.42E-08	-9.48884	9.488842	3.246232	3.246232	1.89E-07	1550	197	267	671.3333	39	30	26	31.66667
yhbU	1421	523	-1104.33	-21.5776	1.42E-14	-10.2896	10.28956	3.36311	3.36311	1.3E-13	572	1443	1459	1158	49	74	38	53.66667
mglB	5445	3515	-4277	-21.6286	6.47E-21	-10.383	10.38302	3.376155	3.376155	1.09E-19	4163	5607	3683	4484.333	292	162	168	207.3333
fliM	2215	1238	-1675.67	-21.7727	2.46E-24	-10.3658	10.36578	3.373757	3.373757	5.57E-23	1321	2287	1661	1756.333	72	83	87	80.66667
caiF	3923	2687	-3195	-21.8824	5.37E-28	-10.278	10.27796	3.361482	-3.36148	1.52E-26	4064	3141	2839	3348	166	152	141	153
SEN4218	72587	23822	-51765.3	-22.2153	9.66E-11	-10.9055	10.90551	3.446985	3.446985	5.8E-10	73846	25389	63381	54205.33	4494	1259	1567	2440
napF	3371	651	-1896.67	-22.391	1.1E-12	-10.7731	10.77313	3.429366	-3.42937	8.6E-12	738	3452	1766	1985.333	81	87	98	88.66667
cdaR	15080	7282	-10048.7	-22.4105	2.22E-16	-10.7216	10.72163	3.422452	3.422452	2.45E-15	15364	7671	8519	10518	735	284	389	469.3333
frdA	101644	84160	-94065.7	-22.4582	0	-10.8522	10.85223	3.43992	-3.43992	0	87919	102427	105002	98449.33	6034	3759	3358	4383.667
flgI	1130	745	-887	-22.6341	8.98E-30	-10.6782	10.67817	3.416593	-3.41659	2.84E-28	831	1169	784	928	39	45	39	41
yadI	1261	608	-925.667	-22.6953	3.43E-28	-10.7167	10.71666	3.421784	3.421784	9.88E-27	1289	966	650	968.3333	58	28	42	42.66667
hypO	14105	10880	-12068.3	-22.7187	0	-10.762	10.76198	3.427872	3.427872	0	14585	11891	11396	12624	671	516	480	555.6667
yobF	8483	6659	-7638.67	-22.7626	0	-10.9253	10.92525	3.449594	3.449594	0	7007	8151	8811	7989.667	348	328	377	351
fliJ	663	421	-520.333	-22.9859	4.76E-30	-11.0244	11.02442	3.462631	-3.46263	1.54E-28	447	681	504	544	27	18	26	23.66667
SEN2914	318	159	-253	-23.3235	5.81E-19	-11.1011	11.10112	3.472633	3.472633	8.2E-18	170	326	297	264.3333	8	15	11	11.33333
pipD	2261	1174	-1572	-23.4571	1.31E-25	-11.1317	11.1317	3.476602	-3.4766	3.27E-24	2306	1381	1239	1642	100	45	65	70
spr	4538	3877	-4197	-23.5242	6.27E-23	-11.2552	11.25522	3.492523	3.492523	1.24E-21	4674	4044	4432	4383.333	256	167	136	186.3333
dmsA	43375	38361	-39558	-23.7214	0	-11.4894	11.48937	3.522228	3.522228	0	44495	39812	39590	41299	2874	1229	1120	1741

Feature ID	Experiment - Range (original values)	Experiment - IQR (original values)	Experiment - Difference (original values)	Experiment - Fold Change (original values)	EDGE test: WT H202 vs WT NT , tagwise dispersions - P-value	EDGE test: WT H202 vs WT NT , tagwise dispersions - Fold change	WT H202 vs WT NT ABS|FC|	WT H202 vs WT NT Log2FC	WT H202 vs WT NT Log2FC +-	EDGE test: WT H202 vs WT NT , tagwise dispersions - FDR p-value correction	WT NT - WT.1.S22 - Expression values	WT NT - WT.2.S23 - Expression values	WT NT - WT.3.S24 - Expression values	WT NT - Means	WT H202 - WT.1.H2O2 - Expression values	WT H202 - WT.2.H2O2 - Expression values	WT H202 - WT.3.H2O2 - Expression values	WT H202 - Means
cbiC	2929	2204	-2553	-23.7946	4.88E-29	-11.4839	11.48394	3.521546	3.521546	1.47E-27	2671	2302	3022	2665	145	93	98	112
nanE	9018	3658	-6076.67	-23.9308	0	-11.378	11.37802	3.508177	3.508177	0	5888	9227	3910	6341.667	334	209	252	265
glpA	73340	51078	-62333.7	-23.9562	0	-11.3722	11.37222	3.507443	3.507443	0	75519	66216	53412	65049	3633	2334	2179	2715.333
tdcB	121064	29720	-74294	-24.1301	8.36E-11	-12.1707	12.17074	3.605345	3.605345	5.08E-10	32091	77325	123102	77506	5227	2371	2038	3212
dmsA1	11180	6742	-8722.33	-24.1771	0	-11.6312	11.63125	3.539934	-3.53993	0	11398	8815	7083	9098.667	570	218	341	376.3333
flgF	2080	1553	-1742.67	-24.2356	2.92E-21	-11.2424	11.24243	3.490882	3.490882	5.07E-20	1616	2134	1703	1817.667	54	108	63	75
SEN1805	155	54	-102	-24.5385	4.79E-15	-11.1902	11.19022	3.484167	3.484167	4.67E-14	158	102	59	106.3333	5	5	3	4.333333
ygiM	3726	1892	-2965.67	-24.7888	3.34E-20	-12.2748	12.27485	3.617633	3.617633	5.36E-19	2006	3459	3806	3090.333	180	80	114	124.6667
nupG	6243	1225	-3221.33	-24.9208	2.03E-13	-11.821	11.82101	3.563281	3.563281	1.71E-12	2354	6348	1366	3356	158	105	141	134.6667
flgC	330	167	-263.667	-24.9697	1.62E-19	-11.9346	11.9346	3.577078	3.577078	2.44E-18	179	308	337	274.6667	7	14	12	11
cspC	27811	20779	-25332.7	-25.2418	0	-12.0929	12.09292	3.596091	-3.59609	0	21809	28830	28494	26377.67	1086	1030	1019	1045
SEN0310	3720	2418	-3053.33	-25.492	2.57E-30	-12.0116	12.01164	3.586361	3.586361	8.45E-29	3827	3179	2528	3178	110	107	157	124.6667
SEN0991	1780	987	-1392	-25.5647	1.57E-23	-12.5615	12.56153	3.65094	3.65094	3.26E-22	1042	1811	1493	1448.667	84	31	55	56.66667
garL	16204	3614	-10712.7	-25.5891	1.87E-07	-12.8282	12.82817	3.681244	-3.68124	7.39E-07	16290	3871	13284	11148.33	964	86	257	435.6667
cbiA	7094	5922	-6308.67	-25.7399	0	-12.2853	12.2853	3.618861	-3.61886	0	7305	6200	6186	6563.667	290	211	264	255
rna-AM93	238	161	-209	-26.08	7.39E-30	-12.2738	12.27382	3.617513	-3.61751	2.37E-28	239	243	170	217.3333	9	5	11	8.333333
napD	1228	250	-662.667	-26.1646	6.1E-13	-12.3421	12.3421	3.625516	3.625516	4.92E-12	278	1246	543	689	18	33	28	26.33333
ydeW	5605	568	-2242.67	-27.3843	3.2E-08	-12.2489	12.24891	3.614581	3.614581	1.41E-07	5674	652	657	2327.667	102	69	84	85
rna-AM93	1082	624	-820.333	-27.75	8.92E-34	-13.1131	13.11312	3.712939	-3.71294	3.63E-32	1104	799	650	851	44	22	26	30.66667
flgE	5936	4194	-4815.67	-28.1051	4.16E-21	-13.0841	13.08411	3.709744	3.709744	7.1E-20	4350	6077	4553	4993.333	141	236	156	177.6667
dmsA3	6508	5806	-6021.33	-28.4529	1.33E-13	-14.1855	14.18553	3.826348	3.826348	1.13E-12	5933	6195	6594	6240.667	445	86	127	219.3333
SEN3658	286	206	-232.667	-28.92	3.69E-28	-13.6379	13.63788	3.769547	3.769547	1.06E-26	215	291	217	241	5	9	11	8.333333
citE	1704	506	-949	-29.1881	7.63E-20	-13.28	13.28004	3.731187	-3.73119	1.2E-18	1728	677	543	982.6667	40	37	24	33.66667
malF	25173	21036	-22564.7	-29.3357	0	-13.9327	13.93274	3.800408	3.800408	0	22294	25947	21842	23361	809	774	806	796.3333
flgH	999	718	-825.333	-29.4598	4.64E-32	-13.7979	13.79794	3.786381	3.786381	1.71E-30	796	1020	747	854.3333	21	37	29	29
SEN1558	2401	1866	-2103	-31.7756	6.32E-37	-15.0334	15.03337	3.910097	3.910097	2.95E-35	2115	2463	1936	2171.333	73	70	62	68.33333
yggM	2494	1722	-2031.67	-31.7828	3.34E-35	-15.2541	15.25405	3.931121	3.931121	1.47E-33	1793	2548	1952	2097.667	73	54	71	66
rna-AM93	207	154	-177.333	-32.2941	1.78E-27	-15.2527	15.2527	3.930993	3.930993	4.97E-26	208	183	158	183	12	1	4	5.666667
cspA	32582	21806	-24940	-32.3842	1.59E-11	-16.1417	16.14174	4.012725	-4.01272	1.06E-10	22166	32870	22168	25734.67	1736	288	360	794.6667
eutP	832	116	-352.667	-33.0606	7.04E-10	-14.8639	14.86389	3.893739	3.893739	3.86E-09	840	125	126	363.6667	16	8	9	11
rna-AM93	214	104	-149.667	-33.0714	1.52E-22	-15.4125	15.41252	3.946031	3.946031	2.9E-21	216	109	138	154.3333	5	2	7	4.666667
rna-AM93	647	411	-529.667	-33.4286	1.55E-38	-15.7345	15.73451	3.975861	3.975861	8E-37	657	556	425	546	25	14	10	16.33333
eutS	639	66	-265.333	-34.1667	4.59E-08	-15.656	15.65599	3.968643	3.968643	1.96E-07	640	105	75	273.3333	14	1	9	8
rna-AM93	945	659	-817.667	-36.0429	1E-30	-17.2023	17.20226	4.104526	4.104526	3.44E-29	958	891	674	841	42	15	13	23.33333
rna-AM93	1097	556	-838.333	-36.4225	4.62E-35	-17.2415	17.24149	4.107813	4.107813	2.01E-33	1109	894	583	862	32	12	27	23.66667
rnpA	908	790	-847.667	-37.3286	2.84E-54	-17.789	17.78899	4.152913	4.152913	2.33E-52	872	927	814	871	27	19	24	23.33333
hypA	2277	1859	-2030.67	-37.479	1.01E-39	-17.8132	17.81321	4.154876	4.154876	5.53E-38	1916	2327	2016	2086.333	60	57	50	55.66667

Feature ID	Experiment - Range (original values)	Experiment - IQR (original values)	Experiment - Difference (original values)	Experiment - Fold Change (original values)	EDGE test: WT H202 vs WT NT , tagwise dispersions - P-value	EDGE test: WT H202 vs WT NT , tagwise dispersions - Fold change	WT H202 vs WT NT ABS|FC|	WT H202 vs WT NT Log2FC	WT H202 vs WT NT Log2FC +-	EDGE test: WT H202 vs WT NT , tagwise dispersions - FDR p-value correction	WT NT - WT.1.S22 - Expression values	WT NT - WT.2.S23 - Expression values	WT NT - WT.3.S24 - Expression values	WT NT - Means	WT H202 - WT.1.H2O2 - Expression values	WT H202 - WT.2.H2O2 - Expression values	WT H202 - WT.3.H2O2 - Expression values	WT H202 - Means
ego	6354	293	-2330.33	-39.6243	7.42E-07	-17.3262	17.32622	4.114885	-4.11488	2.68E-06	6409	412	351	2390.667	68	58	55	60.33333
dmsA2	1999	1375	-1613.33	-39.72	3.27E-38	-18.7215	18.72145	4.226621	4.226621	1.66E-36	1513	2033	1419	1655	44	47	34	41.66667
ynfK	3914	3174	-3515.33	-40.3507	9.97E-40	-19.2835	19.28352	4.269296	4.269296	5.52E-38	3568	3989	3257	3604.667	110	75	83	89.33333
citD	455	107	-237	-40.5	1.12E-16	-18.3546	18.35459	4.198069	-4.19807	1.28E-15	459	157	113	243	6	4	8	6
orfX	332	270	-300.333	-43.9048	2.47E-31	-21.001	21.00097	4.392384	4.392384	8.67E-30	275	313	334	307.3333	14	5	2	7
SEN0990	7030	4282	-5262.67	-44.3736	1.45E-14	-22.1473	22.14731	4.46906	4.46906	1.32E-13	4716	7057	4379	5384	240	27	97	121.3333
yjfN	12743	10323	-11313.3	-45.366	0	-21.7662	21.76619	4.444017	4.444017	0	12925	11167	10613	11568.33	290	182	293	255
rna-AM93	1200	658	-962.667	-45.4308	3.63E-41	-21.5223	21.52233	4.427762	-4.42776	2.09E-39	1212	1063	678	984.3333	33	12	20	21.66667
rna-AM93	1084	924	-997.667	-45.6716	2.42E-53	-21.8787	21.87874	4.451458	4.451458	1.91E-51	1097	1016	947	1020	31	13	23	22.33333
yjfO	7401	6419	-6848.67	-46.2555	1.4E-33	-22.3572	22.35725	4.482671	4.482671	5.63E-32	7499	6597	6904	7000	178	98	178	151.3333
citC	1946	302	-916.333	-49.2281	4.9E-16	-22.1087	22.10872	4.466544	-4.46654	5.32E-15	1960	526	320	935.3333	25	18	14	19
garD	32294	6892	-20039.3	-52.2515	1.77E-13	-25.4678	25.4678	4.670602	-4.6706	1.5E-12	32479	7104	21708	20430.33	776	185	212	391
gudP	24633	4213	-13439.3	-56.8421	1.01E-13	-27.4218	27.42179	4.777251	4.777251	8.71E-13	24725	4396	11919	13680	447	92	183	240.6667
pocR	2492	1772	-2028.33	-67.8681	1.74E-66	-32.0313	32.03127	5.001409	-5.00141	1.88E-64	2519	1857	1800	2058.667	36	27	28	30.33333
SEN0957	340	235	-292.667	-80.8182	4.78E-37	-38.2088	38.2088	5.255833	5.255833	2.25E-35	239	309	341	296.3333	6	4	1	3.666667
tdcA	21949	15012	-19289.3	-270.153	3.85E-62	-131.096	131.0963	7.034484	7.034484	3.63E-60	15073	21003	22007	19361	96	61	58	71.66667
