# Supplementary figures and images for: Insights into the Oxidative Stress Response of Salmonella enterica serovar Enteritidis Revealed by the Next Generation Sequencing Approach
Source: Antioxidants (Basel). 2020 Sep 10;9(9):849. doi: 10.3390/antiox9090849 (PMC7555449; doi:10.3390/antiox9090849)

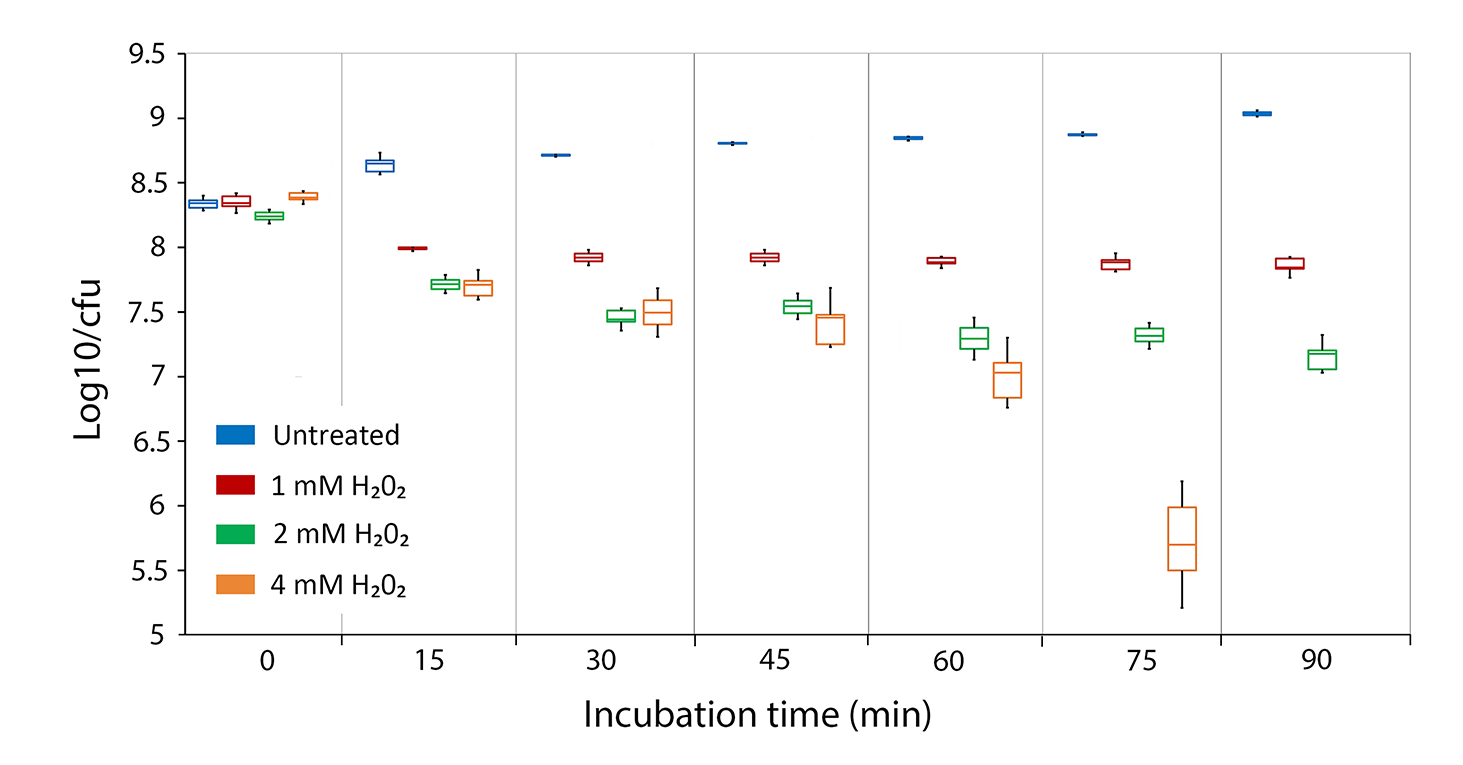

Supplement: Supplementary file 1 [file antioxidants-09-00849-s001.zip › antioxidants-897047-supplementary/antioxidants-897047-suppl-revised.tif]
